# Organic planar π-conjugated anions enable structural design of anion-π^+^ NIR-II phototheranostic agents

**DOI:** 10.1038/s41467-026-75247-7

**Published:** 2026-07-08

**Authors:** Ying Zhang, Mingyang Han, Guoyu Jiang, Lingxiu Liu, Chunbin Li, Lina Feng, Jianye Gong, Jianguo Wang, Ben Zhong Tang

**Affiliations:** 1 College of Chemistry and Chemical Engineering, Institute of Green Chemistry and Environment, Institutes of Biomedical Sciences, Inner Mongolia Key Laboratory of Synthesis and Application of Organic Functional Molecules, Inner Mongolia University, Hohhot, P. R. China; 2 Guangdong Basic Research Center of Excellence for Aggregate Science, School of Science and Engineering, The Chinese University of Hong Kong (Shenzhen), Longgang, Shenzhen, Guangdong, P. R. China

**Keywords:** Photochemistry, Optical materials

## Abstract

Anion-π^+^ phototheranostic agents (PTAs) have attracted considerable interest for their exceptional luminescence and reactive oxygen species (ROS). However, current development is constrained by limited structural diversity and lack of second near-infrared (NIR-II) emission and photothermal performance, with the role of organic π-conjugated anions remaining unexplored. Here, we propose an organic π-conjugated anion-exchange strategy to construct advanced NIR-II PTAs. DTPAP-2B (with Br^-^) and DTPAP-2P (with pentacyanocyclopentadiene anion ((CCN)_5_^-^)), were synthesized. Upon nanoformulation, DTPAP-2P NPs exhibit pronounced absorption redshift, intense NIR-II emission, a large Stokes shift (340 nm), superior ROS generation, and high photothermal conversion efficiency (72%) compared to DTPAP-2B NPs. Single-crystal and theoretical analyses reveal that the planar (CCN)_5_^-^ anion enhances dual anion-π^+^ interactions, promoting intramolecular charge transfer while suppressing intermolecular π-π stacking. This enables high-resolution NIR-II angiography and effective NIR-II imaging-guided synergistic photodynamic/photothermal therapy in female mice model. This work establishes organic π-conjugated anion exchange as a powerful strategy for developing robust NIR-II PTAs.

## Abstract (web summary)

Current anion-π⁺ phototheranostic agents are limited by narrow structural tunability and lack efficient NIR-II emission and photothermal performance. Here, authors introduce an organic π-conjugated anion exchange strategy that strengthens anion–π⁺ interactions, enabling high-performance NIR-II agents with enhanced emission, ROS generation, and synergistic phototherapy.

## Introduction

Phototheranostics^1,2^ that integrating diagnostic modalities (fluorescence^3,4^/photoacoustic imaging^5^) and light-triggered precision therapeutics (photodynamic therapy (PDT)^6,7^/photothermal therapy (PTT)^8^) has become one of the most promising research hotspots for its non-invasiveness and reduced drug resistance possibilities. Wherein, the second near-infrared (NIR-II, 1000‒1700 nm) phototheranostics stands out from in vivo deep tissue therapy due to its advantages including deeper tissue penetration, less light scattering, minimal tissue autofluorescence, excellent spatial-temporal resolution, enhanced signal-to-background ratio, good tolerance of biological tissues, and higher maximum permissible exposure to excitation light^9,10^. As a core element of phototheranostic systems, the design and development of NIR-II phototheranostic agents (PTAs) with high performance is essential^11–13^. Currently, the following design strategies are mainly used to enhance the performance of NIR-II PTAs:(1) extending the conjugation length of the molecules to improve the photophysical properties, including absorption cross-section and photothermal conversion efficiency^14–16^;(2) strengthening donor (D)-acceptor (A) interactions to lower the energy band gap for redshifted absorption/emission spectra and to tune the singlet–triplet energy gap (ΔE_ST_) for promoting ROS production^17,18^;(3) fabricating *J*-aggregates to improve the NIR-II brightness^19,20^. However, these molecular strategies still suffer from cumbersome synthesis, low yield, difficult purification, high cost, and limited efficacy. Worse still, most of the reported NIR-II molecules tend to form strong π-π stacking due to the planarity and severe hydrophobicity, which will further largely impair the phototheranostic performances and hinder their clinical applications^21–25^. Therefore, the development of facile and effective design strategies for high-performance NIR-II PTAs remains a great challenge^26–36^.

In recent years, the emergence of organic NIR-II PTAs with aggregation-induced emission (AIE) feature, which display aggregation-enhanced emission as well as reactive oxygen species (ROS) production has provided a facile way to cirvumvent the aggregation-caused quenching problem and thus attracted increasing interest^37–46^. In 2017, we pioneered the exploitation of AIE luminogens by introducing anion-π^+^ interactions, which has greatly inspired the research interest for developing highly efficient anion-π^+^ type PTAs in the past few years^47^. The original strategy can not only effectively inhibit the detrimental aggregation-caused quenching of fluorescence and ROS generation, but also improve the water solubility and tumor targeting capability in view of the inherent charges in these anion-π^+^ type PTAs^48–51^. Notably, most of the current research efforts on anion-π^+^ type PTAs has been devoted to modifying the π^+^ core for regulating their performances^52–58^, and NIR-II PTAs with anion-π^+^ interactions haven’t yet been successfully developed. Moreover, most reported anion-π^+^ PSs require excitation by ultraviolet or white light, both of which suffer from poor tissue penetration, thereby compromising the in vivo therapeutic efficacy (Supplementary Table 1). Besides, the anions, as another indispensable component in anion-π^+^ type PTAs, have been largely overlooked. Recently, we fabricated highly water soluble AIE-active NIR-I (700‒900 nm) PTAs with anion-π^+^ interactions by simply exchanging inorganic counterions. This provides a facile but very effective method to obtain various anion-π^+^ type PTAs with NIR-I emission^59^. Compared to typical inorganic anions, organic π-conjugated anions are characterized with their large π-conjugation as well as good planarity, which facilitates the formation of electrostatically induced D-A interactions and π-π interactions with cationic π^+^ systems. In addition, intermolecular π-π interactions can be further prohibited to diminish the aggregation-caused quenching of the excited states^60–64^. Therefore, we can infer that the introduction of organic planar π-conjugated anions for constructing anion-π^+^ type NIR-II PTAs will be an innovative, facile and effective strategy.

**Fig. 1:**
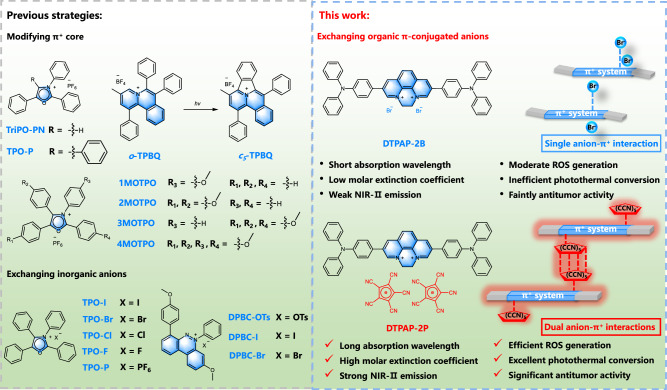
Schematic of strategies for constructing anion-π^+^ type PTAs. Schematic illustration of organic planar π-conjugated anion exchange strategy for structural design of anion-π^+^ type NIR-II PTAs^47,48,52,53,59^.

Herein, as a proof of concept, an anion-π^+^ type compound DTPAP-2B, with phenanthroline as the electron acceptor, triphenylamine (TPA) as the electron donor and Br^-^ as the counterion, is synthesized in high yield^58^. Then, by simply exchanging the inorganic Br^-^ with organic planar π-conjugated pentacyanocyclopentadiene anion ((CCN)_5_^-^), DTPAP-2P is obtained (Fig. 1). DTPAP-2P and DTPAP-2B exhibit similar absorption spectra, negligible NIR-II emission and ROS generation in pure DMSO solution. While in stark contrast, after encapsulation into Pluronic F127 to form nanoparticles (NPs), DTPAP-2P NPs present redshifted absorption with a much higher molar extinction coefficient (1.16 ×10^4 ^L mol^−1^ cm^−1^) at 660 nm, remarkable NIR-II emission, large Stokes shift (340 nm), more efficient ROS production capacity and a photothermal conversion efficiency (PCE) of up to 72% compared with DTPAP-2B NPs. As a result, real-time NIR-II imaging of blood vessels, including the capillary-level micro-vessel in live mice, is achieved with high resolution. Additionally, DTPAP-2P NPs are successfully applied in precise synergistic PDT/PTT cancer theranostics guided by NIR-II fluorescence imaging. This work demonstrates the facile construction of high-performance anion-π^+^ type NIR-II PTAs through a simple strategy of introducing organic planar π-conjugated anions, which will not only pave the way for the exploration of robust NIR-II PTAs, but also promote the early diagnosis and treatment research of cancer.

## Results

### Design and characterization

Firstly, as shown in Supplementary Fig. 1, compound DTPAP with a D-A-D structure was synthesized according to our previous reports^65,66^. Further reaction with 1,2-dibromoethane gave DTPAP-2B with Br^-^ as the counterions^58^. Afterward, DTPAP-2P was obtained by exchanging Br^-^ of DTPAP-2B with organic planar π-conjugated (CCN)_5_^-^. Similarly, DMPHL-2P and DMPHL-2B were also synthesized as control systems to systematically verify the universality and robustness of this design strategy (Supplementary Figs. 2–4). The synthetic details and characterization data were shown in the Supplementary Information. All compounds were fully characterized by ^1^H NMR, ^13^C NMR, 2D NMR spectroscopy, and high-resolution mass spectrometry (HRMS) (Supplementary Figs. 5–19). X-ray photoelectron spectroscopy (XPS) and ion chromatography (Supplementary Figs. 20, 21) evidenced the absence of Br^-^, which further confirmed the successful exchange of Br^-^ to (CCN)_5_^-^ to obtain DTPAP-2P.

**Fig. 2:**
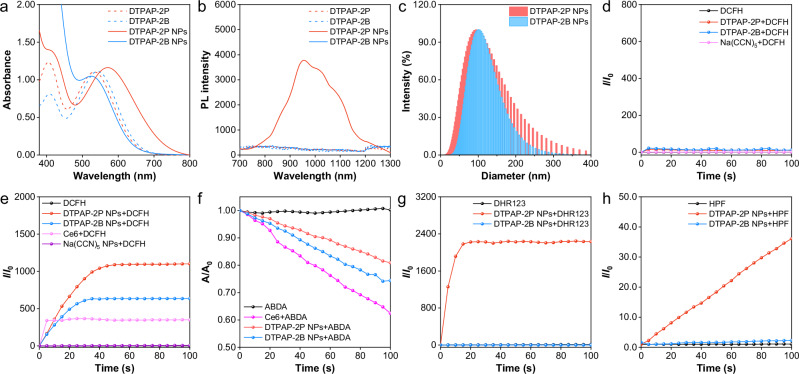
Photophysical properties and ROS generation of DTPAP-2P and DTPAP-2B. **a** Absorption spectra and (**b**) Photoluminescence (PL) spectra of DTPAP-2P, DTPAP-2B in DMSO and DTPAP-2P NPs, DTPAP-2B NPs in PBS. λ_ex_ = 660 nm. **c** DLS of DTPAP-2P NPs and DTPAP-2B NPs in aqueous solution. (**d**) ROS generation of DTPAP-2P, DTPAP-2B and Na(CCN)_5_ (10 µM) in DMSO detected by DCFH upon 660 nm laser irradiation (0.1 W cm^-2^). (**e**) ROS generation of DTPAP-2P NPs, DTPAP-2B NPs, Ce6 and Na(CCN)_5_ NPs (10 µM) in PBS detected by DCFH upon 660 nm laser irradiation (0.1 W cm^-2^). **f** ^1^O_2_ generation of DTPAP-2P NPs, DTPAP-2B NPs and Ce6 in PBS detected by ABDA upon 660 nm laser irradiation (0.1 W cm^-2^). Type I radical ROS generation of DTPAP-2P NPs, DTPAP-2B NPs (10 µM) in PBS detected by (**g**) DHR123 and (**h**) HPF upon 660 nm laser irradiation (0.1 W cm^-2^). Source data are provided as a Source Data file.

In order to explore the photophysical properties of the two compounds, their UV/Vis absorption (Fig. 2a) and photoluminescence (PL) spectra (Fig. 2b) were measured. Both DTPAP-2P and DTPAP-2B exhibited similar absorption spectra and very weak emission in DMSO solution. This indicates that in the solution state, the cations and counter anions exist in a dissociated state, thereby forming negligible anion-π^+^ interactions and thus exerting poor influence on the luminescent properties, which is consistent with previous report^47,67^. Subsequently, the AIE properties of DTPAP-2P and DTPAP-2B were investigated in DMSO/water mixed solution with different water fraction (*f*_w_). As shown in Supplementary Fig. 22, PL intensity of DTPAP-2P was gradually enhanced with the increase of *f*_w_, indicating typical AIE characteristics. Meanwhile, the absorption and emission spectra of DTPAP-2B and DTPAP-2P were further recorded in the solid powder state. Both powders exhibit a broad absorption band ranging from 450 nm to 1050 nm, with absorption maxima at ~575 nm for DTPAP-2B and 660 nm for DTPAP-2P, respectively. Notably, DTPAP-2P displays largely enhanced NIR-II emission compared to DTPAP-2B under the same conditions (Supplementary Fig. 23). This further verified the existence of anion-π^+^ interactions in DTPAP-2P, which helps to prohibit the π-π stacking and intramolecular motion in aggregated state to boost the enhanced emission, favourable for achieving high-resolution imaging in biological environments.

To improve their water solubility and biocompatibility for subsequent biological applications, the corresponding NPs of DTPAP-2B and DTPAP-2P were prepared according to a one-step nanoprecipitation strategy with amphiphilic copolymer Pluronic F127. Dynamic light scattering (DLS) results (Fig. 2c) showed that DTPAP-2B NPs and DTPAP-2P NPs displayed an average hydrodynamic diameter of 102 nm and 96 nm, respectively. Images obtained through transmission electron microscopy (TEM) also verified the spherical morphology of DTPAP-2B NPs and DTPAP-2P NPs as shown in Supplementary Fig. 24. Then the stability of NPs at 4 °C was further monitored. As shown in Supplementary Fig. 25a, during a storage period of up to 7 days, the particle size of DTPAP-2B NPs and DTPAP-2P NPs remained stable, which is beneficial for practical application. Additionally, the absorption and fluorescence spectra of the DTPAP-2P NPs solution remained unchanged during this peroid (Supplementary Fig. 25b‒d). The stability in dulbecco’s modified eagle medium (DMEM) was further assessed. The hydrodynamic diameters of both NPs remained essentially unchanged over the monitored period, indicating robust stability in complex biological environments (Supplementary Fig. 26). These data indicate that the introduction of the hydrophobic organic π-conjugated (CCN)_5_^-^ anions contributes to enhancing the stability of the nanoparticles. To demonstrate the effects induced by the introduction of organic planar π-conjugated anions, photophysical properties of DTPAP-2B NPs and DTPAP-2P NPs were then investigated. Compared with the absorption spectra in DMSO solution, the maximum absorption wavelength of DTPAP-2B NPs displayed slight hypsochromic shift of 17 nm (Fig. 2a). Notably, the maximum absorption peaks of DTPAP-2P in NPs were obviously redshifted from 535 nm to 570 nm, with its molar extinction coefficient at 660 nm calculated to be 1.16 ×10^4 ^L mol^-1^ cm^-1^, which is conducive to better light harvesting capability in longer wavelength window. More surprisingly, under 660 nm laser irradiation, DTPAP-2P NPs presented remarkably enhanced broad fluorescence emission in the near-infrared region of 700 nm‒1300 nm (Fig. 2b) accompanied by a large Stokes shift of 340 nm, favorable for deep tissue NIR-II imaging and for avoiding the interference from excitation light. The NIR-II fluorescence emission quantum yield (QY) of DTPAP-2P NPs was calculated to be 0.62% (Supplementary Table 2) using indocyanine green (ICG) as the reference. Theoretical calculations revealed that the energy bandgaps (*E*_g_) between the highest occupied molecular orbital (HOMO) and the lowest unoccupied molecular orbital (LUMO) of DTPAP-2P and DTPAP-2B were 1.76 eV and 1.94 eV (Supplementary Fig. 27 and Supplementary Data 1), respectively, which is consistent with their absorption and PL spectra. To further elucidate the influence of different anions on the excited-state electronic structures, the electron density distributions were analyzed. Although the anionic components contribute negligibly to the molecular orbitals, their presence affects the degree of charge separation within the cationic moieties. Hole–electron analysis revealed that the charge-transfer (CT) ratios of DTPAP-2P and DTPAP-2B are 82.3% and 82.0%, respectively (Supplementary Fig. 28). These results suggest that DTPAP-2P NPs may exhibit stronger anion-π⁺ interactions than DTPAP-2B NPs, thereby enhancing the D-A interactions and facilitating the formation of an intramolecular charge transfer (ICT) state. Moreover, intermolecular π-π interactions may also be effectively prohibited by the anion-π^+^ interactions to boost the enhanced emission of DTPAP-2P NPs.

To further verify our design concept, the ROS generation ability of these two NPs was compared. Dichlorodihydrofluorescein (DCFH) was chosen as the ROS indicator. As shown in the Fig. 2d and Supplementary Fig. 29, in DMSO solution, none of DTPAP-2B, DTPAP-2P or Na(CCN)_5_ induced obvious changes of DCFH fluorescence intensity under the irradiation of 660 nm laser (0.1 W cm^-2^). Similarly, Na(CCN)_5_ NPs exhibited negligible ROS generation ability. On the contrary, the emission intensity of DCFH containing DTPAP-2B NPs or DTPAP-2P NPs increased rapidly upon 660 nm irradiation, exceeding that of the commercially available photosensitizer Chlorin e6 (Ce6), manifesting enhanced ROS generation ability of both DTPAP-2B and DTPAP-2P in NPs (Fig. 2e and Supplementary Fig. 30). In DMSO solution, both cations and anions can move freely, and the anion–π⁺ interactions between them are weak. When aggregation occurs in NPs, the anions are brought close to the cationic π⁺ systems, which greatly strengthens the anion–π⁺ interactions and promotes efficient ROS generation. It is worth noting that DTPAP-2P NPs exhibit stronger ROS generation ability compared to DTPAP-2B NPs, which may be attributed to the stronger anion-π^+^ interactions induced by organic planar π-conjugated (CCN)_5_^-^ and the higher light-harvesting capability of DTPAP-2P in NPs at 660 nm. This demonstrated the great potential of anion-π^+^ interaction as a general strategy to improve ROS generation. Next, singlet oxygen (^1^O_2_) indicator 9,10-anthracenediyl-bis(methylene)dimalonic acid (ABDA), superoxide anion indicator dihydrorhodamine 123 (DHR123) and hydroxyl radical indicator hydroxyphenyl fluorescein (HPF) were used respectively to further verify the type of ROS produced by NPs. As displayed in Fig. 2f‒h and Supplementary Figs. 31‒33, all indicators showed no obvious signal changes upon 660 nm laser irradiation alone. While in the presence of NPs, there was obvious decrease in the absorbance of ABDA, indicating that both NPs can produce ^1^O_2_. The ^1^O_2_ quantum yield were thus calculated to be 0.21 and 0.25 for DTPAP-2P NPs and DTPAP-2B NPs, respectively, using Ce6 as a reference. Additionally, upon light irradiation, the fluorescence intensity of DHR123 and HPF was greatly enhanced in the presence of DTPAP-2P NPs, indicating the efficient generation of type I ROS. The remarkable type I ROS generation ability of DTPAP-2P NPs was further confirmed by using white light irradiation as shown in Supplementary Figs. 34‒38. While in contrast, DTPAP-2B NPs induced less increase in the fluorescence intensity of DHR123 and HPF, demonstrating much lower type I ROS generation. Collectively, DTPAP-2B NPs mainly produced type II ROS, while DTPAP-2P NPs displayed much better type I radical ROS, which is favorable for overcoming the high oxygen dependence bottleneck of PDT against hypoxia tumors. Theoretical calculations revealed that the anion-π^+^ interactions enhanced the intramolecular donor-acceptor (D-A) interaction. This led to a smaller ΔE_ST_ in the excited state (Supplementary Fig. 39), which in turn facilitated the intersystem crossing (ISC) process in DTPAP-2P, ultimately resulting in the enhancment of ROS generation capability. Based on the above results, we can infer that organic planar π-conjugated anions participated anion-π^+^ interactions may provide a facile and useful strategy for constructing NIR-II PTAs with efficient type I ROS generation.

To validate the generality of this strategy, DMPHL-2P and DMPHL-2B were encapsulated with Pluronic F127 to form nanoparticles. DLS analysis showed average hydrodynamic diameters of 160 nm for DMPHL-2P NPs and 138 nm for DMPHL-2B NPs (Supplementary Fig. 40a). Both NPs exhibited broad absorption from 500 to 1000 nm, with maxima at 750 nm for DMPHL-2P NPs and 620 nm for DMPHL-2B NPs (Supplementary Fig. 40b). Compared with the DTPAP system, the DMPHL framework displayed a clear bathochromic shift, which was further enhanced upon exchange with the planar π-conjugated (CCN)_5_^-^ anion. Both NPs emitted in the NIR-II region with maxima around 1000 nm, while DMPHL-2P NPs showed slightly stronger fluorescence intensity (Supplementary Fig. 40c), indicating suppressed intermolecular π–π stacking due to reinforced anion–π^+^ interactions. ROS evaluation revealed that both NPs generated negligible ROS in the molecularly dissolved state (Supplementary Fig. 40 d). In contrast, type I ROS was predominantly produced by NPs, with a negligible contribution from type II ROS. Notably, DMPHL-2P NPs exhibited obviously enhanced type I ROS generation compared with DMPHL-2B NPs (Supplementary Fig. 40e, h and Supplementary Figs. 41‒45). These results confirm that simple substitution with a planar π-conjugated (CCN)_5_^-^ anion is sufficient to construct NIR-II photothermal agents with reinforced anion–π^+^ interactions and efficient type I ROS generation.

### Photothermal properties

**Fig. 3:**
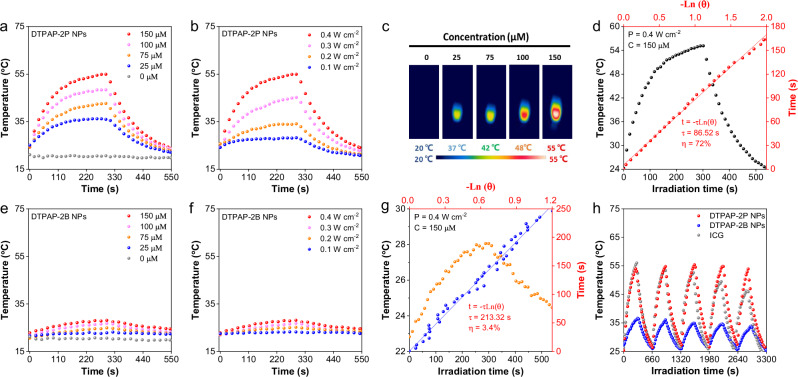
Photothermal properties of DTPAP-2P NPs and DTPAP-2B NPs. The concentration-dependent (**a**) and laser power-dependent (**b**) temperature elevation curves of DTPAP−2P NPs upon 660 nm laser irradiation. **c** Infrared thermal images of various concentrations of DTPAP-2P NPs under 660 nm laser irradiation (0.4 W cm^-2^). **d** Temperature increasing/decreasing curve and cooling time vs -ln(θ) plot (θ: temperature driving force) of DTPAP-2P NPs. The concentration-dependent (**e**) and laser power-dependent (**f**) temperature elevation curves of DTPAP-2B NPs upon 660 nm laser irradiation. **g** Temperature increasing/decreasing curve and cooling time vs -ln(θ) plot of DTPAP-2B NPs. **h** Photothermal stability of DTPAP-2P NPs, DTPAP-2B NPs and ICG during 5 cycles of heating-cooling. Source data are provided as a Source Data file.

In view of the multiple rotating phenyl groups in the TPA donor units, we also investigated the photothermal conversion performances of DTPAP-2P and DTPAP-2B. In DMSO solution, both DTPAP-2P and DTPAP-2B exhibited minor temperature increment as the 660 nm light irradiation time increased. For example, the temperature of DTPAP-2P in DMSO solution (150 μM) increased from 25 °C to 31 °C at a light power density of 0.4 W cm^-2^ (Supplementary Fig. 46). While after aggregates formation in NPs, DTPAP-2P NPs showed concentration and power density-dependent temperature increase as depicted in Fig. 3a, b. After 300 s of irradiation with a 660 nm laser at 0.4 W cm^-2^, the temperature of DTPAP-2P NPs in aqueous solution was increased to 55 °C (Fig. 3c). According to the literature method^68,69^, the PCE of DTPAP-2P and DTPAP-2B in DMSO solution and in NPs were calculated. As shown in Fig. 3d and Supplementary Fig. 46, DTPAP-2P NPs reached a high PCE value of 72%, which is largely enhanced compared to DTPAP-2P in pure DMSO solution (18%). Moreover, DTPAP-2P NPs also showed much higher PCE value than DTPAP-2B NPs (3.4%) under the same experimental condition (Fig. 3e‒g). These data manifested that DTPAP-2P NPs achieved a good balance between acceptor planarization and donor rotation. The cationic phenanthroline acceptor maintained good planarity to form strong anion-π^+^ interactions with the planar π-conjugated (CCN)_5_^-^ anion, which guaranteed the light-harvesting capability and the bright NIR-II emission. And the rotors in TPA donors promote the conversion of light to heat through the non-radiative pathways. Then, the photothermal performance of DTPAP-2B and DTPAP-2P in the solid powder state was further evaluated. Both samples exhibited pronounced concentration- and power density-dependent temperature elevations upon 660 nm laser irradiation. As shown in Supplementary Fig. 47, under 660 nm irradiation at 0.4 W cm^-2^ for 30 s, the temperature of DTPAP-2P powder rapidly increased to 96 °C, whereas DTPAP-2B powder reached 75 °C under identical conditions. In addition, the photothermal stability of DTPAP-2P NPs and DTPAP-2B NPs was evaluated with the FDA-approved clinical fluorescent dye ICG as a comparison. The samples were subjected to five heating–cooling cycles under the consecutive irradiation of a 660 nm laser (0.4 W cm^-2^). During repeated irradiation, the maximum temperature elevation of ICG gradually decreased from 56 °C to 47 °C, reflecting its intrinsic photothermal instability. In contrast, both DTPAP-2B NPs and DTPAP-2P NPs maintained nearly constant temperature profiles throughout the five on/off cycles, demonstrating their superior photothermal stability under repeated laser exposure (Fig. 3h). ICG was also used for comparing their photostability. After continuous irradiation of 660 nm laser (0.4 W cm^-2^) for 30 min, the fluorescence intensity of ICG was largely diminished. While DTPAP-2P NPs showed much better resistance to photobleaching (Supplementary Fig. 48), further assuring their potential performances in cancer phototheranostics.

The photothermal properties of DMPHL-2P NPs and DMPHL-2B NPs were also systematically investigated under 808 nm laser irradiation. As shown in Supplementary Fig. 49, pure aqueous solution exhibited slight temperature variation upon 808 nm irradiation, whereas the temperature elevation of DMPHL-2P NPs displayed a clear dependence on both the concentration and the power density of 808 nm laser. Upon exposure to an 808 nm laser at 0.3 W cm^-2^ for 5 min, the temperature of the DMPHL-2P NPs solution increased to 58 °C (Supplementary Fig. 49a‒c). The PCE of DMPHL-2P NPs was further determined to be 78.8% (Supplementary Fig. 49d), confirming their highly efficient photothermal conversion capability. Under identical conditions, the DMPHL-2B NPs solution induced only a temperature increase to 40 °C, with a remarkably lower PCE of 18.8% (Supplementary Fig. 49e‒h). Collectively, these results demonstrate the superior photothermal performance of DMPHL-2P NPs. The improved PCE as well as the redshifted absorption spectra, enhanced NIR-II emission and ROS generation demonstrated above underscores the effectiveness and generality of introducing planar π-conjugated organic anions as a straightforward strategy to construct high-performance anion–π^+^ type NIR-II photothermal agents.

### Crystal analysis and theoretical calculations

**Fig. 4:**
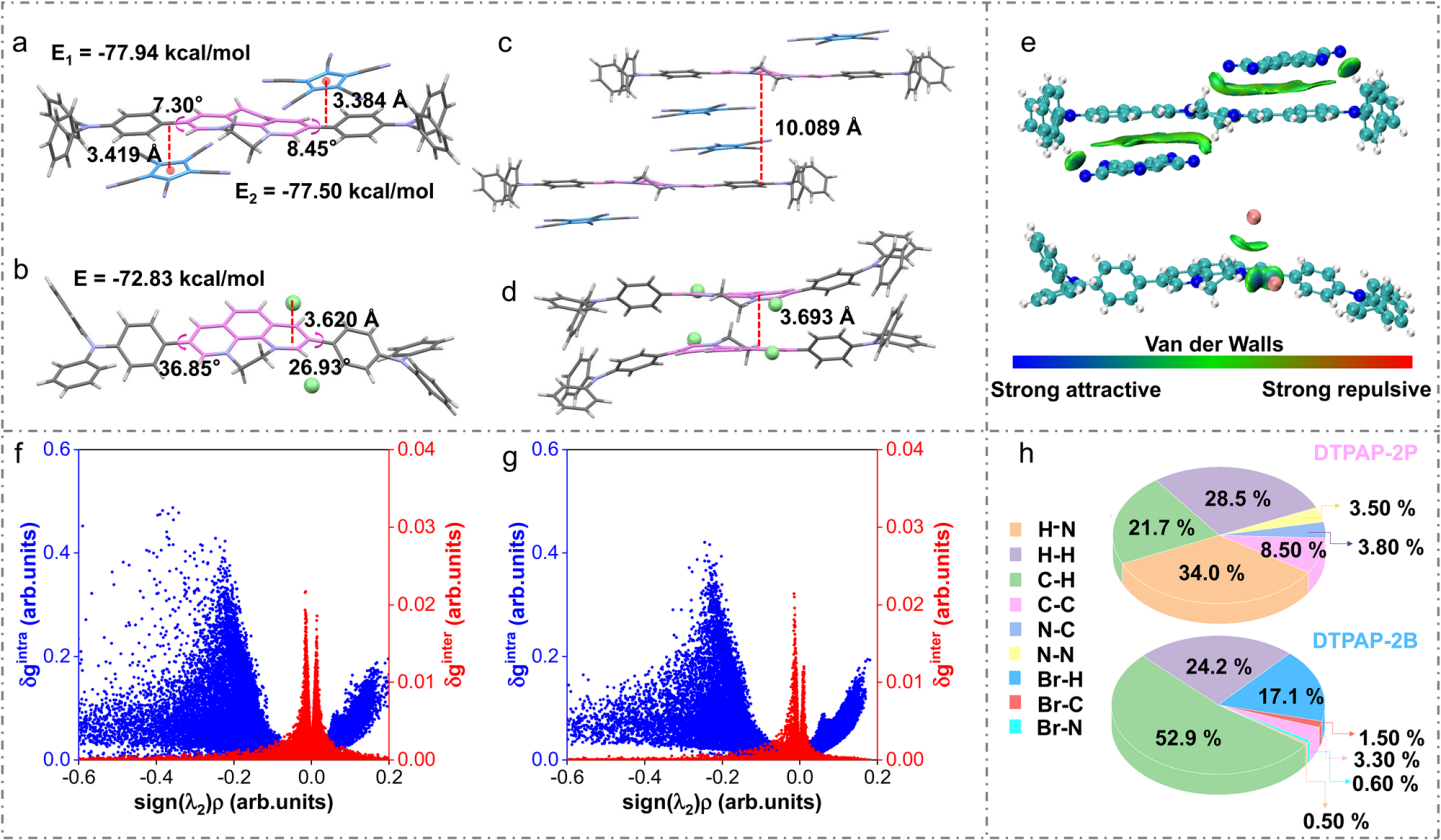
Single crystal X-ray diffraction analysis and theoretical calculations. Torsion angles and anion–π^+^ interaction distances of DTPAP-2P (**a**) and DTPAP-2B (**b**). E is defined as the interaction energy between π^+^ and anion calculated based on the single crystal structure by single-point calculations using the M062X/6-311 g method. Interlayer dimer structure diagrams of DTPAP-2P (**c**) and DTPAP-2B (**d**). **e** The visualized isosurfaces of the IGM analysis for DTPAP−2P (top) and DTPAP-2B (down) (δg^inter^ = 0.006). The 2D plot of δg^intra^ (blue) and δg^inter^ (red) for DTPAP-2P (**f**) and DTPAP-2B (**g**). **h** The proportions of the different noncovalent interactions in DTPAP-2P and DTPAP-2B based on Hirshfeld surface analysis. Source data are provided as a Source Data file.

In order to confirm the existence of anion-π^+^ interactions and to investigate the underlying mechanism that the organic planar π-conjugated anions participated anion-π^+^ interactions induced a simultaneous enhancement of NIR-II luminescence and photothermal properties, single crystals of DTPAP-2B (CCDC: 2387016) and DTPAP-2P (CCDC: 2466906) were obtained by slow evaporation (Supplementary Table 3 and Supplementary Fig. 50). Molecular configurations and stacking patterns in the crystals were investigated. Remarkably, as shown in Fig. 4a, b, the specific presence of (CCN)_5_^-^ on both sides of the positively charged phenanthroline ring with distances of 3.384 Å and 3.419 Å was observed, indicating dual anion-π^+^ interactions between (CCN)_5_^-^ and phenanthroline core in DTPAP-2P. The interaction energies between (CCN)_5_^-^ and phenanthroline core were calculated to be -77.94 and -77.50 kcal mol^−1^ respectively under the method of M062X/6-31 G. Notably, only one Br^-^ in the crystals of DTPAP-2B was found on one side of the phenanthroline core to form single anion-π^+^ interaction with a distance of 3.620 Å and the interaction energy of -72.83 kcal mol^−1^. According to previously reported research^47^, the anion-π^+^ interactions could efficiently block π–π stacking between luminophores, thereby inhibiting aggregation-caused quenching of luminescence. Meanwhile, the torsion angles between the phenanthroline core and the neighboring phenyl rings are relatively small (7.30° and 8.45°) for DTPAP-2P, demonstrating a distinctly planar structure. While for DTPAP-2B, the torsion angles were 36.85° and 26.93°, indicating more twisted structure of DTPAP-2B. The planar molecular conformation induced by planar π-conjugated anions participated anion-π^+^ interactions effectively enhances intramolecular D-A interactions, which results in redshifted absorption and emission in DTPAP-2P NPs. Since intermolecular packing modes also play a crucial role in the photophysical properties of the compounds, further analysis of the intermolecular interactions in DTPAP-2P and DTPAP-2B was conducted. For DTPAP-2P, the π-π distance between adjacent cationic phenanthroline was observed to be 10.089 Å (Fig. 4c), forming a unique sandwich structure. This effectively inhibits π-π stacking between luminophores, thus suppressing the fluorescence quenching induced by molecular aggregation. The stacking pattern endowed DTPAP-2P with a more stable and extended π-conjugated plane, favorable for obtaining redshifted absorption/emission and high PCE. In contrast, strong π-π stacking between adjacent phenanthroline cores with a distance of 3.693 Å was observed in DTPAP-2B (Fig. 4d). For DTPAP-2B, the single anion-π^+^ interaction between Br^-^ and phenanthroline core is not sufficient to prohibit the π-π stacking between luminophores, leading to the quenching of luminescence.

To further elucidate the underlying origins of the distinct luminescence between the two NPs, intramolecular and intermolecular weak interactions were evaluated using independent gradient model (IGM) analysis and the visual molecular dynamics (VMD) software based on the crystal structures^70–72^. DTPAP-2P exhibited much larger green regions between (CCN)_5_^-^ and phenanthroline core, demonstrating the formation of strong van der Waals interactions (Fig. 4e). Conversely, DTPAP-2B showed a much smaller green region between Br^-^ and phenanthroline core. Furthermore, the 2D plots of δg^intra^ and δg^inter^ (the terms of δg^intra^ and δg^inter^ were employed to distinguish between domains of intramolecular and intermolecular interactions) also illustrated more substantial intramolecular and intermolecular interactions in DTPAP-2P (Fig. 4f and g), consistent with the crystal analysis of DTPAP-2P. Altogether, the crystal of DTPAP-2P contained multiple and much stronger anion-π^+^ as well as intermolecular interactions, facilitating the suppression of nonradiative transitions and thereby benefiting the NIR-II luminescence.

Hirshfeld surface analysis was further used to quantify the proportion of intermolecular interactions in crystals (Fig. 4h and Supplementary Figs. 51, 52)^73^. For DTPAP-2P, the C–C interactions accounted for 8.5% of the overall interactions, which correlates with enhanced interactions between the (CCN)_5_^-^ anions and the phenanthroline core, as well as interactions among (CCN)_5_^-^ (Supplementary Fig. 53). These effects resulted in extended absorption/emission wavelengths and intensified NIR-II emission. In contrast, DTPAP-2B exhibits only 3.3% C-C interactions, primarily originating from phenanthroline core stacking, consequently leading to the severe quenching of its luminescent properties (Supplementary Fig. 54). Additionally, the N–H interactions between (CCN)_5_^-^ and phenanthroline core, accounting for 34.0% of the overall interactions, could restrict the motion of the phenanthroline ring, decrease nonradiative transition pathways, and further improve luminescent properties. However, DTPAP-2B exhibited a different interaction profile with a little proportion of 0.5% N–H interactions. These evidences confirm that the organic π planar anion substitution strategy can induce stronger anion-π^+^ interactions, effectively suppress π–π stacking between luminophores, thereby improving the NIR-II emission of DTPAP-2P NPs.

### Cell imaging and in vitro antitumor study

**Fig. 5:**
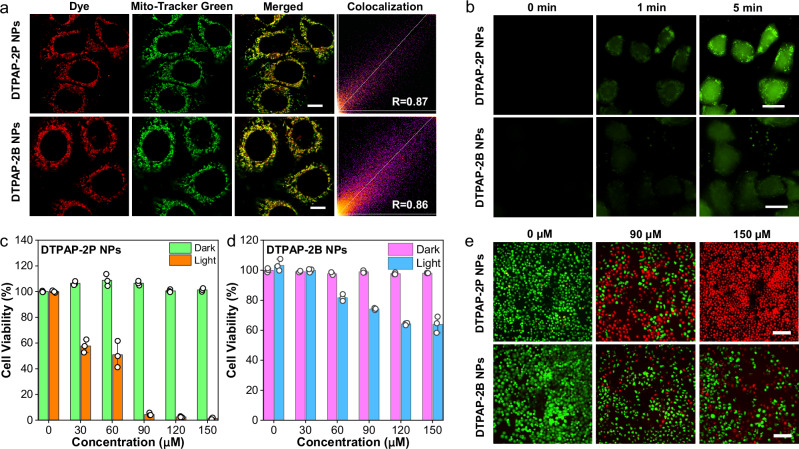
Cell imaging and in vitro antitumor study. **a** Colocalization images of DTPAP-2P NPs and DTPAP-2B NPs (10 μM) with Mito-Tracker Green (1 μM). Scale bar = 5 μm. The inset R value is the Pearson correlation coefficient. (*n* = 3, independent experimental replicates). **b** Imaging of ROS generation in HeLa cells with DTPAP-2P NPs and DTPAP-2B NPs upon white light irradiation for different times. Scale bar= 20 μm. (*n *= 3, independent experimental replicates). Viability of 4T1 cancer cells incubated with increasing concentrations of DTPAP-2P NPs (**c**) and DTPAP-2B NPs (**d**) without or with 660 nm laser irradiation (0.4 W cm^-2^) for 10 min (n = 3, independent experimental replicates). Data in (**c**) and (**d**) were presented as mean values ± SEM. **e** Live/dead cell staining assays of DTPAP-2P NPs and DTPAP-2B NPs under 660 nm laser (0.4 W cm^-2^) irradiation for 10 min. Scale bar= 100 μm. Source data are provided as a Source Data file.

Inspired by the enhanced NIR-II emission of DTPAP-2P NPs, they were applied to HeLa cells for real-time fluorescence imaging. First of all, HeLa cells were incubated with DTPAP-2P NPs or DTPAP-2B NPs for different duration (Supplementary Fig. 55a). Within the first 2 h, faint red fluorescence can be observed. As time goes by, the red fluorescent signals enhanced obviously, reaching a plateau at 8 h (Supplementary Fig. 56a). A similar trend was observed in 4T1 cells, where the intracellular uptake approached a plateau at ~8 h (Supplementary Figs. 55b and 56b). To further explore the subcellular localization ability of DTPAP-2P NPs and DTPAP-2B NPs, HeLa cells were further co-stained with commercially available mitochondria-targeting dye Mito-Tracker Green. After incubation for 8 h, bright red signals inside the cells were recorded and overlapped well with the signals of Mito-Tracker Green (Fig. 5a) with the Pearson correlation coefficients of 0.87 and 0.86, respectively. These results indicated DTPAP-2P NPs and DTPAP-2B NPs can be efficiently taken up by cancer cells and target mitochondria with high specificity. It is well acknowledged that mitochondria play a crucial role in regulating cell apoptosis. Hence, these NPs exhibited great promise for improved PDT performances. Thereafter, 2’,7’-dichlorodihydrofluorescein diacetate (DCFH-DA) was used as the indicator for assessing the intracellular ROS generation. Under white light irradiation, gradually enhanced green fluorescent signals were observed inside HeLa cells, which demonstrated that both NPs produced ROS inside cells (Fig. 5b). On the contrary, no ROS signal was observed in cells in the absence of light. Additionally, DTPAP-2P NPs induced much brighter green signals than DTPAP-2B NPs after 5 min irradiation, suggesting that DTPAP-2P NPs are more capable of generating ROS in cells, which is in line with their ROS generation in PBS. Then, dihydroethidium (DHE) and aminophenyl fluorescein (APF) were used as fluorescent probes for O_2_^•−^ and •OH to evaluate the intracellular production of type I ROS by DTPAP-2P NPs and DTPAP-2B NPs. As expected, the cells showed enhanced fluorescence signals within the cells in the presence of both NPs and laser light irradiation, suggesting that both NPs allow type I ROS generation inside cells. Importantly, after 5 min of irradiation, DTPAP-2P NPs induced brighter red and green signals compared to DTPAP-2B NPs under identical conditions (Supplementary Fig. 57), suggesting that DTPAP-2P NPs possess a superior capacity for intracellular type I ROS generation. This trend is fully consistent with the ROS generation profiles measured in PBS, further supporting the enhanced photodynamic activity of DTPAP-2P NPs. These data revealed that DTPAP-2P NPs can not only serve as photosensitizers for real-time and in situ imaging of cancer cells, but also show enormous potential for phototherapy in cancer treatment.

Considering the efficient ROS generation and high PCE of the two NPs, their cytotoxicity toward 4T1 cancer cells was evaluated in vitro. As shown in the Fig. 5c, d, both NPs displayed negligible toxicity toward 4T1 cells at a concentration of 150 μM in the dark, with the survival rate of 4T1 cells exceeded 90%. Even a higher concentration of 400 μM DTPAP-2P NPs exhibited minimal toxicity to 4T1 cells, with cell viability remaining above 80% (Supplementary Fig. 58). While after 10 min of irradiation with 660 nm laser, the survival rate of cancer cells incubated with DTPAP-2P NPs decreased to less than 10%. This indicated the superior cancer cell killing ability of DTPAP-2P NPs. However, under the same conditions, the killing rate of DTPAP-2B NPs is much weaker compared to DTPAP-2P NPs, with the cell survival rate exceeding 60%. Consistent results were likewise obtained in HeLa cells, further corroborating the photocytotoxicity of DTPAP-2P NPs (Supplementary Fig. 59). Moreover, live/dead cell co-staining experiments using Calcein-AM (green) and PI (red) further verified the superior cancer cell ablation capability of DTPAP-2P NPs. As shown in Fig. 5e, after incubated with 150 μM DTPAP-2P NPs, almost 100% of 4T1 cells showed bright red fluorescent signals under 660 nm laser (0.4 W cm^-2^) irradiation for 10 min, demonstrating robust cell death. In contrast, green emissions were observed in the dark, indicating the negligible cell death induced by DTPAP-2P NPs without the aid of laser light. Furthermore, a small portion of bright red signals was observed in 4T1 cells incubated with DTPAP-2B NPs. These data collectively confirmed that DTPAP-2P NPs provide excellent and much better synergistic PDT/PTT performance against cancer cells compared to DTPAP-2B NPs, which is in good accordance with their superior ROS generation and high PCE. To evaluate the synergistic effect of PDT/PTT, 4T1 cells were incubated with DTPAP-2P NPs in 4 °C ice-water bath for single PDT or pretreated with Vc inhibitor for single PTT. After incubated with 150 μM DTPAP-2P NPs and further irradiated by 660 nm laser light for 10 min, viability of 4T1 cells was 53.6 % for single PDT group and 9.85 % for single PTT group, respectively (Supplementary Fig. 60). However, cell viability was down to 1.30 % in the PDT/PTT synergistic phototherapy group (Fig. 5c). These results also verified that PTT played a major role in the synergistic phototherapeutic performance.

### NIR-II fluorescence imaging and photothermal imaging

**Fig. 6:**
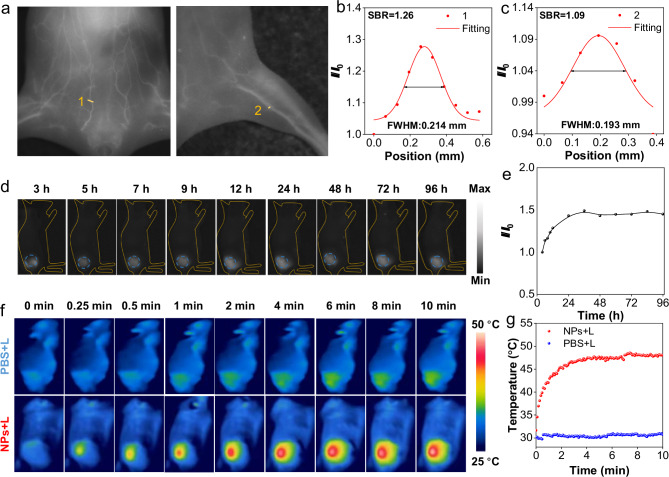
NIR-II fluorescence imaging and photothermal imaging. **a** The NIR-II fluorescence images of abdomen or hind-limb vessels in nude mice using 900 nm long-pass filters with 2000 ms laser activation. Corresponding signal-to-background ratio (SBR) analysis along the yellow line for position 1 (**b**) and position 2 (**c**) in (**a**). FWHM is defined as full width at half maximum. **d** NIR-II fluorescence imaging of the 4T1 tumor-bearing mice after DTPAP-2P NPs (600 μM, 50 μL) injection. A 660 nm laser was used as the excitation source. Images were recorded by a 900 nm LP filter after 2000 ms exposure. **e** Quantitative analysis of NIR-II emission in tumor region at 0-96 h after the injection of DTPAP-2P NPs. **f** In vivo infrared thermal images of PBS and DTPAP-2P NPs treated mice under 660 nm laser irradiation (0.4 W cm^-2^). **g** Corresponding temperature changes curves as a function of the 660 nm laser irradiation time. Source data are provided as a Source Data file.

Inspired by the stark NIR-II emission and the powerful performance in cancer cells, the feasibility of DTPAP-2P NPs in NIR-II imaging-guided synergistic PDT/PTT ablation of cancer cells was further evaluated in tumor-bearing mice. First, NIR-II vascular imaging in live mice has been studied. After intravenous injection (i.v. injection) of DTPAP-2P NPs, the blood vessels in the abdomen and hind limbs were clearly visible as shown in Fig. 6a. The NIR-II images of the abdomen capillary-level micro-vessel showed a narrow full width at half maximum (FWHM) of 214 μm and a high signal-to-background ratio (SBR) of 1.26. At the same time, the hind limb blood vessels with a diameter of 193 μm was clearly depicted with an SBR of 1.09 (Fig. 6b, c). The results of NIR-II angiography indicated that DTPAP-2P NPs can be further applied in imaging-guided precise therapy. Thereafter, 4T1 tumor-bearing mice was intratumorally injected with DTPAP-2P NPs. As shown in Fig. 6d, e, the NIR-II fluorescence signals at tumor site were rapidly increased and then maintained constantly for 96 h after the NPs administration, indicating DTPAP-2P NPs may be used for long-term tracking of the therapeutic process. After 24 h of DTPAP-2P NPs injection, the fluorescence signal of the tumor was very strong. Then, the major organs (heart, liver, spleen, lung, and kidney) and tumors of mice were dissected for ex vivo NIR-II imaging. As displayed in Supplementary Fig. 61, the bright NIR-II luminescence on tumor, in contrast to the negligible signal on the heart and lung, further confirmed the prolonged retention capability of DTPAP-2P NPs at the tumor site. The fluorescence quantitative assay demonstrated, DTPAP-2P NPs are mainly distributed in the liver, spleen, kidney and tumor, suggesting liver, spleen and kidney may be involved in the metabolism and clearance of DTPAP-2P NPs. Subsequently, the photothermal imaging of DTPAP-2P NPs in tumor site was also studied. As shown in Fig. 6f, the temperature at the tumor site gradually increased as 660 nm laser irradiation time prolonged. After 5 min of irradiation, the temperature at the tumor site reached a plateau of 50 °C. While a negligible temperature increase was observed in the control group with PBS instead of NPs injection (Fig. 6g). When the temperature reaches above 45 °C, the tumor cells could be effectively killed. Thus, DTPAP-2P NPs is of great promise for photothermal therapy against cancer cells. Moreover, the results here collectively demonstrated that DTPAP-2P NPs are ideal candidates for NIR-II imaging-guided phototherapy.

### In vivo anticancer study

**Fig. 7:**
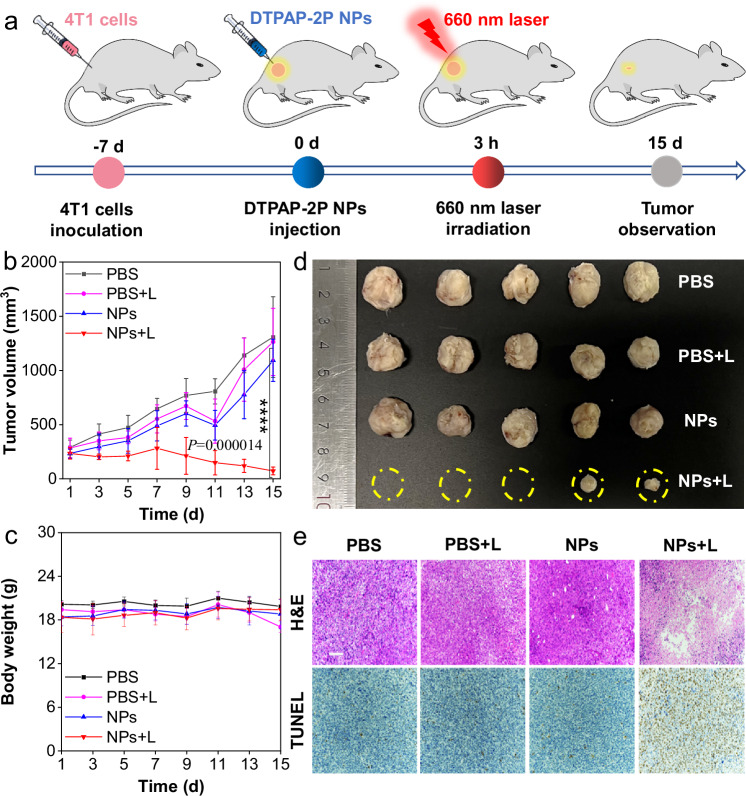
In vivo anticancer study. **a** Schematic illustration of the theranostic schedule using DTPAP-2P NPs. **b** Tumor volume, (**c**) body weight and (**d**) digital photographs of ex vivo tumor after 15 days treatment (*n* = 5, biologically independent mice). (**e**) H&E and TUNEL staining of tumors in mice after various treatments. Scale bar = 100 μm. (*n* = 3, biologically independent mice). Data in (**b**) and (**c**) were presented as mean values ± SEM. Statistical significance in (**b**) was determined by one-way ANOVA and multiple-comparison test (two-sided). Exact *P* values are indicated in the figure. ****P < 0.0001. Source data are provided as a Source Data file.

To verify the potential of DTPAP-2P NPs for NIR-II imaging-guided synergistic PDT/PTT ablation of tumors in vivo, twenty 4T1 tumor-bearing mice were randomly divided into four groups for different treatment: (a) injection of PBS without light irradiation (PBS group), (b) injection of PBS with 660 nm laser irradiation (PBS + L group), (c) injection of DTPAP-2P NPs without light irradiation (NPs group), and (d) injection of DTPAP-2P NPs with 660 nm laser irradiation (NPs+L group). After different treatments, the changes in tumor volume and mice body weight were monitored for 15 days (Fig. 7a). As shown in the Fig. 7b, the tumor growth of mice in NPs+L group was clearly suppressed. While in contrast, the tumor volumes of the other three groups were gradually increased with time. Fortunately, the mice body weight in all four groups remained constant with normal fluctuation (Fig. 7c), indicating that DTPAP-2P NPs have good biocompatibility and excellent phototherapeutic effects. Additionally, 15 days after the treatment, tumor tissues were dissected and directly observed. Images in Fig. 7d demonstrated that the tumor growth in NPs+L group was greatly suppressed or even ablated, solidly evidenced the powerful synergistic PDT/PTT effect of DTPAP-2P NPs. In addition, DTPAP-2P NPs are likely to induce immunogenic cell death (ICD) in tumor cells upon laser irradiation, thereby activating the host immune response and enhancing therapeutic efficacy. ICD is typically characterized by the release of various damage-associated molecular patterns (DAMPs), such as calreticulin (CRT), heat-shock proteins (HSPs), and high-mobility group box−1 (HMGB1)^74^. To validate this process, DAMPs expression levels in tumor tissues from different treatment groups were evaluated by immunofluorescence staining. As shown in Supplementary Fig. 62, the green fluorescence signals of CRT and HSP60 were enhanced in the DTPAP-2P NPs+L group, suggesting prominent CRT exposure and HSPs upregulation on tumor cell surfaces. Meanwhile, the level of HMGB1 in the DTPAP-2P NPs+L group was markedly decreased compared with the PBS and DTPAP-2P NPs+D groups, indicating massive extracellular release of HMGB1. Collectively, these results demonstrate that DTPAP-2P NPs plus light irradiation can effectively induce ICD and promote the release of multiple DAMPs, thereby showing great potential to activate the host immune system and enhance antitumor immune responses. In order to thoroughly evaluate the potential toxicity of NPs, the H&E (hematoxylin and eosin)-stained main organs slices and tumor sections were analyzed. The main organs (heart, liver, spleen, lungs, kidneys) of mice in each group showed normal tissue morphology without any obvious changes (Supplementary Fig. 63). Meanwhile, the results of H&E and terminal deoxynucleotidyl transferase-mediated nick end labeling (TUNEL) staining assay indicated that nucleus shrink and apoptosis of tumor cells were clearly observed in NPs+L group compared to other groups (Fig. 7e). The above results suggest that DTPAP-2P NPs not only exhibited excellent phototherapeutic performance against tumors, but also showed negligible systemic toxicity. Furthermore, the hemolysis analysis suggested that DTPAP-2P NPs had excellent blood compatibility even at a high concentration of 150 µM, providing additional evidence of their biosafety (Supplementary Fig. 64). The routine blood indices and blood biochemical indices of all mice were within the normal range (Supplementary Figs. 65, 66), which further confirmed that the systemic toxicity of DTPAP-2P NPs in mice was negligible. Overall, the advantages of high safety and good phototherapeutic effects of DTPAP-2P NPs makes them ideal choices for application in NIR-II imaging-guided cancer theranostics.

## Discussion

The development of robust NIR-II PTAs is of high significance for advanced deep-tissue phototheranostics. Although anion–π⁺-type photosensitizers have shown great promise, current studies have largely focused on modifying the π⁺-system or introducing simple inorganic counterions, leaving the development of anion-π^+^ type NIR-II PTAs largely unexplored. In this work, we propose a simple yet effective strategy to construct an anion-π^+^ type NIR-II PTA (DTPAP-2P) by replacing the inorganic Br^-^ in DTPAP-2B with organic planar π-conjugated (CCN)_5_^-^. Stark contrast in properties between DTPAP-2B and DTPAP-2P underscores the critical role of the organic planar π-conjugated anion. In solution, both DTPAP-2B and DTPAP-2P exhibited similar and poor optical properties. In contrast, upon formation of NPs, DTPAP-2P NPs demonstrated redshifted absorption, bright NIR-II emission, and dramatically enhanced ROS generation and PCE, illustrating the advantages of the strong, dual anion-π^+^ interactions induced by the introduction of organic planar π-conjugated (CCN)_5_^-^. Single crystal structure analysis confirms that DTPAP-2P exhibits much stronger dual anion–π⁺ interactions compared to the weak single anion–π⁺ interaction in DTPAP-2B. Further crystallographic analysis and theoretical calculations reveal that these dual anion–π⁺ interactions enhance intramolecular D–A interactions while suppressing detrimental intermolecular π–π stacking between luminogens. Consequently, DTPAP-2P NPs exhibit redshifts in maximum absorption and emission wavelengths, as well as a marked increase in the molar extinction coefficient at 660 nm, relative to DTPAP-2B NPs. The successful extension of this strategy to other π-conjugated cations (DMPHL-2B and DMPHL-2P) further demonstrates the generality of our approach.

The biological evaluation validates the power of our molecular design. Efficient mitochondrial targeting, high-resolution NIR-II angiography capable of resolving capillary-level microvessels, and successful NIR-II imaging-guided synergistic therapy in a murine tumor model collectively demonstrate the great potential of DTPAP-2P NPs for cancer theranostics. The absence of systemic toxicity in major organs and blood panels further confirms the excellent biocompatibility of this nanoplatform. Overall, the strategy of introducing an organic planar π-conjugated counterion provides a facile route to anion–π⁺-type NIR-II PTAs.

## Methods

### Materials

Amphiphilic copolymer Pluronic F127 (F127) was purchased from Avanti Polar Lipids. Phosphate buffered solution (PBS, pH 7.4), 2’,7’-dichlorodihydrofluorescein diacetate (DCFH-DA), 9,10-anthracenediyl-bis(methylene)dimalonic acid (ABDA), hydroxyphenyl fluorescein (HPF), dihydrorhodamine 123 (DHR123) and calcein acetoxymethylester (Calcein-AM) were purchased from Sigma-Aldrich. Thiazolyl blue tetrazolium bromide (MTT) were purchased from Beyotime biotechnology Co., Ltd. Dulbecco’s modified eagle’s medium (DMEM), fetal bovine serum (FBS), penicillin and streptomycin were purchased from Gibco. All other solvents and reagents used in this study were purchased from Admas-beta^®^ and used directly without further purification.

### Instruments

^1^H, ^13^C NMR and 2D NMR spectroscopy spectra were recorded with Bruker ARX 500 NMR or Bruker ARX 600 NMR spectrometer using tetramethylsilane (TMS) as a reference at room temperature. High-resolution mass spectra were collected by a LCT premier XE (Waters) HRMS spectrometry or XEVO G2-XS QTOF (Waters) HRMS spectrometry. X-ray photoelectron spectroscopy (XPS) was measured by a Thermo ESCALAB 250Xi spectrometer. The ion chromatography (Combustion IC) was measured by a 930 Combustion IC PP (AJ) system. Absorption spectra were measured on a SHIMADZU UV-2600i spectrophotometer. Photoluminescence (PL) spectra were recorded with an FS5 spectrofluorometer using 660 nm laser as excitation light source (CNI laser, MDL-XD-660). The absolute fluorescence quantum yield was measured using a Hamamatsu quantum yield spectrometer C11347−11 Quantaurus QY. The size distribution was analyzed by dynamic light scattering (DLS) using an Omni NanoBrook. Particle size and morphology were observed on a Hitachi HT 7800 transmission electron microscope (TEM). Temperature change was monitored by an FLIR E8-XT camera (FLIR System). Photodynamic and photothermal experiments were conducted using a 660 nm laser (CNI laser, MDL-XD-660) as the excitation light source. The cell viability was assessed by MTT assay, and the absorbance of each sample was measured at 490 nm using a microplate reader (BioTek). The in vivo fluorescence images were acquired on the commercial NIR-II Small Animal Imaging System Series III 900/1700S (Suzhou NIR-Optics Technology Co., Ltd., China) equipped with 900 nm long-pass (LP) filters. The blood biochemistry parameters were collected by the fully automatic biochemical analysis (Chemray 240). Cellular imaging experiments were performed with confocal laser scanning microscope (LSM880, ZEISS, Germany) or inverted fluorescence microscope (Nikon Ti2).

### Synthesis

DTPAP and sodium pentacyanocyclopentadienide (Na(CCN)_5_) were easily synthesized according to the previous reported literature^65,66,75^.

Synthesis of DTPAP-2B: To a 100 mL two-neck flask was added DTPAP (667.5 mg, 0.5 mmol), 1,2-dibromoethane (187.9 mg, 0.5 mmol) and freshly distilled THF (10 mL). The reaction mixture was refluxed for 24 h. Then, the solution was filtered and washed with 80-100 mL of CH_2_Cl_2_. The purplish-black solid precipitate was extracted to obtain the target product DTPAP-2B in 90% yield. ^1^H NMR (600 MHz, DMSO-*d*_6_) δ 10.25 (s, 2H), 9.80 (s, 2H), 8.64 (s, 2H), 8.08 (d, *J* = 8.5 Hz, 4H), 7.44 (t, *J* = 7.7 Hz, 8H), 7.22 (t, *J* = 7.4 Hz, 4H), 7.20 (d, *J* = 7.9 Hz, 8H), 7.16 (d, *J* = 8.4 Hz, 4H), 5.67 (s, 4H). ^13^C NMR (151 MHz, DMSO-*d*_6_) δ 150.43, 147.47, 146.59, 140.78, 138.09, 130.99, 130.44, 129.85, 129.49, 127.34, 126.02, 125.27, 125.21, 121.64, 52.59. HRMS [M]^2+^ calcd 347.1548, found 347.1557.

Synthesis of DTPAP-2P: DTPAP-2B (85.2 mg, 0.1 mmol) were dissolved in a mixed solvent of methanol and CH_2_Cl_2_. Then excess amount of Na(CCN)_5_ (213.0 mg, 1 mmol) was added and stirred for 3 h at room temperature for counterion exchange. After the stirring was completed, the solution was concentrated. Then the ultrapure water was added, followed by repeated extraction with CH_2_Cl_2_. Finally, the obtained organic phase was concentrated and crystallized to give DTPAP-2P in 99% yield. ^1^H NMR (500 MHz, DMSO-*d*_6_) δ 10.14 (s, 2H), 9.73 (s, 2H), 8.59 (s, 2H), 8.00 (d, *J* = 7.5 Hz, 4H), 7.41 (t, *J* = 7.2 Hz, 8H), 7.21 – 7.13 (m, 16H), 5.57 (s, 4H). ^13^C NMR (151 MHz, DMSO-*d*_6_) δ 150.48, 147.47, 146.60, 140.81, 138.17, 131.04, 130.41, 129.87, 129.45, 127.32, 126.02, 125.25, 121.64, 113.47, 102.22, 52.62. HRMS [M]^2+^ calcd 347.1548, found 347.1555.

Synthesis of **1**: To a 100 mL round-bottom flask were added 2-ethylpyridine (10.0 g, 93.4 mmol), bromoacetophenone (18.6 g, 93.4 mmol), and acetone (50 mL). The reaction mixture was stirred at reflux under air for 21 h. After completion, the mixture was cooled to room temperature, and the resulting white precipitate was collected by filtration and washed with acetone. The obtained white solid was then transferred to a 250 mL flask, followed by the addition of NaHCO_3_ (31.0 g, 370.0 mmol). The mixture was stirred at reflux under air for 1.5 h. A biphasic system gradually formed during the reaction. Upon cooling to room temperature, the upper layer crystallized to give a dark solid. The crystals were collected by filtration, dissolved in CH_3_CN, passed through a thin pad of silica gel, and concentrated under reduced pressure to afford an off-white solid (17.6 g, 85.1 mmol, 92% yield). ^1^H NMR (600 MHz, Chloroform-*d*) δ 7.88 (d, *J* = 7.0 Hz, 1H), 7.60 (d, *J* = 6.8 Hz, 2H), 7.49 (t, *J* = 7.6 Hz, 2H), 7.43 (s, 1H), 7.39 - 7.35 (m, 2H), 6.66 (dd, *J* = 9.1, 6.4 Hz, 1H), 6.46 (t, *J* = 6.1 Hz, 1H), 2.50 (s, 3H).

Synthesis of **2**: In a 100 mL double-necked round-bottom flask, 2,3-diaminonaphthalene (l89.8 mg, 1.2 mmol) and 3,8-dibromo-1,10-phenanthroline-5,6-dione (368.0 mg, 1.0 mmol) were dissolved in acetic acid (20 mL). The reaction mixture was thoroughly degassed and purged with nitrogen, and then heated at 110 °C for 12 h under a nitrogen atmosphere. After completion of the reaction, the mixture was allowed to cool to room temperature, and the resulting precipitate was collected and washed with ethanol to afford compound 2 as a brownish-yellow solid (439.1 mg, 90% yield).

Synthesis of DMPHL: To a flame dried pressure flask under N_2_ was added compound **1** (1.3 g, 6.3 mmol), compound **2** (1.3 g, 2.7 mmol), Pd(OAc)_2_ (61.4 mg, 0.3 mmol), PCy_3_HBF_4_ (201.0 mg, 0.6 mmol), Cs_2_CO_3_ (5.3 g, 16.4 mmol), and anhydrous toluene (11 mL). The reaction was degassed with N_2_ for 15 min before sealing and heating to 120 °C for 16 h. The reaction was cooled to room temperature and transferred to a separatory funnel containing water and CH_2_Cl_2_, and the organic layer was collected. The aqueous layer was washed three more times with CH_2_Cl_2_. The organic layers were combined, dried over Na_2_SO_4_, and concentrated to dryness to yield the crude product. Purification by silica gel column chromatography (CH_2_Cl_2_/PE = 20:1, *v*/*v*) yielded the product as a matte red solid (0.7 g, 1.0 mmol, 35%). ^1^H NMR (400 MHz, Chloroform-*d*) δ 9.70 (d, *J* = 2.3 Hz, 2H), 8.97 (br, 2H), 8.84 (s, 2H), 8.34 (d, *J* = 7.1 Hz, 2H), 8.15 (dd, *J* = 6.6, 3.2 Hz, 2H), 7.59 (dd, *J* = 6.6, 3.2 Hz, 2H), 7.49 (dt, *J* = 9.1, 1.2 Hz, 2H), 7.32 – 7.29 (m, 8H), 7.25 – 7.20 (m, 2H), 6.80 (dd, *J* = 9.1, 6.3 Hz, 2H), 6.59 (td, *J* = 6.8, 1.3 Hz, 2H), 2.40 (s, 6H).

Synthesis of DMPHL-2B: To a 100 mL two-neck round-bottom flask were added DMPHL (742.3 mg, 0.5 mmol), 1,2-dibromoethane (1.9 g, 5 mmol), and freshly distilled tetrahydrofuran (THF, 10 mL). The reaction mixture was heated to reflux and stirred for 24 h. After cooling to room temperature, the mixture was transferred to a separatory funnel containing water and dichloromethane (CH_2_Cl_2_). The organic layer was collected, and the aqueous phase was further extracted with CH_2_Cl_2_ (×3). The combined organic extracts were dried over anhydrous Na_2_SO_4_, filtered, and concentrated under reduced pressure to afford the crude product. Purification by silica gel column chromatography (CH_2_Cl_2_/MeOH = 50:1, *v*/*v*) yielded the product as a matte green solid (92.8 mg, 0.1 mmol, 20% yield). ^1^H NMR (600 MHz, Chloroform-*d*) δ 9.58 (s, 2H), 9.21 (d, *J* = 6.9 Hz, 2H), 9.18 (s, 2H), 8.80 (s, 2H), 8.28 (br, 2H), 7.74 (br, 2H), 7.55 (d, *J* = 9.0 Hz, 2H), 7.51 – 7.48 (m, 4H), 7.45 (d, *J* = 7.6 Hz, 4H), 7.37 (d, *J* = 7.4 Hz, 2H), 7.11 (t, *J* = 7.7 Hz, 2H), 6.94 (t, *J* = 6.6 Hz, 2H), 5.67 (s, 4H), 2.35 (s, 6H). ^13^C NMR (151 MHz, CDCl_3_) δ 146.09, 139.13, 138.37, 137.53, 136.00, 135.89, 133.89, 133.75, 131.87, 131.14, 130.68, 129.86, 129.51, 128.97, 128.73, 128.59, 128.08, 127.96, 123.05, 122.53, 118.26, 115.58, 114.39, 112.55, 52.77, 29.79. HRMS [M]^2+^ calcd 385.1576, found 385.1580.

Synthesis of DMPHL-2P: DMPHL-2B (74.2 mg, 0.1 mmol) were dissolved in a mixed solvent of methanol and CH_2_Cl_2_. Then excess amount of Na(CCN)_5_ (213.0 mg, 1.0 mmol) was added and stirred for 3 h at room temperature for counterion exchange. After the stirring was completed, the solution was concentrated. Then the ultrapure water was added, followed by repeated extraction with CH_2_Cl_2_. Finally, the obtained organic phase was concentrated and crystallized to give the matte green solid. HRMS [M]^2+^ calcd 385.1576, found 385.1572.

### Halogen analysis by combustion IC

The halogen contents in the samples were determined using a 930 Combustion IC PP (AJ) system (Metrohm, Switzerland) by the combustion ion chromatography (Combustion IC) method. In this method, the samples were decomposed by high-temperature combustion, and the resulting halide gases were absorbed into a trapping solution for subsequent ion chromatographic analysis. For anion analysis, a Metrohm A7-250 column was used with 0.36 mmol/L Na_2_CO_3_ as the eluent at a flow rate of 0.7 mL/min. A suppressor was employed to reduce background conductivity and enhance detection sensitivity. Sample injection was performed via a preconcentration column to improve the accuracy of trace ion detection.

### Density functional theory calculations

The molecular geometries were optimized by Gaussian 09 on the calculation level of B3LYP/6-311 G(d) under normal convergence criteria with consideration of water solvent environment^76^. On the basis of the optimized geometries, time-dependent density functional theory (TD-DFT) was utilized at the same level to calculate the first singlet state (S_1_) and the first triplet state (T_1_). The Hole–electron distribution were calculated based on optimized S_1_ structures using Multiwfn. The Hirshfeld surfaces and decomposed fingerprint plots were mapped using Crystal Explorer 17.5 package^77–79^. The independent gradient model (IGM) analysis^72,80^ of weak interaction based on single crystal structure was conducted by using Multiwfn program^70^. The corresponding structure and IGM isosurfaces were generated using visual molecular dynamics (VMD) program^71^.

### Preparation of nanoparticles

All nanoparticles were prepared by a well-established nanoprecipitation method. Compounds DTPAP-2B, DTPAP-2P or DMPHL-2P, DMPHL-2B (1 mg) and F127 (5 mg) were dissolved respectively into 1 mL of dimethyl sulfoxide (DMSO) and mixed homogeneously. The mixed solution was injected once into 10 mL of double distilled water under ultrasonic conditions for 5 min, then transferred to a dialysis bag (molecular weight cutoff = 3600) and dialyzed against deionized water for 24 h. The as-prepared NPs were concentrated before use. The concentration of these nanoparticles was estimated by UV–Vis absorption spectroscopy using a calibration curve established from DMSO solutions of the corresponding compounds as the reference.

### Determination of molar extinction coefficient of the molecular compound in the NP suspension

Molar concentration of the molecular compound in the NP suspension was determined using the calibration curve established from the corresponding molecular compound in DMSO. Briefly, a series of standard solutions of the molecular compound in DMSO were prepared, and a calibration curve was constructed by plotting absorbance versus concentration. The absorption spectrum of the NPs dispersed in DMSO was then recorded, and molar concentration of the molecular compound in the NP suspension was determined according to the standard calibration curve. Subsequently, the UV–vis absorption spectrum of DTPAP-2B or DTPAP-2P in the nanoparticle suspension (2 mL PBS, working concentration: 50 μM) was recorded at room temperature using a UV–vis spectrophotometer equipped with a 1 cm quartz cuvette. The absorbance at 660 nm was extracted from the recorded spectrum. The ε at 660 nm was calculated according to the Beer–Lambert law, based on Eq. (1):

1 $$\varepsilon=\frac{A}{c\times l}$$

Where A is the absorbance at 660 nm, c is the molar concentration of the NPs, and l is the optical path length (1 cm).

### Determination of relative fluorescence quantum yield of DTPAP-2P NPs

The relative QY of DTPAP-2P NPs was determined by comparing them to ICG, which exhibited a total QY of 2.7%^81,82^. The fluorescence emission spectrum of the ICG dye in water was recorded at varying absorbance levels (0.10, 0.08, 0.06, 0.04, and 0.02) at 660 nm. Subsequently, the absorption and emission spectra of the NPs in water were analyzed using an Edinburgh FS5 fluorescence spectrophotometer. The integrated PL intensity within the wavelength range of 900-1300 nm was plotted against the absorbance at 660 nm. A linear function was fitted to the data and utilized in the determination of the QY for DTPAP-2P NPs, based on Eq. (2):

2 $${{QY}}_{{sample}}={{QY}}_{{ref}}\times \frac{{{slope}}_{{sample}}}{{{slope}}_{{ref}}}\times {\left(\frac{{n}_{{sample}}}{{n}_{{ref}}}\right)}^{2}$$

Where slope_sample_ and slope_ref_ represent the slopes of DTPAP-2P NPs and ICG dye in water, respectively, which are obtained after linear fitting curves within certain ranges. Both *n*_sample_ and *n*_ref_ denote the refractive index of water.

### Storage stability

The concentrated aqueous solution of as-prepared NPs was diluted with PBS and stored at 4 °C in a fridge. At designated time, aliquot of the solution was taken and the stability was monitored by DLS during 7 day’s storage.

### Total ROS detection by DCFH

Dichlorodihydrofluorescein (DCFH) was applied to detect ROS. Typically, stock solutions of DCFH (40 μM) in PBS, Ce6 (1 mM) in DMSO and NPs (1 mM) in aqueous solution were prepared respectively. In a general procedure of ROS detection in PBS solution, 50 μL of DCFH stock solution and 20 μL of Ce6 or NPs stock solution were added to 2 mL PBS buffer (pH 7.4). Therefore, the final concentration of DCFH in the assay system was 1 μM. And the final concentration of Ce6 or NPs in the assay system was 10 μM. Control groups were prepared by adding 50 μL DCFH stock solution into 2 mL PBS. After that, the mixtures were exposed to 660 nm irradiation (0.1 W cm^-2^, spot size 7.5 × 7.5 cm) or white light irradiation (10 mW cm^-2^), and the PL intensities at ~530 nm were recorded for every five seconds in a PL spectrofluorometer (Excitation wavelength: 489 nm). The detection of ROS generation in DMSO solutions was carried out in similar procedure. And for DMPHL-2P NPs and DMPHL-2B NPs, an 808 nm laser (spot size 6.5 × 4.5 cm) was used instead of the 660 nm laser.

### Detection of ^1^O_2_ generation by ABDA

^1^O_2_ generation in solution was monitored with ABDA as the indicator, and Ce6 was employed as the standard photosensitizer. Briefly, stock solutions of ABDA (10 mM) in DMSO, Ce6 (1 mM) in DMSO and NPs (1 mM) in aqueous solution were prepared respectively. Then, 10 μL ABDA stock solution and 20 μL stock solutions of DTPAP-2B NPs, DTPAP-2P NPs or Ce6 were added into 2 mL PBS. Thus, the final concentration of ABDA was ~50 μM, and the final concentration of Ce6 or NPs in the assay system was ~10 μM. The mixture was irradiated by 660 nm laser (0.1 W cm^-2^, spot size 7.5 × 7.5 cm) or white light irradiation (10 mW cm^-2^). Then, the absorbance of ABDA at 325-425 nm was recorded for every five seconds. And for DMPHL-2P NPs and DMPHL-2B NPs, an 808 nm laser was used instead of the 660 nm laser. ROS quantum yield of NPs was calculated by the Eq. (3):

3 $${{{\varnothing }}}_{{NPs}}={{{\varnothing }}}_{{Ce}6}\frac{{K}_{{NPs}}}{{K}_{{Ce}6}}/\frac{{A}_{{NPs}}}{{A}_{{Ce}6}}$$

where *K*_NPs_ and *K*_Ce6_ are the decomposition rate constants of ABDA with NPs and Ce6, respectively. *A*_NPs_ and *A*_Ce6_ represent the light absorbed by NPs and Ce6, respectively, which are determined by integration of the areas under the absorption bands in the wavelength range of 400–800 nm. *Φ*_Ce6_ is the ^1^O_2_ quantum yield of Ce6, which is 0.65 in water^83,84^.

### Detection of O_2_^•−^ generation by DHR123

Compound DHR123 was used as indicator for detection of O_2_^•−^ in solution. When O_2_^•−^ is generated in the system, DHR123 will be oxidized to emit strong fluorescence at 530 nm. Briefly, stock solutions of DHR123 (5 mM) in DMSO, Ce6 (1 mM) in DMSO and NPs (1 mM) in aqueous solution were prepared respectively. Then, 2 μL DHR123 stock solution and 20 μL stock solutions of DTPAP-2B NPs, DTPAP-2P NPs or Ce6 were added into 2 mL PBS. Thus, the final concentration of DHR123 was ~5 μM, and the final concentration of Ce6 or NPs in the assay system was ~10 μM. The mixture was then placed in a cuvette and irradiated by 660 nm laser (0.1 W cm^-2^, spot size 7.5 × 7.5 cm) or white light irradiation (10 mW cm^-2^). And for DMPHL-2P NPs and DMPHL-2B NPs, an 808 nm laser was used instead of the 660 nm laser. The fluorescence changes of sample at 530 nm were recorded by the PL spectrofluorometer (Excitation wavelength: 480 nm).

### Detection of •OH generation by HPF

Compound HPF was used as indicator for detection of •OH in solution. When •OH is generated in the system, HPF will be oxidized to emit strong fluorescence at 515 nm. Briefly, stock solutions of HPF (5 mM) in DMSO, Ce6 (1 mM) in DMSO and NPs (1 mM) in aqueous solution were prepared respectively. Then, 2 μL HPF stock solution and 20 μL stock solutions of DTPAP-2B NPs, DTPAP-2P NPs or Ce6 were added into 2 mL PBS. Thus, the final concentration of HPF was ~5 μM, and the final concentration of Ce6 or NPs in the assay system was ~10 μM. The mixture was then placed in a cuvette and irradiated by 660 nm laser (0.1 W cm^-2^, spot size 7.5 × 7.5 cm) or white light irradiation (10 mW cm^-2^). And for DMPHL-2P NPs and DMPHL-2B NPs, an 808 nm laser was used instead of the 660 nm laser. The fluorescence changes of sample at 530 nm were recorded by the PL spectrofluorometer (Excitation wavelength: 490 nm).

### Photothermal properties

To determine the photothermal properties of DTPAP-2B NPs or DTPAP-2P NPs, the solution of NPs (150 μM) was irradiated by 660 nm laser at different power densities (0.1 W cm^-2^, 0.2 W cm^-2^, 0.3 W cm^-2^ and 0.4 W cm^-2^). The temperature changes were monitored by FLIR E8-XT camera. Different concentrations of DTPAP-2B NPs or DTPAP-2P NPs were prepared (0 μM, 25 μM, 75 μM, 100 μM and 150 μM), and irradiated by 0.4 W cm^-2^ with 660 nm laser for 5 min, and then the laser was turned off for 4 min. The temperature changes were monitored during irradiation. The photothermal conversion efficiency of NPs was determined according to previously reported publications^74^. The solution of DTPAP-2B NPs or DTPAP−2P NPs (150 μM) were exposed to 660 nm laser irradiation at 0.4 W cm^-2^ for 5 min when the temperature reached a plateau. At this time point, the laser was shut off. Then the solution was cooled down to room temperature. The temperature of the solution was recorded at an interval of 15 s during this process. The photothermal conversion efficiency was determined according to Equation (d), and the other parameters in Eq. (4) were calculated from equation (5), (6) and (7).

4 $$\eta=\frac{{hS}\left({{T}_{{Surr}}T}_{{Max}}-\right)-{Q}_{{Dis}}}{I\left(1-{10}^{{-A}_{660}}\right)}$$

5 $${\tau }_{s}=\frac{{\sum }_{i}{m}_{i}{C}_{p,i}}{hS}$$

6 $$t={\tau }_{s}\left(-\mathrm{ln}\theta \right)$$

7 $$\theta=\frac{T-{T}_{{Surr}}}{{T}_{{Max}}-{T}_{{Surr}}}$$

In Eq. (4), *η* is the photothermal conversion efficiency, *h* is the heat transfer coefficient; *S* is the surface area of the container. *Q*_*dis*_ represents heat dissipated from the laser mediated by the solvent and container. *I* is the laser power and *A*_*660*_ is the absorbance of the sample at 660 nm. In Eq. (5), m is the mass of the solution containing the photoactive material, *C* is the specific heat capacity of the solution (*C*_*water*_ = 4.2 J g^-1^). In Eq. (6), *τ*_*s*_ is the associated time constant. In Eq. (7), *θ* is a dimensionless parameter, known as the driving force temperature. *T*_*max*_ and *T*_*Surr*_ are the maximum steady state temperature and the environmental temperature, respectively.

For determining the photothermal properties of DMPHL-2P NPs and DMPHL-2B NPs, a similar procedure was adopted expect that an 808 nm laser was used instead of the 660 nm laser.

### Cell culture

HeLa and 4T1 cells were respectively cultured in DMEM or Roswell Park Memorial Institute (RPMI) 1640 medium (containing 10% heat-inactivated FBS, 1% 100 mg∙mL^-1^ penicillin and 100 mg∙mL^-1^ streptomycin) at 37 °C in a humidified incubator with 5% CO_2_. Before the experiments, the cells were precultured until confluence was reached.

### Cellular uptake assay

20 μM of DTPAP-2P NPs and DTPAP-2B NPs were added to 5 × 10^6^ HeLa cells or 4T1 cells and incubated at 37 °C for 2, 4, 6, 8, 10, 12 h. Then cells were imaged using confocal laser scanning microscopy (CLSM). The excitation wavelength of DTPAP-2P NPs and DTPAP-2B NPs was 543 nm and the emission collection wavelength was LP570 nm.

### Subcellular organelle colocalization

HeLa cells were seeded in confocal microscopy dishes and incubated for at least 24 h. Then DTPAP-2B NPs or DTPAP-2P NPs (10 μM) were added for 8 h incubation, respectively. The cells were washed with PBS three times and Mito-Tracker Green (1 μM) were added for another 30 min incubation after NPs treatment. Then, the cells were observed with a confocal microscope (Zeiss) in sequential scanning mode. DTPAP-2B NPs or DTPAP-2P NPs: λ_ex_ = 543 nm, λ_em_=Longpass 570 nm. Mito-Tracker Green 490/516: λ_ex_ = 488 nm, λ_em_ = 495-620 nm.

### Intracellular ROS detection

For the total ROS detection, HeLa cells were respectively incubated with 10 μM of DTPAP-2B NPs or DTPAP-2P NPs for 8 h followed by incubation with 20 μM DCFH-DA for 30 min. After being washed by PBS for three times, cells were irradiated by white light at a power density of 10 mW cm^-2^ for 1 min. Then, the fluorescence was immediately observed using an inverted fluorescent microscope with the excitation wavelength of 426-466 nm, and emission collection wavelength was 500-550 nm. For •OH detection, HeLa cells were incubated with 10 μM of DTPAP-2B NPs or DTPAP-2P NPs for 8 h followed by incubation with 10 μM APF for another 30 min. After being washed by PBS for three times, cells were irradiated by 660 nm laser (0.1 W cm^-2^) for different times. Then, the green fluorescence was immediately observed using an inverted fluorescent microscope with the excitation wavelength of 426-466 nm. Emissions at 500-550 nm were recorded. For O_2_^•−^ detection, HeLa cells were incubated with 10 μM of DTPAP-2B NPs or DTPAP-2P NPs for 8 h, followed by incubation with 10 μM DHE for another 30 min. After being washed by PBS for three times, cells were irradiated by 660 nm laser (0.1 W cm^-2^) for different times. Then the cells were immediately observed using an inverted fluorescent microscope with the excitation wavelength of 511-551 nm, and emission collection wavelength. Emissions at 573-613 nm were collected.

### Cell viability

4T1 cells or HeLa cells were seeded on 96-well plates in RPMI 1640 medium or DMEM medium (with 10% FBS, 1% penicillin/streptomycin) in 5% CO_2_, 20% O_2_ at 37 °C in a humidified incubator. Then, the cells in a logarithmic growth phase were harvested and seeded in 96-well plates at a density of 5 × 10^3^ cells for 24 h incubation. Subsequently, the medium was replaced with the fresh medium containing different concentrations of DTPAP-2B NPs or DTPAP-2P NPs. After further incubation for 20 h, the cells were exposed to 660 nm laser irradiation (0.4 W cm^-2^) for 10 min. Meanwhile, the DTPAP-2B NPs or DTPAP-2P NPs-incubated cells without laser irradiation were also conducted for the dark cytotoxicity study. After further incubation for 4 h, MTT was added and cells were incubated for another 4 h in the dark. Finally, the absorbance of the products was measured at a wavelength of 490 nm by a microplate reader. The results were expressed as the viable percentage of cells after different treatments relative to the control cells without any treatment. The relative cell viability was calculated according to the following formula (8):

8 $${\rm{Cell\; viability}}(\%)=({{\rm{OD}}}_{{\rm{sample}}}-{{\rm{OD}}}_{{\rm{background}}})/({{\rm{OD}}}_{{\rm{control}}}-{{\rm{OD}}}_{{\rm{background}}})\times 100\%$$

For single PTT: 0.5 mM radical scavenger Vc was co-incubated with 4T1 cells to eliminate the influence of PDT. For single PDT: HeLa cells were kept at 4 °C ice-water bath during laser irradiation to eliminate the influence of PTT.

### Live/dead cell staining

First, 4T1 cells were seeded on 6-well plate at a density of ~10^5^ cells per well and cultured in incubator for 24 h. Second, the culture medium was replaced with different concentrations (0, 90, or 150 μM) of DTPAP-2B NPs or DTPAP-2P NPs, and incubated continuously for about 12 h in dark condition. Third, the cells were exposed to 660 nm laser (0.4 W cm^-2^) or in dark conditions for 10 min, and after the treatment, the cells were further cultured for 4 h. Finally, the cells were rinsed with PBS and stained by using 1 μM Calcein-AM and 1 μM propidium iodide (PI) for 30 min, and the residual dyes were washed out by PBS for three times. The inverted fluorescent microscope was applied to observe the green fluorescence of Calcein-AM and red fluorescence of PI indicating live and dead cells, respectively. Conditions: excitation wavelength: 426-466 nm for Calcein-AM, 500-550 nm for PI; emission filter: 511-551 nm for Calcein-AM, 573-613 nm for PI.

### Animals

Female nude mice and BALB/c mice (6 − 8 weeks old, average body weight 16 − 18 g) were purchased from SPF Biotechnology Co., Ltd. (Beijing, China). All procedures were approved by the Institutional Animal Care and Use Committee at the Inner Mongolia University (IMU-2023-mouse-017). All mice were acclimatized to the animal facility for one week prior to experimentation and housed under pathogen-free conditions, fed under conditions of 25 °C and 55% of humidity and allowed free access to standard laboratory water and chow. The maximal allowable tumor size was set at 2000 mm^3^ or 20 mm in diameter as per the committee’s guidelines. During the experiments, the tumor size was monitored regularly, and at no point did it exceed the permitted limit. Female mice were used in this study. This selection was based on the majority of existing literatures. This study results were independent of sex and gender. Therefore, sex disaggregation of data was not applicable. At the end of the experiments, all animals were humanely euthanized by carbon dioxide inhalation followed by cervical dislocation. Euthanasia was performed by trained personnel in accordance with the guidelines of the Institutional Animal Care and Use Committee at the Inner Mongolia University (IMU-2023-mouse-017).

### In vivo NIR-II fluorescence imaging of blood vessels

The NIR-II fluorescence images of blood vessels in nude mice were recorded on the commercial NIR-II Small Animal Imaging System Series III 900/1700S (Suzhou NIR-Optics Technology Co., Ltd., China) equipped with 900 nm long-pass (LP) filters after intravenous injection of DTPAP-2P NPs (600 μM, 200 μL).

### Tumor xenograft model and imaging

To establish the 4T1 tumor-bearing mouse models, 4T1 breast cancer cells (1 × 10^6^) suspended in 100 μL PBS were injected subcutaneously into the right flanks of each mouse (BALB/c mice). After about 7 days, mice with tumor volumes of about 100 mm^3^ were used subsequently. The 4T1 tumor-bearing mice were administered with DTPAP-2P NPs in PBS (600 μM, 50 μL) through intratumor injection. Then, the fluorescence images of mice were obtained as a function of post injection times (0, 1, 3, 5, 7, 9, 12, 24, 48, 60, 72, 96 h) with 900 nm long-pass filter.

### Photothermal imaging

The 4T1 tumor-bearing BALB/c mice were intratumorally injected with DTPAP-2P NPs (600 μM, 50 μL). The infrared thermal images of mice were acquired by using an FLIR E8-XT camera with 660 nm laser (0.4 W cm^-2^) irradiation for 10 min at 3 h after administration of DTPAP-2P NPs. Mice injected with PBS under the same irradiation condition were used as the control.

### In vivo synergistic photodynamic and photothermal cancer therapy

The 4T1 tumor-bearing BALB/c mice were randomly divided into 4 groups (*n* = 5 for each group), which were named “PBS”, “PBS + L”, “NPs” and “NPs + L”, respectively. For “PBS” and “NPs” groups, 50 μL of PBS or DTPAP-2P NPs (600 μM) were intratumorally injected into the mice, respectively, without further laser irradiation. While for “PBS + L” and “NPs + L” groups, the mice were further irradiated by 660 nm laser (0.4 W cm^-2^) for 10 min at 3 h post-injection of PBS or DTPAP-2P NPs (50 μL, 600 μM), respectively. Tumor volumes were measured and calculated every other day using a caliper and calculated using the following formula (9):

9 $${\rm{volume}}=(({\rm{tumor\; length}})\times ({\rm{tumor\; width}})2)/2.$$

### Immunofluorescence Staining of HSP60, CRT and HMGB1

After treatments, the tumors from the various groups were collected and instantly fixed in a 4% formaldehyde solution. Then the tumor tissue was sectioned and blocked with 3% BSA for 30 min. Afterward, the blocking solution was removed, and the sections were placed flat in a wet box. Primary antibodies: CRT (Beyotime, Shanghai, China, catalog no. AF1666, rabbit polyclonal; IF 1:200), HMGB1 (Servicebio, Wuhan, China, catalog no. GB11103, rabbit polyclonal; IF 1:500) or HSP60 (Servicebio, Wuhan, China, catalog no. GB15243, mouse monoclonal; IF 1:500) were added and incubated overnight at 4 °C. The next day, the sections were washed three times with PBS on a shaking incubator for 5 min each time. Subsequently, the following secondary antibodies were added: Alexa Fluor® 488-conjugated Goat Anti-Rabbit IgG (H + L) (Servicebio, Wuhan, China, catalog no. GB25303; 1:400), Cy3-conjugated Goat Anti-Rabbit IgG (H + L) (Servicebio, Wuhan, China, catalog no. GB21303; 1:300) or Alexa Fluor® 488-conjugated Goat Anti-Mouse IgG (H + L) (Servicebio, Wuhan, China, catalog no. GB25301; 1:400), and incubated at room temperature in the dark for 50 min. The samples were then washed three times with PBS, each for 5 min. Thereafter, DAPI stain was added and incubated at room temperature in the dark for 10 min. The sections were washed three times with PBS, each for 5 min. Solution B of the autofluorescence quencher was added dropwise, and incubated at room temperature for 5 min, followed by rinsing under running water for 10 min. The sections were mounted with an anti-fluorescence quenching mountant. Finally, the tissue sections were imaged using a fluorescence microscope^55,85^.

### Hemocompatibility test

Whole blood was collected from healthy mice (2000 rpm, 10 min). PBS was used to wash the obtained erythrocytes for three times. Subsequently, 25 µL of blood cells were treated with varying concentrations of DTPAP-2P NPs (0, 30, 60, 90, 120, 150 µM) in polyethylene pipe containing 500 μL of PBS, and incubated for 2 h at 37 °C. PBS and 1% Triton X-100 (Triton) were used as negative control and positive control, respectively. Then, all samples were centrifuged for 10 min, and images were captured. The absorbance of the supernatant was measured at 540 nm. To correct for compound absorbance, DTPAP-2P NPs in PBS solution with the same concentration was used as the blank group. Hemolysis was calculated using the following formula (10):

10 $${\rm{Hemolysis}}\left(\%\right)=\frac{{A}_{{Blood}}-{A}_{{Sample}}}{{A}_{{Triton}}-{A}_{{PBS}}}\times 100\%$$

### Histological examination

After 15 days of treatment, the mice of various groups were sacrificed for further haematoxylin-eosin (H&E) staining and terminal deoxynucleotidyl transferase (TdT)-mediated dUTP nick-end labeling (TUNEL) analysis on tumor tissues and normal organs (heart, liver, spleen, lung, and kidneys).

### Biosafety evaluation

To further evaluate the safety of different treatments in vivo, blood samples were collected from mice with various treatments for complete blood panel analysis. Red blood cell (RBC), hemoglobin (HGB), hematocrit (HCT), mean corpuscular volume (MCV), mean corpuscular hemoglobin (MCH), mean corpuscular hemoglobin concentration (MCHC), red blood cell volume distribution width (RDW), platelets (PLT) and mean platelet volume (MPV) were measured. For biochemical blood analysis, alanine aminotransferase (ALT), transaminase (AST), albumin (ALB), blood urea nitrogen (BUN), creatinine (CREA) and uric acid (UA) were measured.

### Statistical analysis

Origin was used for all data analysis and graph plotting. Data were presented as mean values ± SEM. The *P* values were calculated by one-way ANOVA and multiple-comparison test (two-sided). Exact *P* values were provided accordingly in the figures or captions. *P* < 0.05 was used as the threshold for statistical significance; (****) indicates *P* < 0.0001. The exact number of replicates and statistical tests are indicated in the figure legends. Unless otherwise indicated, n represents the number of independent experimental replicates.

### Reporting summary

Further information on research design is available in the Nature Portfolio Reporting Summary linked to this article.

## Supplementary information

Supplementary Information

Description of Additional Supplementary Files

Supplementary Data 1

Reporting Summary

Transparent Peer Review file

## Source data

Source Data

## Data Availability

All original data generated in this study are provided in the Supplementary Information/Source Data file. The X-ray crystallographic coordinates for structures reported in this study have been deposited at the Cambridge Crystallographic Data Center (CCDC), under deposition number 2387016 for DTPAP-2B and 2466906 for DTPAP-2P. These data can be obtained free of charge from The Cambridge Crystallographic Data Center via (www.ccdc.cam.ac.uk/data_request/cif). There are no restrictions on data availability in the current work. Source data are provided with this paper. Correspondence and requests for materials should be addressed to Jianguo Wang. Source data are provided with this paper.
