# Supplementary material for: Organic planar π-conjugated anions enable structural design of anion-π+ NIR-II phototheranostic agents
Source: Nat Commun. 2026 Jul 8;17:8449. doi: 10.1038/s41467-026-75247-7 (PMC13478202; doi:10.1038/s41467-026-75247-7)
Supplement: Supplementary file 1 [file 41467_2026_75247_MOESM1_ESM.pdf]

Supplementary Information

Organic Planar π-Conjugated Anions Enable Structural Design of Anion-π^+ NIR-II Phototheranostic Agents

Ying Zhang[1], Mingyang Han [1], Guoyu Jiang [1,*], Lingxiu Liu [1], Chunbin Li [1], Lina Feng [1], Jianye Gong [1], Jianguo Wang [1,*], Ben Zhong Tang [2,*]

[1]College of Chemistry and Chemical Engineering, Institute of Green Chemistry and Environment, Institutes of Biomedical Sciences, Inner Mongolia Key Laboratory of Synthesis and Application of Organic Functional Molecules, Inner Mongolia University, Hohhot 010021, P. R. China

[2]Guangdong Basic Research Center of Excellence for Aggregate Science, School of Science and Engineering, The Chinese University of Hong Kong (Shenzhen), Longgang, Shenzhen, Guangdong 518172, P. R. China

[*]Corresponding Author. Email: jiangguoyu@mail.ipc.ac.cn (G. J.), wangjg@iccas.ac.cn (J. W.), tangbenz@cuhk.edu.cn (B. T.)

Supplementary Table 1. Comparison of parameters between the reported anion-π^{+} type PSs and DTPAP-2P in this study.

Ref.	Chemical structure	$\lambda_{ex}/\lambda_{em}$ (nm)	Light source	ROS type
S1	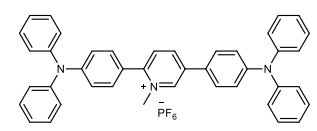	456/543<br>in THF	475 nm	-
S2	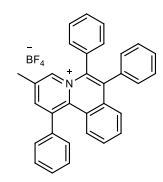	380/450<br>in water	365 nm	-
S3	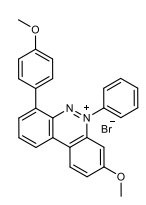	518/732<br>in water	white light	Type I
S4	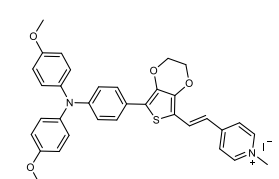	540/687<br>in DMSO/toluene	white light	Type I
S5	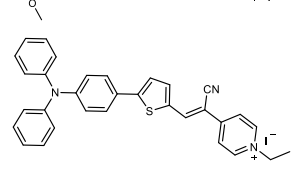	534/~725<br>in DMSO	white light	Type I
S6	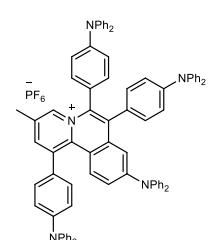	440/~496<br>in DMSO	white light	Type II
S7	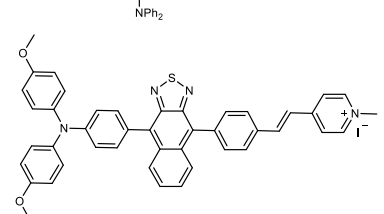	528/686<br>in THF	white light	Type I
S8	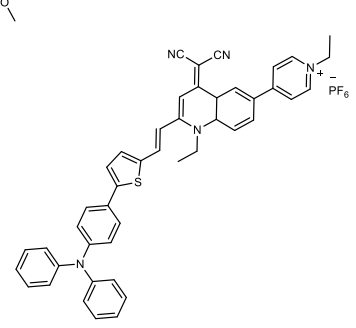	477/731<br>in water	white light	Type I/II
S9	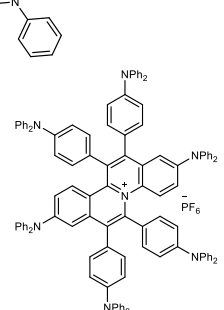	560/~650<br>(in THF)	white light	Type I/II
S10	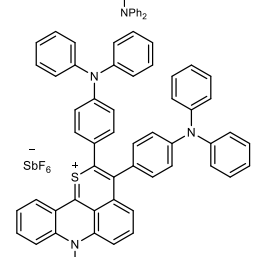	579/790<br>in PBS	635 nm laser	Type I
S11	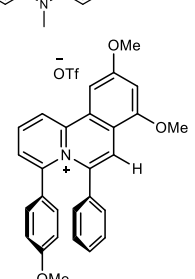	430/602<br>in DMSO	488 nm laser	-
S12	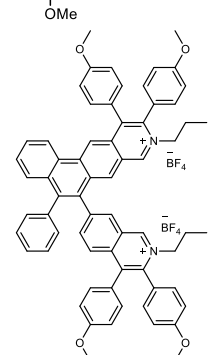	320/547<br>in DMSO	white light	Type I
This work	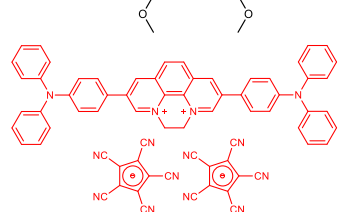	635/1000<br>in PBS	660 nm laser<br>(0.4 W/cm^2)	Type I/II

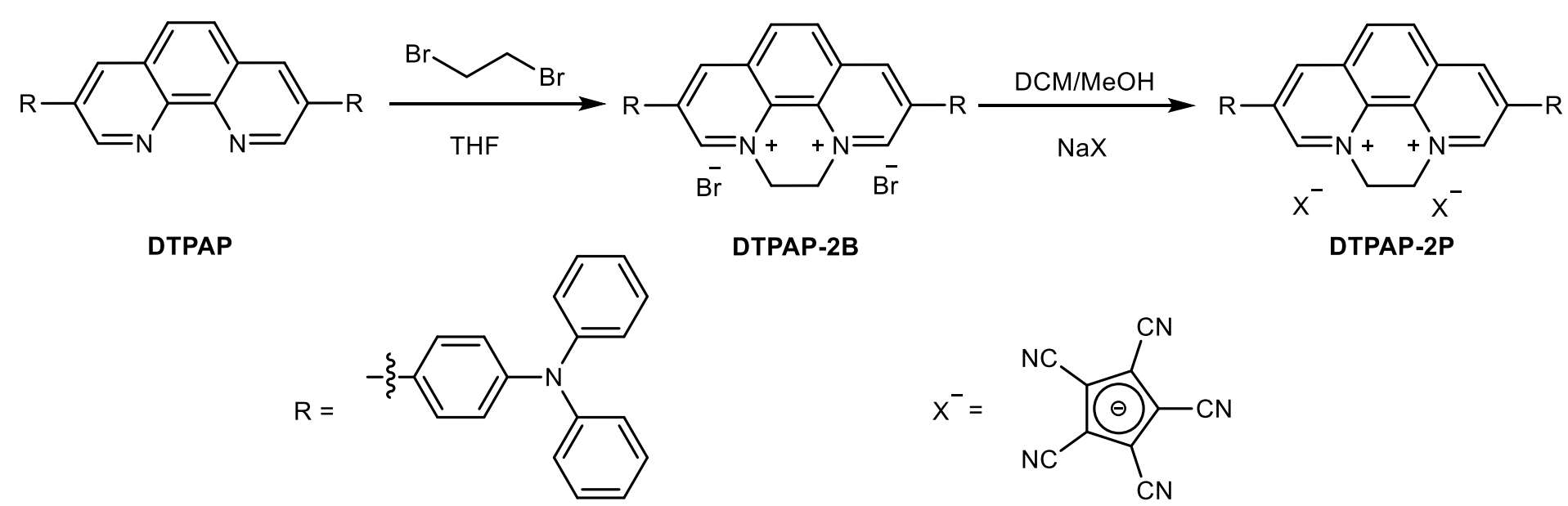

Supplementary Figure 1. General synthetic procedure for DTPAP-2B and DTPAP-2P.

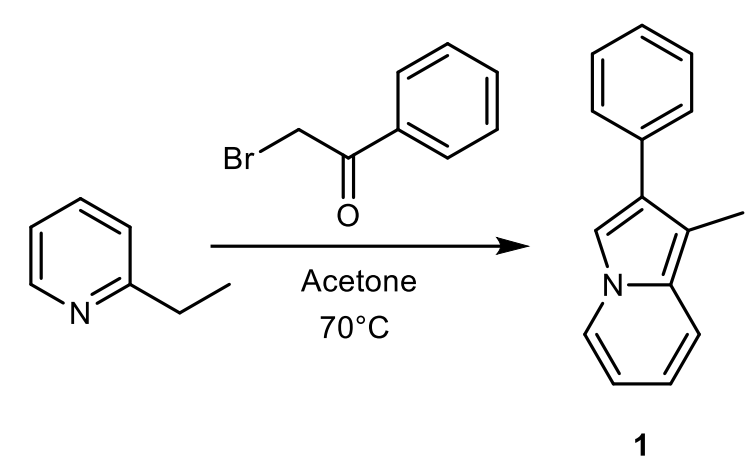

Supplementary Figure 2. General synthetic procedure for compound **1**.

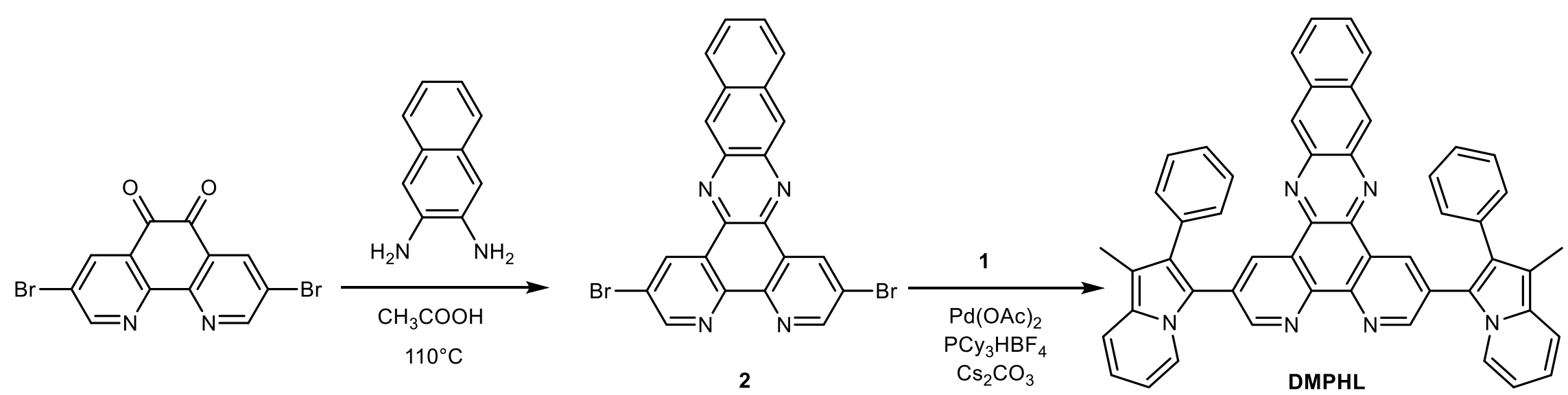

Supplementary Figure 3. General synthetic procedure for DMPHL.

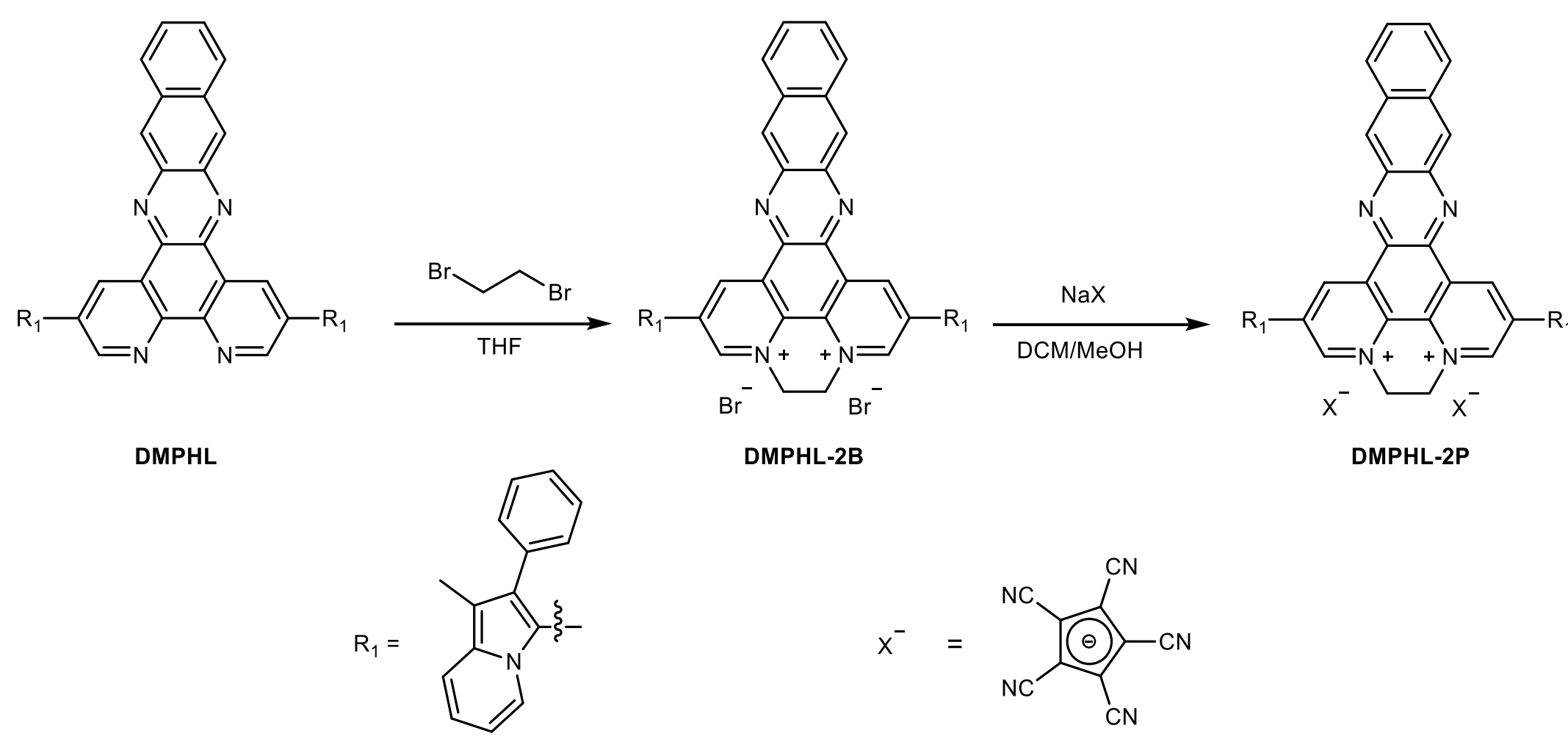

Supplementary Figure 4. General synthetic procedure for DMPHL-2B and DMPHL-2P.

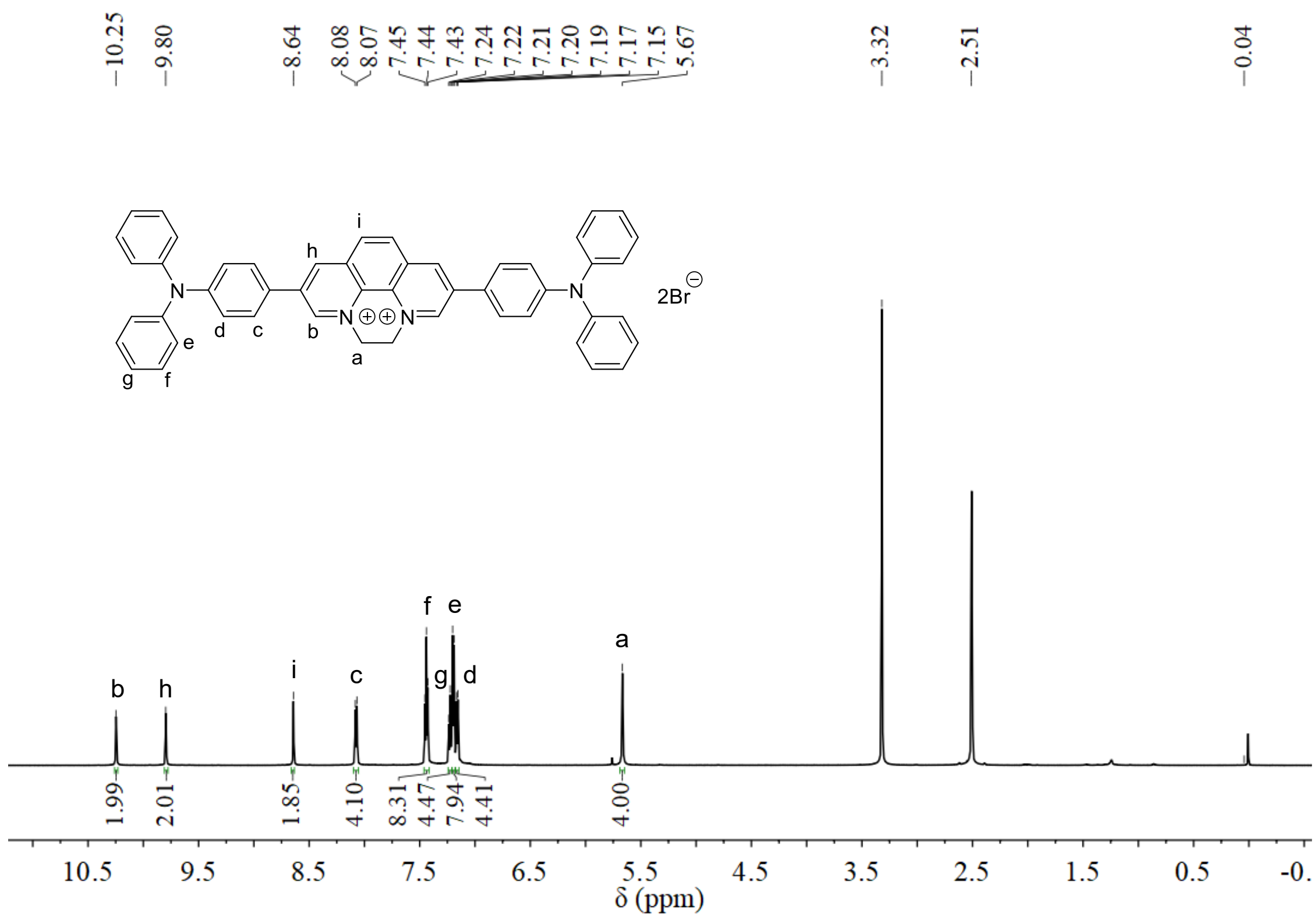

Supplementary Figure 5. ^{1}H NMR spectrum of DTPAP-2B in DMSO-d_6.

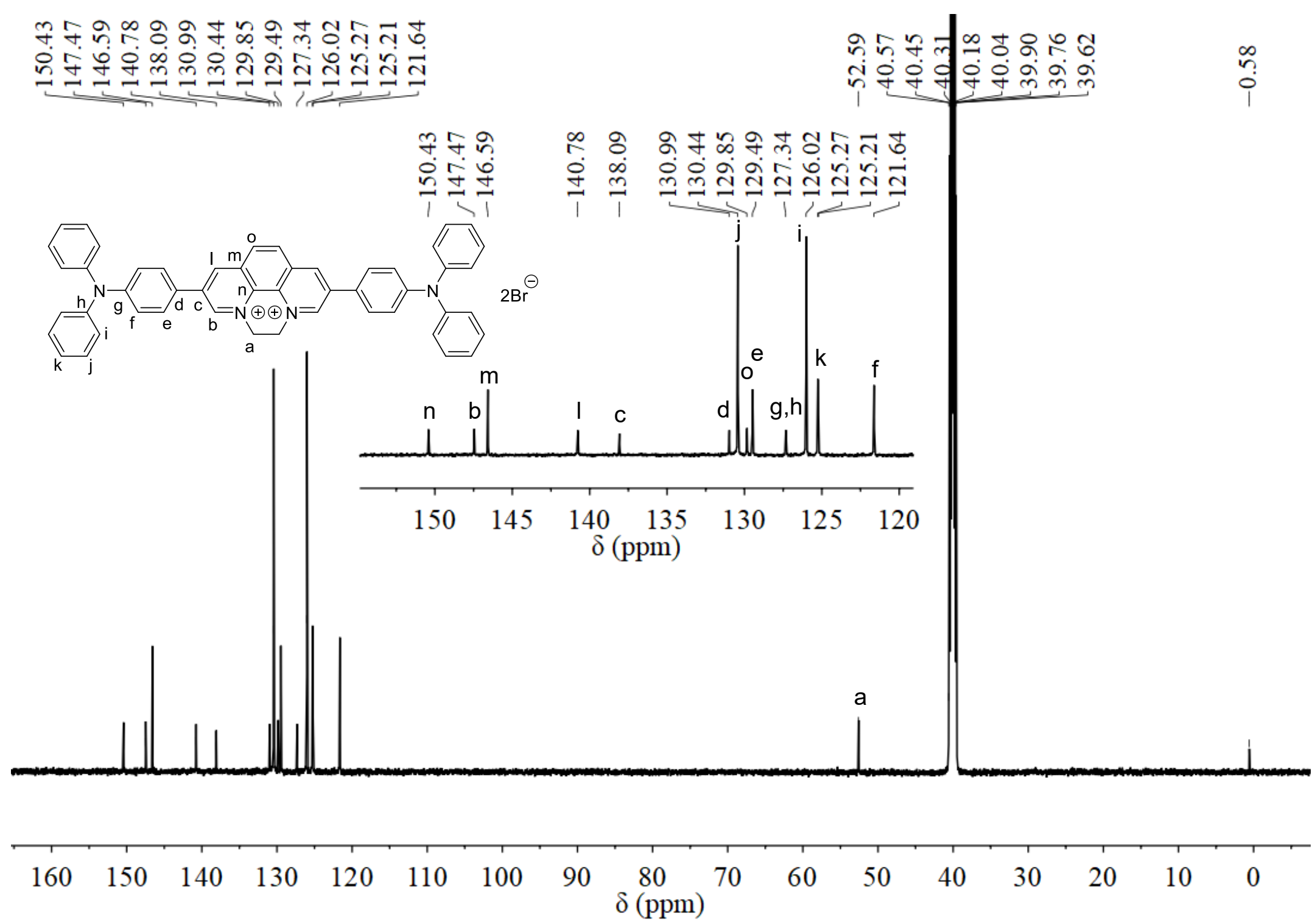

Supplementary Figure 6. ^{13}C NMR spectrum of DTPAP-2B in DMSO-d_6.

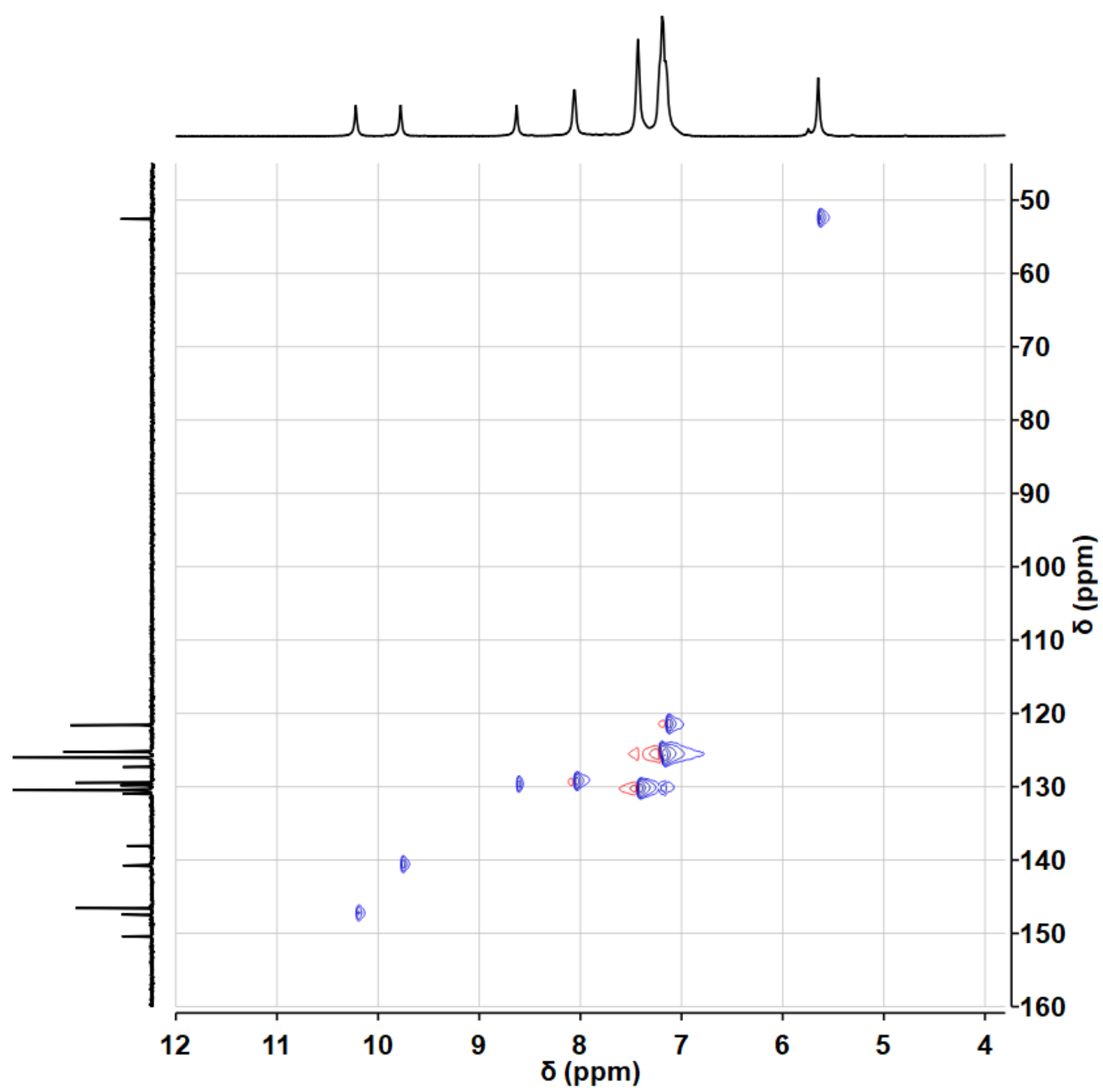

Supplementary Figure 7. 2D NMR characterization of DTPAP-2B between carbon atoms (^{13}C) and their directly attached protons (^{1}H) (HSQC).

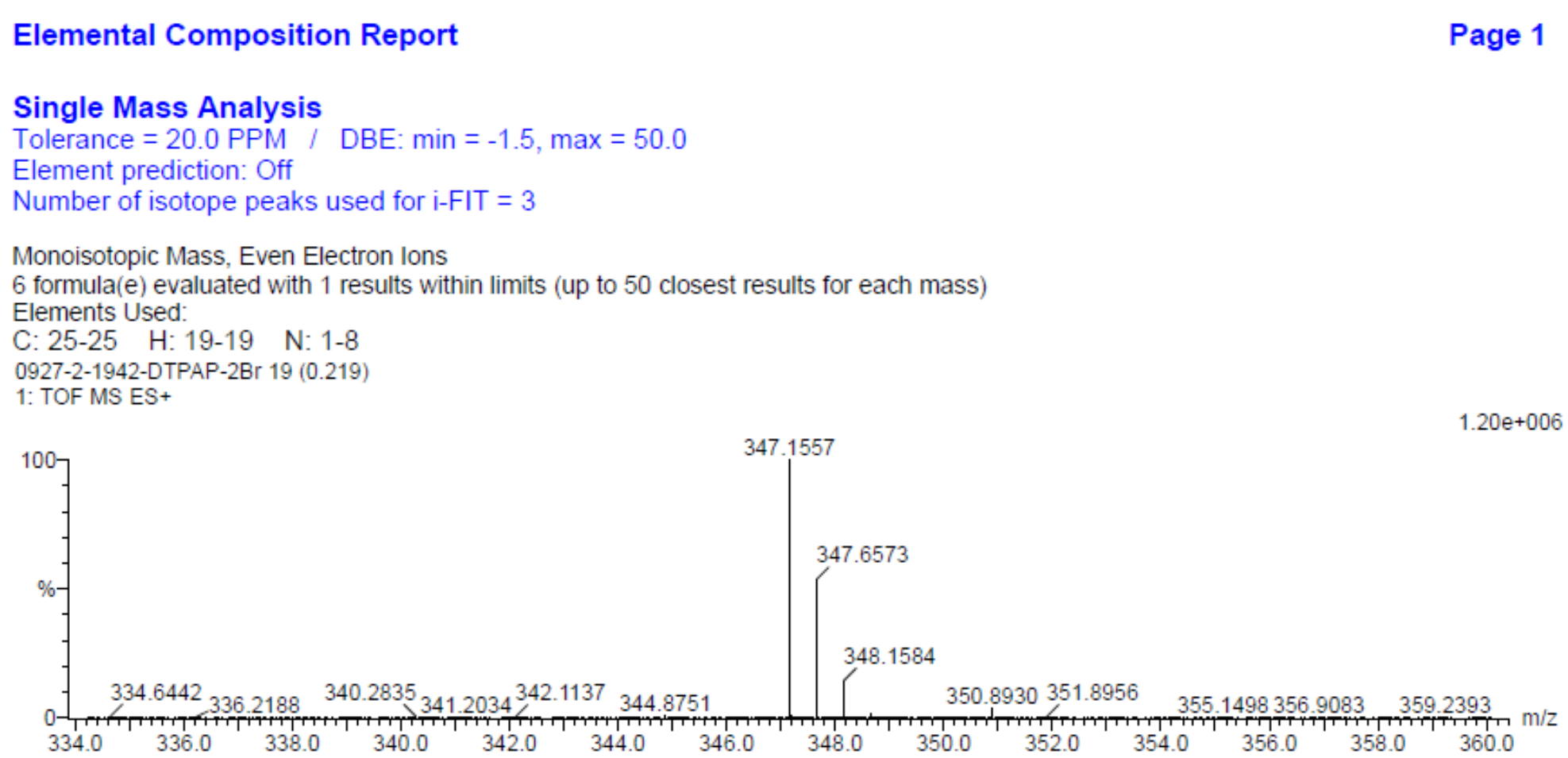

Supplementary Figure 8. HRMS spectrum of DTPAP-2B.

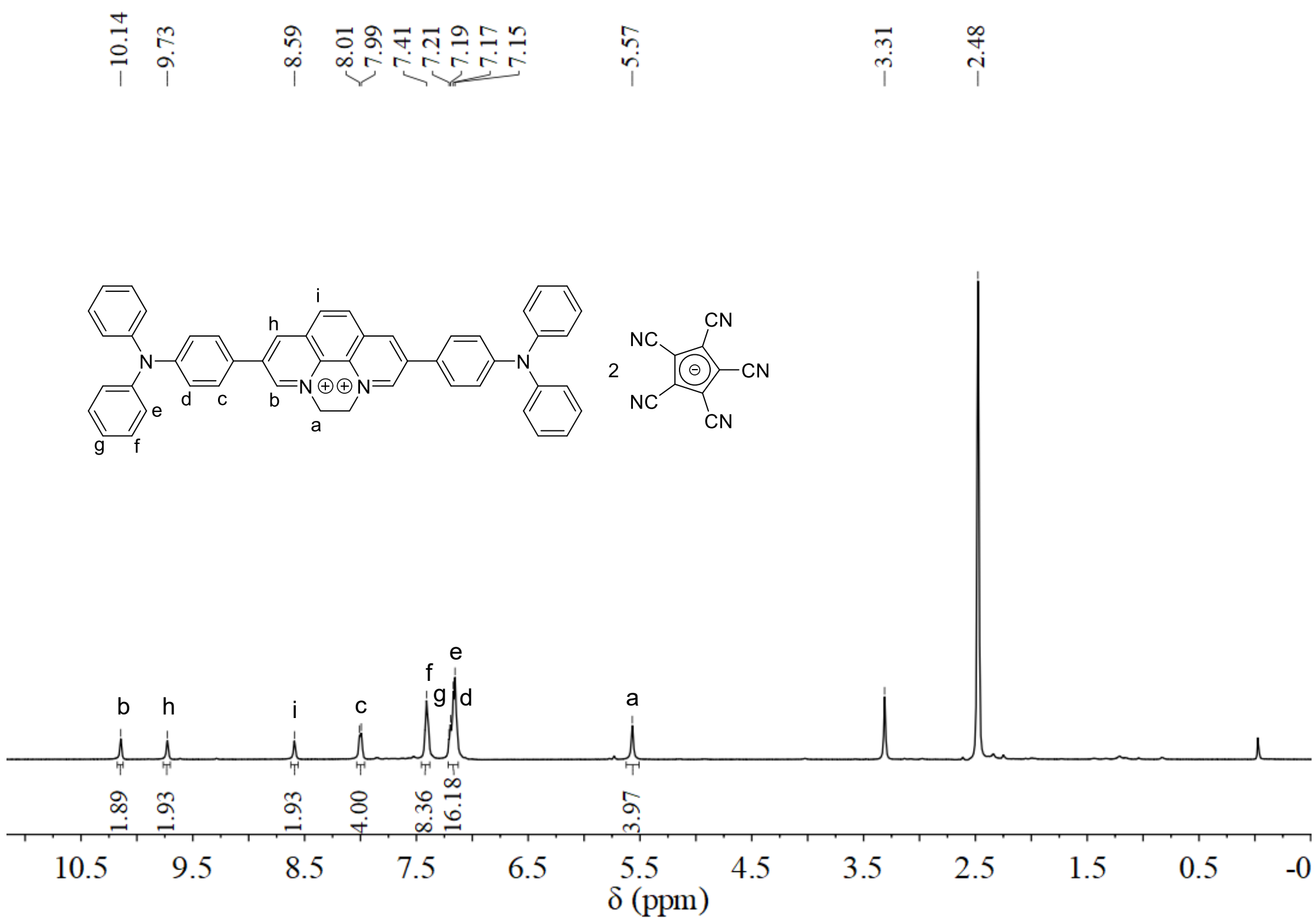

Supplementary Figure 9. ^{1}H NMR spectrum of DTPAP-2P in DMSO-d_6.

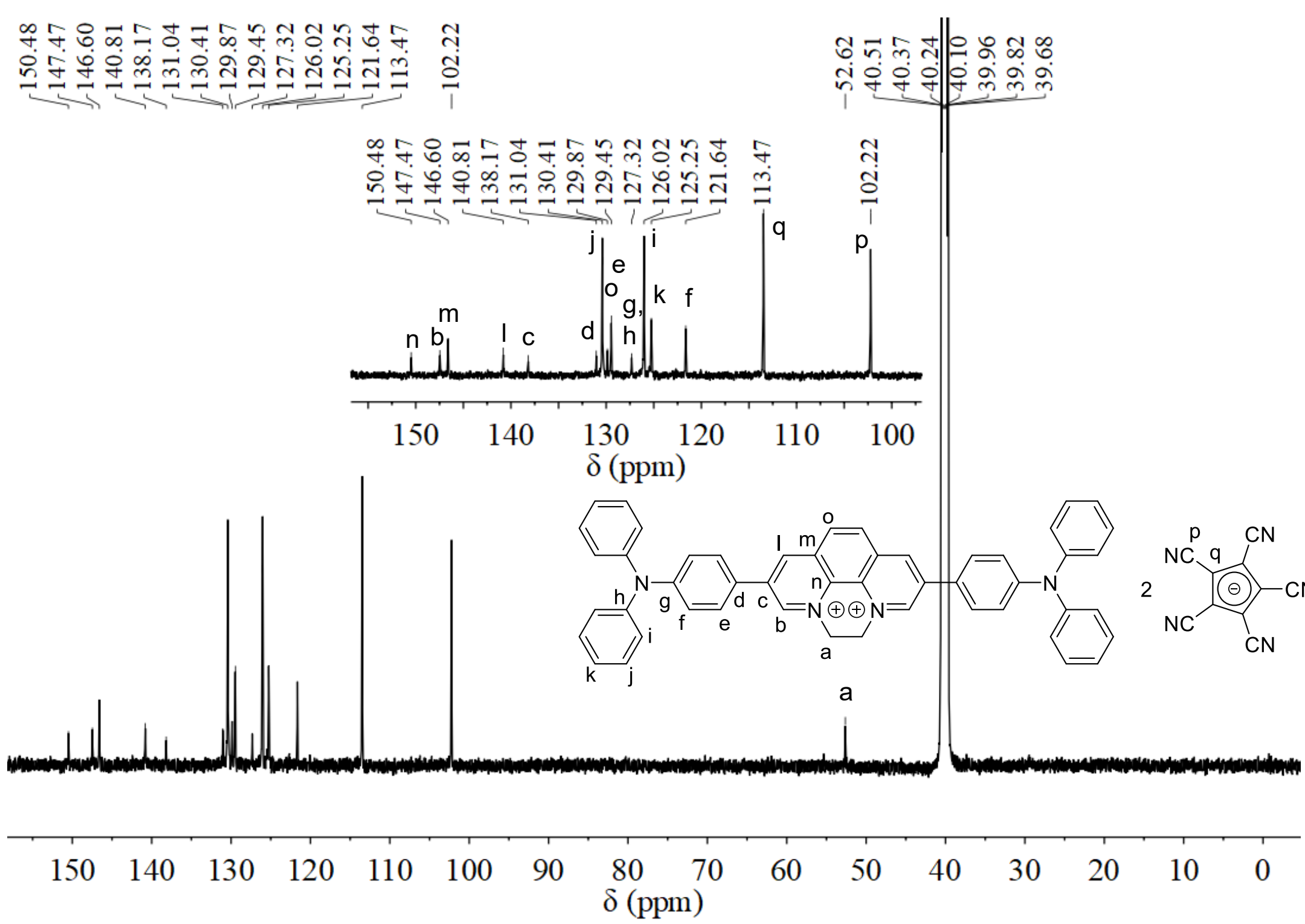

Supplementary Figure 10. ^{13}C NMR spectrum of DTPAP-2P in DMSO-d_6.

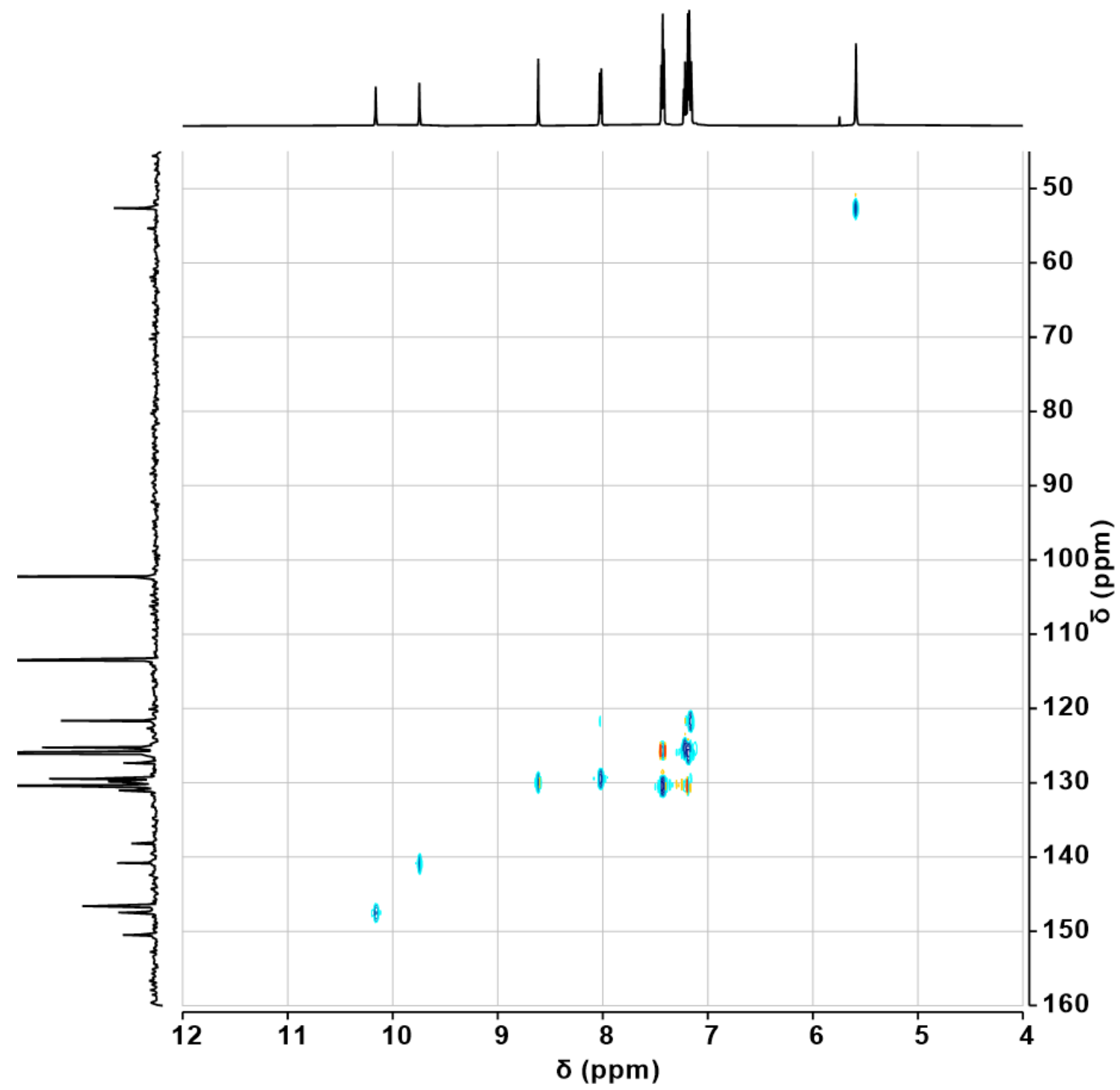

Supplementary Figure 11. 2D NMR characterization of DTPAP-2P between carbon atoms (^{13}C) and their directly attached protons (^{1}H) (HSQC).

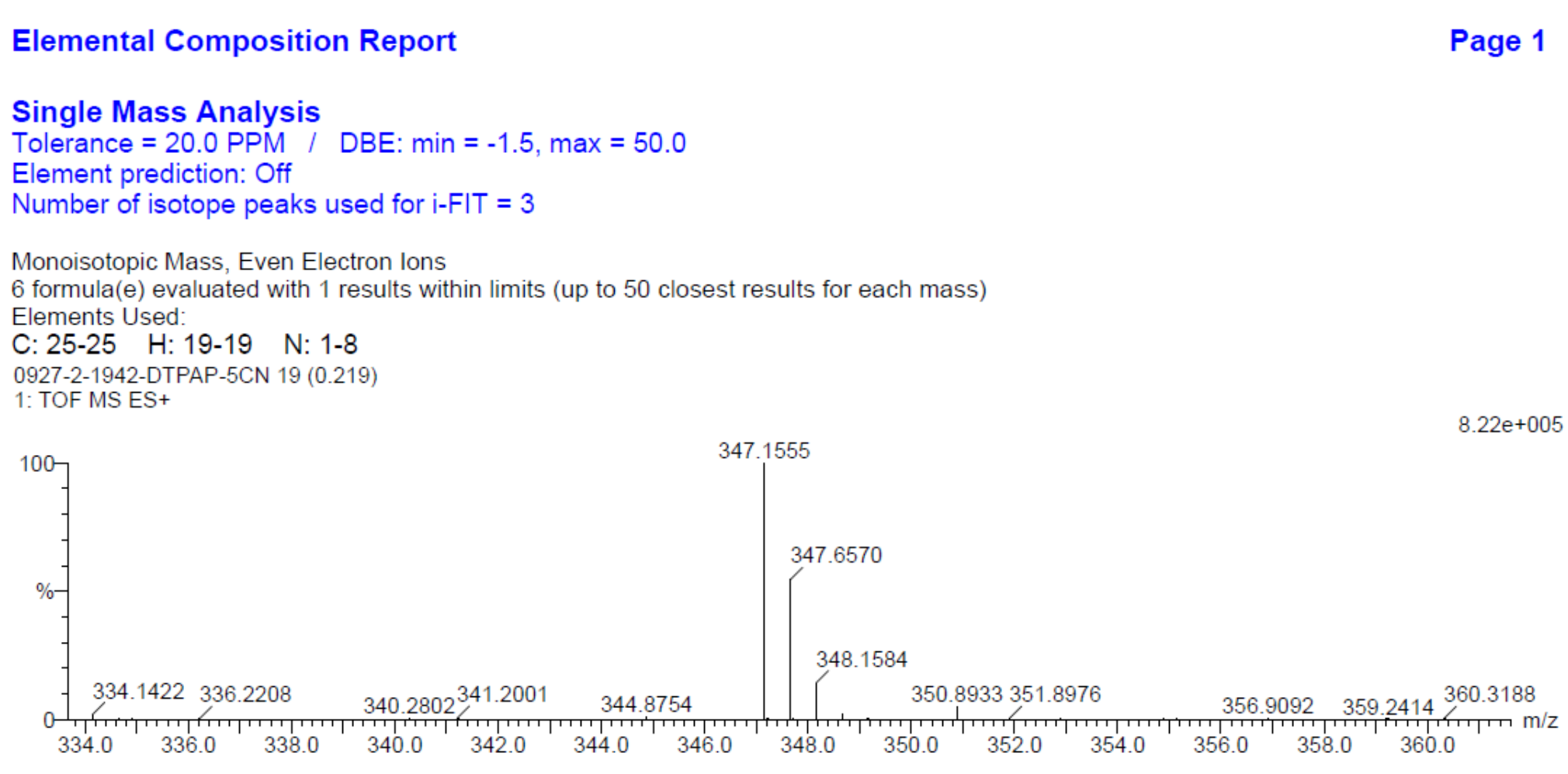

Supplementary Figure 12. HRMS spectrum of the cationic part of DTPAP-2P.

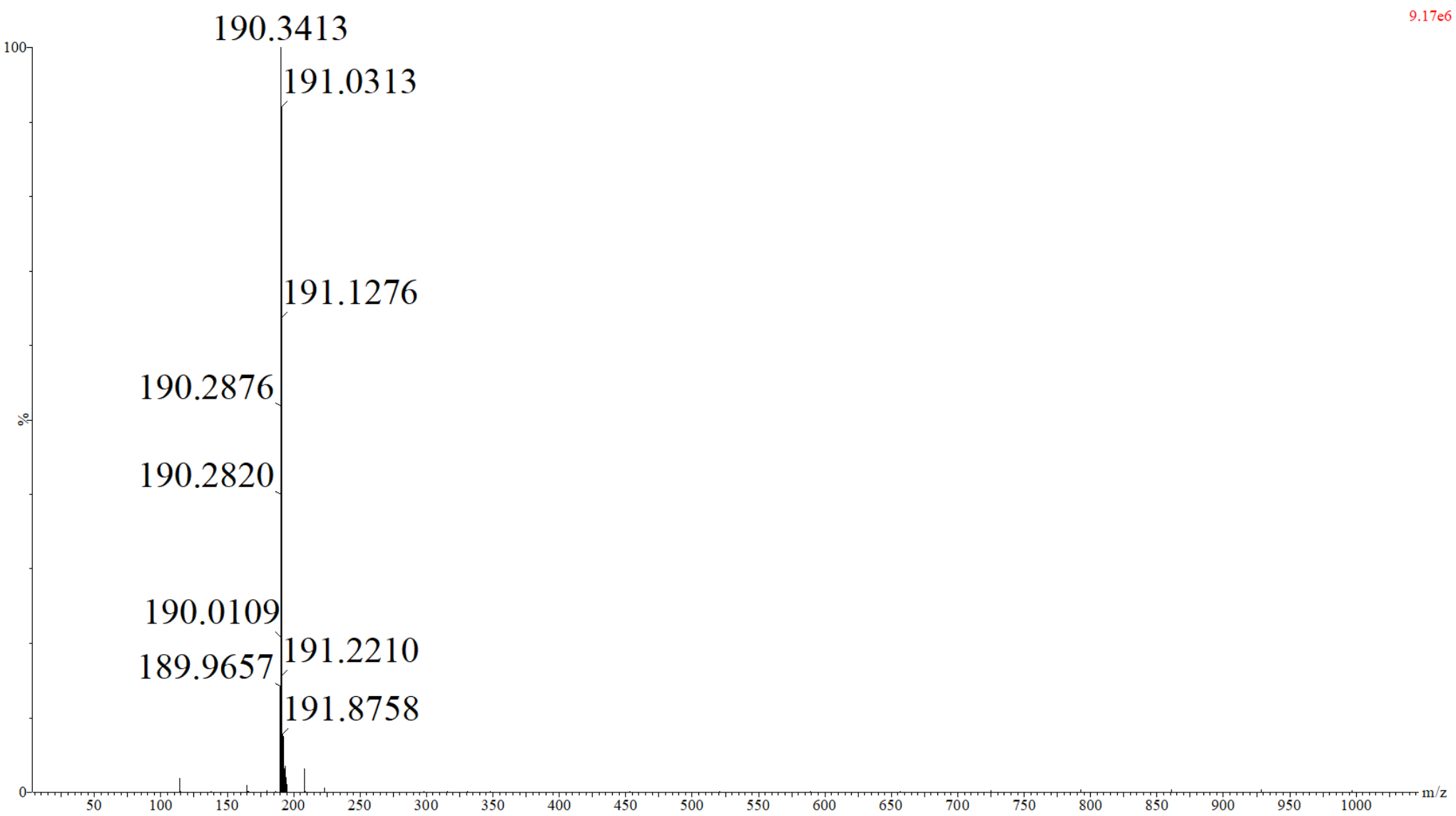

Supplementary Figure 13. HRMS spectrum of $(CCN)_5^-$ in DTPAP-2P.

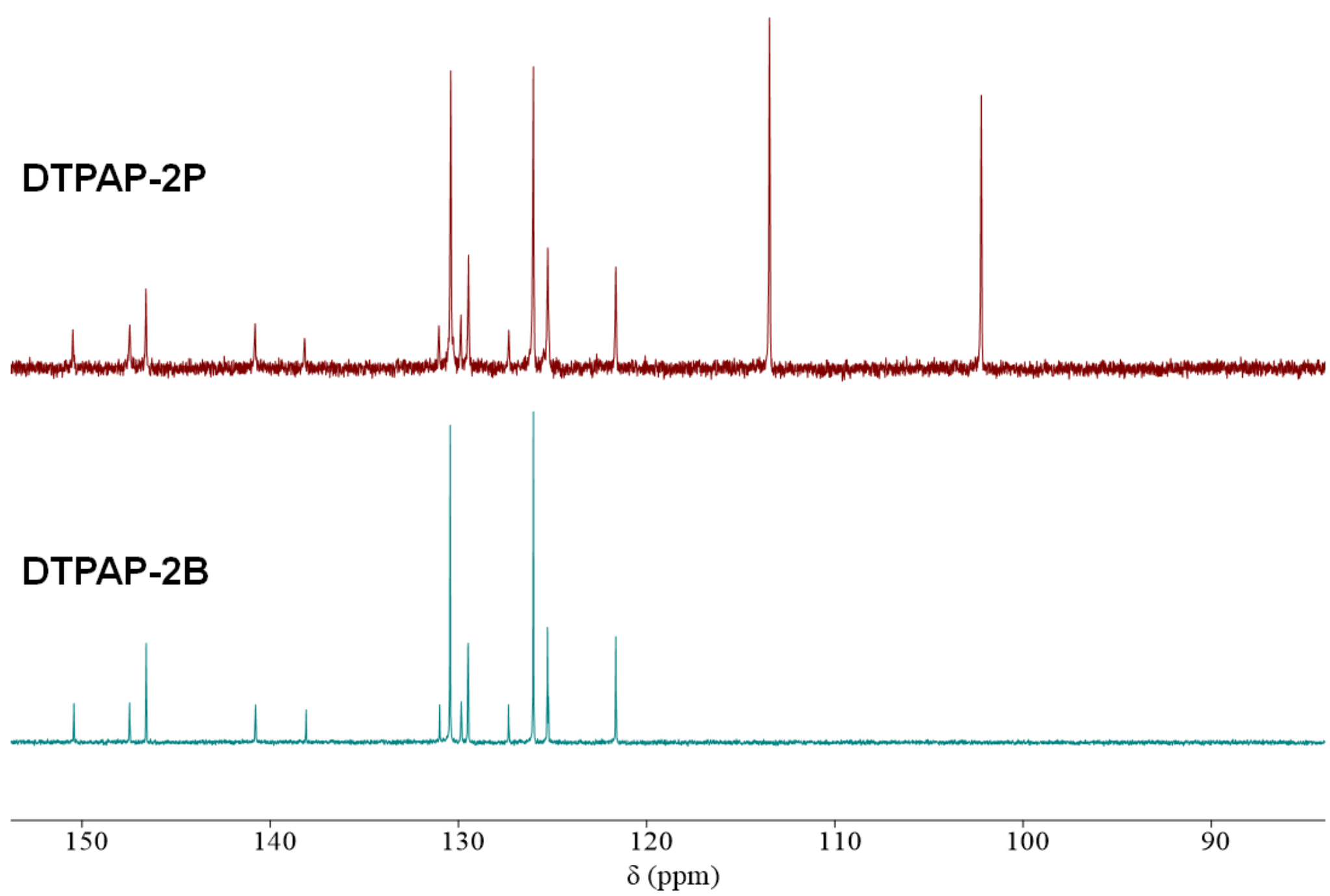

Supplementary Figure 14. The stacked ^{13}C NMR spectrum of DTPAP-2P and DTPAP-2B in DMSO-d_6.

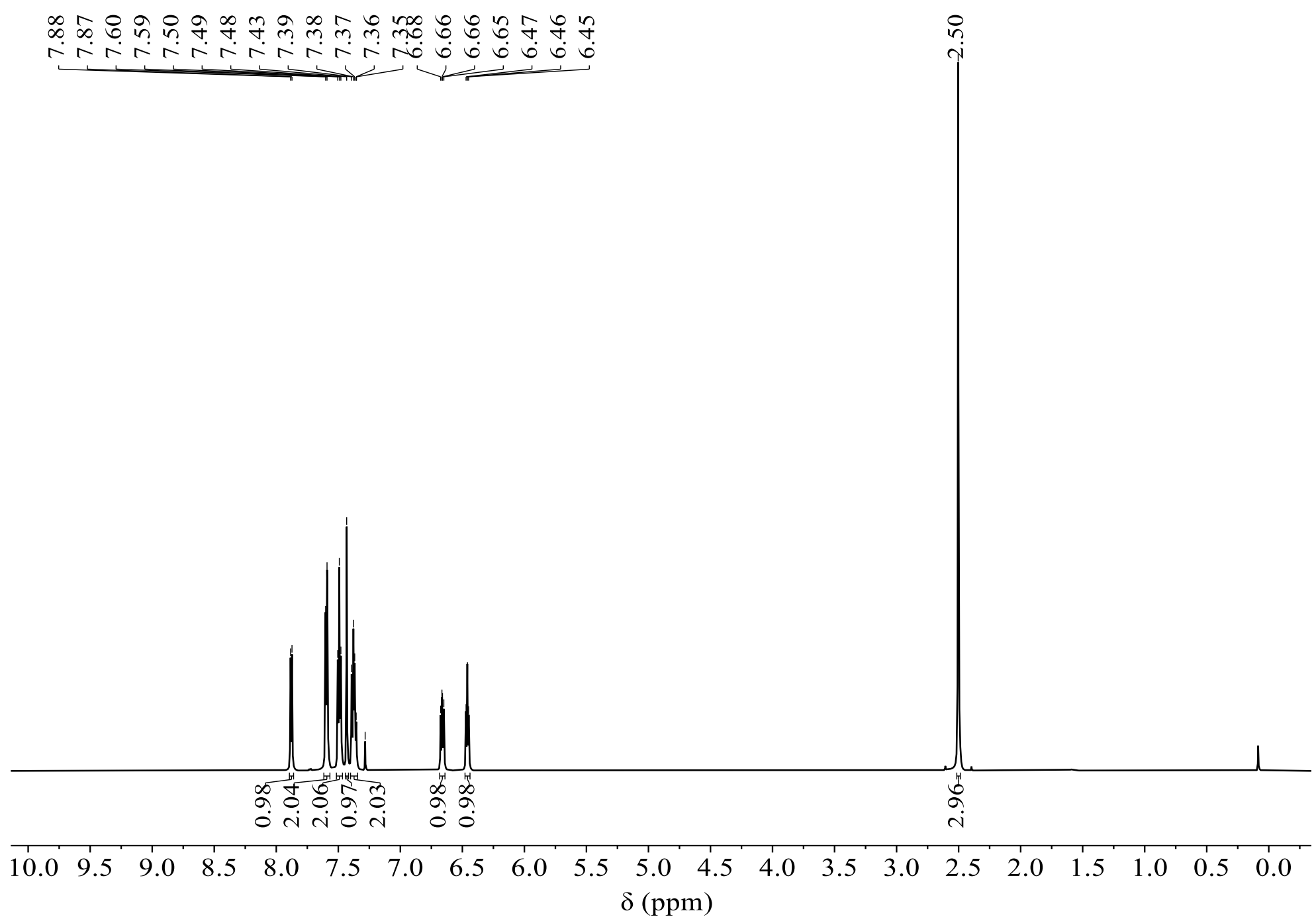

Supplementary Figure 15. ^{1}H NMR spectrum of **1** in $CDCl_3$.

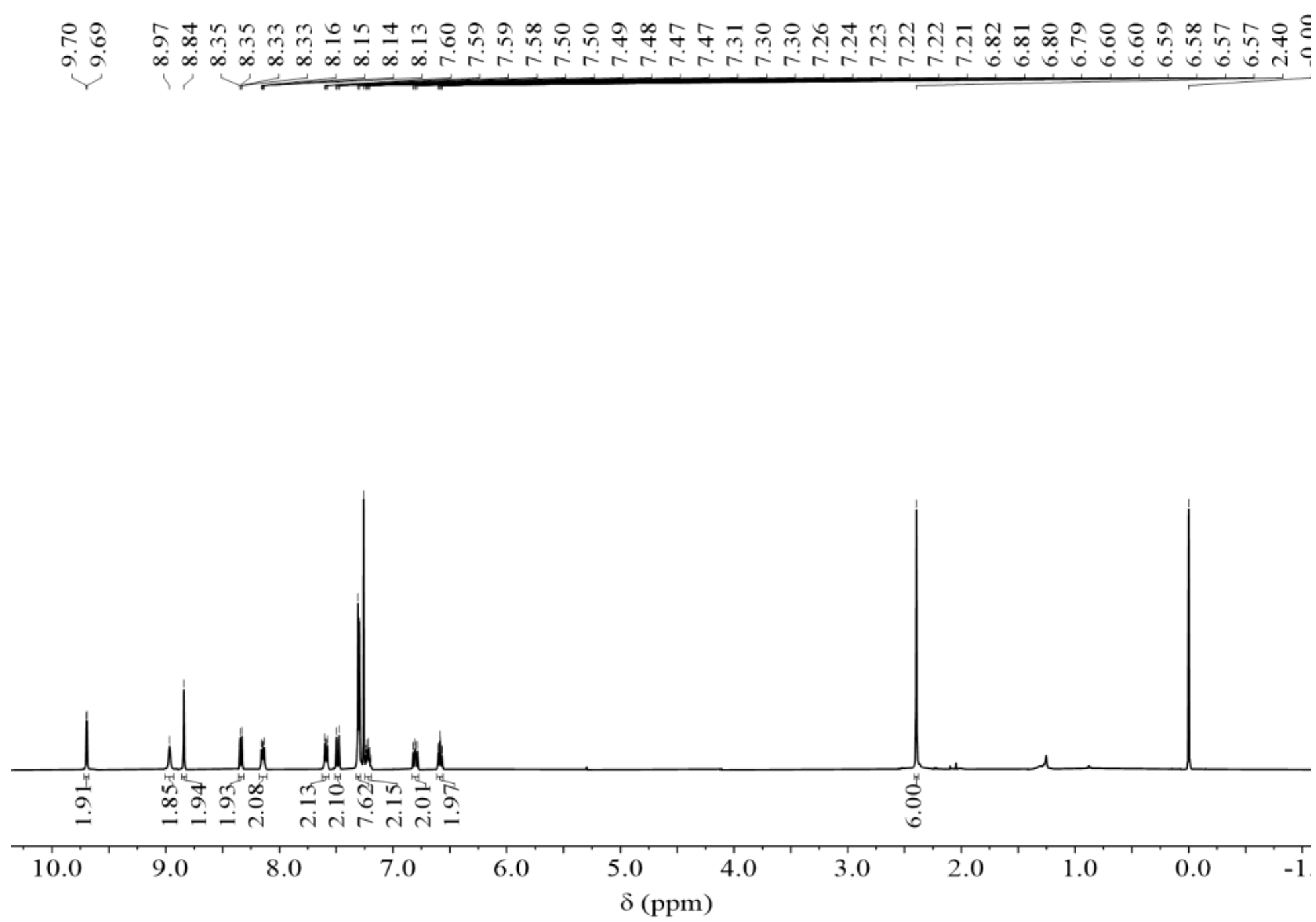

Supplementary Figure 16. ^{1}H NMR spectrum of DMPHL in $CDCl_3$.

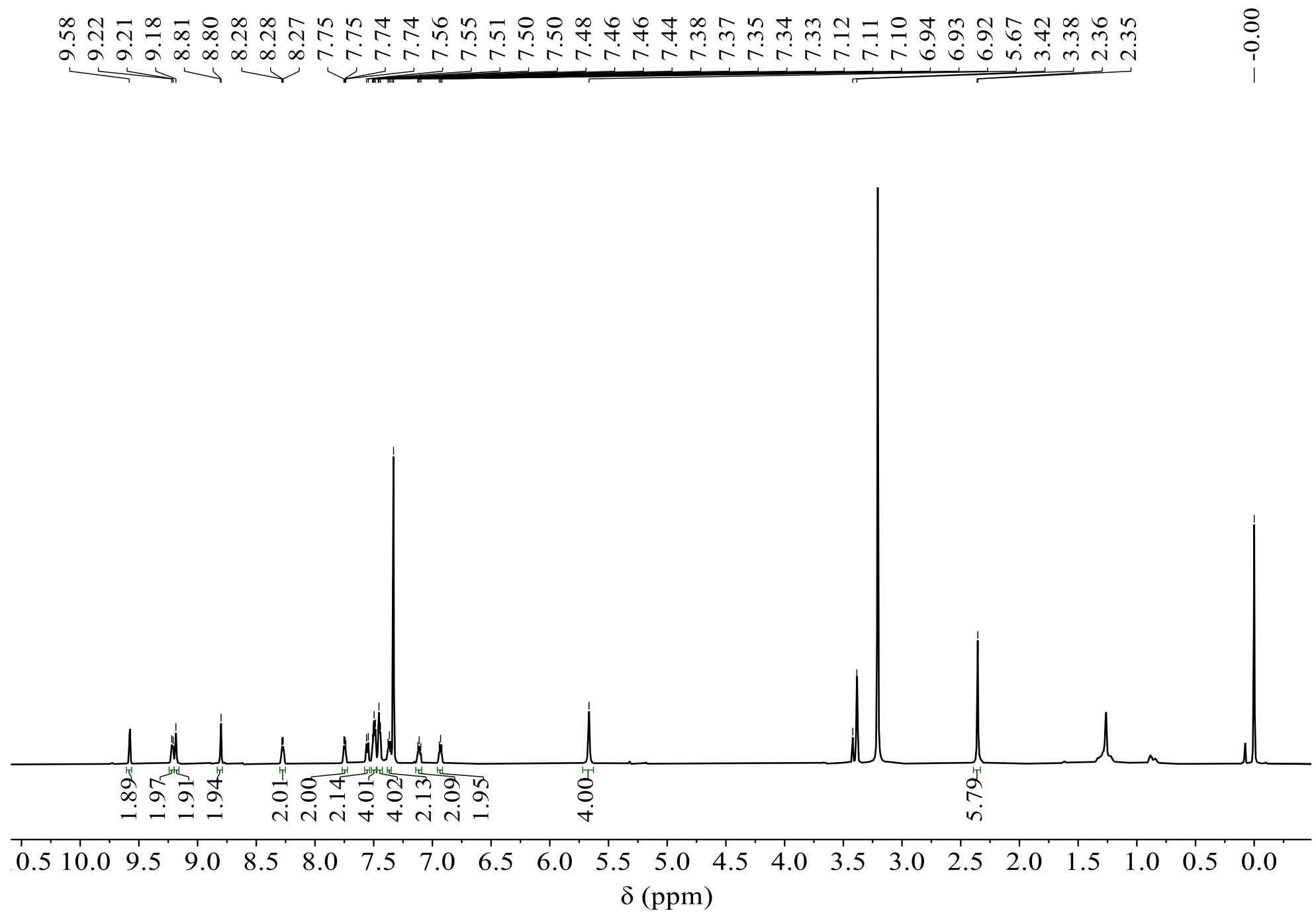

Supplementary Figure 17. ^{1}H NMR spectrum of DMPHL-2B in $CDCl_3$ and CD_3OD.

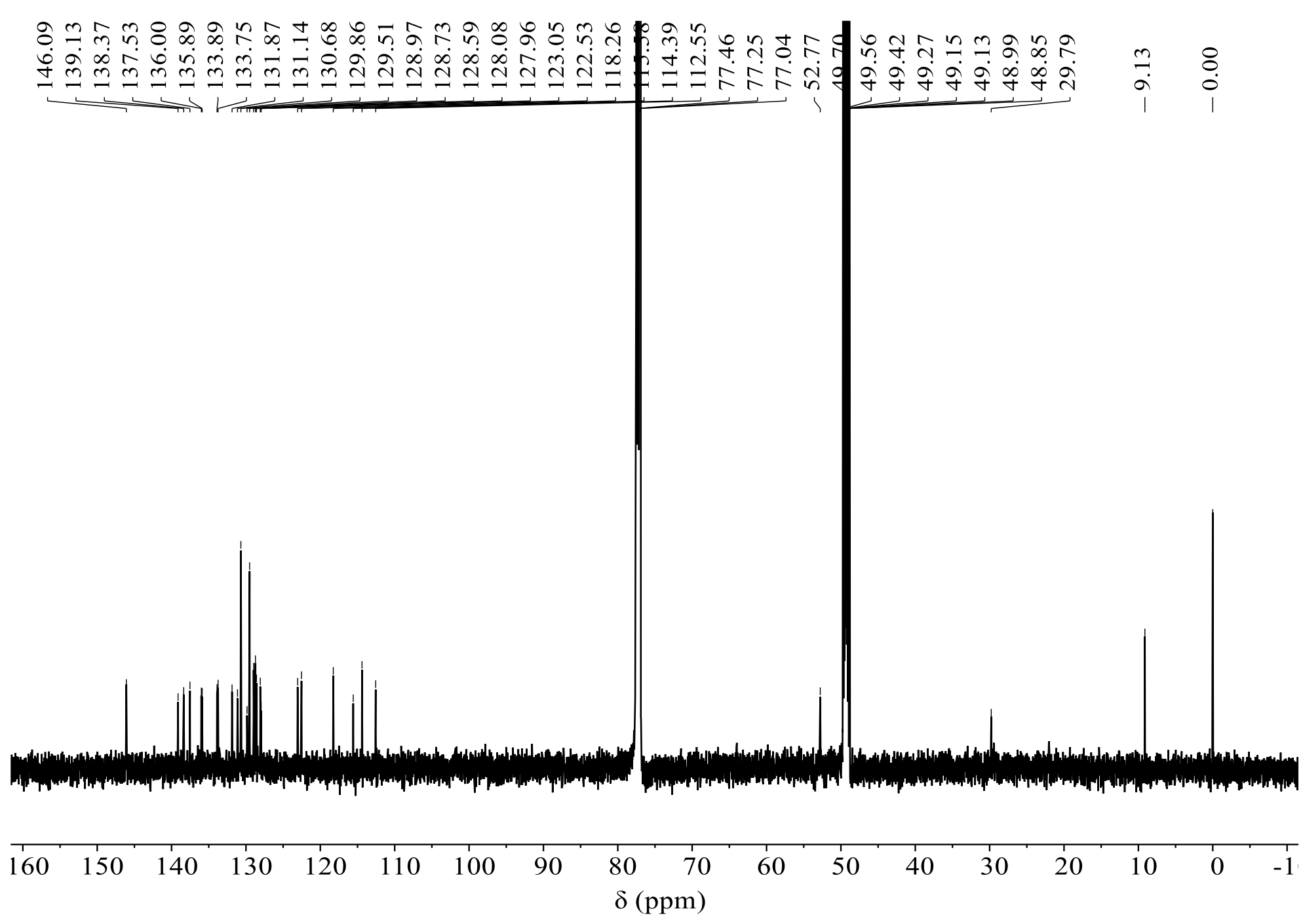

Supplementary Figure 18. ^{13}C NMR spectrum of DMPHL-2B in $CDCl_3$ and CD_3OD.

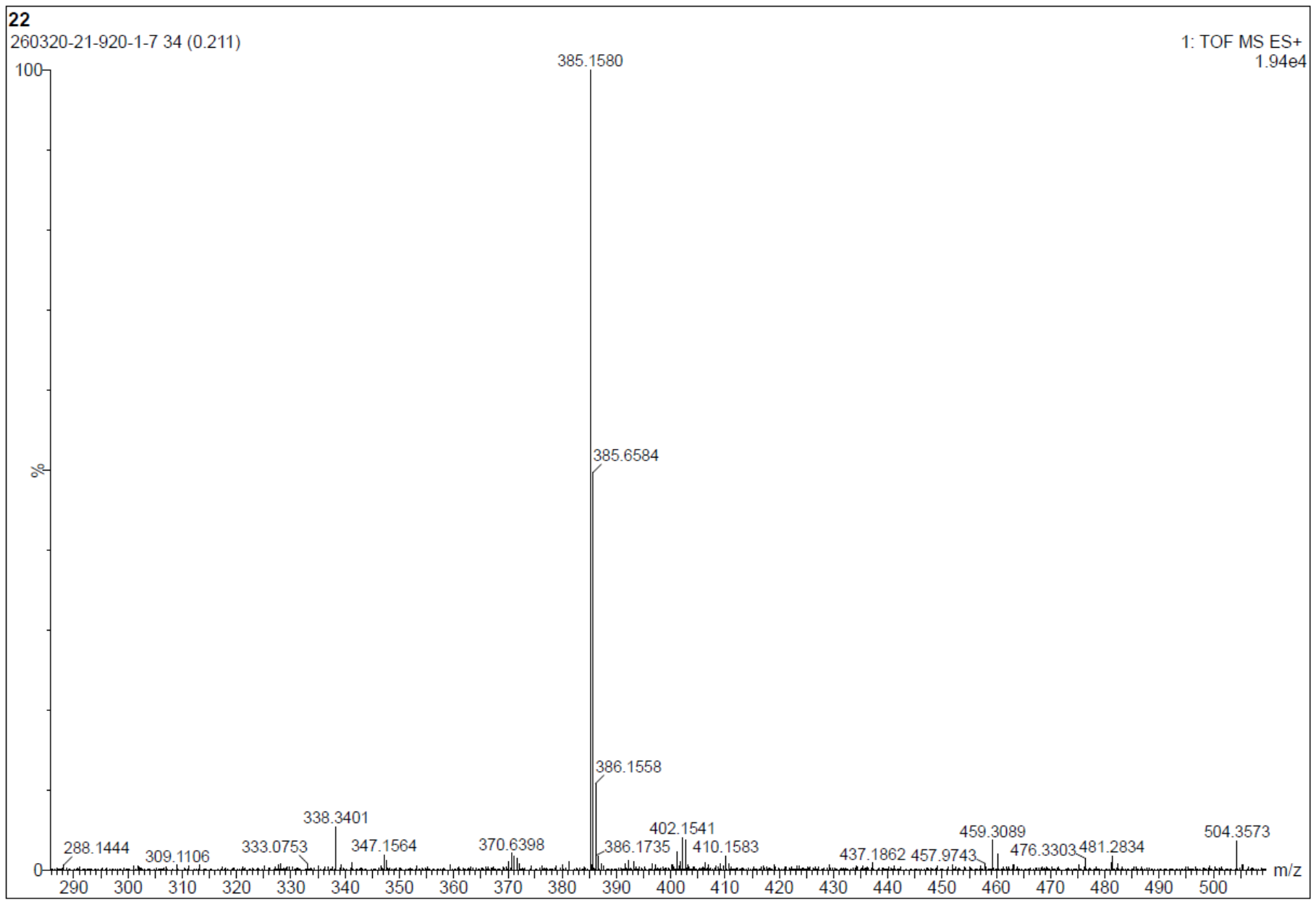

Supplementary Figure 19. HRMS spectrum of the cationic part of DMPHL-2B.

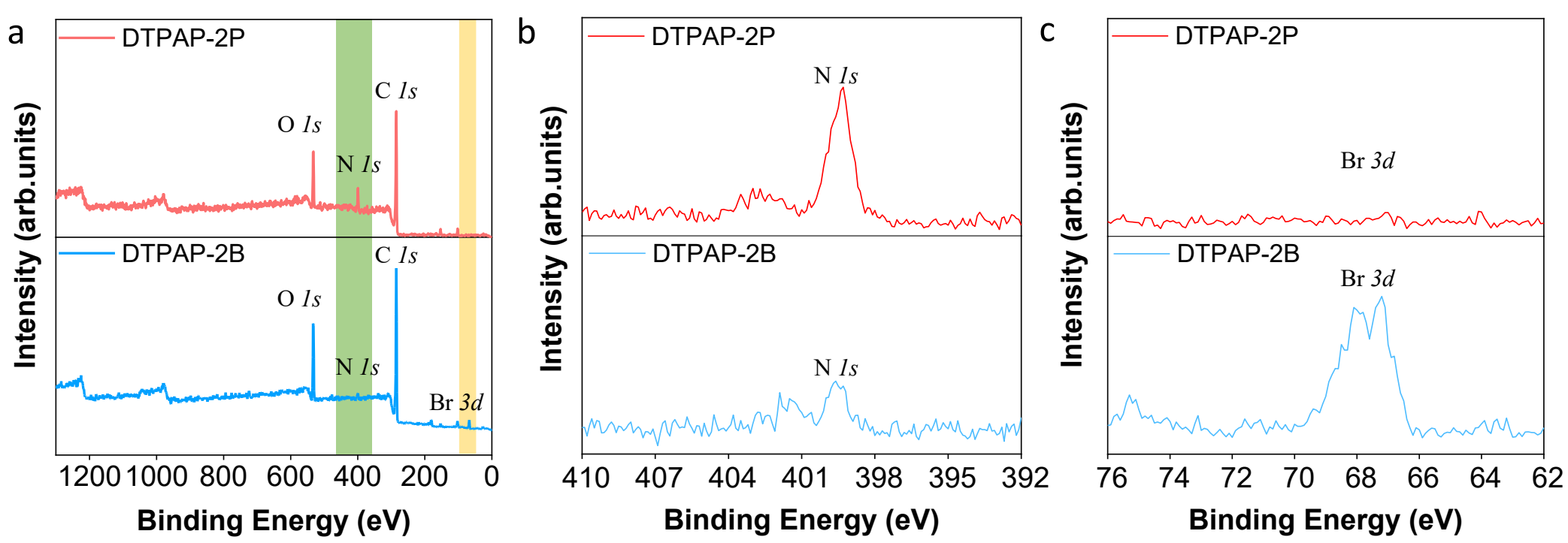

Supplementary Figure 20. The XPS results for DTPAP-2P and DTPAP-2B solids are presented as follows: (**a**) shows the general survey scans, while (**b**) and (**c**) display the enlarged regions of the spectra for the respective solid samples.

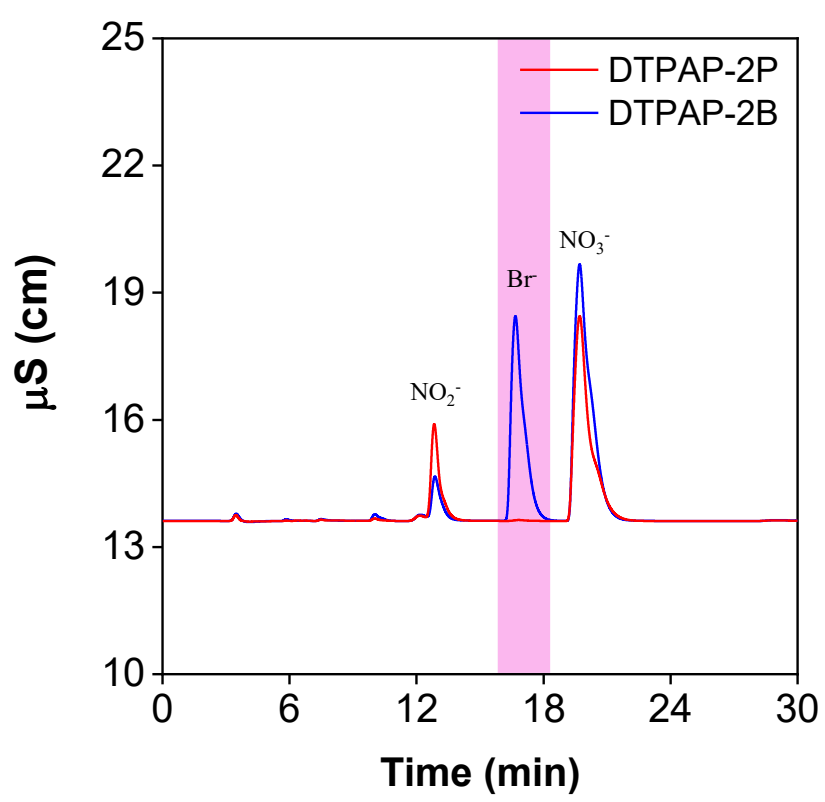

Supplementary Figure 21. The anion chromatograms of DTPAP-2P and DTPAP-2B solids.

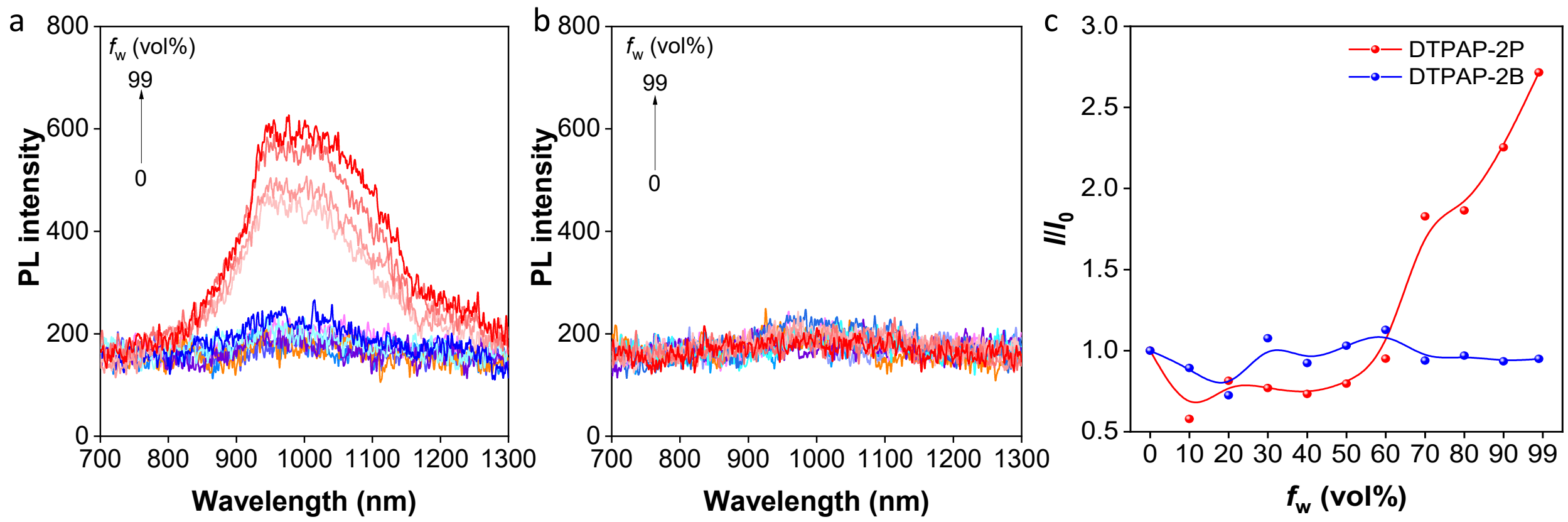

Supplementary Figure 22. PL spectra of DTPAP-2P (**a**) and DTPAP-2B (**b**) in DMSO/H_2O mixtures with different water fractions (f_w). (**c**) Plot of the relative emission intensity (I/I_0) versus f_w. λ_{ex}= 660 nm.

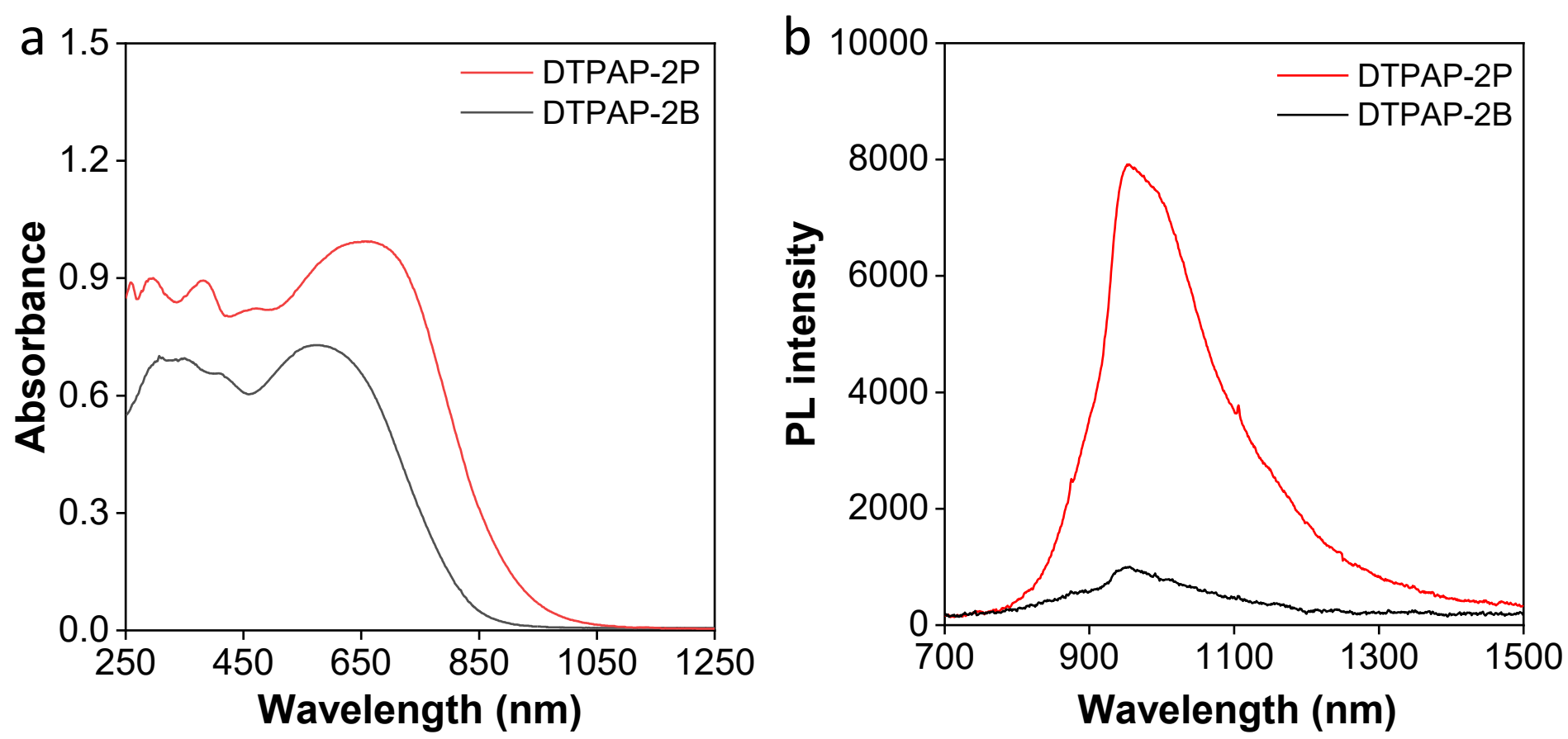

Supplementary Figure 23. (**a**) Absorption spectra and (**b**) Photoluminescence (PL) spectra of DTPAP-2P, DTPAP-2B as a raw powder. λ_{ex}=660 nm.

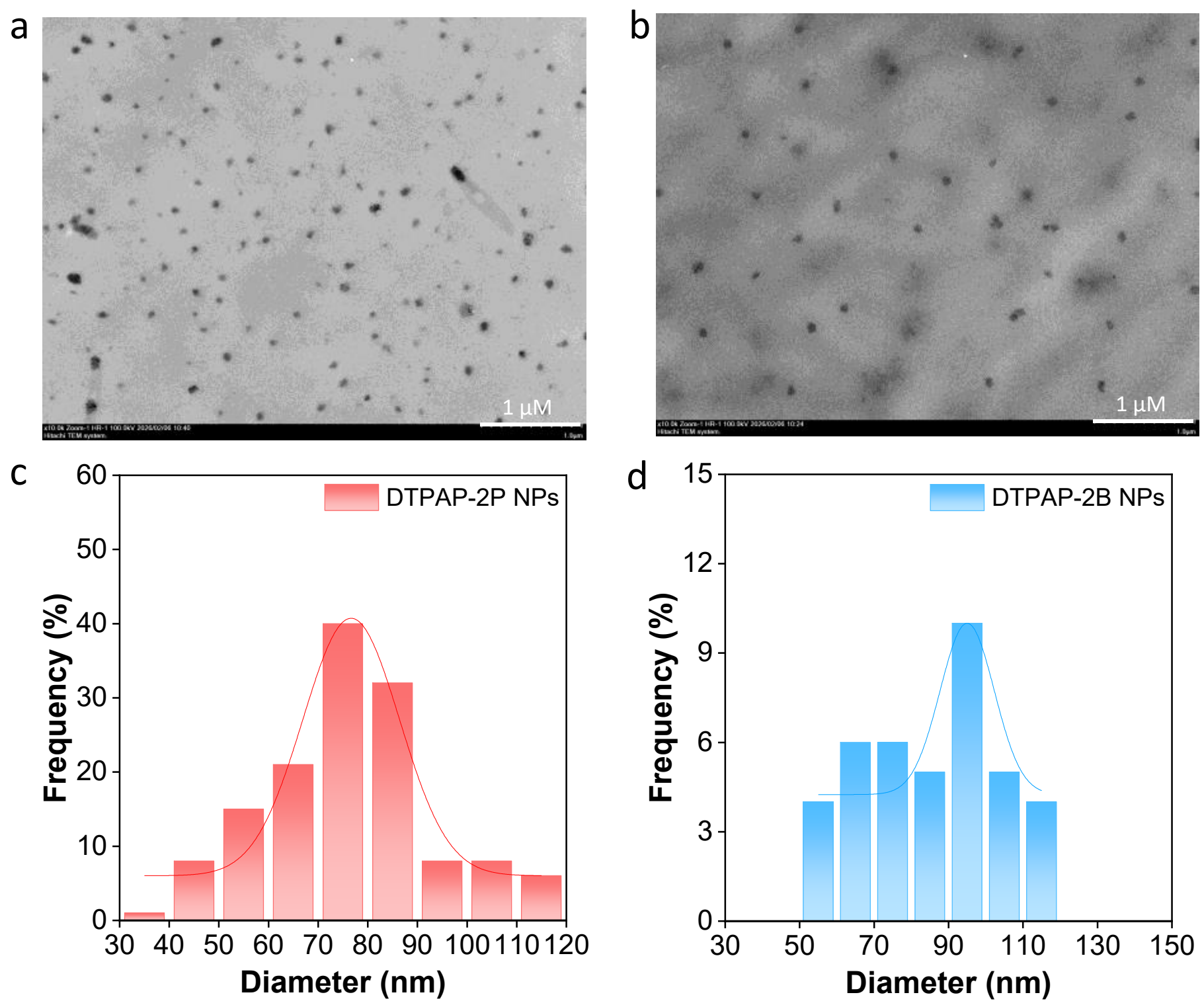

Supplementary Figure 24. TEM images of (**a**) DTPAP-2P NPs and (**b**) DTPAP-2B NPs. The size histogram for (**c**) DTPAP-2P NPs and (**d**) DTPAP-2B NPs.

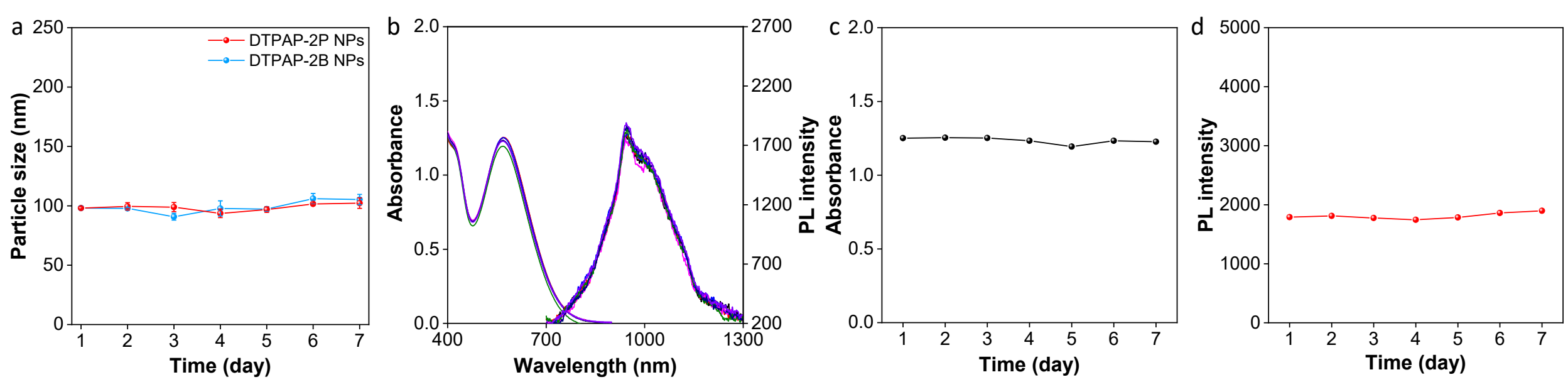

Supplementary Figure 25. (**a**) Size stability of DTPAP-2P NPs and DTPAP-2B NPs in H_2O during 7 days of storage at 4 °C. (**b**) The UV-Vis absorption and fluorescence spectra of the DTPAP-2P NPs solution during 7 days of storage at 4 °C. (**c**) Time-dependent absorbance variation of DTPAP-2P NPs at 572 nm. (**d**) Time-dependent emission intensity of DTPAP-2P NPs at 950 nm under 660 nm excitation. Data in (**a**) were presented as mean values ± SEM.

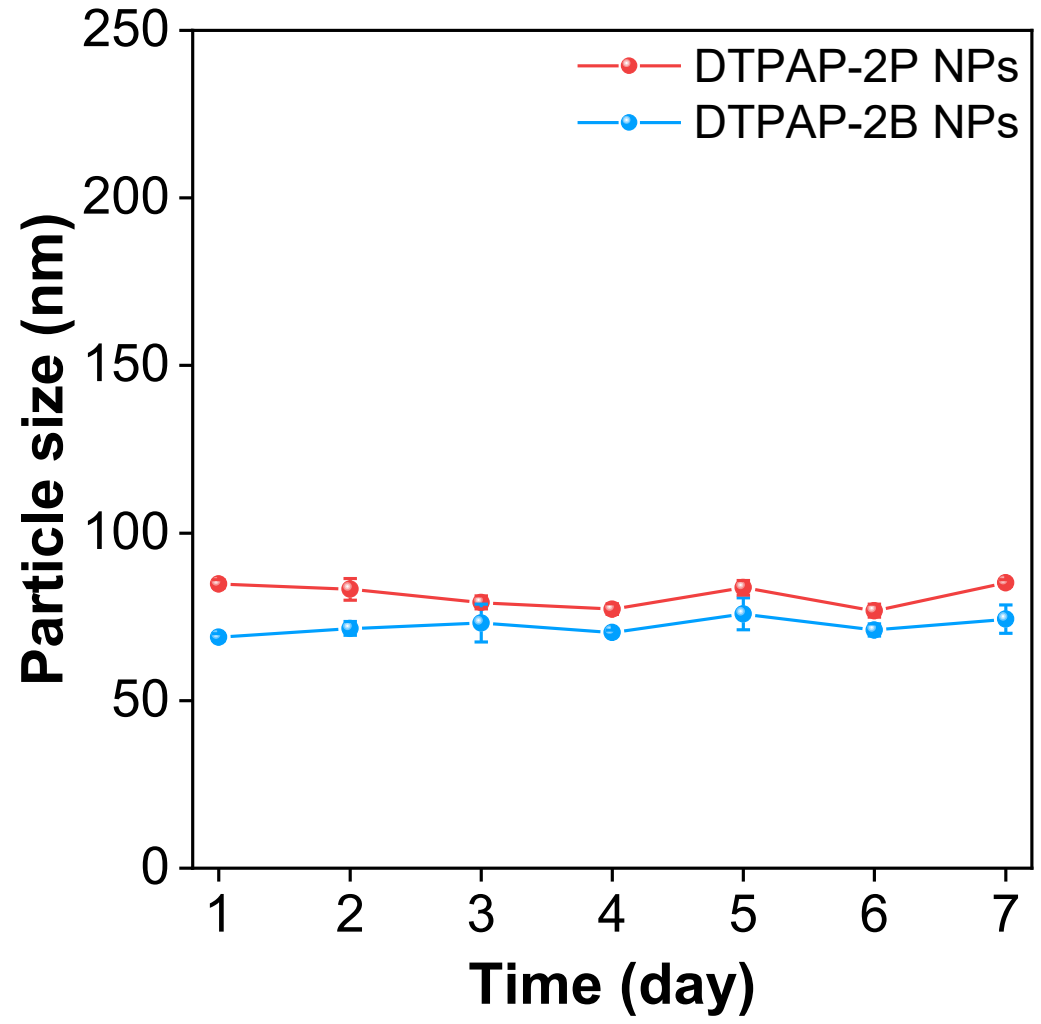

Supplementary Figure 26. Size stability of DTPAP-2P NPs and DTPAP-2B NPs in dulbecco's modified eagle medium (DMEM) during 7 days of storage at 4 °C. Data were presented as mean values ± SEM.

Supplementary Table 2. Photophysical properties and excited-state decay rate of DTPAP-2B and DTPAP-2P.

Sample	QY (%)	τ_f (10^{-9} s)	k_r (10^6 s^{-1})	k_{nr} (10^8 s^{-1})	$^1O_2$
DTPAP-2B	0.30[a]	2.48	1.21	4.02	0
DTPAP-2B NPs	0.30[a]	0.76	3.95	13.12	0.25
DTPAP-2P	0.50[a]	2.37	2.11	4.20	0
DTPAP-2P NPs	0.62[b]	1.01	6.14	9.84	0.21

[a] Absolute fluorescence quantum yield were determined using an integrating sphere. The QY of DTPAP-2P and DTPAP-2B was determined in DMSO upon excited at 540 nm. The QY of DTPAP-2B NPs was determined in aqueous solution upon excitation at 530 nm.

[b] Relative fluorescence quantum yield. The QY of DTPAP-2P NPs upon 660 nm laser irradiation was calculated using ICG (QY = 2.7%) in water as the reference.

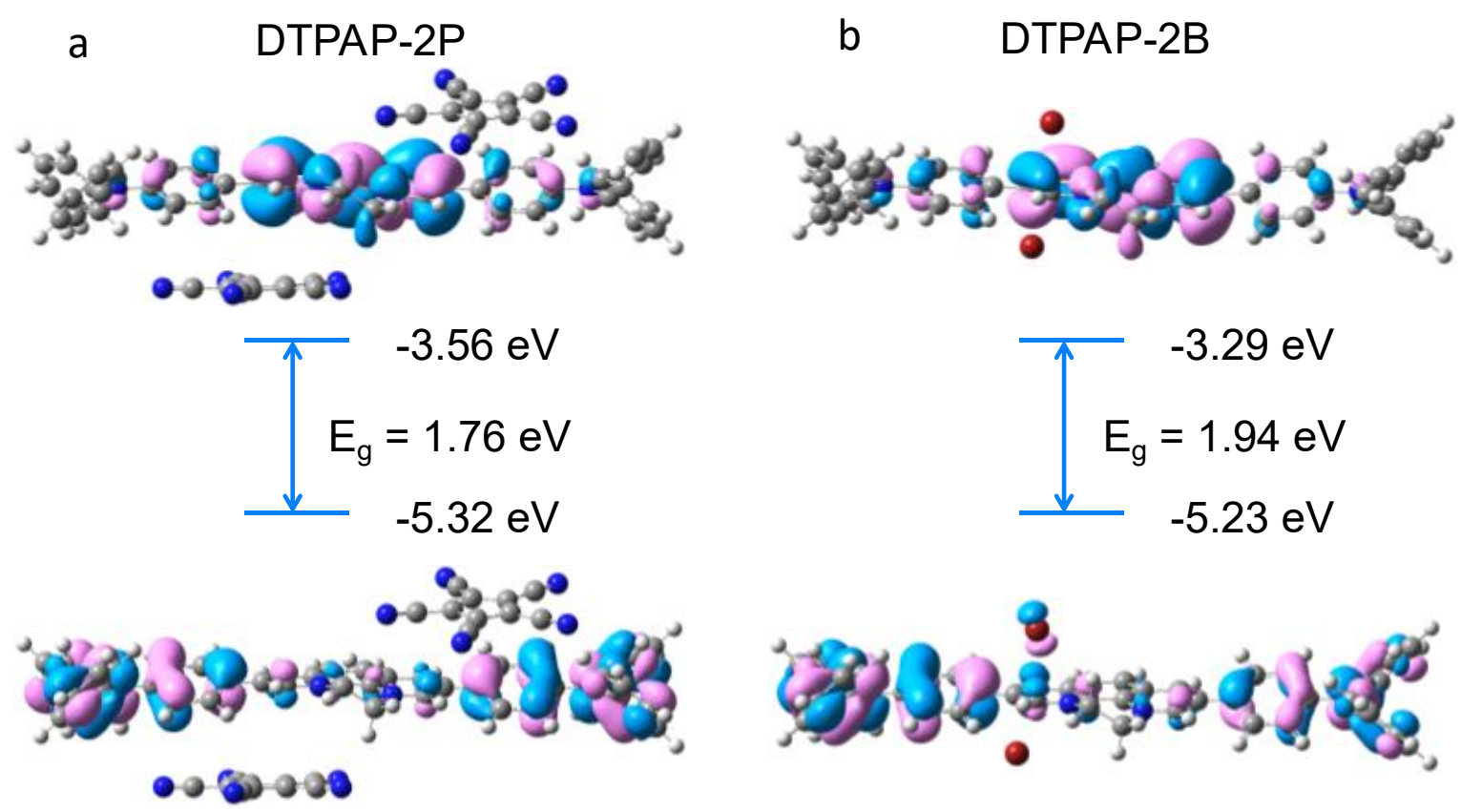

Supplementary Figure 27. The HOMO and LUMO electron cloud distributions of (**a**) DTPAP-2P and (**b**) DTPAP-2B.

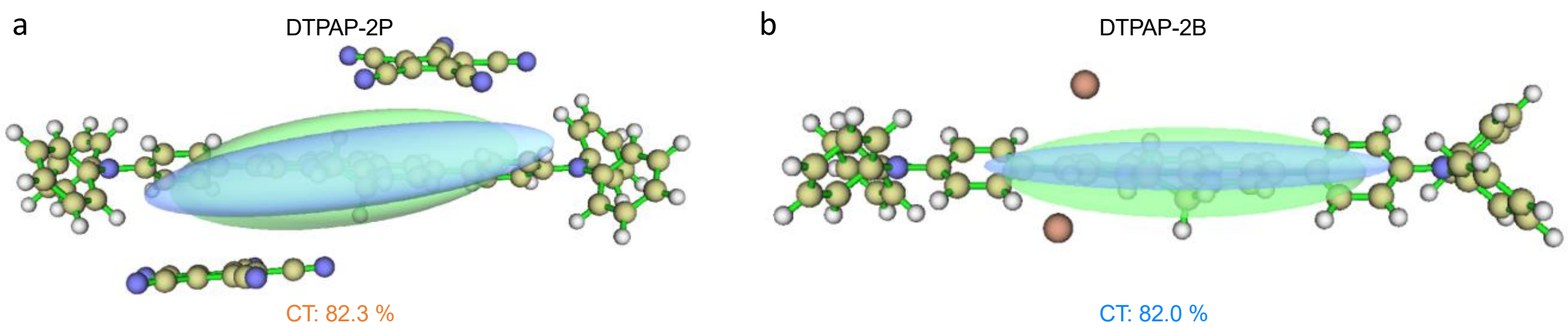

Supplementary Figure 28. C_{hole}-C_{ele} isosurface plots of DTPAP-2P (**a**) and DTPAP-2B (**b**) in the S_1 states (isovalue = 0.0003), CT represented the proportion of CT state.

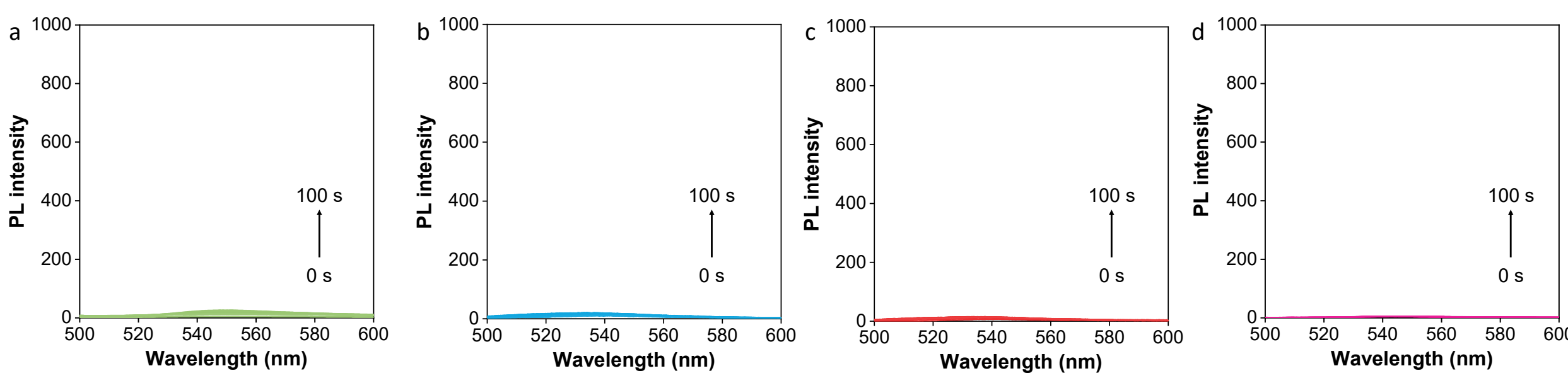

Supplementary Figure 29. (**a**) Fluorescence spectra changes of DCFH alone after 660 nm laser irradiation. Fluorescence spectra changes of DCFH induced by DTPAP-2B (**b**), DTPAP-2P (**c**) and $Na(CCN)_5$ (**d**) in DMSO solution under the 660 nm laser irradiation (0.1 W cm^{-2}). The concentration of DCFH was 1 μM and the concentration of compounds were 10 μM, respectively.

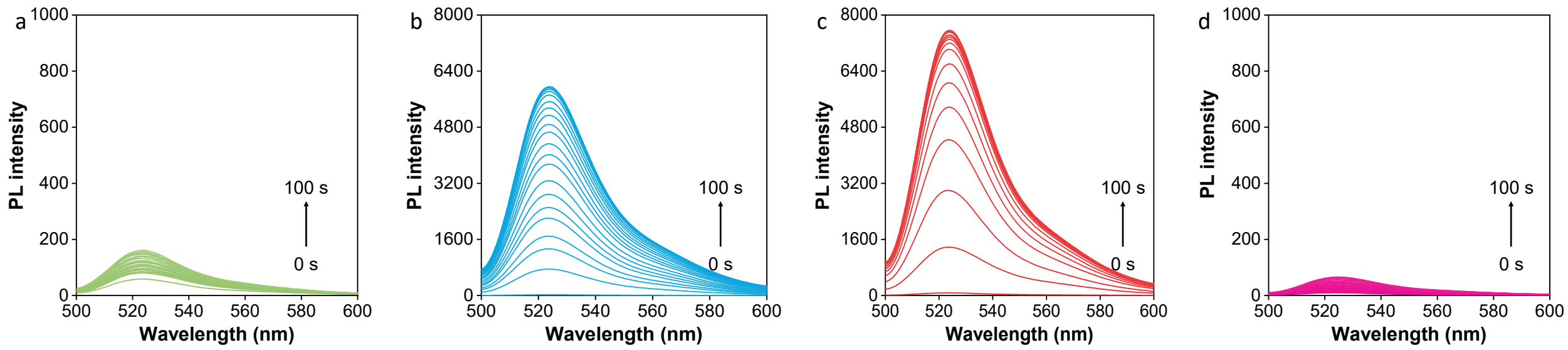

Supplementary Figure 30. (**a**) Fluorescence spectra changes of DCFH alone after 660 nm laser irradiation. Fluorescence spectra changes of DCFH induced by DTPAP-2B NPs (**b**), DTPAP-2P NPs (**c**) and Na(CCN)$_5$ NPs (**d**) in PBS (pH 7.4) under the 660 nm laser irradiation (0.1 W cm^{-2}). The concentration of DCFH was 1 μM and the concentration of NPs were 10 μM, respectively.

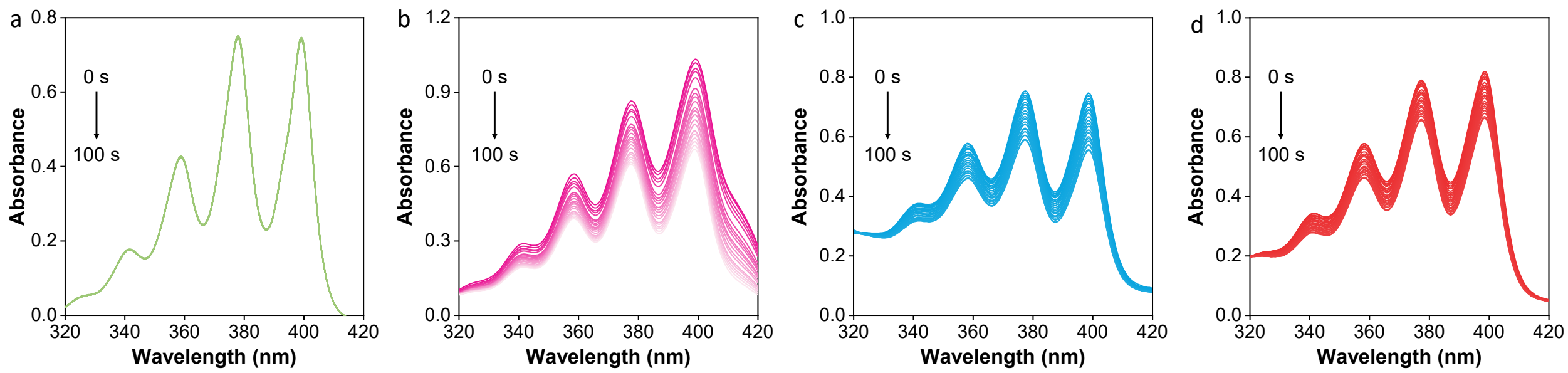

Supplementary Figure 31. (**a**) Absorption spectra changes of ABDA alone after 660 nm laser irradiation. Absorption spectra changes of ABDA induced by Ce6 (**b**), DTPAP-2B NPs (**c**) and DTPAP-2P NPs (**d**) in PBS (pH 7.4) under the 660 nm laser irradiation (0.1 W cm^{-2}). The concentration of ABDA was 50 μM and the concentration of NPs were 10 μM, respectively.

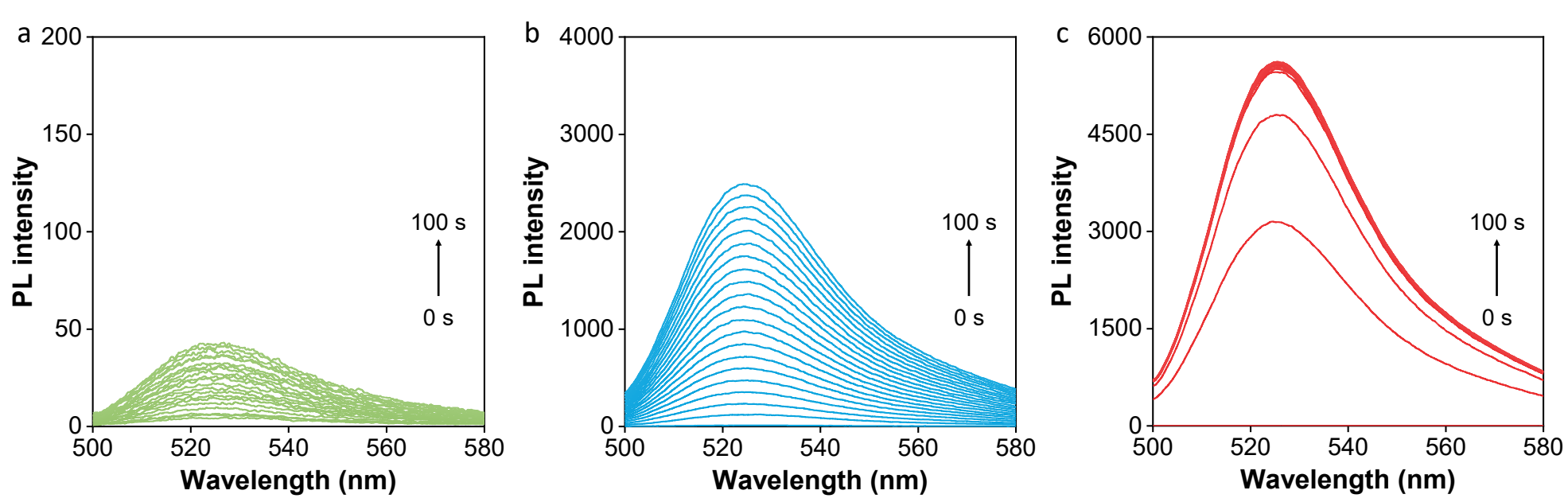

Supplementary Figure 32. (**a**) Fluorescence spectra changes of DHR123 alone after the 660 nm laser irradiation. Fluorescence spectra changes of DHR123 induced by DTPAP-2B NPs (**b**) and DTPAP-2P NPs (**c**) in PBS (pH 7.4) under the 660 nm laser irradiation (0.1 W cm^{-2}). The concentration of DHR123 was 5 μM and the concentration of NPs were 10 μM, respectively.

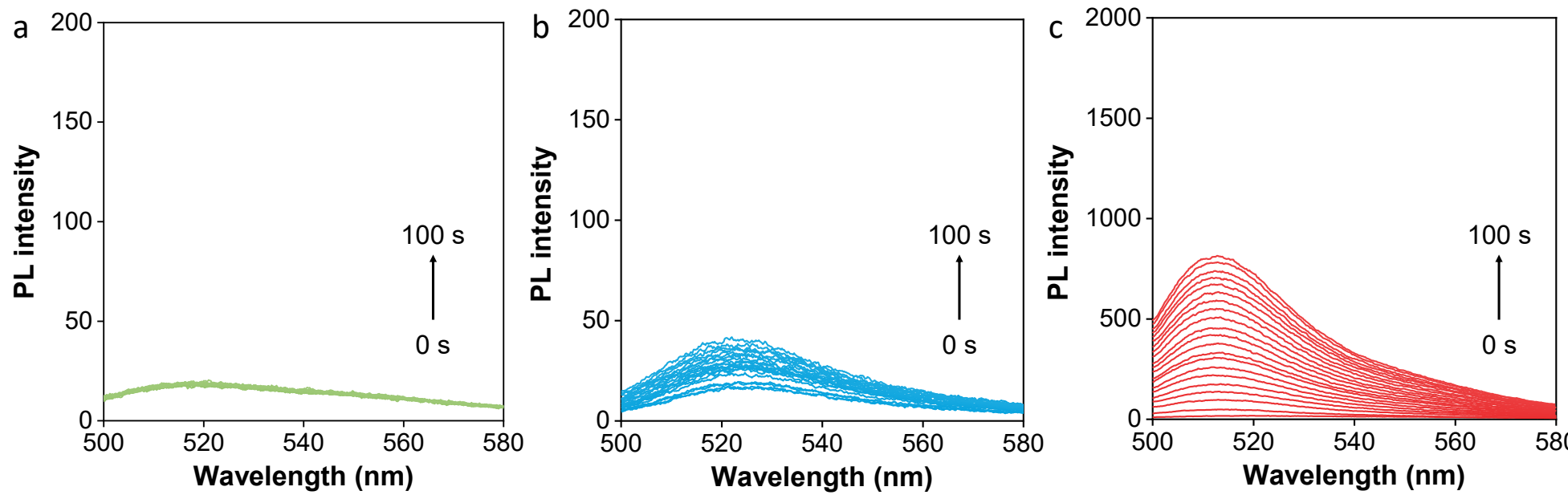

Supplementary Figure 33. (**a**) Fluorescence spectra changes of HPF alone after the 660 nm laser irradiation. Fluorescence spectra changes of HPF induced by DTPAP-2B NPs (**b**) and DTPAP-2P NPs (**c**) in PBS (pH 7.4) under the 660 nm laser irradiation (0.1 W cm^{-2}). The concentration of HPF was 5 μM and the concentration of NPs were 10 μM, respectively.

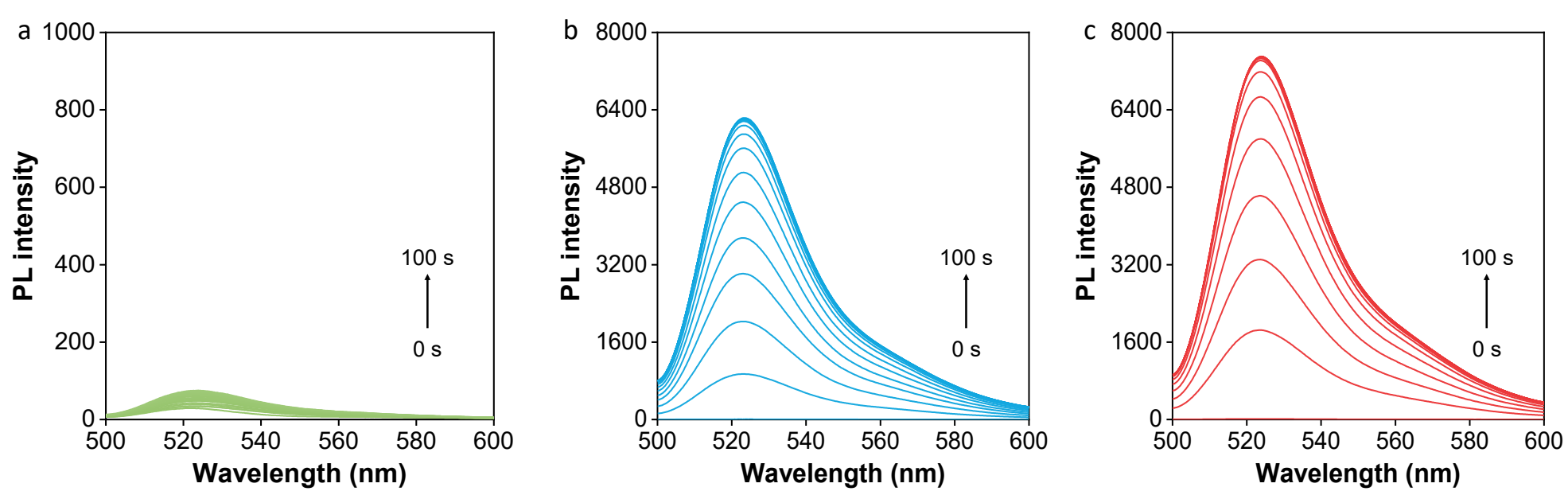

Supplementary Figure 34. (**a**) Fluorescence spectra changes of DCFH alone after white light irradiation. Fluorescence spectra changes of DCFH induced by DTPAP-2B NPs (**b**) and DTPAP-2P NPs (**c**) in PBS (pH 7.4) under the white light irradiation (10 mW cm^{-2}). The concentration of DCFH was 1 μM and the concentration of NPs were 10 μM, respectively.

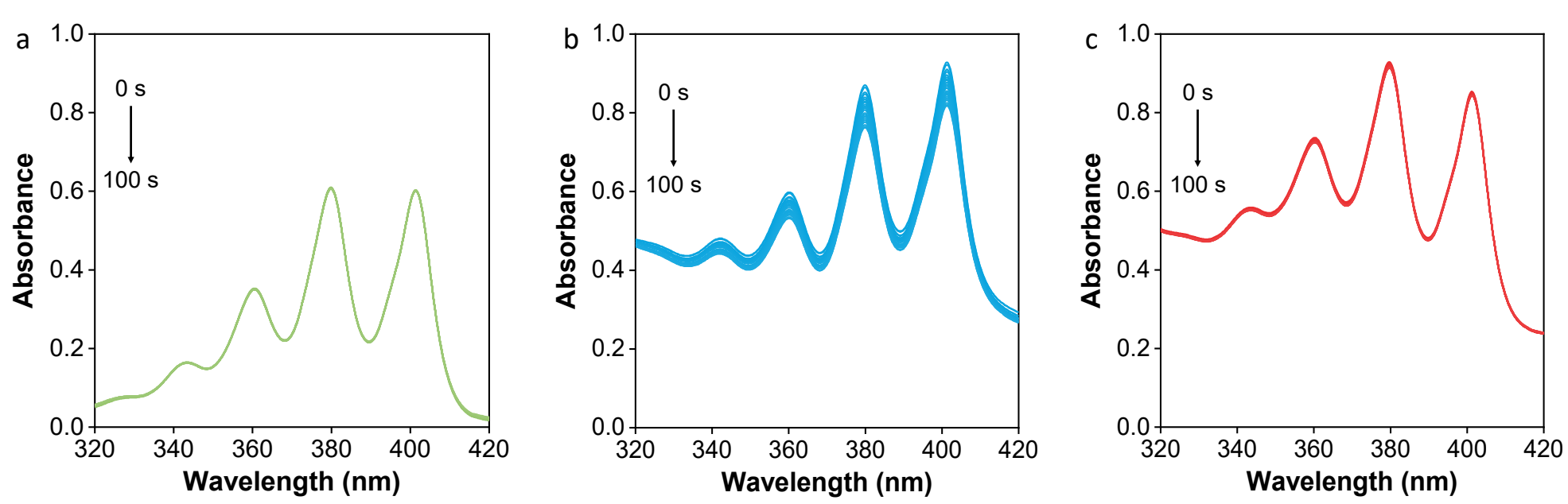

Supplementary Figure 35. (**a**) Absorption spectra changes of ABDA alone after white light irradiation. Absorption spectra changes of ABDA induced by DTPAP-2B NPs (**b**) and DTPAP-2P NPs (**c**) in PBS (pH 7.4) under white light irradiation (10 mW cm^{-2}). The concentration of ABDA was 50 μM and the concentration of NPs were 10 μM, respectively.

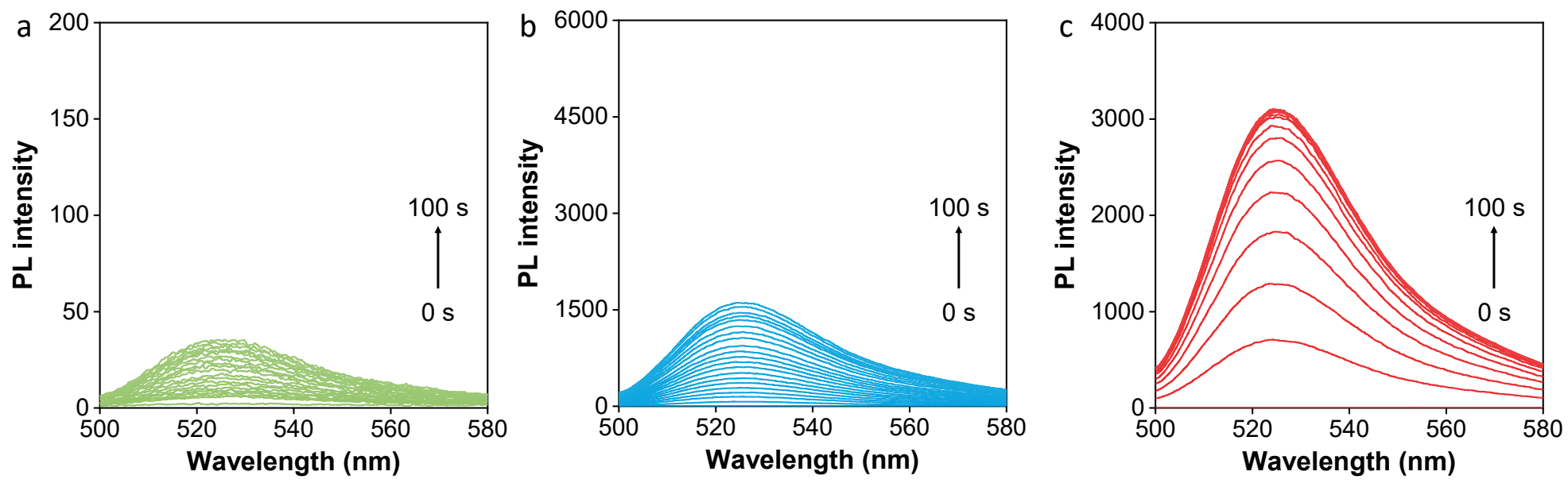

Supplementary Figure 36. (**a**) Fluorescence spectra changes of DHR123 alone after white light irradiation. Fluorescence spectra changes of DHR123 induced by DTPAP-2B NPs (**b**) and DTPAP-2P NPs (**c**) in PBS (pH 7.4) under white light irradiation (10 mW cm^{-2}). The concentration of DHR123 was 5 μM and the concentration of NPs were 10 μM, respectively.

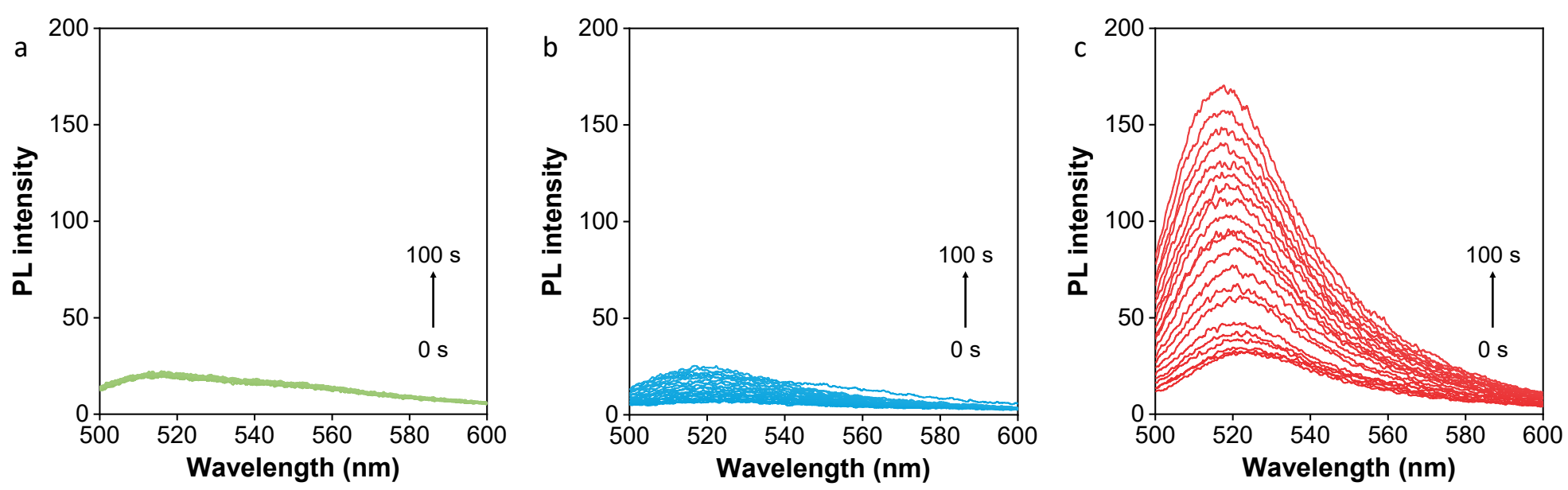

Supplementary Figure 37. (**a**) Fluorescence spectra changes of HPF alone after white light irradiation. Fluorescence spectra changes of HPF induced by DTPAP-2B NPs (**b**) and DTPAP-2P NPs (**c**) in PBS (pH 7.4) the white light irradiation (10 mW cm^{-2}). The concentration of HPF was 5 μM and the concentration of NPs were 10 μM, respectively.

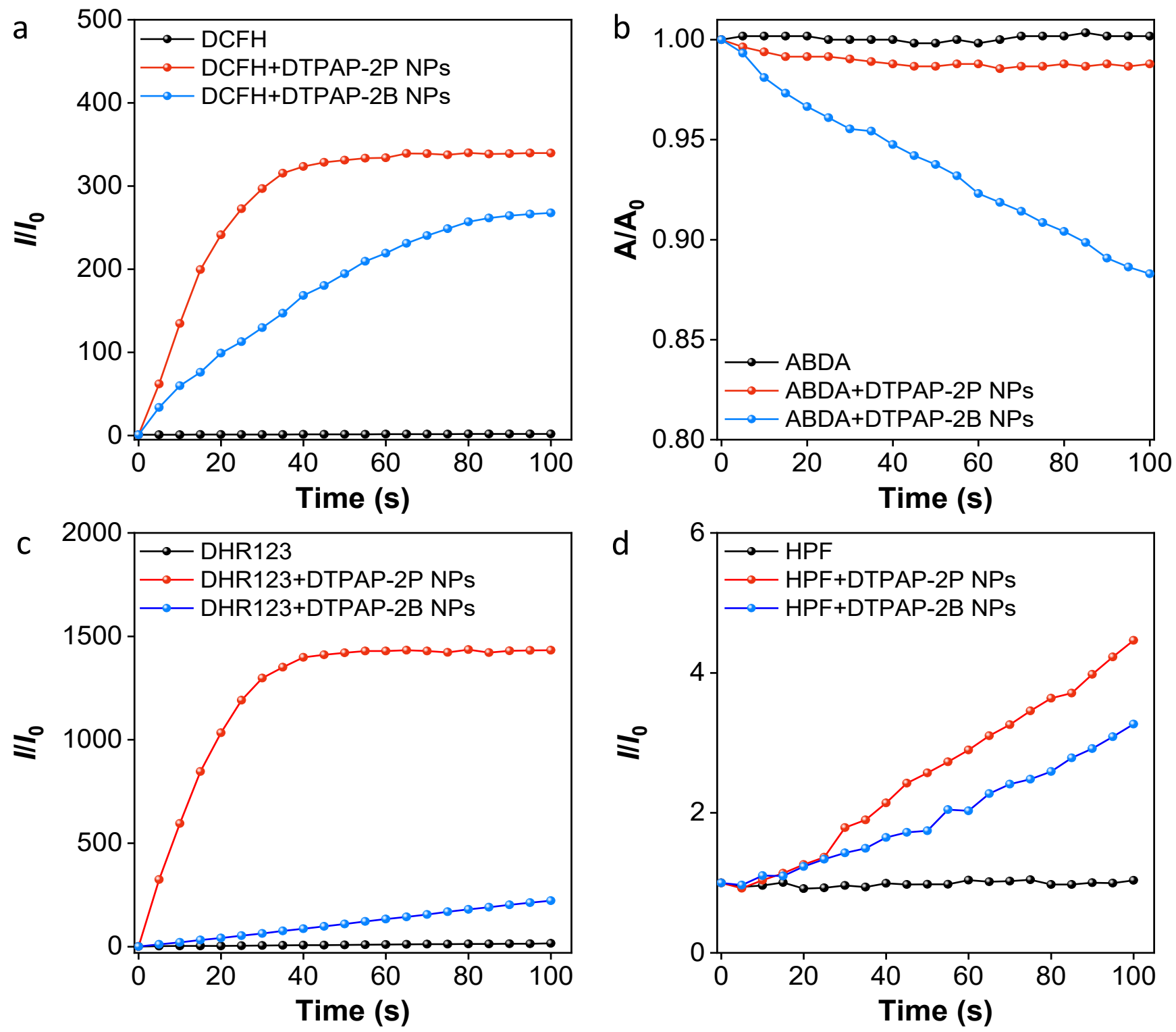

Supplementary Figure 38. ROS generation of DTPAP-2B NPs and DTPAP-2P NPs detected by (**a**) DCFH, (**b**) ABDA, (**c**) DHR123 and (**d**) HPF in PBS (pH 7.4) upon exposure to white light irradiation (10 mW cm^{-2}).

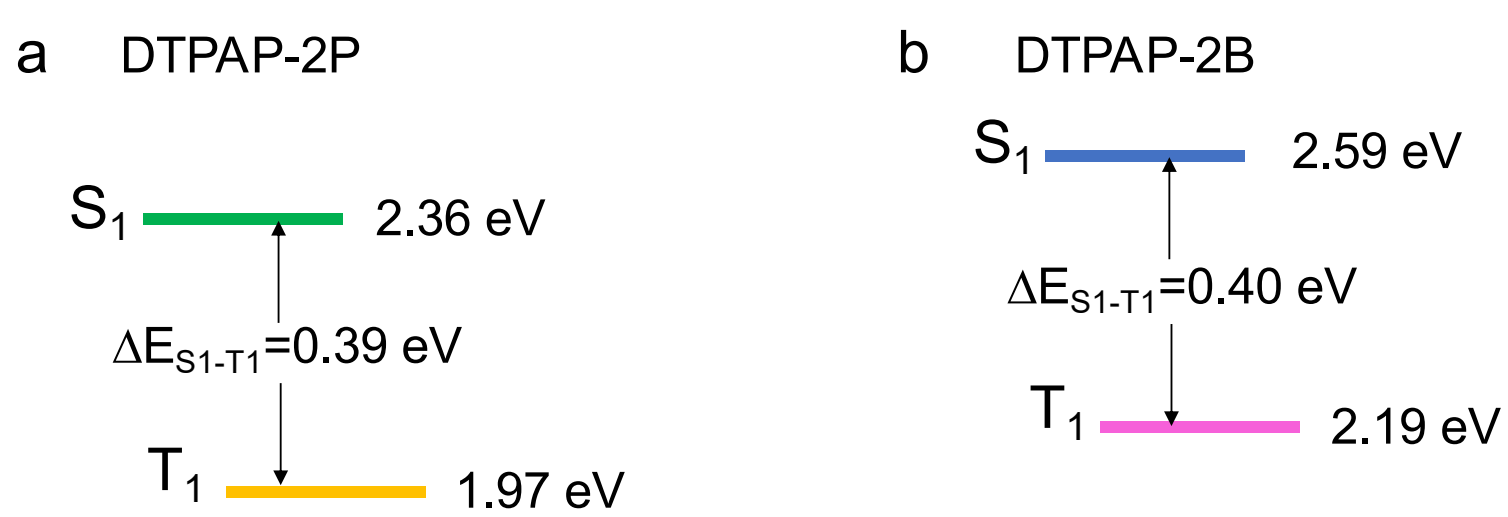

Supplementary Figure 39. The energy levels of S_1 and T_1 states calculated based on single crystals of DTPAP-2P (**a**) and DTPAP-2B (**b**).

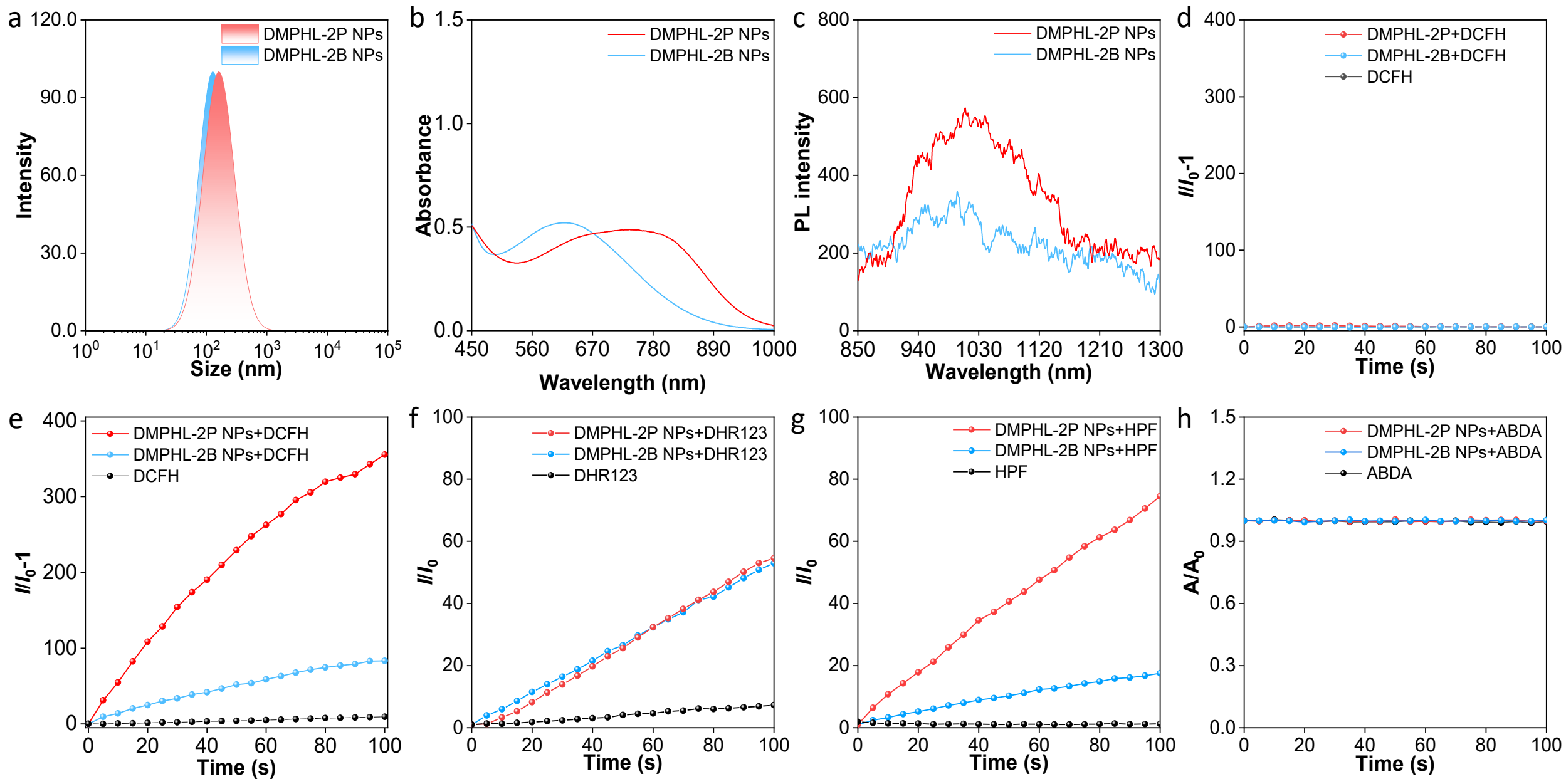

Supplementary Figure 40. (**a**) DLS of DMPHL-2P NPs and DMPHL-2B NPs in aqueous solution. (**b**) Absorption spectra and (**c**) photoluminescence (PL) spectra of DMPHL-2P NPs and DMPHL-2B NPs in PBS. λ_{ex}=808 nm. (**d**) ROS generation of DMPHL-2P and DMPHL-2B (10 μM) in DMSO detected by DCFH upon 808 nm laser irradiation (0.1 W cm^{-2}). (**e**) ROS generation of DMPHL-2P NPs and DMPHL-2B NPs (10 μM) in PBS detected by DCFH upon 808 nm laser irradiation (0.1 W cm^{-2}). Type I radical ROS generation of DMPHL-2P NPs and DMPHL-2B NPs (10 μM) in PBS detected by (**f**) DHR123 and (**g**) HPF upon 808 nm laser irradiation (0.1 W cm^{-2}). (**h**) $^1O_2$ generation of DMPHL-2P NPs and DMPHL-2B NPs in PBS detected by ABDA upon 808 nm laser irradiation (0.1 W cm^{-2}).

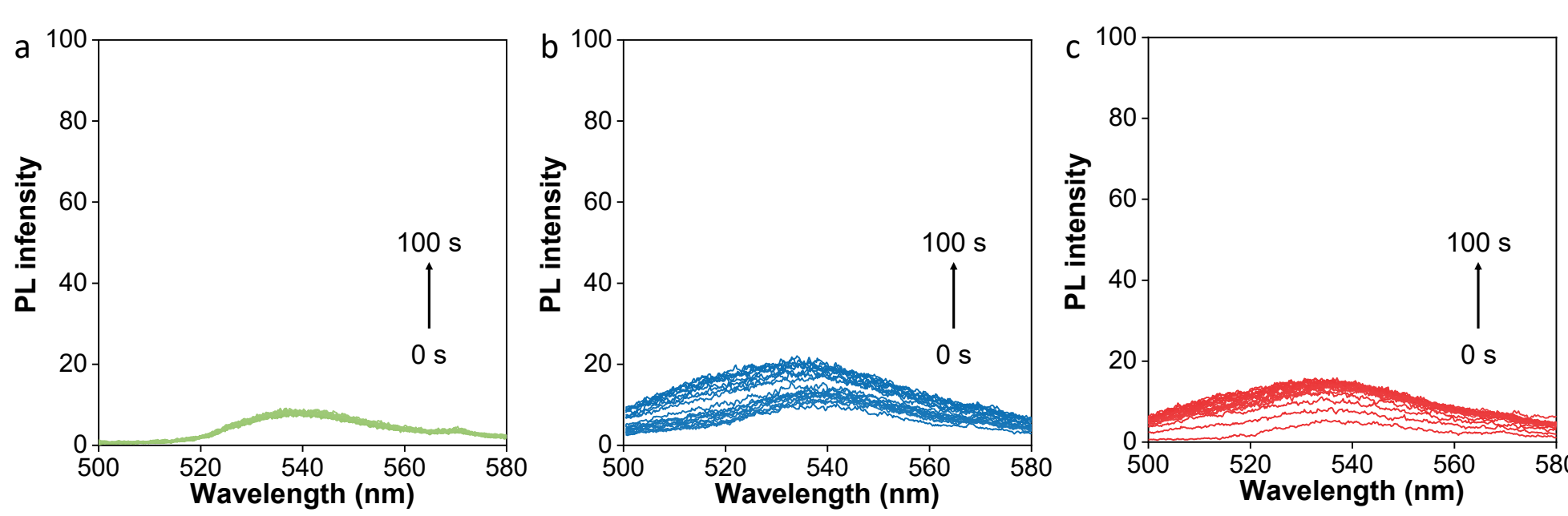

Supplementary Figure 41. Fluorescence spectra changes of DCFH alone (**a**) after 808 nm laser irradiation. Fluorescence spectra changes of DCFH induced by DMPHL-2B (**b**) and DMPHL-2P (**c**) in DMSO solution under the 808 nm laser irradiation (0.1 W cm^{-2}). The concentration of DCFH was 1 μM and the concentration of compounds were 10 μM, respectively.

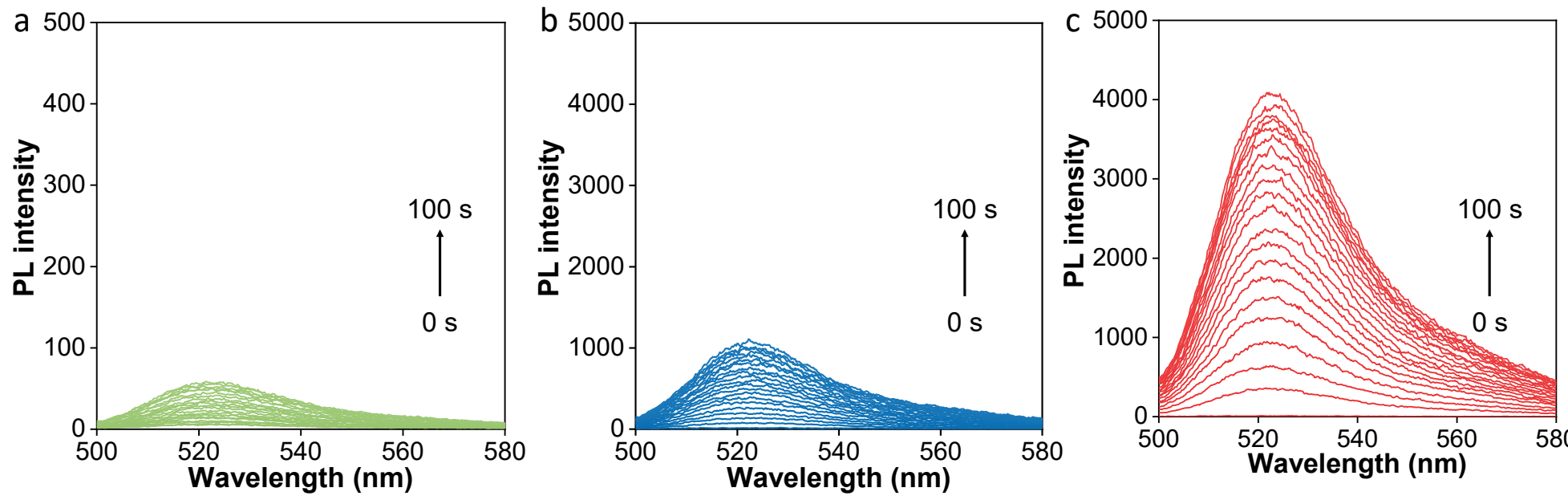

Supplementary Figure 42. Fluorescence spectra changes of DCFH alone (**a**) after 808 nm laser irradiation. Fluorescence spectra changes of DCFH induced by DMPHL-2B NPs (**b**) and DMPHL-2P NPs (**c**) in PBS (pH 7.4) under the 808 nm laser irradiation (0.1 W cm^{-2}). The concentration of DCFH was 1 μM and the concentration of NPs were 10 μM, respectively.

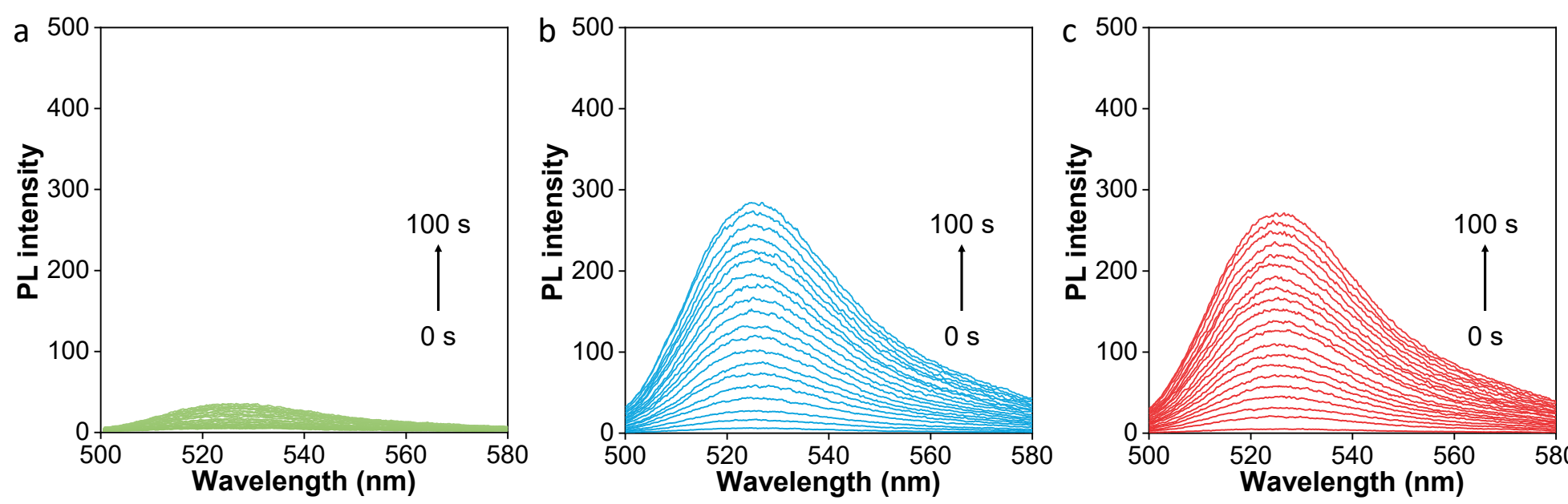

Supplementary Figure 43. Fluorescence spectra changes of DHR123 alone (**a**) after the 808 nm laser irradiation. Fluorescence spectra changes of DHR123 induced by DMPHL-2B NPs (**b**) and DMPHL-2P NPs (**c**) in PBS (pH 7.4) under the 808 nm laser irradiation (0.1 W cm^{-2}). The concentration of DHR123 was 5 μM and the concentration of NPs were 10 μM, respectively.

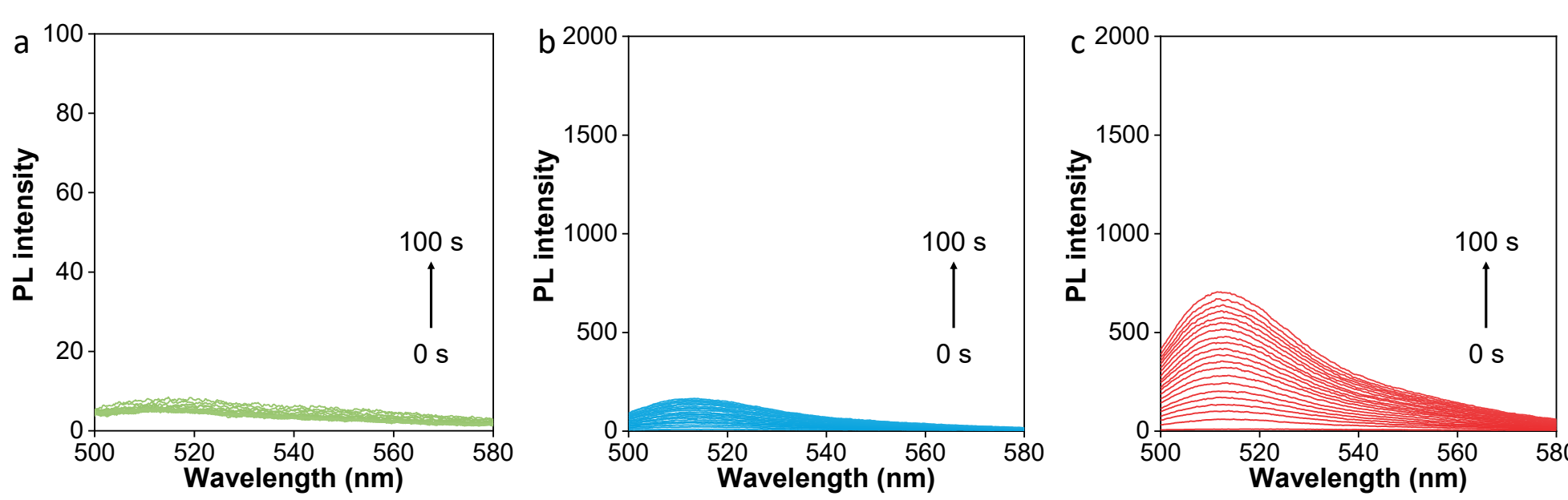

Supplementary Figure 44. Fluorescence spectra changes of HPF alone (**a**) after the 808 nm laser irradiation. Fluorescence spectra changes of HPF induced by DMPHL-2B NPs (**b**) and DMPHL-2P NPs (**c**) in PBS (pH 7.4) under the 808 nm laser irradiation (0.1 W cm^{-2}). The concentration of HPF was 5 μM and the concentration of NPs were 10 μM, respectively.

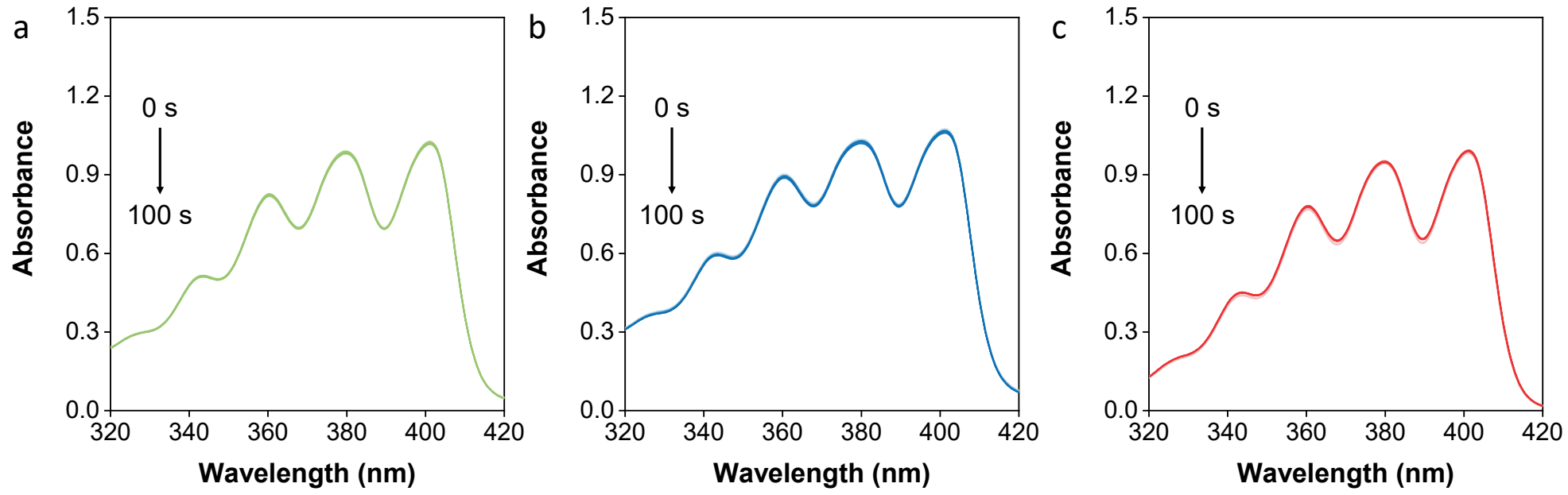

Supplementary Figure 45. Absorption spectra changes of ABDA alone (**a**) after 808 nm laser irradiation. Absorption spectra changes of ABDA induced by DMPHL-2B NPs (**b**) and DMPHL-2P NPs (**c**) in PBS (pH 7.4) under the 808 nm laser irradiation (0.1 W cm^{-2}). The concentration of ABDA was 50 μM and the concentration of NPs were 10 μM, respectively.

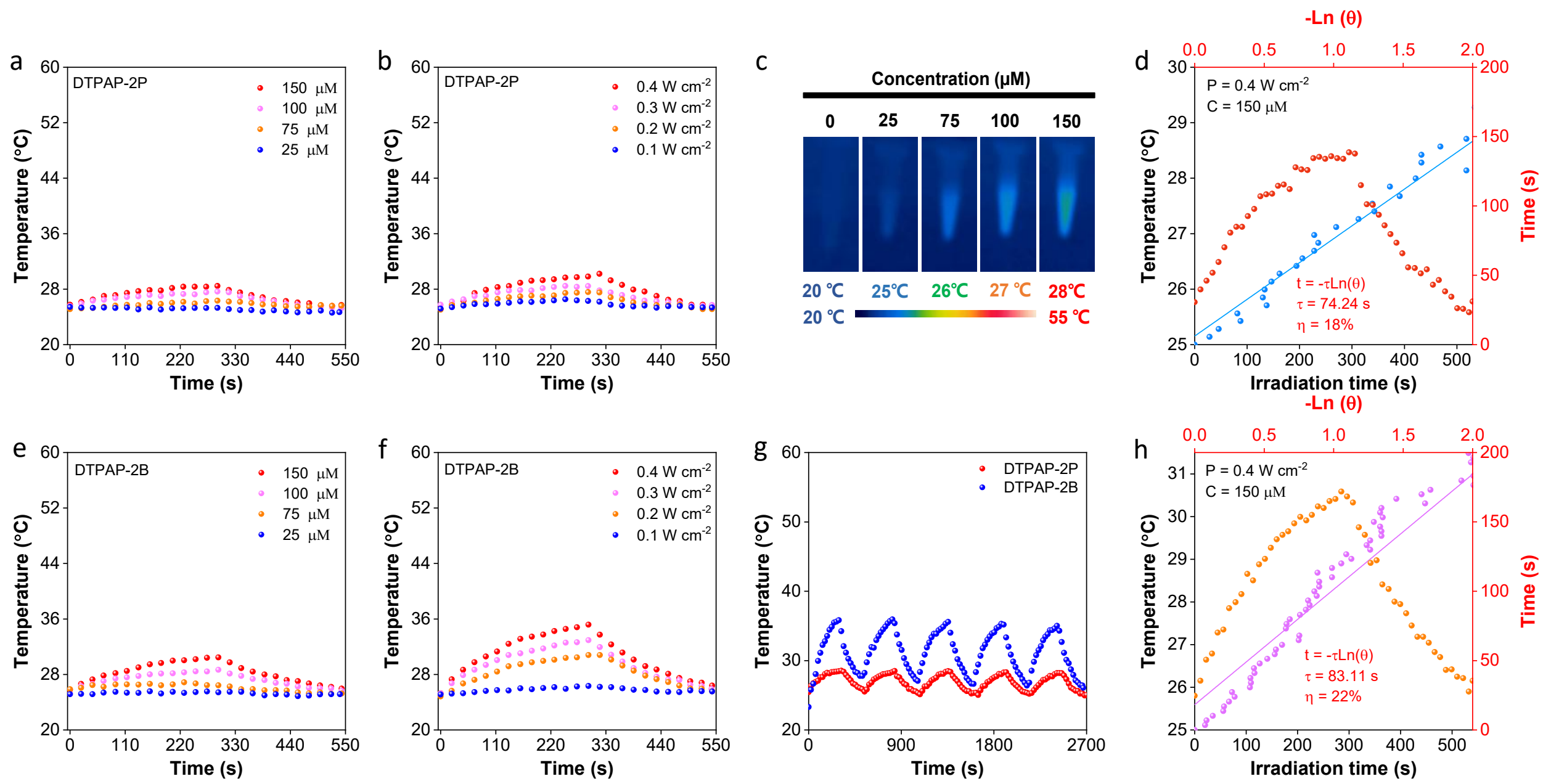

Supplementary Figure 46. The concentration-dependent (**a**) and laser power-dependent (**b**) temperature elevation curves of DTPAP-2P in DMSO solution upon 660 nm laser irradiation. (**c**) Infrared thermal images of various concentrations of DTPAP-2P under 660 nm laser irradiation (0.4 W cm^{-2}). (**d**) Temperature increasing/decreasing curve and cooling time vs -ln(θ) plot (θ: temperature driving force) of DTPAP-2P in DMSO solution. The concentration-dependent (**e**) and laser power-dependent (**f**) temperature elevation curves of DTPAP-2B in DMSO solution upon 660 nm laser irradiation. (**g**) Photothermal stability of DTPAP-2P and DTPAP-2B in DMSO solution during 5 cycles of heating-cooling. (**h**) Temperature increasing/decreasing curve and cooling time vs -ln(θ) plot of DTPAP-2B in DMSO solution.

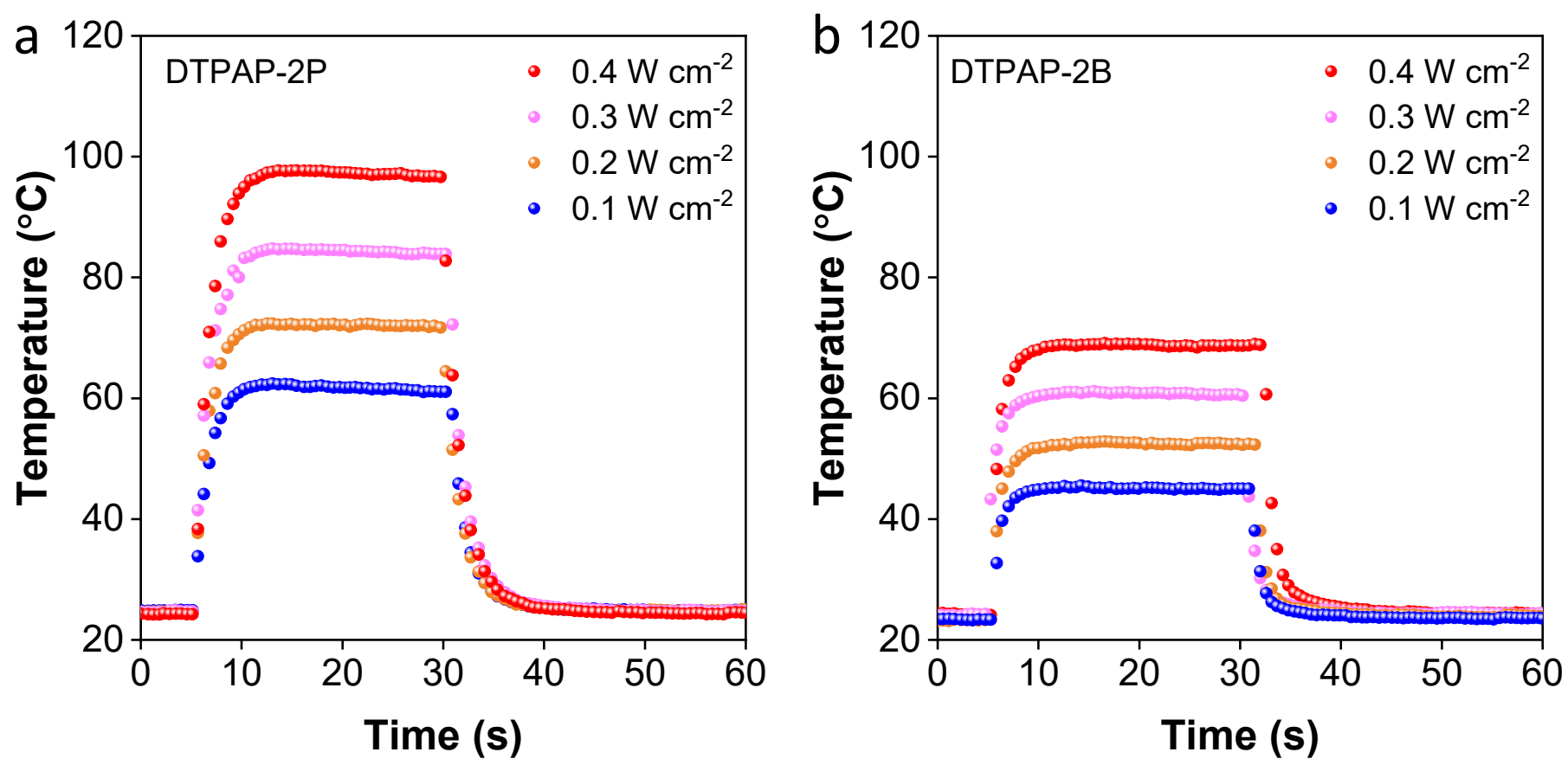

Supplementary Figure 47. The laser power-dependent temperature elevation curves of DTPAP-2P (**a**) and DTPAP-2B (**b**) as a raw powder upon 660 nm laser irradiation.

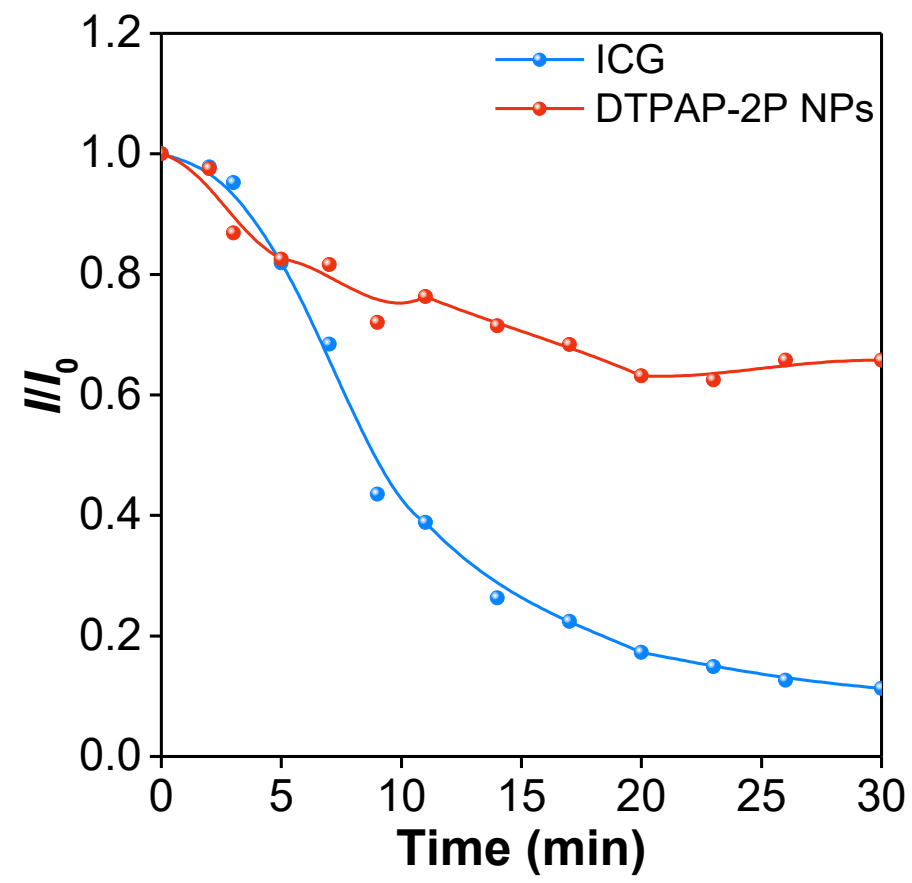

Supplementary Figure 48. Photostability of DTPAP-2P NPs and ICG in PBS under 660 nm laser irradiation (0.4 W cm^{-2}).

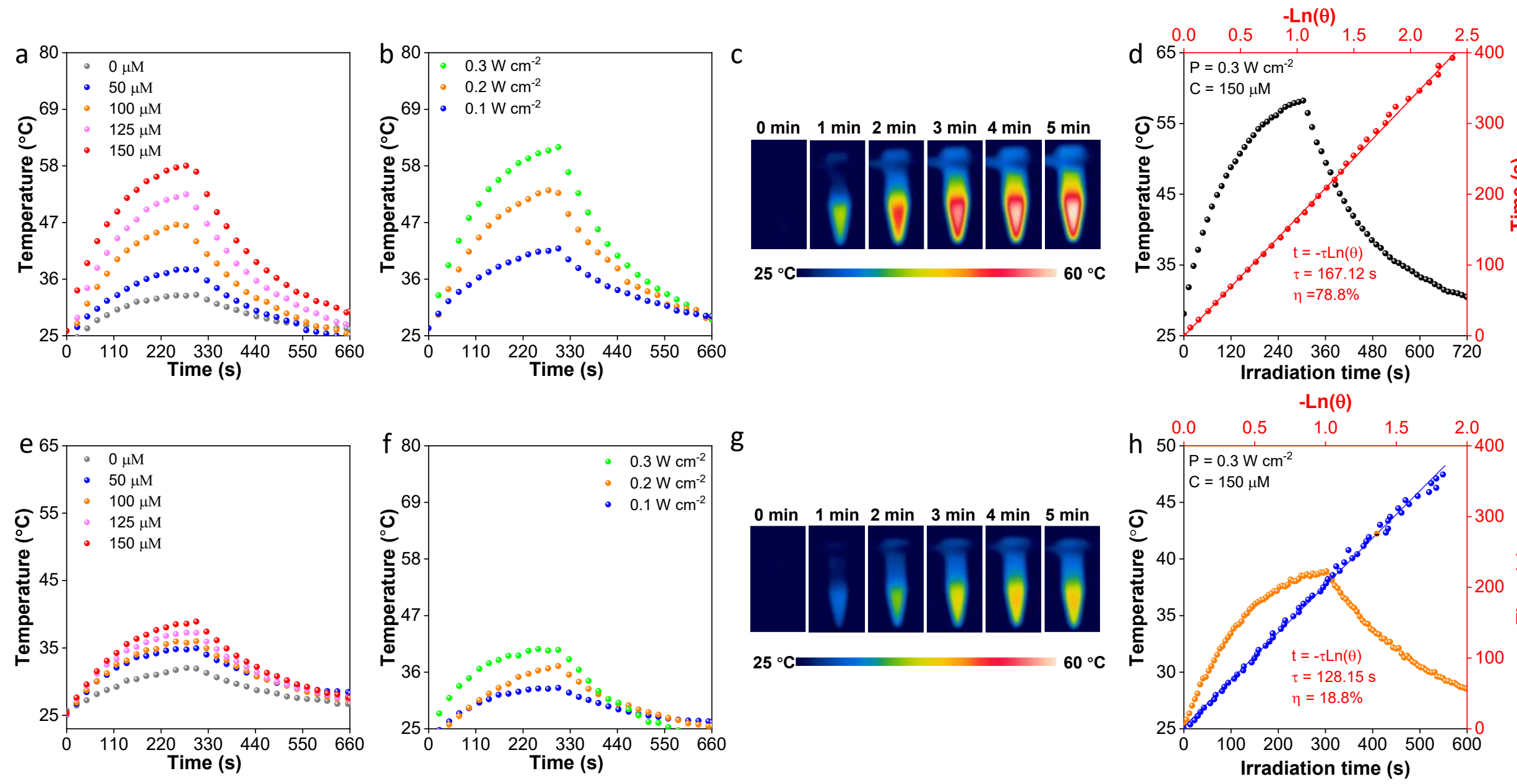

Supplementary Figure 49. The concentration-dependent (**a**) and laser power-dependent (**b**) temperature elevation curves of DMPHL-2P NPs upon 808 nm laser irradiation. (**c**) Infrared thermal images of various times of DMPHL-2P NPs under 808 nm laser irradiation (0.3 W cm^{-2}). (**d**) Temperature increasing/decreasing curve and cooling time vs -ln(θ) plot (θ: temperature driving force) of DMPHL-2P NPs. The concentration-dependent (**e**) and laser power-dependent (**f**) temperature elevation curves of DMPHL-2B NPs upon 808 nm laser irradiation. (**g**) Infrared thermal images of various times of DMPHL-2B NPs under 808 nm laser irradiation (0.3 W cm^{-2}). (**h**) Temperature increasing/decreasing curve and cooling time vs -ln(θ) plot of DMPHL-2B NPs.

Supplementary Table 3. Crystallographic and structural refinement data.

Name	DTPAP-2B[a]	DTPAP-2P[b]
Empirical formula	$C_{50}H_{38}Br_2N_4$	$C_{70}H_{38}N_{14}$
Formula weight	886.70	1075.14
Temperature (K)	219.98(10)	103(5)
Crystal system	monoclinic	orthorhombic
Crystal size/mm^3	0.14 × 0.11 × 0.09	0.16 × 0.13 × 0.12
Space group	$P2_1/n$	$Pca2_1$
a (Å)	11.0375(6)	15.4701(10)
b (Å)	8.4725(5)	27.7287(8)
c (Å)	48.750(4)	12.7508(6)
α (º)	90	90
β (º)	90.538(7)	90
γ (º)	90	90
Volume (Å^3)	4558.6(5)	5469.7(5)
Z	4	4
2θ range for data collection (°)	7.254 to 147.308	8.564 to 146.822
Index ranges	$-12 \le h \le 13$, $-7 \le k \le 10$, $-40 \le l \le 60$	$-18 \le h \le 17$, $-34 \le k \le 32$, $-15 \le l \le 15$
Reflections collected	20195	42037
Final R indexes [I>=2σ (I)]	R_1 = 0.0609, wR_2 = 0.1456	R_1 = 0.0865, wR_2 = 0.2288
Final R indexes [all data]	R_1 = 0.0839, wR_2 = 0.1614	R_1 = 0.1104, wR_2 = 0.2482
Independent reflections	8940 [R_{int} = 0.0526, R_{sigma} = 0.0701]	10040 [R_{int} = 0.0477, R_{sigma} = 0.0347]
Data/restraints/parameters	8940/0/525	10040/9800/912
Goodness-of-fit on F^2	1.026	1.053

[a]CCDC: 2387016. [b]CCDC: 2466906. These data can be obtained free of charge via the joint Cambridge Crystallographic Data Centre (CCDC) and Fachinformationszentrum Karlsruhe Access Structures service.

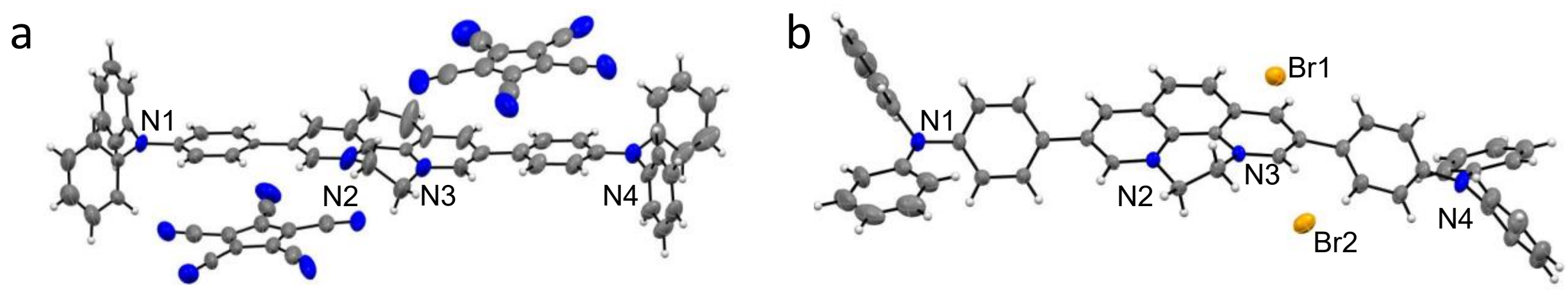

Supplementary Figure 50. Representative diagram for crystal structure of DTPAP-2P (**a**) and DTPAP-2B (**b**) with thermal ellipsoids set at 50% probability level. CCDC: 2466906 for DTPAP-2P and 2387016 for DTPAP-2B.

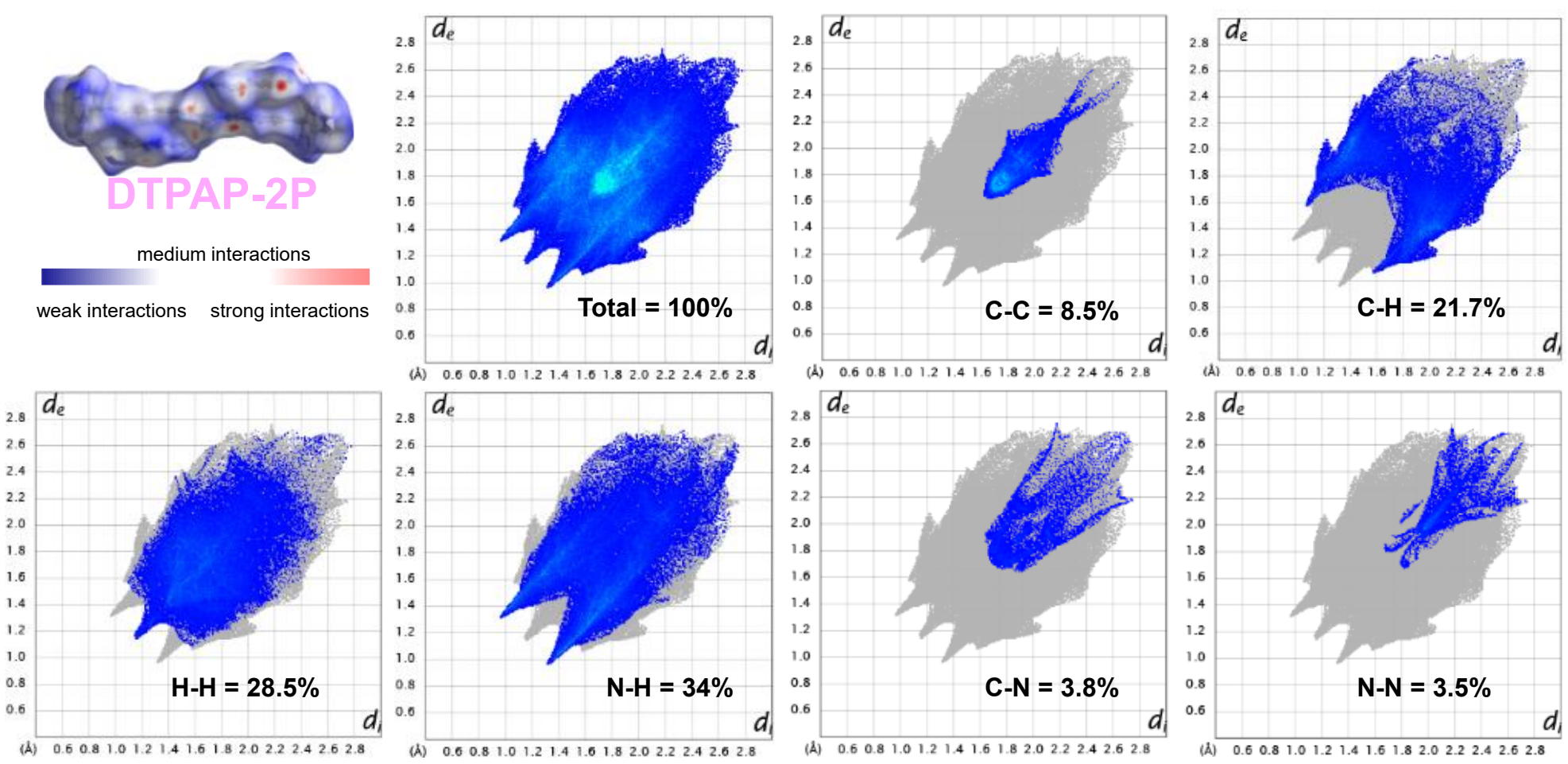

Supplementary Figure 51. Hirshfeld surface analysis and fingerprint plot of DTPAP-2P.

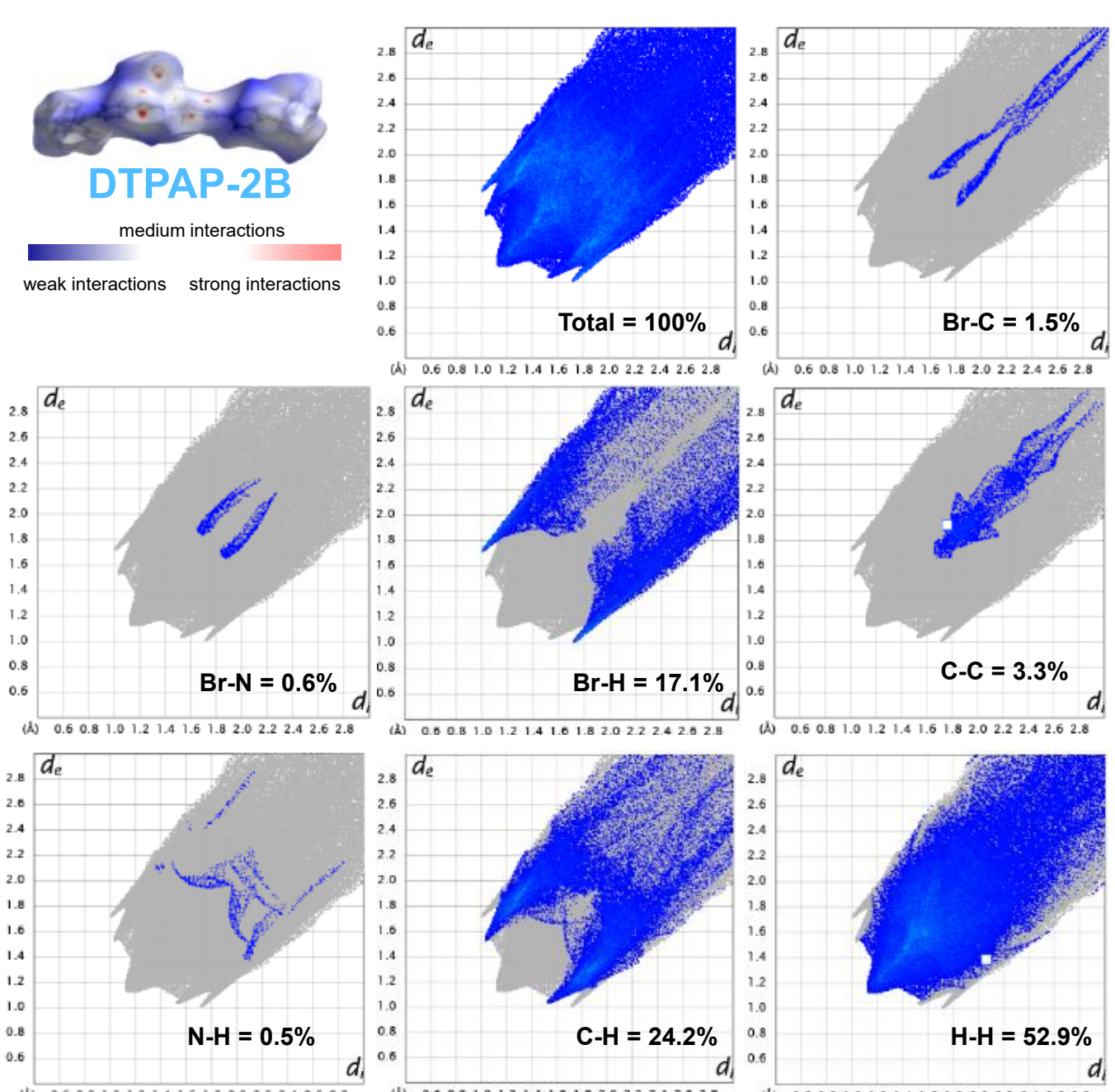

Supplementary Figure 52. Hirshfeld surface analysis and fingerprint plot of DTPAP-2B.

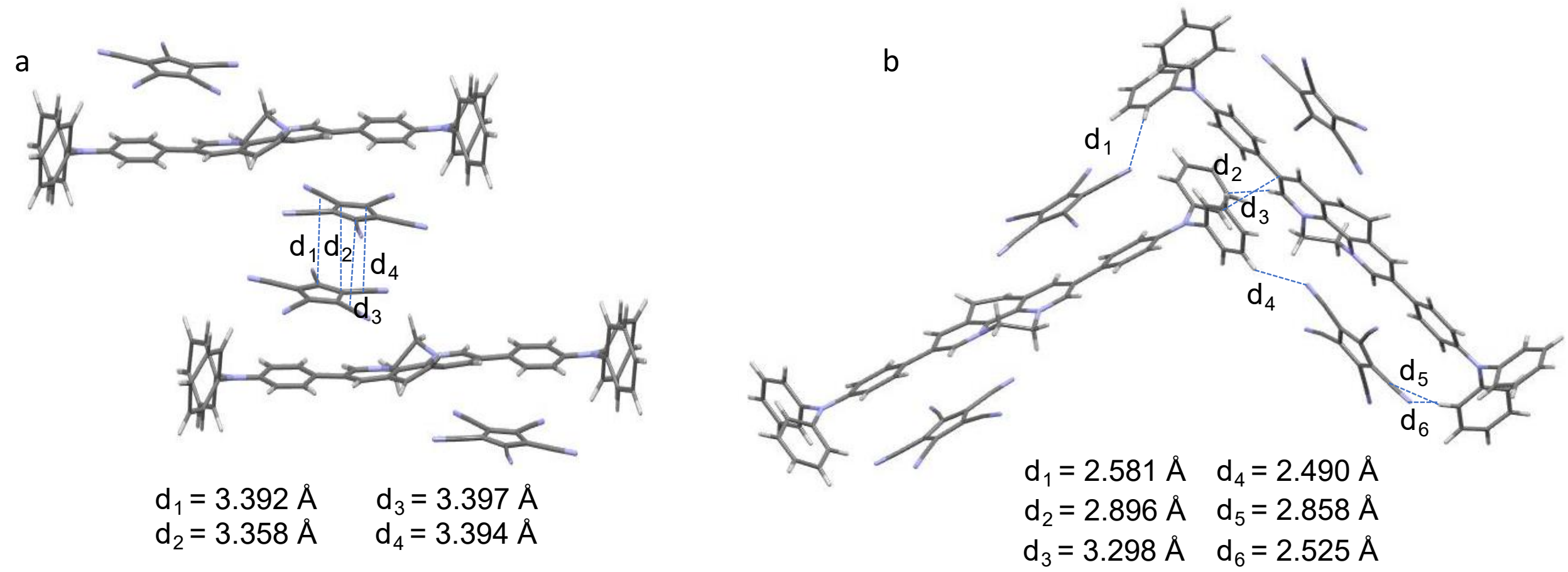

Supplementary Figure 53. Interlayer dimer (**a**) and monolayer (**b**) intermolecular interactions of DTPAP-2P.

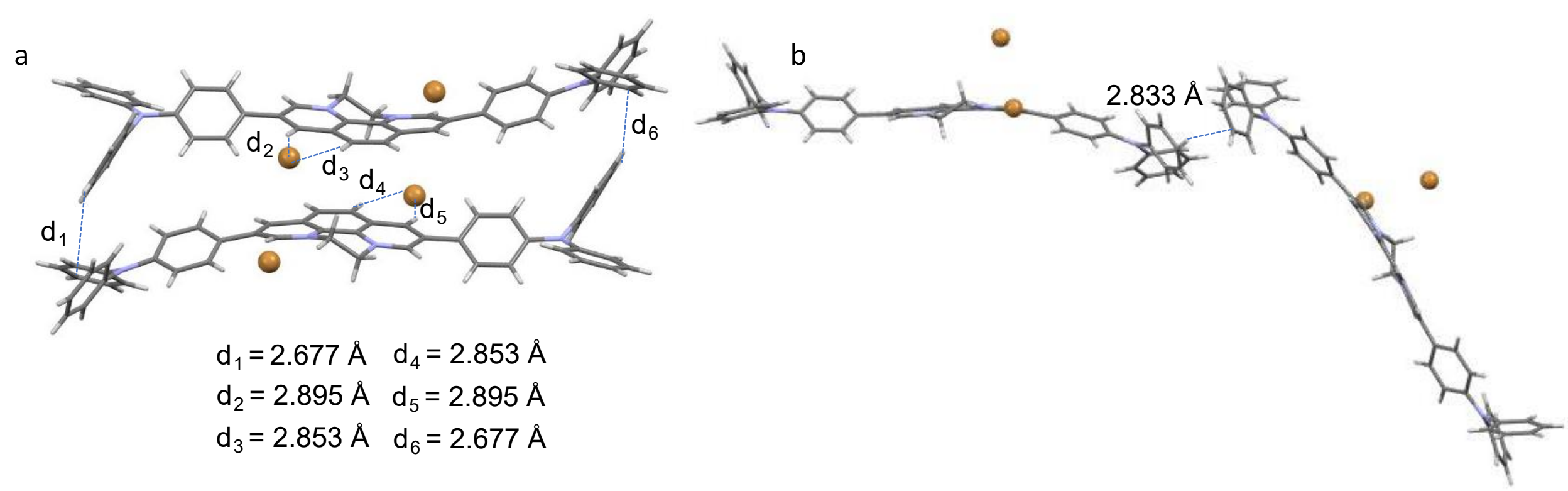

Supplementary Figure 54. Interlayer dimer (**a**) and monolayer (**b**) intermolecular interactions of DTPAP-2B.

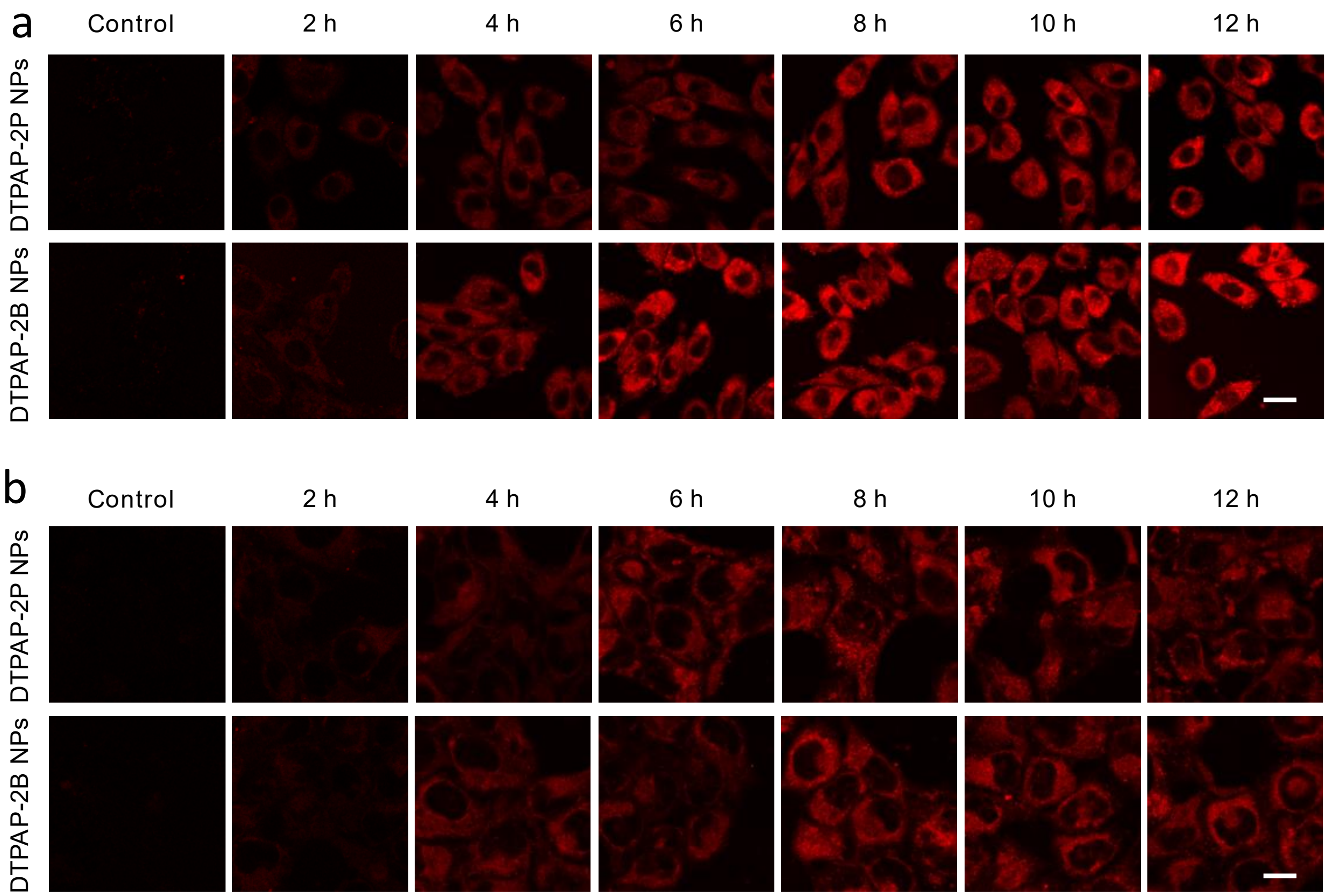

Supplementary Figure 55. Uptake of DTPAP-2P NPs and DTPAP-2B NPs by 4T1 cells (**a**) and HeLa cells (**b**) at different incubation times. Scale bar = 20 μm. Control represents the cells incubated with the absence of any NPs.

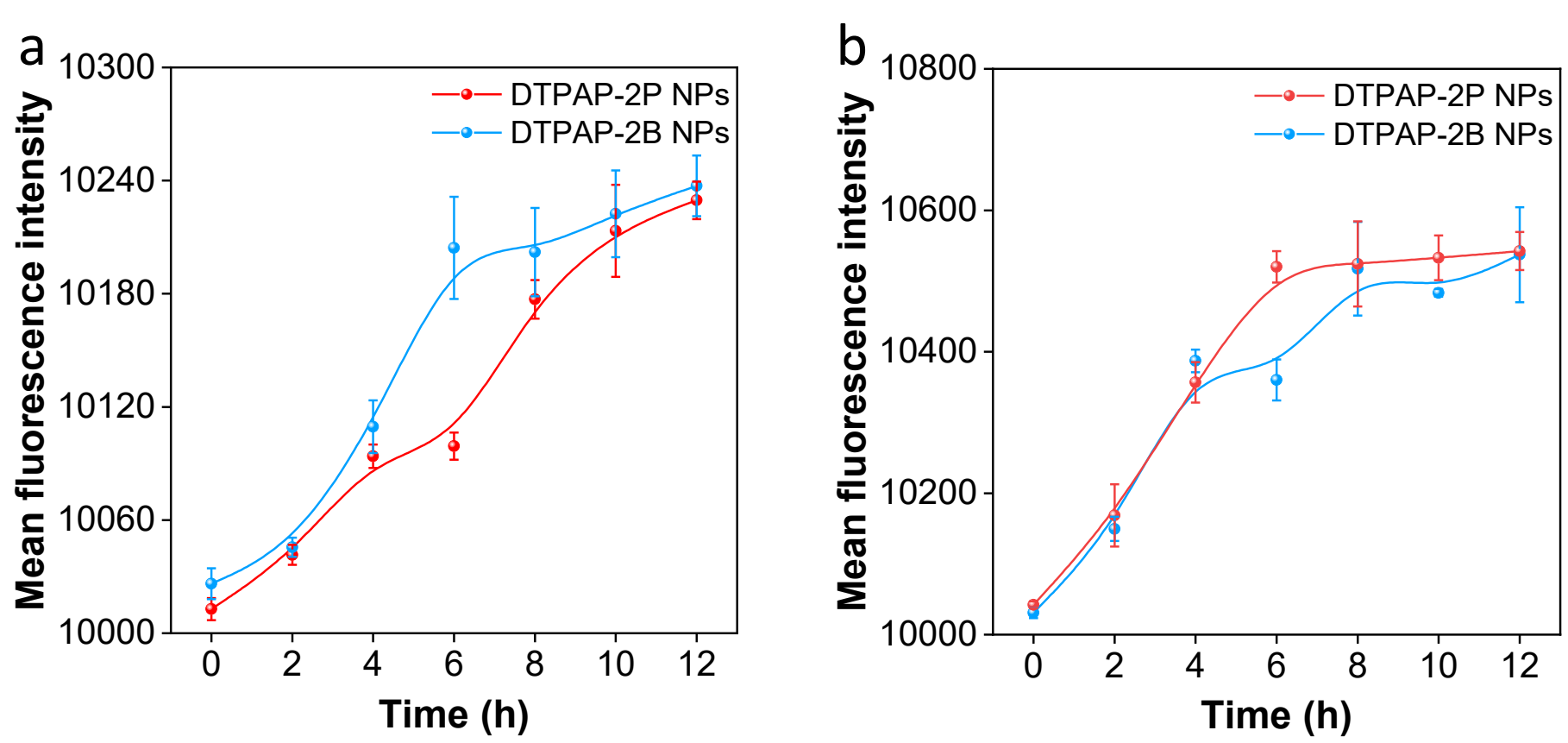

Supplementary Figure 56. The mean fluorescence intensity of DTPAP-2P NPs and DTPAP-2B NPs inside 4T1 cells (**a**) and HeLa cells (**b**) at different incubation times obtained from Supplementary Figure 55. Data were presented as mean values ± SEM.

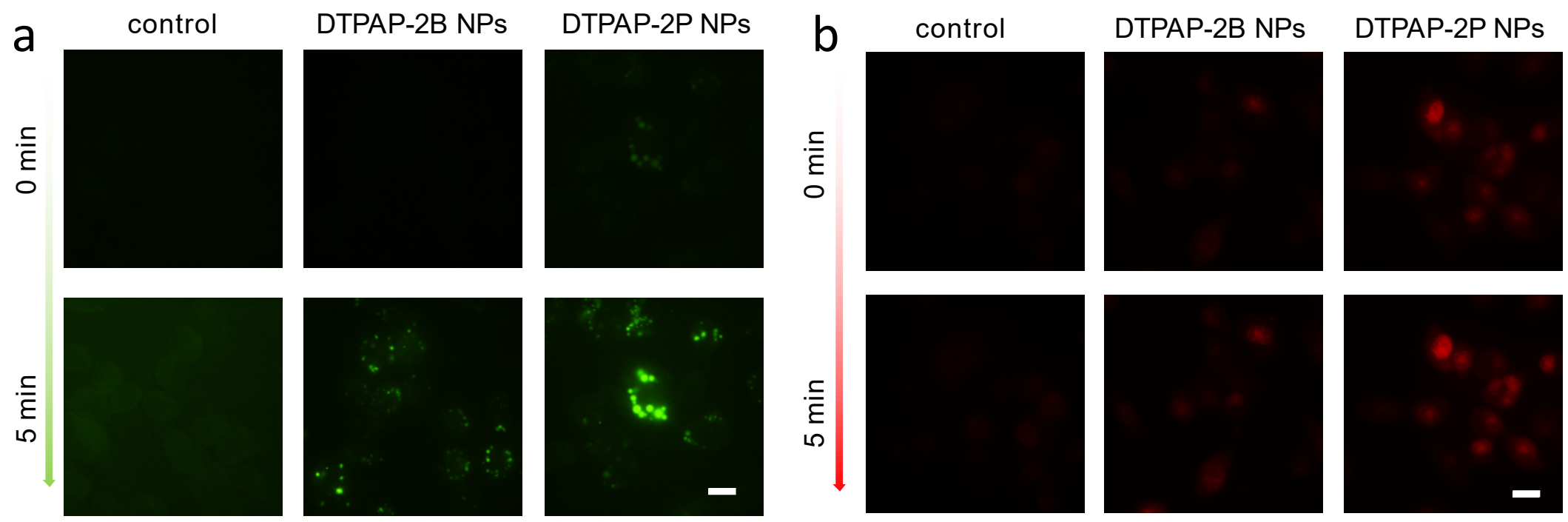

Supplementary Figure 57. Imaging of (**a**) APF and (**b**) DHE inside HeLa cells incubated with 660 nm irradiation for different times (0.1 W cm^{-2}). Scale bar = 20 μm.

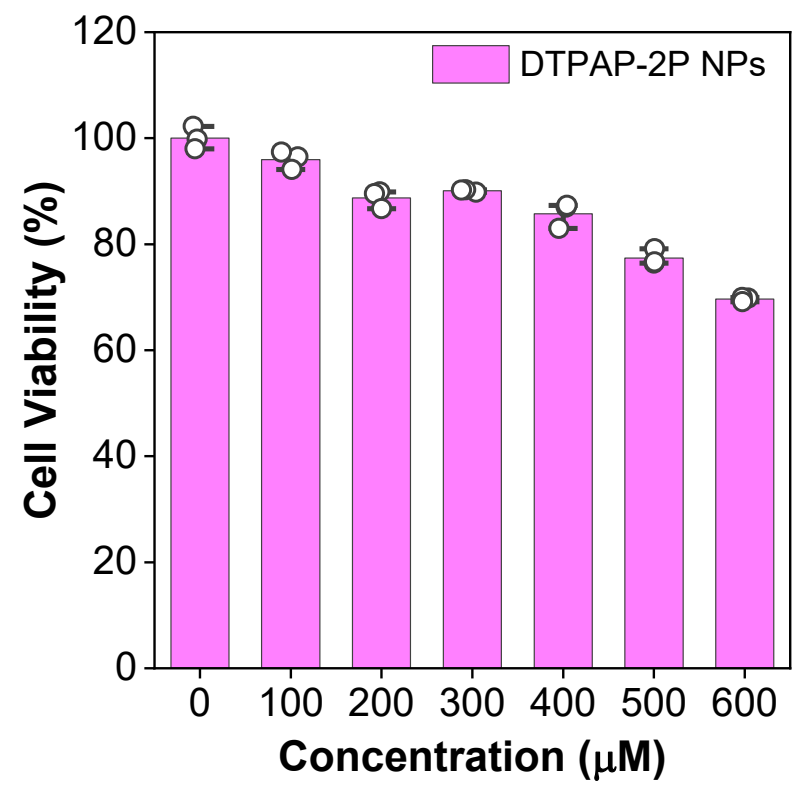

Supplementary Figure 58. The MTT assay was used to detect the dark cytotoxicity of DTPAP-2P NPs toward 4T1 cells in different concentrations (n = 3, independent experimental replicates). Data were presented as mean values ± SEM.

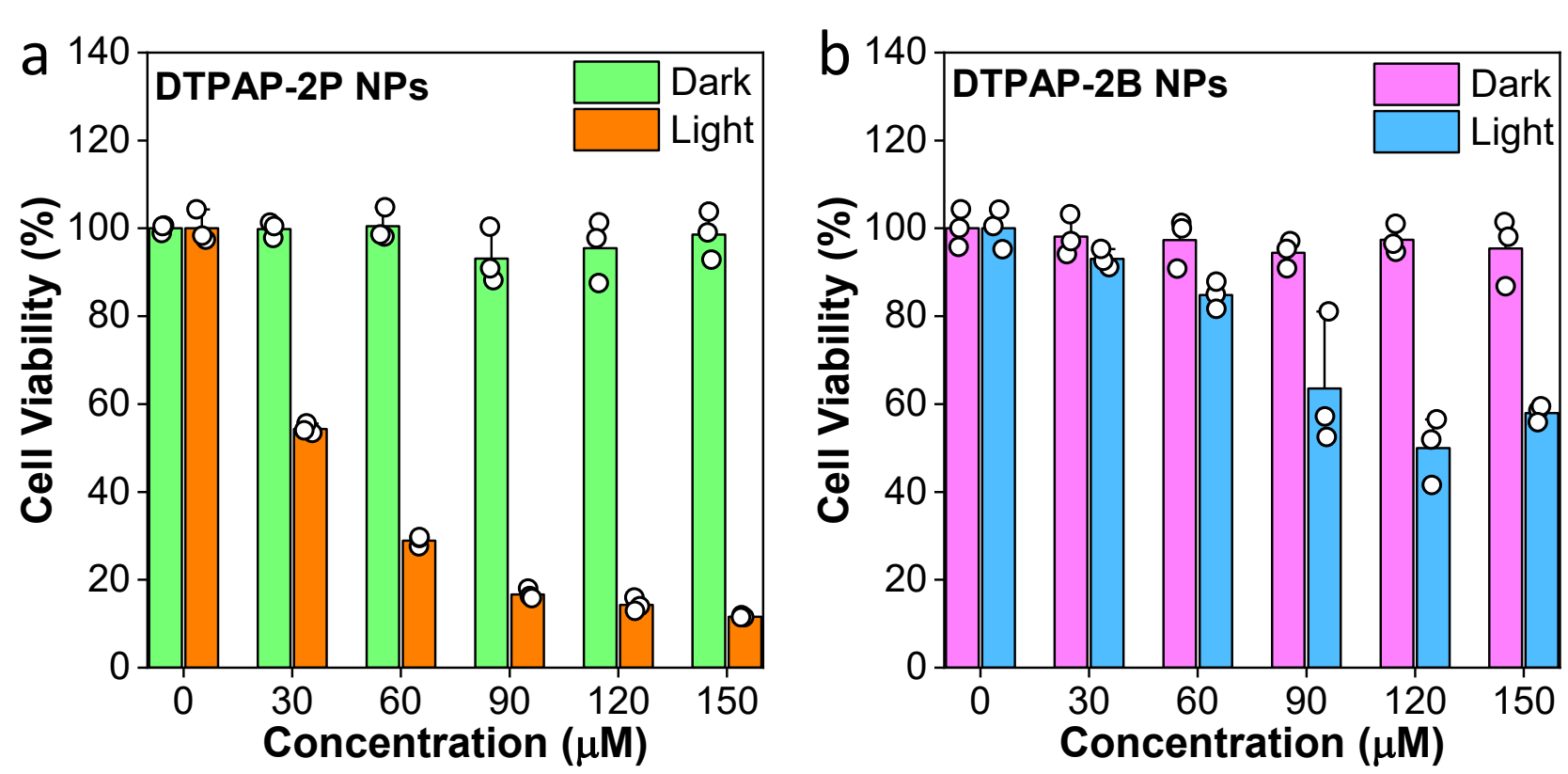

Supplementary Figure 59. Viability of HeLa cancer cells incubated with increasing concentrations of DTPAP-2P NPs (**a**) and DTPAP-2B NPs (**b**) without or with 660 nm laser irradiation (0.4 W cm^{-2}) for 10 min (n = 3, independent experimental replicates). Data in **a** and **b** were presented as mean values ± SEM.

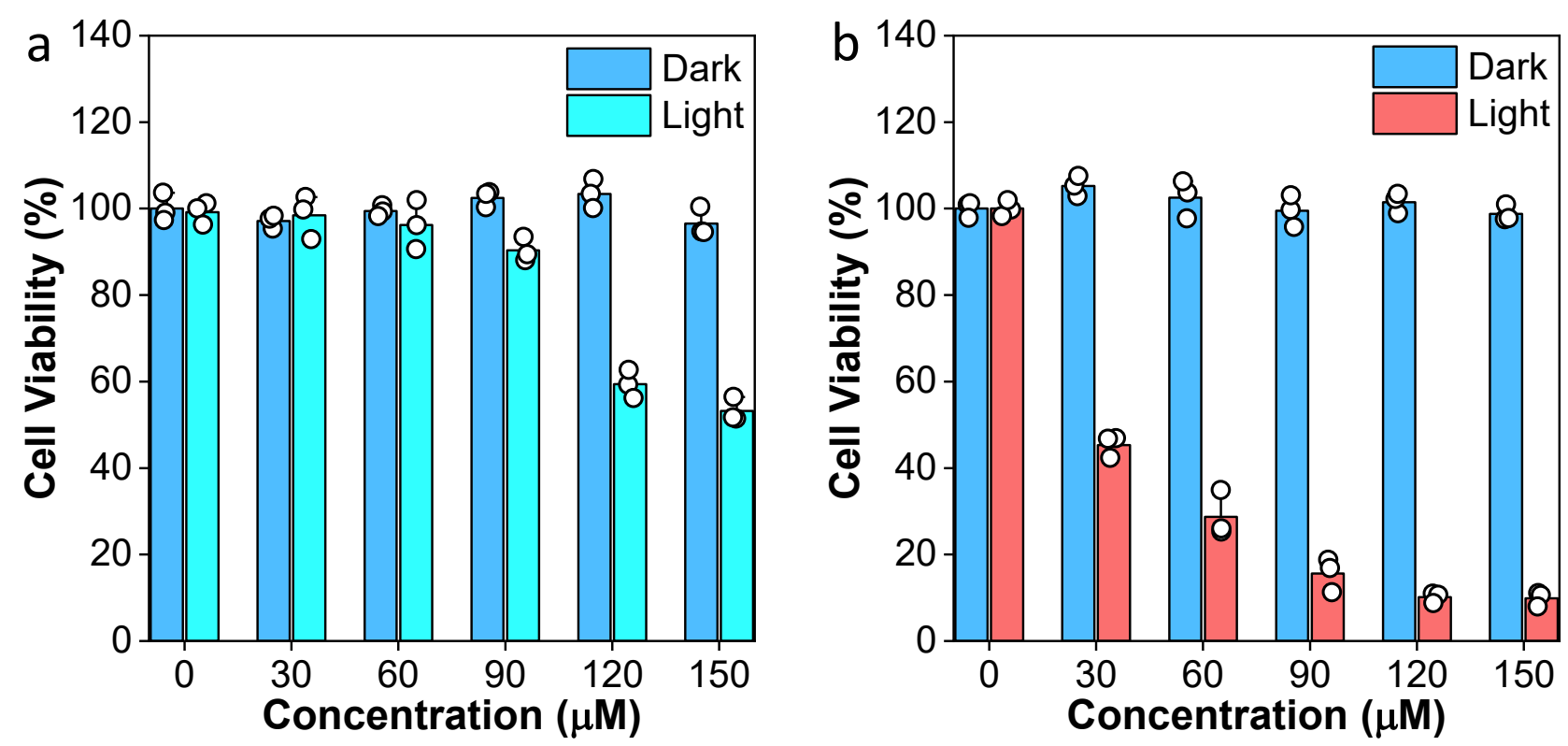

Supplementary Figure 60. (**a**) Viability of 4T1 cells treated by various amounts of DTPAP-2P NPs (0–150 μM) without and with 660 nm light exposure in 4 °C ice-water. (**b**) Viability of 4T1 cells pretreated with Vc, following treatment with increasing concentrations (0–150 μM) of DTPAP-2P NPs with or without 660 nm light irradiation (n = 3, independent experimental replicates). Data in **a** and **b** were presented as mean values ± SEM.

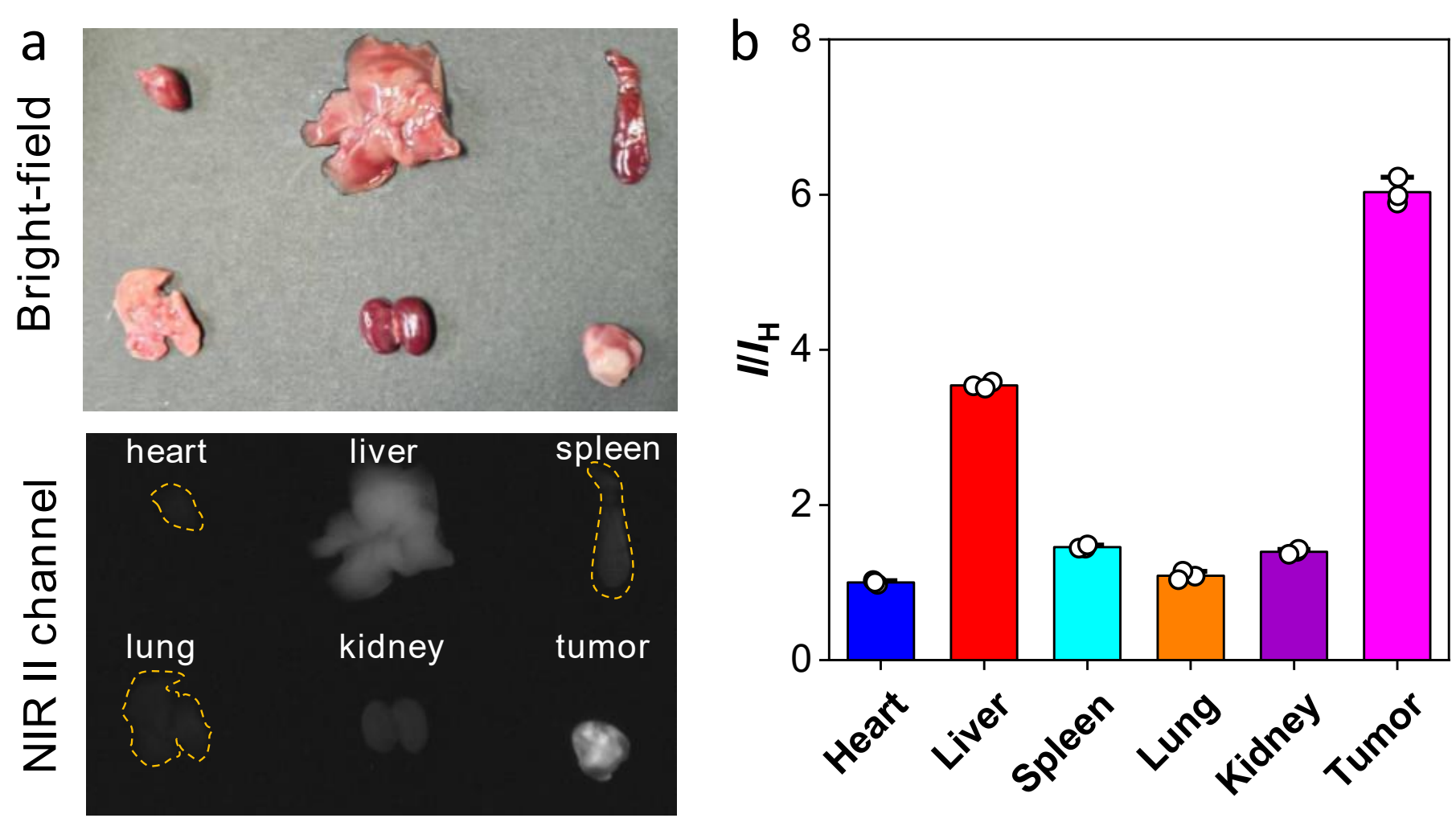

Supplementary Figure 61. (**a**) The images of the major organs (heart, liver, spleen, lung, kidney) and tumor in bright-field and NIR-II channel after 24 h post-injection of DTPAP-2P NPs. (**b**) The I/I_H ratio of the major organ (heart, liver, spleen, lung, kidney) after 24 h post-injection of DTPAP-2P NPs. I represents the PL intensity of the major organ, while I_H indicates the PL intensity of the heart. Data in (**b**) were presented as mean values ± SEM.

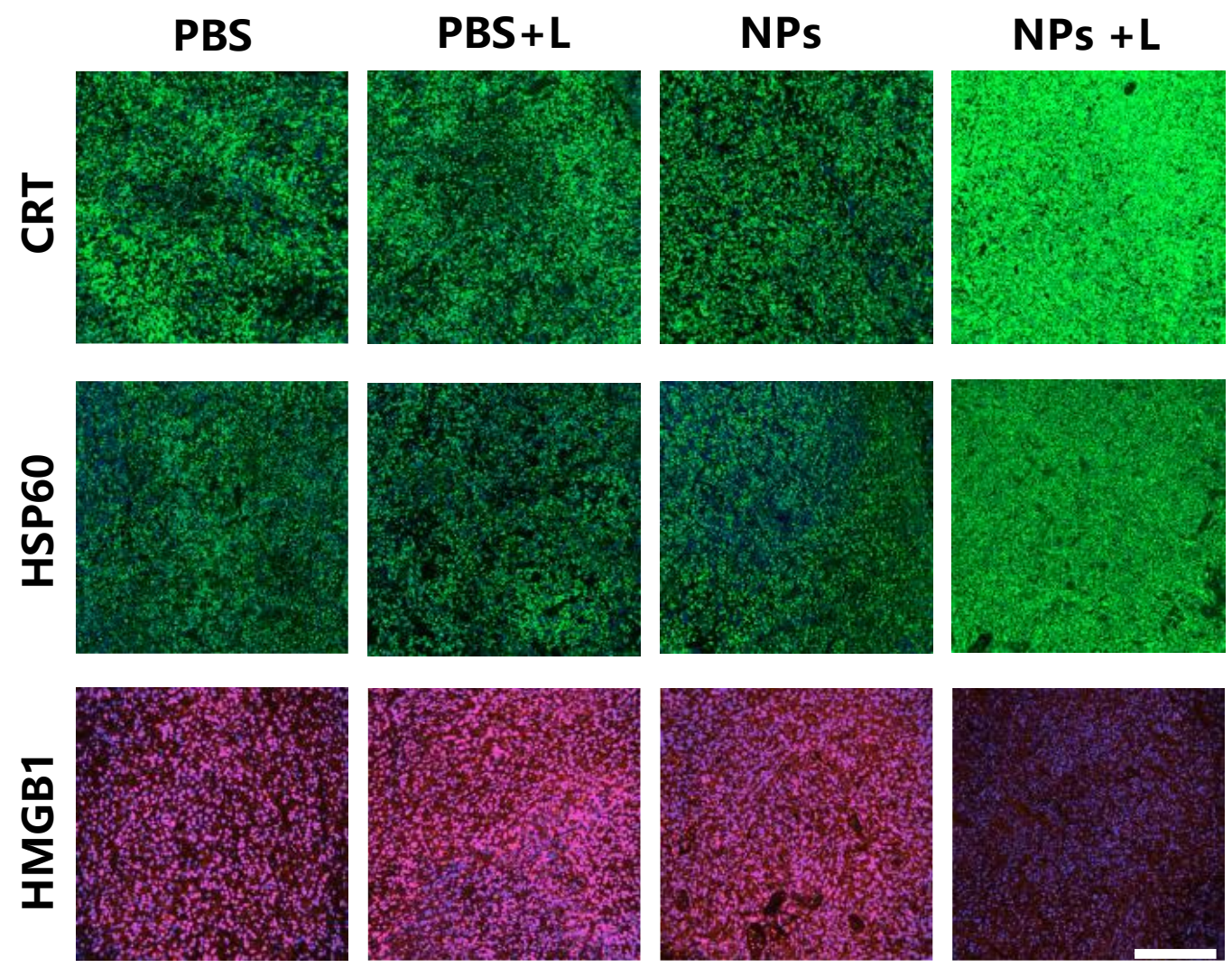

Supplementary Figure 62. Immunofluorescence staining of CRT, HSP60 and HMGB1 to evaluate ICD-related DAMPs exposure in tumor tissues after different treatments. Scale bar: 200 μm.

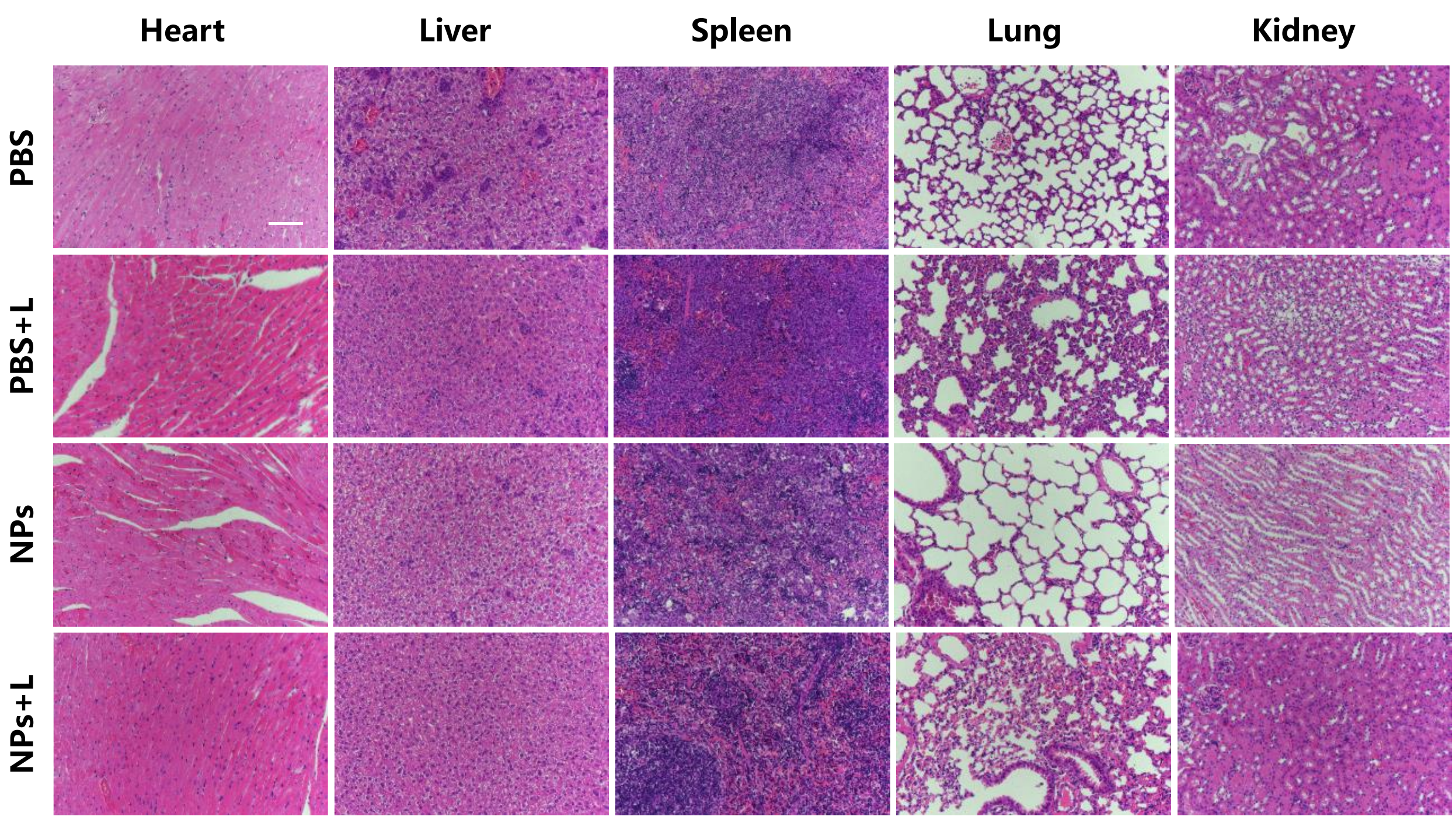

Supplementary Figure 63. Histological H&E staining of major organs (heart, liver, spleen, lung, kidney) of mice in different groups. Scale bar =100 μm.

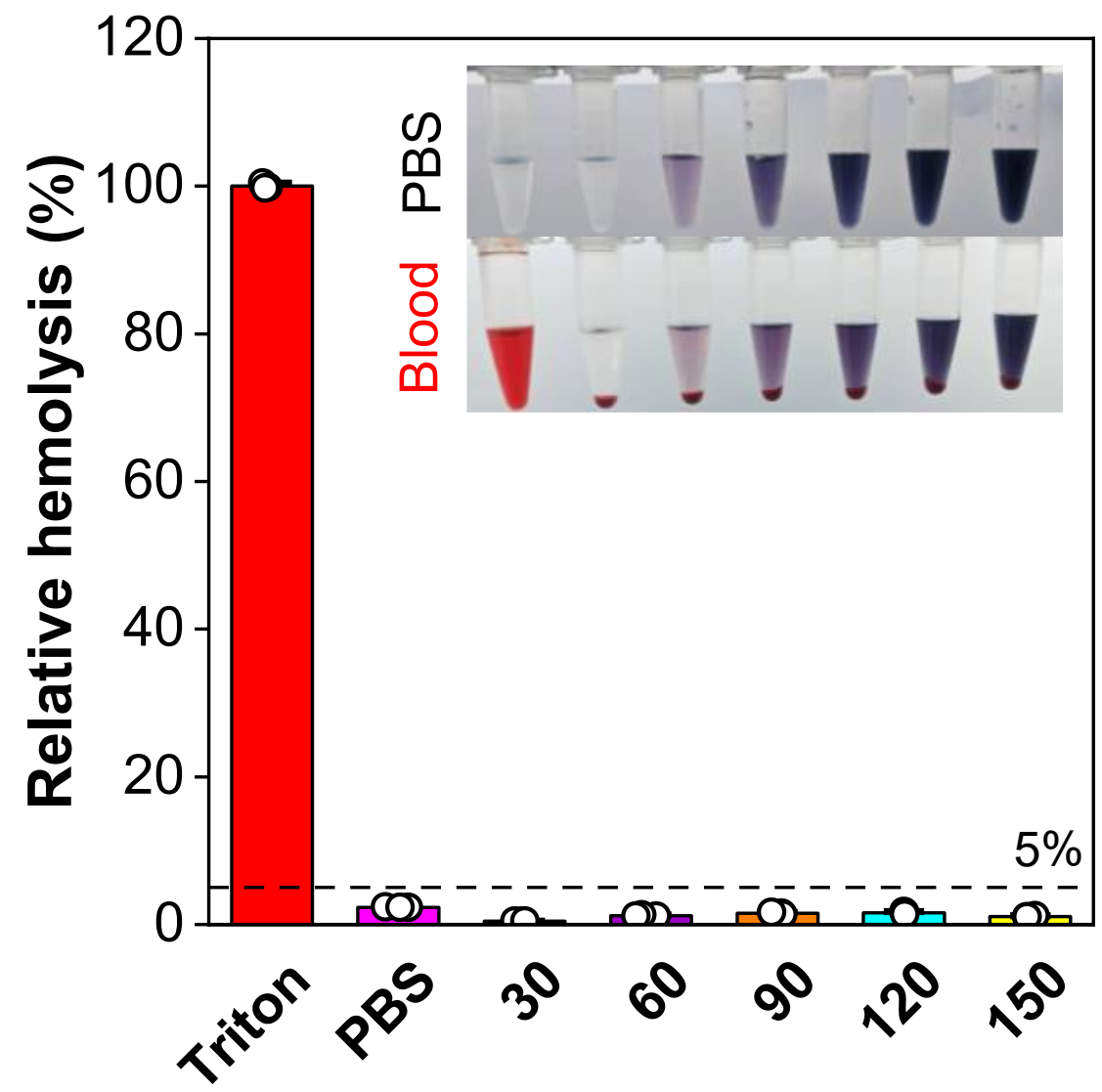

Supplementary Figure 64. Hemolysis rates of DTPAP-2P NPs. Triton and PBS were used as the positive control and negative control, respectively. Inset: the photographs of hemolytic activity (n = 3, independent experimental replicates). Data were presented as mean values ± SEM.

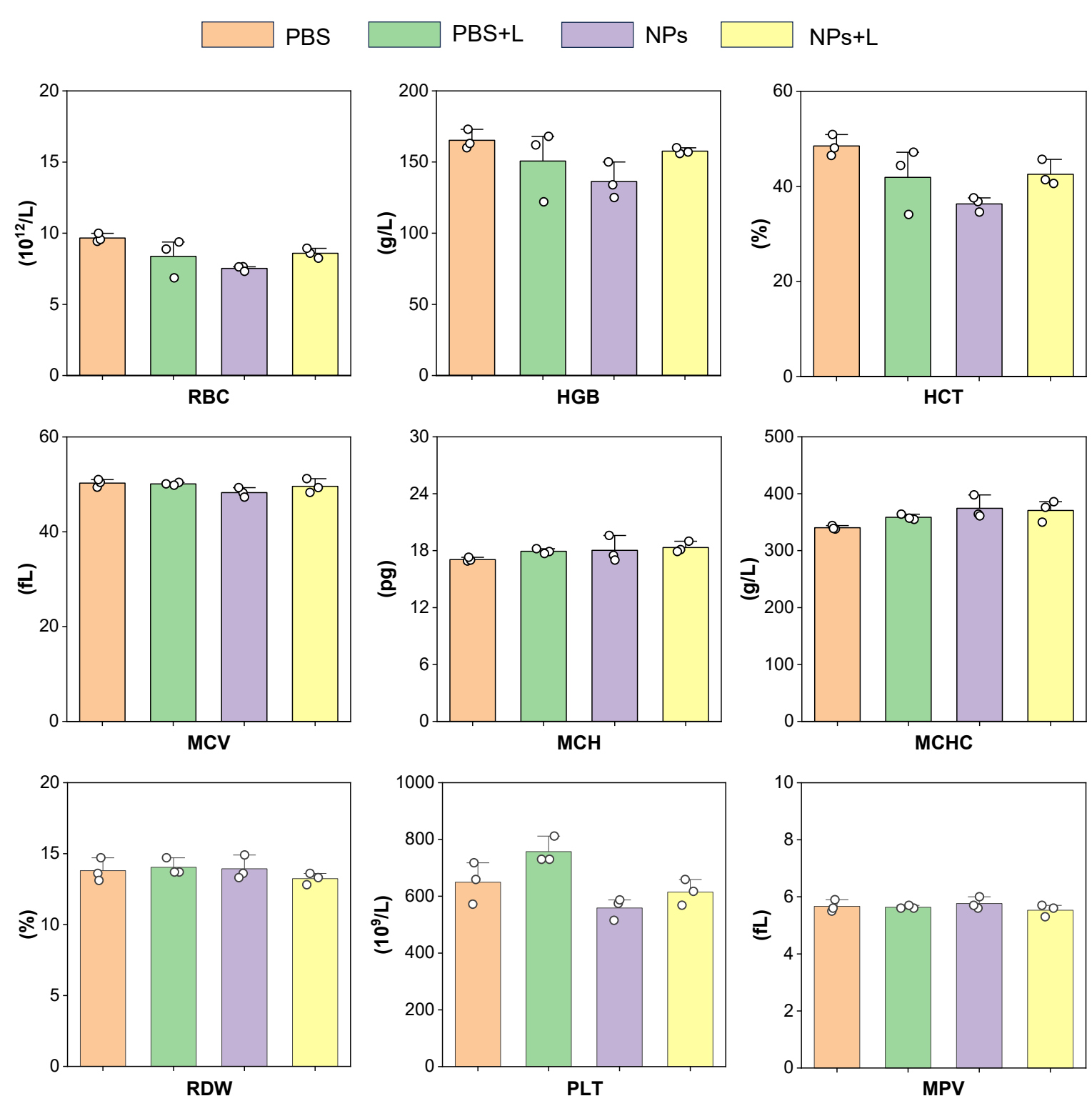

Supplementary Figure 65. Blood routine assays of mice in different groups. (n = 3, biologically independent mice). Data were presented as mean values ± SEM.

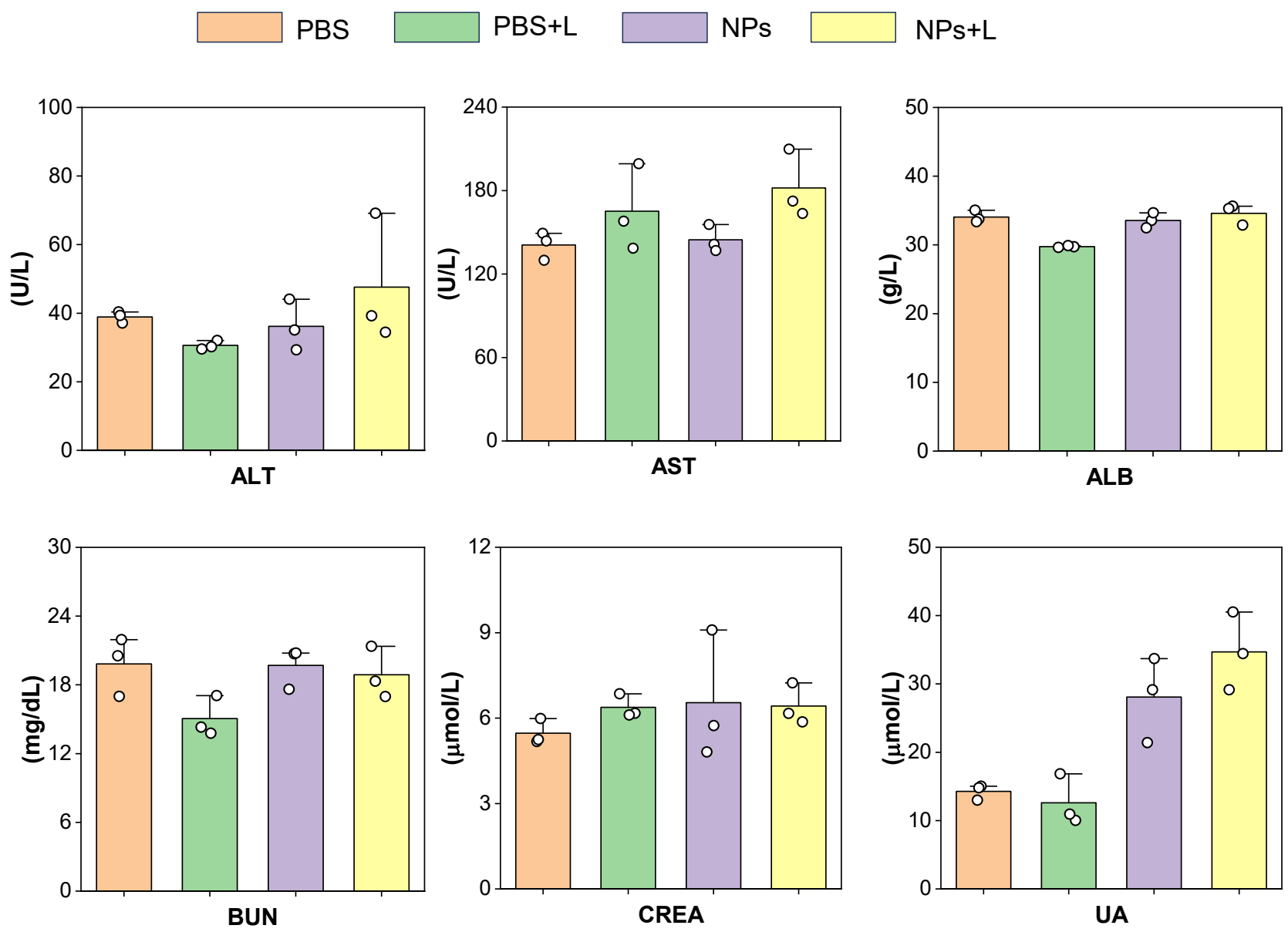

Supplementary Figure 66. Blood biochemistry test regarding liver and kidney function of the mice in different groups. (n = 3, biologically independent mice). Data were presented as mean values ± SEM.

References

S1. Xu, Y.; Dang, D.; Zhang, N.; Zhang, J.; Xu, R.; Wang, Z.; Zhou, Y.; Zhang, H.; Liu, H.; Yang, Z.; Meng, L.; Lam, J. W. Y.; Tang, B. Z., *ACS Nano* **2022**, 16, 5932-5942.

S2. Li, Q.; Gong, J.; Li, Y.; Zhang, R.; Wang, H.; Zhang, J.; Yan, H.; Lam, J. W. Y.; Sung, H. H. Y.; Williams, I. D.; Kwok, R. T. K.; Li, M.-H.; Wang, J.; Tang, B. Z., *Chem. Sci.* **2021**, 12, 709−717.

S3. Liu, L.; Li, C.; Gong, J.; Zhang, Y.; Ji, W.; Feng, L.; Jiang, G.; Wang, J.; Tang, B. Z., *Angew. Chem., Int. Ed.* **2023**, 62, e202307776.

S4. Wang, Y.; Zhang, C.; Li, Y.; Li, R.; Li, J.; Feng, L., *Dyes Pigm.* **2023**, 218, 111517.

S5. Xiao, P.; Shen, Z.; Wang, D.; Pan, Y.; Li, Y.; Gong, J.; Wang, L.; Wang, D.; Tang, B. Z., *Adv. Sci.* **2022**, 9, 2104079.

S6. Li, Q.; Li, Y.; Min, T.; Gong, J.; Du, L.; Phillips, D. L.; Liu, J.; Lam, J. W. Y.; Sung, H. H. Y.; Williams, I. D.; Kwok, R. T. K.; Ho, C. L.; Li, K.; Wang, J.; Tang, B. Z., *Angew. Chem., Int. Ed.* **2020**, 59, 9470-9477.

S7. Wan, Q.; Zhang, R.; Zhuang, Z.; Li, Y.; Huang, Y.; Wang, Z.; Zhang, W.; Hou, J.; Tang, B. Z., *Adv. Funct. Mater.* **2024**, 34, 2312870.

S8. Wang, Y.; Liao, J.; Lyu, Y.; Guo, Q.; Zhu, Z.; Wu, X.; Yu, J.; Wang, Q.; Zhu, W.-H., *Adv. Funct. Mater.* **2023**, 33, 2301692.

S9. Yang, S.; Yu, H.; Liu, J.; Ma, L.; Hou, Z.; Ma, J.; Miao, M. Z.; Kwok, R. T. K.; Sun, J.; Sung, H. H. Y.; Williams, I. D.; Lam, J. W. Y.; Liu, X.; Tang, B. Z., *ACS Nano* **2023**, 17, 21182-21194.

S10. Liu, L.; Gong, J.; Wang, X.; Zhang, Y.; Jiang, G.; Wang, J.; Tang, B. Z., *Aggregate* **2026**, 7, e70267.

S11. Mule, R. D.; Roy, R.; Mandal, K.; Chopra, D.; Dutta, T.; Sancheti, S. P.; Shinde, P. S.; Banerjee, S.; Koner, A. L.; Bhowal, R.; Senthilkumar, B.; Patil, N. T., *Chem. Eur. J.* **2022**, 28, e202200632.

S12. Chen, K.; He, P.; Wang, Z.; Tang B. Z., *ACS Nano* **2021**, 15, 7735-7743.
