# Supplementary material for: Organic planar π-conjugated anions enable structural design of anion-π+ NIR-II phototheranostic agents
Source: Nat Commun. 2026 Jul 8;17:8449. doi: 10.1038/s41467-026-75247-7 (PMC13478202; doi:10.1038/s41467-026-75247-7)
Supplement: Supplementary file 2 [file 41467_2026_75247_MOESM2_ESM.pdf]

Description of Additional Supplementary Files

File Name: Supplementary Data 1. Atomic coordinates of the optimized computational models

Description: Atomic coordinates of DTPAP-2P and DTPAP-2B in .txt format
