# Supplementary material for: Organic planar π-conjugated anions enable structural design of anion-π+ NIR-II phototheranostic agents
Source: Nat Commun. 2026 Jul 8;17:8449. doi: 10.1038/s41467-026-75247-7 (PMC13478202; doi:10.1038/s41467-026-75247-7)
Supplement: Supplementary file 3 [file 41467_2026_75247_MOESM4_ESM.pdf]

nature portfolio

Corresponding author(s): Guoyu Jiang, Jianguo Wang, Ben Zhong Tang

Last updated by author(s): June 18, 2026

Reporting Summary

Nature Portfolio wishes to improve the reproducibility of the work that we publish. This form provides structure for consistency and transparency in reporting. For further information on Nature Portfolio policies, see our Editorial Policies and the Editorial Policy Checklist.

Statistics

For all statistical analyses, confirm that the following items are present in the figure legend, table legend, main text, or Methods section.

n/a	Confirmed	
[ ]	[x]	The exact sample size (*n*) for each experimental group/condition, given as a discrete number and unit of measurement
[ ]	[x]	A statement on whether measurements were taken from distinct samples or whether the same sample was measured repeatedly
[ ]	[x]	The statistical test(s) used AND whether they are one- or two-sided<br>*Only common tests should be described solely by name; describe more complex techniques in the Methods section.*
[x]	[ ]	A description of all covariates tested
[ ]	[x]	A description of any assumptions or corrections, such as tests of normality and adjustment for multiple comparisons
[ ]	[x]	A full description of the statistical parameters including central tendency (e.g. means) or other basic estimates (e.g. regression coefficient) AND variation (e.g. standard deviation) or associated estimates of uncertainty (e.g. confidence intervals)
[ ]	[x]	For null hypothesis testing, the test statistic (e.g. *F*, *t*, *r*) with confidence intervals, effect sizes, degrees of freedom and *P* value noted<br>*Give P values as exact values whenever suitable.*
[x]	[ ]	For Bayesian analysis, information on the choice of priors and Markov chain Monte Carlo settings
[x]	[ ]	For hierarchical and complex designs, identification of the appropriate level for tests and full reporting of outcomes
[ ]	[x]	Estimates of effect sizes (e.g. Cohen's *d*, Pearson's *r*), indicating how they were calculated

Our web collection on statistics for biologists contains articles on many of the points above.

Software and code

Policy information about availability of computer code

Data collection	Structural characterization. The organic compounds synthesized were characterized for their structures and purities using 1H, 13C NMR and 2D NMR spectroscopy spectra with Bruker ARX 500 NMR or Bruker ARX 600 NMR spectrometer using tetramethylsilane (TMS) as a reference at room temperature. High-resolution mass spectra were collected by a LCT premier XE (Waters) HRMS spectrometry or XEVO G2-XS QTOF (Waters) HRMS spectrometry. X-ray photoelectron spectroscopy (XPS) was measured by a Thermo ESCALAB 250Xi spectrometer. The ion chromatography (Combustion IC) was measured by a 930 Combustion IC PP (AJ) system. Absorption spectra were measured on a SHIMADZU UV-2600i spectrophotometer. Photoluminescence (PL) spectra were recorded with an FS5 spectrofluorometer using 660 nm laser as excitation light source (CNI laser, MDL-XD-660). The absolute fluorescence quantum yield was measured using a Hamamatsu quantum yield spectrometer C11347-11 Quantaurus QY. The size distribution was analyzed by dynamic light scattering (DLS) using an Omni NanoBrook. Particle size and morphology were observed on a Hitachi HT 7800 transmission electron microscope (TEM). Temperature change was monitored by an FLIR E8-XT camera (FLIR System). Photodynamic and photothermal experiments were conducted using a 660 nm laser (CNI laser, MDL-XD-660) as the excitation light source. The cell viability was assessed by MTT assay, and the absorbance of each sample was measured at 490 nm using a microplate reader (BioTek). The in vivo fluorescence images were acquired on the commercial NIR-II Small Animal Imaging System Series III 900/1700S (Suzhou NIR-Optics Technology Co., Ltd., China) equipped with 900 nm long-pass (LP) filters. The blood biochemistry parameters were collected by the fully automatic biochemical analysis (Chemray 240). Cellular imaging experiments were performed with confocal laser scanning microscope (LSM880, ZEISS, Germany) or inverted fluorescence microscope (Nikon Ti2).
Data analysis	Weak interaction analysis from single crystal structure were performed using Multiwfn, and corresponding structure and IGM isosurfaces were generated using the VMD program. Statistical analysis of data was conducted using SPSS 23 software.

For manuscripts utilizing custom algorithms or software that are central to the research but not yet described in published literature, software must be made available to editors and reviewers. We strongly encourage code deposition in a community repository (e.g. GitHub). See the Nature Portfolio guidelines for submitting code & software for further information.

Data

Policy information about availability of data

All manuscripts must include a data availability statement. This statement should provide the following information, where applicable:

- Accession codes, unique identifiers, or web links for publicly available datasets
- A description of any restrictions on data availability
- For clinical datasets or third party data, please ensure that the statement adheres to our policy

All original data generated in this study are provided in the Supplementary Information/Source Data file. The X-ray crystallographic coordinates for structures reported in this study have been deposited at the Cambridge Crystallographic Data Centre (CCDC), under deposition number 2387016 for DTPAP-2B and 2466906 for DTPAP-2P. These data can be obtained free of charge from The Cambridge Crystallographic Data Centre via www.ccdc.cam.ac.uk/data_request/cif. There are no restrictions on data availability in the current work. Source data are provided with this paper. Correspondence and requests for materials should be addressed to Jianguo Wang.

Research involving human participants, their data, or biological material

Policy information about studies with human participants or human data. See also policy information about sex, gender (identity/presentation), and sexual orientation and race, ethnicity and racism.

Reporting on sex and gender	N/A
Reporting on race, ethnicity, or other socially relevant groupings	N/A
Population characteristics	N/A
Recruitment	N/A
Ethics oversight	N/A

Note that full information on the approval of the study protocol must also be provided in the manuscript.

Field-specific reporting

Please select the one below that is the best fit for your research. If you are not sure, read the appropriate sections before making your selection.

☒ Life sciences ☐ Behavioural & social sciences ☐ Ecological, evolutionary & environmental sciences

For a reference copy of the document with all sections, see nature.com/documents/nr-reporting-summary-flat.pdf

Life sciences study design

All studies must disclose on these points even when the disclosure is negative.

Sample size	No statistical method was used to predetermine sample size in the study. Sample sizes were chosen based on reproducibility across independent experiments and established conventions in the field. Reported results were consistently replicated across multiple experiments with all replicates generating similar results. For cell-based experiments, n= 3 biologically independent experiments were performed. For animal studies, group sizes were typically n= 3-6 biologically independent mice per group. The definition of n and the exact sample sizes for each experiment are provided in the figure legends.
Data exclusions	No data were excluded from the analyses.
Replication	All major experiments were performed in at least 3 independent experimental replicates following identical procedures to verify the reproducibility of the experimental results.
Randomization	The cells and animals used in this paper were randomly distributed into several groups for the further experiments.
Blinding	No formal blinding was used. The investigator organizing the experimental groups and involved in sample collection was not blinded because the metrics were quantified and analyzed objectively. However, colleagues aiding in data collection were blinded.

Reporting for specific materials, systems and methods

We require information from authors about some types of materials, experimental systems and methods used in many studies. Here, indicate whether each material, system or method listed is relevant to your study. If you are not sure if a list item applies to your research, read the appropriate section before selecting a response.

Materials & experimental systems

n/a	Involved in the study	
☐	☒	Antibodies
☐	☒	Eukaryotic cell lines
☒	☐	Palaeontology and archaeology
☐	☒	Animals and other organisms
☒	☐	Clinical data
☒	☐	Dual use research of concern
☒	☐	Plants

Methods

n/a	Involved in the study	
☒	☐	ChIP-seq
☒	☐	Flow cytometry
☒	☐	MRI-based neuroimaging

Antibodies

Antibodies used	The primary antibodies used in this study included CRT (Beyotime, shanghai, China, catalog no. AF1666, rabbit polyclonal; IF1:200;), HMGB1 (Servicebio, wuhan, China, catalog no. GB11103, rabbit polyclonal; IF 1:500), and HSP60 (Servicebio, wuhan, China, catalog no. GB15243, mouse monoclonal; IF 1:500). For immunofluorescence assays, fluorophore-conjugated secondary antibodies were applied, including Alexa Fluor® 488-conjugated Goat Anti-Rabbit IgG (H+L) (Servicebio, wuhan, China, catalog no, GB25303; 1:400), Cy3 conjugated Goat Anti-Rabbit IgG (H+L) (Servicebio, wuhan, China, catalog no, GB21303; 1:300) and Alexa Fluor® 488-conjugated Goat Anti-Mouse IgG (H+L) (Servicebio, wuhan, China, catalog no, GB25301; 1:400). All antibodies were used according to the manufacturers' instructions.
Validation	All primary antibodies used in this study were validated by the suppliers, with each lot quality-tested. CRT (Beyotime, shanghai, China, catalog no. AF1666, https://www.beyotime.com/product/AF1666.htm; rabbit polyclonal; IF 1:200), HMGB1 (Servicebio, wuhan, China, catalog no. GB11103, https://www.servicebio.cn/goodsdetail?id=1383; rabbit polyclonal; IF 1:500), and HSP60 (Servicebio, wuhan, China catalog no. GB15243, https://www.servicebio.cn/goodsdetail?id=20511; mouse monoclonal; IF 1:500) were validated for human and mouse in immunofluorescence staining.<br>Alexa Fluor® 488-conjugated Goat Anti-Rabbit IgG (H+L) (Servicebio, wuhan, China, catalog no, GB25303, https://www.servicebio.cn/goodsdetail?id=273; 1:400), Cy3 conjugated Goat Anti-Rabbit IgG (H+L) (Servicebio, wuhan, China, catalog no, GB21303, https://www.servicebio.cn/goodsdetail?id=253; 1:300) and Alexa Fluor® 488-conjugated Goat Anti-Mouse IgG (H+L) (Servicebio, wuhan, China, catalog no, GB25301, https://www.servicebio.cn/goodsdetail?id=272; 1:400) were used according to the manufacturer's instructions and validated by the suppliers. Validation data are available on the manufacturers' websites.

Eukaryotic cell lines

Policy information about cell lines and Sex and Gender in Research

Cell line source(s)	The HeLa and 4T1 cells lines used herein were obtained from our laboratory stocks. And the original sources were ATCC cell bank (HeLa: ATCC® CCL-2; 4T1: ATCC® CRL-2539).
Authentication	The cell lines were authenticated by periodic morphology checks using a microscope.
Mycoplasma contamination	We confirm that HeLa and 4T1 cells tested negative for mycoplasma contamination.
Commonly misidentified lines (See ICLAC register)	There are no misidentified cell lines in our study.

Animals and other research organisms

Policy information about studies involving animals; ARRIVE guidelines recommended for reporting animal research, and Sex and Gender in Research

Laboratory animals	Female nude mice and BALB/c mice (6–8 weeks old, average body weight 16–18 g) were purchased from SPF Biotechnology Co., Ltd. (Beijing, China). All procedures were approved by the Institutional Animal Care and Use Committee at the Inner Mongolia University (IMU-2023-mouse-017). All mice were acclimatized to the animal facility for one week prior to experimentation and housed under pathogen-free conditions, fed under conditions of 25 °C and 55% of humidity and allowed free access to standard laboratory water and chow.
Wild animals	The study did not involve wild animals.
Reporting on sex	For the tumor mouse model, female nude mice and BALB/c mice (6–8 weeks old, average body weight 16–18 g) were used. This selection was based on the majority of existing literatures. This study results were independent of sex and gender.
Field-collected samples	The study did not involve samples collected from the field.
Ethics oversight	All procedures were approved by the Institutional Animal Care and Use Committee at the Inner Mongolia University (IMU-2023-mouse-017). All mice were acclimatized to the animal facility for one week prior to experimentation and housed under pathogen-free conditions, fed under conditions of 25 °C and 55% of humidity and allowed free access to standard laboratory water and chow.

Note that full information on the approval of the study protocol must also be provided in the manuscript.

Plants

Seed stocks	This study doesn't involve plants.
Novel plant genotypes	This study doesn't involve plants.
Authentication	This study doesn't involve plants.
