# Supplementary material for: Organic planar π-conjugated anions enable structural design of anion-π+ NIR-II phototheranostic agents
Source: Nat Commun. 2026 Jul 8;17:8449. doi: 10.1038/s41467-026-75247-7 (PMC13478202; doi:10.1038/s41467-026-75247-7)
Supplement: Supplementary file 4 [file 41467_2026_75247_MOESM5_ESM.pdf]

nature portfolio

Peer Review File

Organic Planar π-Conjugated Anions Enable Structural Design of Anion-π^+ NIR-II Phototheranostic Agents

Corresponding Author: Professor Jianguo Wang

This file contains all reviewer reports in order by version, followed by all author rebuttals in order by version.

Version 0:

Reviewer comments:

Reviewer #1

(Remarks to the Author)
In this manuscript, Zhang et al. propose an organic planar π-conjugated anion exchange strategy for constructing high-performance anion–π^+ type NIR-II photothermal agents (PTAs). Previous studies on anion–π^+ type phototheranostic systems have predominantly focused on inorganic counteranions. In contrast, the authors ingeniously exploit the large π-conjugation and intrinsic planarity of an organic π-conjugated anion to induce strong electrostatically driven donor–acceptor interactions and anion–π^+ interactions. This strategy effectively promotes red-shifted absorption and emission as well as enhanced emission intensity, enabling the realization of an anion–π^+ phototheranostic system with NIR-II emission. As a proof of concept, the authors synthesized two anion-π^+ type phototheranostic agents, DTPAP-2B with Br- and DTPAP-2P with an organic planar π-conjugated pentadecyanocyclopentadiene anion (C5CN5-). Experimental results demonstrate that DTPAP-2P NPs exhibit a higher molar extinction coefficient (1.16×10^4 L·mol^{-1}·cm^{-1} at 660 nm), pronounced NIR-II emission, more efficient ROS generation, and a significantly enhanced photothermal conversion efficiency (72%) compared with DTPAP-2B NPs.
Overall, this manuscript is highly interesting and innovative. The study is well organized and supported by solid experimental evidence, including comprehensive structural characterization, systematic photophysical analysis, and convincing in vitro and in vivo evaluations. The conclusions are well justified by the presented data. The research content fits well within the scope of Nature Communications. Therefore, I believe this work is suitable for publication after minor revision. The following points are suggested for further clarification and strengthening of the manuscript:
(1) Please provide additional details on the size distribution and stability of DTPAP-2P NPs and DTPAP-2B NPs (e.g., DLS in biological media) to support their suitability for in vivo imaging and therapy.
(2) The authors attribute the improved performance of DTPAP-2P mainly to anion–π^+ interactions introduced by the π-conjugated counterion. What distinguishes this interaction from general anion effects or other counterion strategies, and what are its specific advantages in this system?
(3) The authors state that NIR-II PTAs based on anion–π^+ interactions have not yet been successfully developed. However, the manuscript lacks a concise comparison with previously reported anion–π^+ PSs. Could the authors briefly summarize the typical absorption and emission ranges of reported anion–π^+ PSs, and clearly clarify the key advantages of DTPAP-2P NPs?
(4) Given the potential application of DTPAP-2P NPs in long-term NIR-II imaging and phototherapy, it would be helpful to evaluate their photothermal stability under repeated laser irradiation. Including a comparison with a representative commercial NIR dye would further clarify the practical advantages of DTPAP-2P NPs.
(5) The authors are encouraged to include comparative cellular imaging using additional ROS probes to further validate the type and intracellular generation of ROS induced by the photosensitizer.
(6) The evaluation of biological safety of new system is very important. The hemocompatibility of DTPAP-2P NPs should be performed.

Reviewer #2

(Remarks to the Author)
In the present work, J. Wang and his co-worker, reports on how the association of a pi-conjugated cation with a planar pi-conjugated anion can enhance the absorption and fluorescence properties in the NIR region in the solid state in the form of nanoparticles as well as the ROS production and photothermal activity. This nanoparticulate system efficiently enable high-

resolution NIR-II angiography and effective NIR-II imaging-guided synergistic photodynamic/photothermal therapy. This work is original in the context of new fluorescent probe and photothermal agents, is well referenced, and the results obtained support the stated conclusions and claims. However, the authors state that the introduction of planar π-conjugated anions paves the way for the exploration of new NIR-II photothermal agents. While the concept is interesting, no other types of planar π-conjugated anions are discussed, and it is not demonstrated whether the $C_5CN_5^-$ anion can be combined with other π-conjugated cations, as the ones presented in the previous strategy in the scheme 1. Consequently, the scope of this work and its strategy is restricted to a single example, which limits its overall significance and falls short of the impact as expected for publication in such a high-impact journal. In addition, the experimental descriptions lack sufficient rigor, which may compromise the reproducibility of the work (see below).

General comments:
In Scheme 1 the corresponding references should be added for the previous strategies. In the bottom left, all the compounds have the same name.
What about the absorption, fluorescence and photothermal properties of DTPAP-2P as a raw powder?
How can the mean diameter observed by TEM be superior than the hydrodynamic diameter measured by DLS?
Why the cellular uptake was studied on HeLa cells and the phototoxicity on 4T1 cells?
For the in vivo cancer study, the nanoparticles (NPs) were administered via intratumoral injection. Could the authors clarify whether systemic administration (e.g. intravenous injection) was also considered or evaluated? In addition, since the NPs are locally injected and confined to the tumor site, is it expected that their toxicity toward other organs would be limited? Further discussion or experimental evidence regarding systemic distribution and off-target toxicity would strengthen the manuscript.
The final paragraph functions as a conclusion rather than a discussion. The authors should use “results and discussion“.

In SI:
The 13C NMR peak should attributed.
equation a, nref not nnef
In table S1, which reference was used to calculate absolute fluorescence quantum yield in DMSO? Please revised caption.
Figure S17b, where Is the second bromide atom?
Why Figure S18a and S19a are not the same? Which concentration have been used?
For the irradiation experiments, in addition of the laser power, the spot size should also be given.
Page 4, “The as-prepared NPs were concentrated and calibrated before use.“? Please explain how the exact concentration of compound was determined in the NPs suspensions after dialysis. How the molar extinction coefficient was calculated?
For the Total ROS Detection by DCFH, 50 μL DCFH (concentration: 40 μM) was mixed with the NPs solution or Ce6 (working concentration: 10 μM). Which volume of solution of NPs or Ce6 was used? The concentration of DCFH cannot be calculated. It is also mentioned that PBS has been used ? Not in figure 1. This part must be strongly revised.
Page 5, “10 μM of DTPAP-2B NPs or DTPAP-2P NPs were dissolved in 2 mL PBS containing 5 μM of DHR123.” Did the authors mean aqueous solution when using PBS? For me, aqueous solution is in pure water. Idem, here the volume of DTPAP-2B NPs or DTPAP-2P NPs is not given.

Version 1:

Reviewer comments:

Reviewer #1

(Remarks to the Author)
I have carefully reviewed the revised manuscript (NCOMMS-26-005084A) and the authors’ point-by-point response to my previous comments. I am pleased to note that the authors have addressed all the issues raised in the first round of review in a thorough and convincing manner.

Specifically, the additional data on nanoparticle size distribution and colloidal stability in biological media (Response to Comment 1), the clarified discussion distinguishing anion–π^+ interactions from general counterion effects (Response to Comment 2), and the expanded comparison with previously reported anion–π^+ photosensitizers (Response to Comment 3) have significantly strengthened the manuscript. The newly provided photothermal cycling experiments and comparison with a commercial NIR dye (Response to Comment 4), along with the complementary ROS probe assays (Response to Comment 5) and hemocompatibility evaluation (Response to Comment 6), provide solid experimental support for the conclusions and demonstrate the robustness and translational potential of the proposed system.

The revised manuscript is well organized, methodologically rigorous, and meets the high standards expected for publication in Nature Communications. The results remain highly novel and noteworthy: the strategic use of an organic planar π-conjugated anion to construct anion–π^+ type NIR-II phototheranostic agents represents an important conceptual advance over conventional approaches relying on inorganic counteranions. This work not only fills a critical gap in the development of NIR-II anion–π^+ systems but also provides valuable design principles that are likely to inspire further research in the field of organic phototheranostics.

In my judgment, the authors have satisfactorily addressed all concerns, and no further revision is required. I recommend that the manuscript be accepted for publication in its current form.

Reviewer #2

(Remarks to the Author)
The authors have properly addressed and corrected all the points raised by the reviewers. The article is now fully consistent with the claims and can be published.
Minor corrections:
Please add the storage temperature on Figure S21 and S22.
ABDA is also instead of DHR123 or HPF in the experimental descriptions. Revised.
I still do not agree with the expression “the molar concentration of NPs,” because this actually corresponds to the molar concentration of the molecular compound in the NP suspension rather than the molar concentration of nanoparticles themselves. The exact nanoparticle concentration (i.e., the exact particle count or number of nanoparticles per unit volume) has not been determined. Therefore, throughout the manuscript, the correct terminology should be “molar concentration of the molecular compound in the NP suspension.”
Consequently, the molar extinction coefficient (ε) of the nanoparticles themselves cannot be accurately determined, since the nanoparticle molar concentration is unknown.
For example, when the manuscript states “NPs in 2 mL PBS (working concentration: 50 μM),” this concentration actually refers to DTPAP-2B or DTPAP-2P in the nanoparticle suspension, not to the nanoparticles themselves.
“Notably, the maximum absorption peaks of DTPAP-2P NPs were obviously redshifted from 535 nm to 570 nm, with its molar extinction coefficient at 660 nm calculated to be 1.16 ×104 L mol-1 cm-1”, this is the molar extinction coefficient of DTPAP-2P aggregated into nanoparticles and not the one of a nanoparticle. The sentence should be "“Notably, the maximum absorption peaks of DTPAP-2P in NPs were obviously redshifted from 535 nm to 570 nm, with its molar extinction coefficient at 660 nm calculated to be 1.16 ×104 L mol-1 cm-1”

Remark:
“Thus, the final concentration of ABDA was 5 μM, and the final concentration of Ce6 or NPs in the assay system was 10 μM.” Should be “Thus, the final concentration of ABDA was ~5 μM, and the final concentration of Ce6 or NPs in the assay system was ~10 μM.” as the final concentration is 2.022 mL and not 2mL

Open Access This Peer Review File is licensed under a Creative Commons Attribution 4.0 International License, which permits use, sharing, adaptation, distribution and reproduction in any medium or format, as long as you give appropriate credit to the original author(s) and the source, provide a link to the Creative Commons license, and indicate if changes were made.
In cases where reviewers are anonymous, credit should be given to 'Anonymous Referee' and the source.
The images or other third party material in this Peer Review File are included in the article’s Creative Commons license, unless indicated otherwise in a credit line to the material. If material is not included in the article’s Creative Commons license and your intended use is not permitted by statutory regulation or exceeds the permitted use, you will need to obtain permission directly from the copyright holder.
To view a copy of this license, visit https://creativecommons.org/licenses/by/4.0/

REVIEWER COMMENTS

Reviewer #1 (Remarks to the Author):

In this manuscript, Zhang et al. propose an organic planar π-conjugated anion exchange strategy for constructing high-performance anion–π^+ type NIR-II photothermal agents (PTAs). Previous studies on anion–π^+ type phototheranostic systems have predominantly focused on inorganic counteranions. In contrast, the authors ingeniously exploit the large π-conjugation and intrinsic planarity of an organic π-conjugated anion to induce strong electrostatically driven donor–acceptor interactions and anion–π^+ interactions. This strategy effectively promotes red-shifted absorption and emission as well as enhanced emission intensity, enabling the realization of an anion–π^+ phototheranostic system with NIR-II emission. As a proof of concept, the authors synthesized two anion-π^+ type phototheranostic agents, DTPAP-2B with Br- and DTPAP-2P with an organic planar π-conjugated pentadecyanocyclopentadiene anion ($C5CN5^-$). Experimental results demonstrate that DTPAP-2P NPs exhibit a higher molar extinction coefficient (1.16×10^4 $L \cdot mol^{-1} \cdot cm^{-1}$ at 660 nm), pronounced NIR-II emission, more efficient ROS generation, and a significantly enhanced photothermal conversion efficiency (72%) compared with DTPAP-2B NPs. Overall, this manuscript is highly interesting and innovative. The study is well organized and supported by solid experimental evidence, including comprehensive structural characterization, systematic photophysical analysis, and convincing in vitro and in vivo evaluations. The conclusions are well justified by the presented data. The research content fits well within the scope of Nature Communications. Therefore, I believe this work is suitable for publication after minor revision. The following points are suggested for further clarification and strengthening of the manuscript:

Reply: We sincerely thank the reviewer for recognizing the novelty of our manuscript. We also appreciate a lot for the reviewer's precious time to review our manuscript and make professional and constructive suggestions to improve further the quality of our manuscript. We have revised the manuscript from point-to-point following your suggestions.

(1) Please provide additional details on the size distribution and stability of DTPAP-2P NPs and DTPAP-2B NPs (e.g., DLS in biological media) to support their suitability for in vivo imaging and therapy.

Reply: We thank the reviewer for this constructive suggestion. To substantiate the suitability of DTPAP-2P NPs and DTPAP-2B NPs for *in vivo* imaging and therapeutic applications, we performed a systematic evaluation of their size distribution and colloidal stability using DLS according to your suggestion. In aqueous solution, both NPs displayed narrow size distributions with uniform hydrodynamic diameters of approximately 102 nm (DTPAP-2B NPs) and 96 nm (DTPAP-2P NPs). Notably, this size range (80–120 nm) is widely regarded as favorable for enhanced permeability and EPR mediated passive tumor accumulation. We further assessed their stability in dulbecco's modified eagle medium (DMEM). The hydrodynamic diameters of both NPs remained essentially unchanged over the monitored period, indicating robust stability in complex biological environments. Taken together, these results demonstrate that both DTPAP-2P NPs and DTPAP-2B NPs maintain colloidal stability in biologically media, thereby supporting their potential for systemic administration in *in vivo* imaging and therapeutic applications.

On page 7 in the revised manuscript:

In order to improve their water solubility and biocompatibility for subsequent biological applications, the corresponding NPs of DTPAP-2B and DTPAP-2P were prepared according to a one-step nanoprecipitation strategy with amphiphilic copolymer Pluronic F127. Dynamic light scattering (DLS) results (Figure 1c) showed that DTPAP-2B NPs and DTPAP-2P NPs displayed an average hydrodynamic diameter of 102 nm and 96 nm, respectively. Images obtained through transmission electron microscopy (TEM) also verified the spherical morphology of DTPAP-2B NPs and DTPAP-2P NPs as shown in Figure S20. Then the stability of NPs at 4 °C was further monitored. As shown in Figure S21A, during a storage period of up to 7 days, the particle size of DTPAP-2B NPs and DTPAP-2P NPs remained stable at approximately 105 nm and 102 nm, which is beneficial for practical application. Additionally, the absorption and fluorescence spectra of the DTPAP-2P NPs solution remained unchanged during this peroid (Figure S21B-S21D). The stability in dulbecco's modified eagle medium (DMEM) were further assessed. The hydrodynamic diameters of both NPs remained essentially unchanged over the monitored period, indicating robust stability in complex biological environments (Figure S22).

On page S27 in the revised Supporting Information:

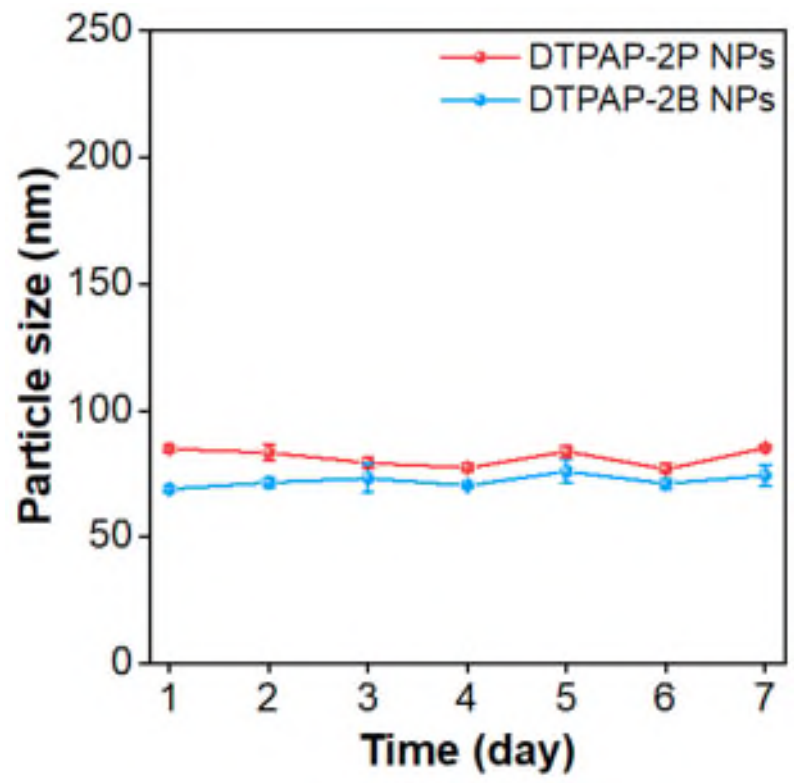

Figure S22. Size stability of DTPAP-2P NPs and DTPAP-2B NPs in dulbecco's modified eagle medium (DMEM) during 7 days of storage.

(2) The authors attribute the improved performance of DTPAP-2P mainly to anion–π^+ interactions introduced by the π-conjugated counterion. What distinguishes this interaction from general anion effects or other counterion strategies, and what are its specific advantages in this system?

Reply: We appreciate the reviewer's important question regarding the distinction between anion–π^+ interactions and general ionic or counterion effects. Anion–π^+ interactions refer to the noncovalent attraction between electron-deficient π^+–systems and anions. The anion–π^+ PSs developed in this work exhibit several distinctive advantages that make them distinguished from the general ionic or counterion effects. Firstly, the positively charged π^+ core may promote strong electrostatic interactions and directional anion–π^+ interactions with negatively charged cancer cell membranes, facilitating rapid cellular internalization and preferential accumulation in specific subcellular organelles, particularly mitochondria. Such precise subcellular localization shortens the diffusion distance between the PS and critical biomolecular targets, thereby may enhance ROS utilization efficiency to result disruption of intracellular redox homeostasis and metabolic balance. Secondly, most reported ionic AIE-active PSs rely on structural motifs such as pyridinium salts, triphenylphosphonium salts, quaternary ammonium salts, or methylquinolinium units to introduce ionic characteristics, which often involve multistep synthesis and challenging purification. Although some ionic AIE PSs exhibit NIR-II emission, they are frequently excited with broadband or white-light sources, limiting tissue penetration and compromising photodynamic efficacy. In contrast, the anion–π^+ PSs described here are synthetically more accessible. The strongly electron-withdrawing

π^+ core facilitates charge-transfer state formation, promoting spectral red-shifting toward the near-infrared region and enabling more suitable excitation conditions for deep-tissue applications.

In addition, counterions mainly contribute through nonspecific electrostatic screening or modulation of hydrophobicity in conventional ionic systems. By contrast, the π-conjugated organic anion in DTPAP-2P participates in directional anion-π^+ interactions with the electron-deficient aromatic cationic core. This interaction provides not only electrostatic stabilization but also enhanced polarization and charge-transfer coupling, which cannot be achieved with small inorganic counterions (Br^-). Consequently, the anion–π^+ interaction effectively regulates intermolecular packing in the aggregated state, suppressing detrimental π–π stacking while preserving sufficient electronic communication. This finely tuned balance enables the simultaneous enhancement of NIR-II emission, ROS generation, and photothermal conversion. Therefore, the improved performance does not arise from a generic ionic effect, but from the specific, directional, and electronically active characteristics of the anion–π^+ interaction introduced by the π-conjugated counterion.

(3) The authors state that NIR-II PTAs based on anion–π^+ interactions have not yet been successfully developed. However, the manuscript lacks a concise comparison with previously reported anion–π^+ PSs. Could the authors briefly summarize the typical absorption and emission ranges of reported anion–π^+ PSs, and clearly clarify the key advantages of DTPAP-2P NPs?

Reply: Thank you very much for your professional suggestions. Based on your suggestion, we have compared the spectra and excitation light source of DTPAP-2P NPs with those previously reported anion-π^+ dyes in Table S1.

On page 3-4 in the revised manuscript:

Moreover, most reported anion-π^+ PSs require excitation by ultraviolet or white light, both of which suffer from poor tissue penetration, thereby compromising the *in vivo* therapeutic efficacy (Table S1).

On page S16 in the revised Supporting Information:

Table S1. Comparison of parameters between previously reported anion-π^{+} PSs and DTPAP-2P in this study.

Ref.	Chemical structure	$\lambda_{ex}/\lambda_{em}$ (nm)	Light source	ROS type
S19	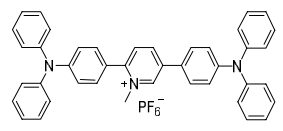	456/543 in THF	475 nm	-
S20	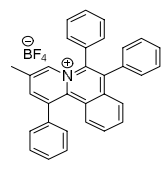	380/450 in water	365 nm	-
S21	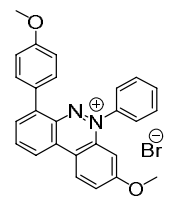	518/732 in water	white light	Type I
S22	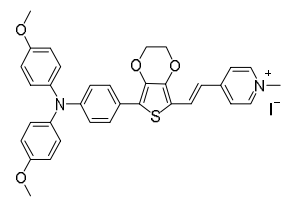	540/687 in toluene	white light	Type I
S23	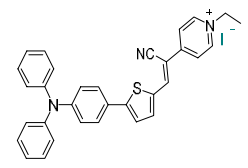	534/~725 in DMSO	white light	Type I
S24	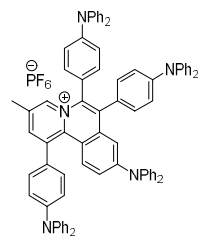	440/~496 in DMSO	white light	Type II
S25	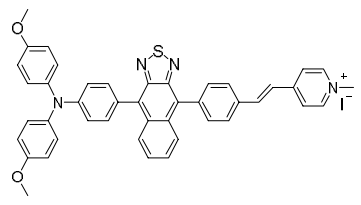	528/686 in THF	white light	Type I
S26	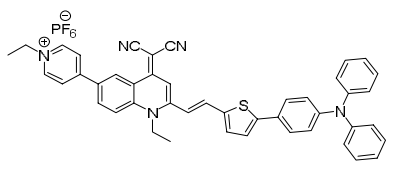	477/731 in water	white light	Type I/II
S27	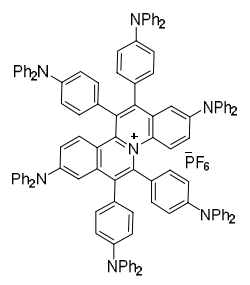	560/~650 (in THF)	white light	Type I/II
S28	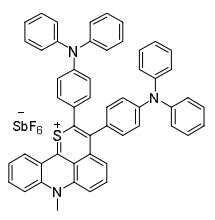	579/790 in PBS	635 nm laser	Type I
S29	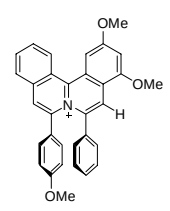	430/602 in DMSO	488 nm laser	-
S30	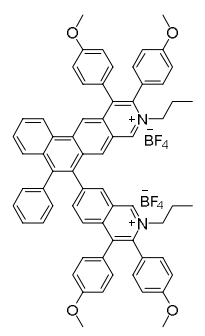	320/547 in DMSO	white light (10 mW/cm^{2})	Type I
This work	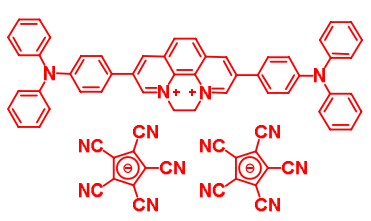	635/1000 in PBS	660 nm laser (0.4 W/cm^{2})	Type I/II

On page S47-S48 in the revised Supporting Information:

S19. Xu, Y.; Dang, D.; Zhang, N.; Zhang, J.; Xu, R.; Wang, Z.; Zhou, Y.; Zhang, H.; Liu, H.; Yang, Z.; Meng, L.; Lam, J. W. Y.; Tang, B. Z., *ACS Nano* **2022**, 16, 5932-5942.

S20. Li, Q.; Gong, J.; Li, Y.; Zhang, R.; Wang, H.; Zhang, J.; Yan, H.; Lam, J. W. Y.; Sung, H. H. Y.; Williams, I. D.; Kwok, R. T. K.; Li, M.-H.; Wang, J.; Tang, B. Z., *Chem. Sci.* **2021**, 12, 709−717.

S21. Liu, L.; Li, C.; Gong, J.; Zhang, Y.; Ji, W.; Feng, L.; Jiang, G.; Wang, J.; Tang, B. Z., *Angew. Chem., Int. Ed.* **2023**, 62, e202307776.

S22. Wang, Y.; Zhang, C.; Li, Y.; Li, R.; Li, J.; Feng, L., *Dyes Pigm.* **2023**, 218, 111517.

S23. Xiao, P.; Shen, Z.; Wang, D.; Pan, Y.; Li, Y.; Gong, J.; Wang, L.; Wang, D.; Tang, B. Z., *Adv. Sci.* **2022**, 9, 2104079.

S24. Li, Q.; Li, Y.; Min, T.; Gong, J.; Du, L.; Phillips, D. L.; Liu, J.; Lam, J. W. Y.; Sung, H. H. Y.; Williams, I. D.; Kwok, R. T. K.; Ho, C. L.; Li, K.; Wang, J.; Tang, B. Z., *Angew. Chem., Int. Ed.* **2020**, 59, 9470-9477.

S25. Wan, Q.; Zhang, R.; Zhuang, Z.; Li, Y.; Huang, Y.; Wang, Z.; Zhang, W.; Hou, J.; Tang, B. Z., *Adv. Funct. Mater.* **2024**, 34, 2312870.

S26. Wang, Y.; Liao, J.; Lyu, Y.; Guo, Q.; Zhu, Z.; Wu, X.; Yu, J.; Wang, Q.; Zhu, W.-H., *Adv. Funct. Mater.* **2023**, 33, 2301692.

S27. Yang, S.; Yu, H.; Liu, J.; Ma, L.; Hou, Z.; Ma, J.; Miao, M. Z.; Kwok, R. T. K.; Sun, J.; Sung, H. H. Y.; Williams, I. D.; Lam, J. W. Y.; Liu, X.; Tang, B. Z., *ACS Nano* **2023**, 17, 21182-21194.

S28. Liu, L.; Gong, J.; Wang, X.; Zhang, Y.; Jiang, G.; Wang, J.; Tang, B. Z., *Aggregate* **2026**, 7, e70267.

S29. Mule, R. D.; Roy, R.; Mandal, K.; Chopra, D.; Dutta, T.; Sancheti, S. P.; Shinde, P. S.; Banerjee, S.; Koner, A. L.; Bhowal, R.; Senthilkumar, B.; Patil, N. T., *Chem. Eur. J.* **2022**, 28, e202200632.

S30. Chen, K.; He, P.; Wang, Z.; Tang B. Z., *ACS Nano* **2021**, 15, 7735-7743.

(4) Given the potential application of DTPAP-2P NPs in long-term NIR-II imaging and phototherapy, it would be helpful to evaluate their photothermal stability under repeated laser

irradiation. Including a comparison with a representative commercial NIR dye would further clarify the practical advantages of DTPAP-2P NPs.

Reply: We sincerely thank the reviewer for this insightful suggestion. As you suggested, we compared the photothermal stability of DTPAP-2P NPs under repeated laser irradiation using indocyanine green (ICG) as a representative commercial NIR dye for comparison. As shown in Figure 2h, over five consecutive laser on/off cycles, the temperature profiles of DTPAP-2P NPs exhibit negligible attenuation, indicating excellent photothermal stability. In contrast, under identical experimental conditions, ICG shows a gradual decrease in temperature increment with each successive cycle. The results show that DTPAP-2P NPs exhibit significantly improved photothermal stability compared to ICG, highlighting their suitability for long-term NIR-II imaging and therapeutic applications.

On page 11-12 in the revised manuscript:

In addition, the photothermal stability of DTPAP-2P NPs and DTPAP-2B NPs was evaluated with the FDA-approved clinical fluorescent dye ICG as a comparison. The samples were subjected to five heating–cooling cycles under the consecutive irradiation of a 660 nm laser (0.4 W cm^{-2}). During repeated irradiation, the maximum temperature elevation of ICG gradually decreased from 56 °C to 47 °C, reflecting its intrinsic photothermal instability. In contrast, both DTPAP-2B NPs and DTPAP-2P NPs maintained nearly constant temperature profiles throughout the five on/off cycles, demonstrating their superior photothermal stability under repeated laser exposure (Figure 2h).

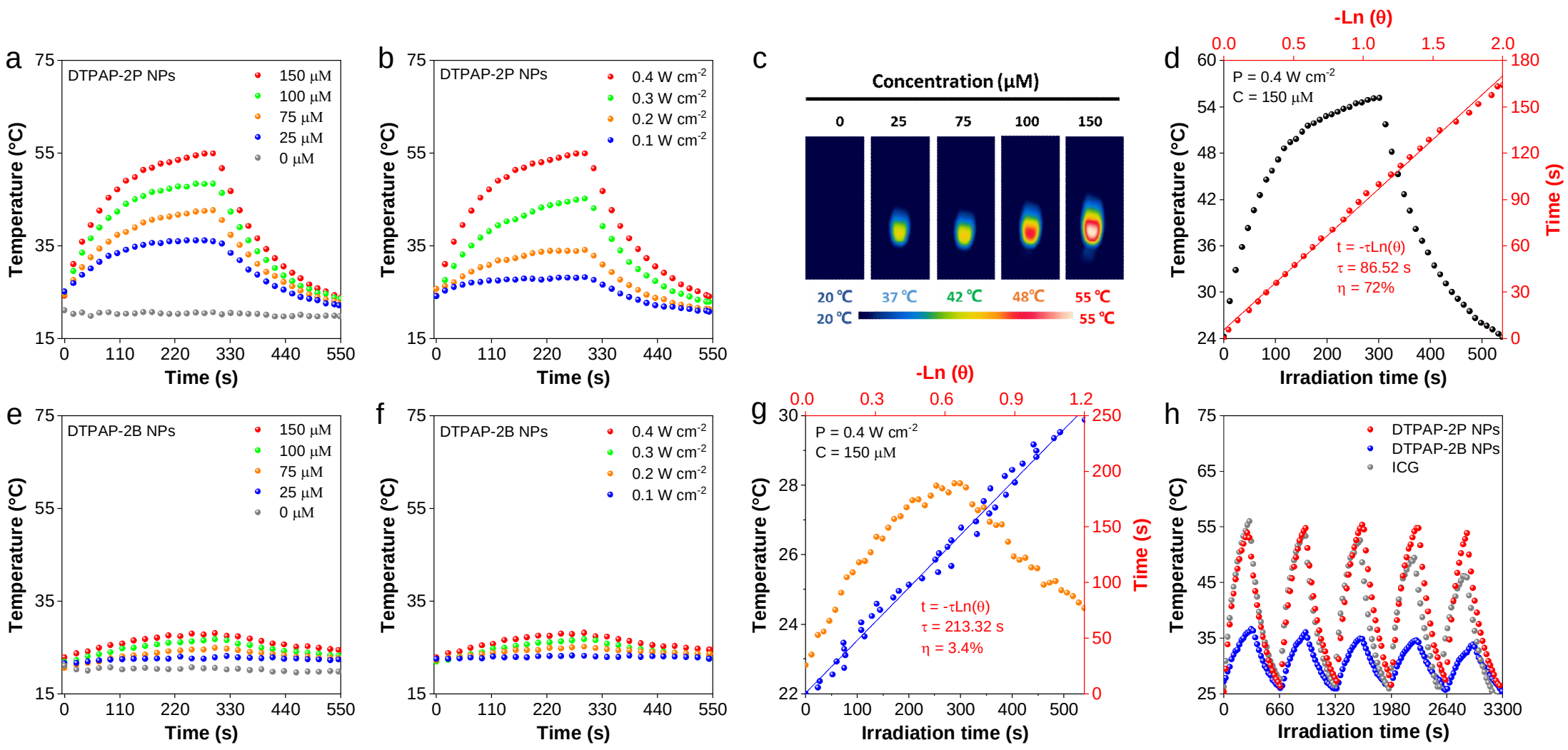

Fig. 2. Photothermal properties of DTPAP-2P NPs and DTPAP-2B NPs. The concentration-dependent (a) and laser power-dependent (b) temperature elevation curves of DTPAP-2P NPs upon 660 nm laser irradiation. (c) Infrared thermal images of various concentrations of DTPAP-2P NPs under 660 nm laser irradiation (0.4 W cm^{-2}). (d) Temperature increasing/decreasing curve and cooling time vs -ln(θ) plot (θ: temperature driving force) of DTPAP-2P NPs. The concentration-dependent (e) and laser power-dependent (f) temperature elevation curves of DTPAP-2B NPs upon 660 nm laser irradiation. (g) Temperature increasing/decreasing curve and cooling time vs -ln(θ) plot of DTPAP-2B NPs. (h) Photothermal stability of DTPAP-2P NPs, DTPAP-2B NPs and ICG during 5 cycles of heating-cooling.

(5) The authors are encouraged to include comparative cellular imaging using additional ROS probes to further validate the type and intracellular generation of ROS induced by the photosensitizer.

Reply: We sincerely thank the reviewer for this valuable suggestion. In response, we have conducted supplementary cellular imaging experiments using complementary ROS-sensitive probes (HPF for •OH, DHE for $O_2^{\bullet-}$) under identical irradiation conditions. Upon light exposure in the presence of the photosensitizer, both probes exhibited markedly enhanced fluorescence signals, whereas negligible changes were observed in the control groups (laser light only or probe only), confirming light-triggered intracellular ROS generation. Importantly, the selective response of specific probes supports the predominance of a type I ROS generation pathway, which is fully consistent with the ROS characterization results obtained in PBS.

On page 17 in the revised manuscript:

Then, dihydroethidium (DHE) and aminophenyl fluorescein (APF) were used as fluorescent probes for $O_2^{\bullet-}$ and •OH to evaluate the intracellular production of type I ROS by DTPAP-2P NPs and DTPAP-2B NPs. As expected, the cells showed enhanced fluorescence signals within the cells in the presence of both NPs and laser light irradiation, suggesting that both NPs allow type I ROS generation inside cells. Importantly, after 5 min of irradiation, DTPAP-2P NPs induced brighter red and green signals compared to DTPAP-2B NPs under identical conditions (Figure S52), suggesting that DTPAP-2P NPs possess a superior capacity for intracellular type I ROS generation. This trend

is fully consistent with the ROS generation profiles measured in PBS, further supporting the enhanced photodynamic activity of DTPAP-2P NPs. These data revealed that DTPAP-2P NPs can not only serve as photosensitizers for real-time and in situ imaging of cancer cells, but also show enormous potential for phototherapy in cancer treatment.

On page S8-S9 in the revised Supporting Information:

For the total ROS detection, HeLa cells were respectively incubated with 10 μM of DTPAP-2B NPs or DTPAP-2P NPs for 8 h followed by incubation with 20 μM DCFH-DA for 30 min. After being washed by PBS for three times, cells were irradiated by white light at a power density of 10 mW cm^{-2} for 1 min. Then, the fluorescence was immediately observed using an inverted fluorescent microscope with the excitation wavelength of 426-466 nm, and emission collection wavelength was 500-550 nm. For •OH detection, HeLa cells were incubated with 10 μM of DTPAP-2B NPs or DTPAP-2P NPs for 8 h followed by incubation with 10 μM APF for another 30 min. After being washed by PBS for three times, cells were irradiated by 660 nm laser (0.1 W cm^{-2}) for different times. Then, the green fluorescence was immediately observed using an inverted fluorescent microscope with the excitation wavelength of 426-466 nm. Emissions at 500-550 nm were recorded. For $O_2^{\bullet-}$ detection, HeLa cells were incubated with 10 μM of DTPAP-2B NPs or DTPAP-2P NPs for 8 h, followed by incubation with 10 μM DHE for another 30 min. After being washed by PBS for three times, cells were irradiated by 660 nm laser (0.1 W cm^{-2}) for different times. Then the cells were immediately observed using an inverted fluorescent microscope with the excitation wavelength of 511-551 nm, and emission collection wavelength. Emissions at 573-613 nm were collected.

On page S41 in the revised Supporting Information:

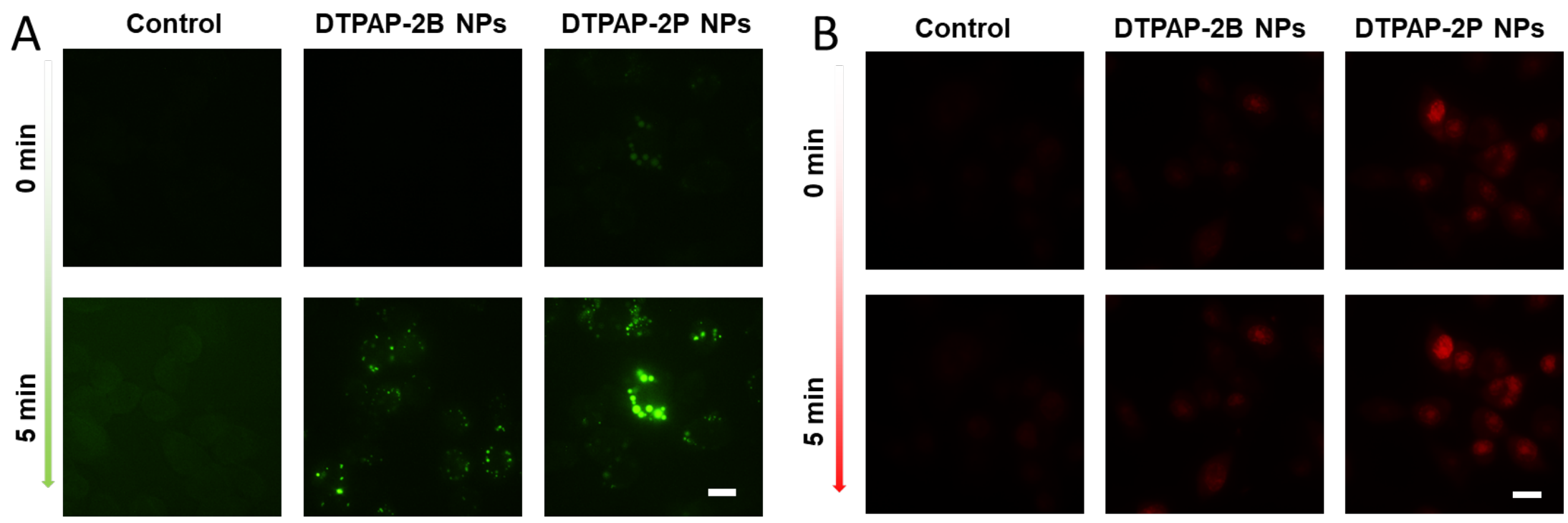

Figure S52. Imaging of type I ROS using (A) APF and (B) DHE as indicators respectively with 660 nm irradiation for different times (0.1 W cm^{-2}). Scale bar = 20 μm.

(6) The evaluation of biological safety of new system is very important. The hemocompatibility of DTPAP-2P NPs should be performed.

Reply: Thank you very much for your professional and valuable comments. Based on your suggestion, the hemocompatibility of DTPAP-2P NPs was evaluated to further confirm their biosafety. DTPAP-2P NPs exhibited a hemolysis rate of less than 5% at 150 μM, providing additional evidence of their biosafety. The relevant discussion has been incorporated into the revised manuscript.

On page 22 in the revised manuscript:

Furthermore, the hemolysis analysis suggested that DTPAP-2P NPs had excellent hemocompatibility even at a high concentration of 150 μM, providing additional evidence of their biosafety (Figure S59).

On page S44 in the revised Supporting Information:

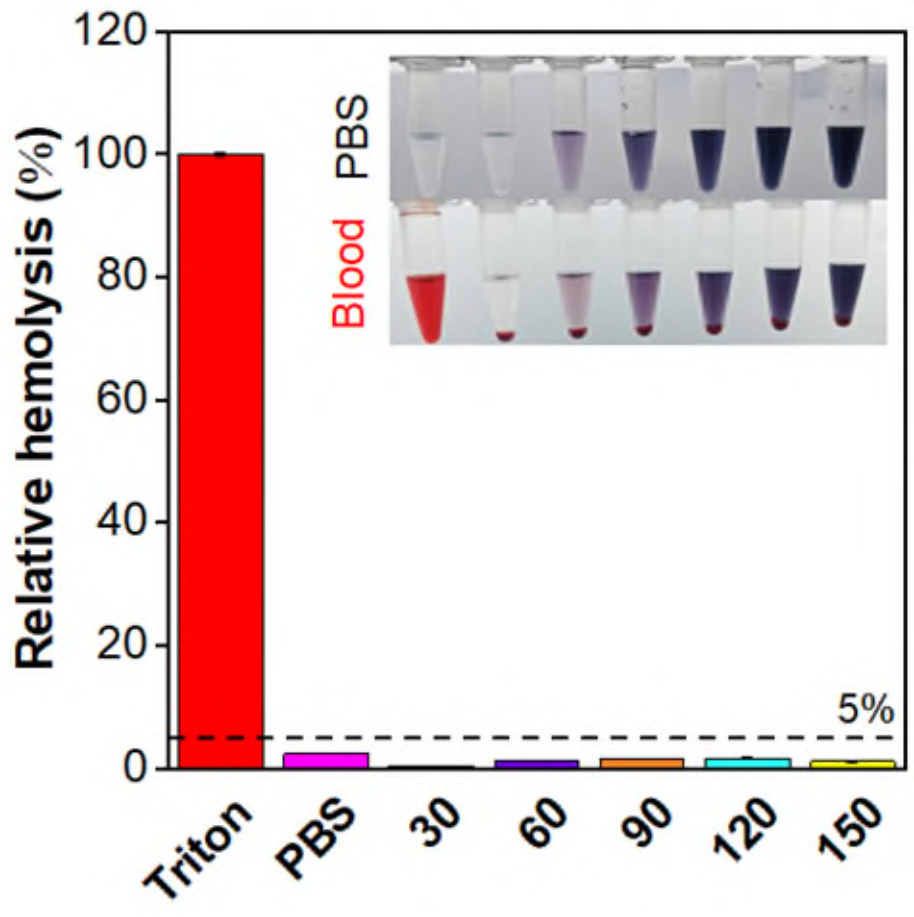

Figure S59. Hemolysis rates of DTPAP-2P NPs. Triton and PBS were used as the positive control and negative control, respectively. Inset: the photographs of hemolytic activity.

On page S10-S11 in the revised Supporting Information:

Hemocompatibility test

Whole blood was collected from healthy mice (2000 rpm, 10 min). PBS was used to wash the obtained erythrocytes for three times. Subsequently, 25 μL of blood cells were treated with varying concentrations of DTPAP-2P NPs (0, 30, 60, 90, 120, 150 μM) in polyethylene pipe containing 500 μL of PBS, and incubated for 2 h at 37 °C. PBS and 1% Triton X-100 (Triton) were used as negative control and positive control, respectively. Then, all samples were centrifuged for 10 min, and images were captured. The absorbance of the supernatant was measured at 540 nm. To correct for compound absorbance, DTPAP-2P NPs in PBS solution with the same concentration was used as the blank group. Hemolysis was calculated using the following formula (i):

$$\text{Hemolysis (\%)} = \frac{A_{Blood} - A_{Sample}}{A_{Triton} - A_{PBS}} \times 100\% \qquad \text{(i)}$$

Reviewer #2 (Remarks to the Author):

In the present work, J. Wang and his co-worker, reports on how the association of a pi-conjugated cation with a planar pi-conjugated anion can enhance the absorption and fluorescence properties in the NIR region in the solid state in the form of nanoparticles as well as the ROS production and photothermal activity. This nanoparticulate system efficiently enable high-resolution NIR-II angiography and effective NIR-II imaging-guided synergistic photodynamic/photothermal therapy. This work is original in the context of new fluorescent probe and photothermal agents, is well referenced, and the results obtained support the stated conclusions and claims. However, the authors state that the introduction of planar π-conjugated anions paves the way for the exploration of new NIR-II photothermal agents. While the concept is interesting, no other types of planar π-conjugated anions are discussed, and it is not demonstrated whether the $C_5CN_5^-$ anion can be combined with other π-conjugated cations, as the ones presented in the previous strategy in the scheme 1. Consequently, the scope of this work and its strategy is restricted to a single example, which limits its overall significance and falls short of the impact as expected for publication in such a high-impact journal. In addition, the experimental descriptions lack sufficient rigor, which may compromise the reproducibility of the work (see below).

Reply: We sincerely thank the reviewer for the careful evaluation of our work and for recognizing its originality and the validity of the conclusions. We also appreciate the constructive concerns regarding the generality of the strategy and the rigor of the experimental description.

Regarding the scope of the planar π-conjugated anion strategy, we agree that the present study primarily focuses on $(CCN)_5^-$ as a representative example. To demonstrate the generality of this strategy, as suggested by the reviewer, we have experimentally extended it to other π-conjugated cationic scaffolds. Specifically, we synthesized two new compounds, DMPHL-2B and DMPHL-2P, with Br^- and $(CCN)_5^-$ as the counterions, respectively (synthetic details can be found in the revised supporting information). As displayed in Figures S36-S41 and Figure S45, DMPHL-2P NPs with $(CCN)_5^-$ as the counterion exhibited redshifted absorption spectra, enhanced NIR-II emission and ROS generation, as well as remarkably improved PCE compared to DMPHL-2B NPs. These results further support that anion–π^+ interaction can similarly regulate aggregation behaviors and photophysical properties across different π-conjugated cationic systems. We have incorporated

these data and discussed how this design principle can be extended to other electron-deficient π-conjugated cationic frameworks.

Regarding attempts to identify other types of planar π-conjugated anions, we have experimentally explored candidates such as cyanurate and isocyanurate anions. However, upon combining these anions with our π-conjugated cationic scaffolds, the resulting products exhibited poor stability and underwent rapid degradation under ambient conditions, making them unsuitable for further characterization or application. To date, we have not identified other planar π-conjugated anions that combine sufficient chemical stability with the desired electronic properties for this strategy. We acknowledge that this remains an important direction for future work. Therefore, in the revised manuscript, we have more clearly positioned the current contribution as the demonstration and mechanistic validation of an anion–π^+ interaction-driven photophysical regulation principle, with the goal of inspiring further exploration of additional anion/cation combinations.

With respect to experimental rigor and reproducibility, we fully agree that detailed methodological descriptions are essential. Accordingly, we have carefully revised the Experimental Section and Supporting Information to provide more comprehensive and detailed information, including concentration calibration procedures, instrumental parameters, irradiation conditions, calibration methods, and the number of independent replicates. Ambiguities in figure captions and structural representations have also been corrected to improve clarity and reproducibility.

We sincerely appreciate the reviewer's critical insights. These comments have helped us refine the positioning of our work, clarify its scope, and substantially strengthen the methodological transparency and scientific rigor of the manuscript. We have therefore undertaken a thorough revision, preparing a detailed point-by-point response to each comment. We hope this revised version effectively addresses all of the reviewer's concerns. Thank you again for your time and effort in providing such a detailed assessment.

On page 5 in the revised manuscript:

Firstly, as shown in Scheme S1, compound DTPAP with a D-A-D structure was synthesized according to our previous reports.[65-66] Further reaction with 1,2-dibromoethane gave DTPAP-2B with Br^- as the counterions.[58] Afterward, DTPAP-2P was obtained by exchanging Br^- of DTPAP-2B with organic planar π-conjugated $(CCN)_5^-$. Similarly, DMPHL-2P and DMPHL-2B were also

synthesized as control systems to systematically verify the universality and robustness of this design strategy (Scheme S2-S4). The synthetic details and characterization data were shown in the Supporting Information.

On page 10 in the revised manuscript:

To validate the generality of this strategy, DMPHL-2P and DMPHL-2B were encapsulated with Pluronic F127 to form nanoparticles. DLS analysis showed average hydrodynamic diameters of 160 nm for DMPHL-2P NPs and 138 nm for DMPHL-2B NPs (Figure S36A). Both NPs exhibited broad absorption from 500 to 1000 nm, with maxima at 750 nm for DMPHL-2P NPs and 620 nm for DMPHL-2B NPs (Figure S36B). Compared with the DTPAP system, the DMPHL framework displayed a clear bathochromic shift, which was further enhanced upon exchange with the planar π-conjugated $(CCN)_5^-$ anion. Both NPs emitted in the NIR-II region with maxima around 1000 nm, while DMPHL-2P NPs showed stronger fluorescence intensity (Figure S36C), indicating suppressed intermolecular π–π stacking due to reinforced anion–π^+ interactions. ROS evaluation revealed that both NPs generated negligible ROS in the molecularly dissolved state (Figure S36D). In contrast, type I ROS was predominantly produced by NPs, with a negligible contribution from type II ROS. Notably, DMPHL-2P NPs exhibited significantly enhanced type I ROS generation compared with DMPHL-2B NPs (Figure S36E-S36H and Figure S37-S41). These results confirm that simple substitution with a planar π-conjugated $(CCN)_5^-$ anion is sufficient to construct NIR-II photothermal agents with reinforced anion–π^+ interactions and efficient type I ROS generation.

On page 11-12 in the revised manuscript:

The photothermal properties of DMPHL-2P NPs and DMPHL-2B NPs were also systematically investigated under 808 nm laser irradiation. As shown in Figures S45, pure aqueous solution exhibited negligible temperature variation upon 808 nm irradiation, whereas the temperature elevation of DMPHL-2P NPs displayed a clear dependence on both the concentration and the power density of 808 nm laser. Upon exposure to an 808 nm laser at 0.3 W cm^{-2} for 5 min, the temperature of the DMPHL-2P NPs solution increased to 58 °C (Figure S45A-S45C). The PCE of DMPHL-2P NPs was further determined to be 78.8% (Figure S45D), confirming their highly efficient photothermal conversion capability. Under identical conditions, the DMPHL-2B NPs solution

induced only a temperature increase to 40 °C, with a remarkably lower PCE of 18.8% (Figure S45E-S45H). Collectively, these results demonstrate the superior photothermal performance of DMPHL-2P NPs. The improved PCE as well as the redshifted absorption spectra, enhanced NIR-II emission and ROS generation demonstrated above underscores the effectiveness and generality of introducing planar π-conjugated organic anions as a straightforward strategy to construct high-performance anion–π^+ type NIR-II photothermal agents.

On page S33-S37 in the revised Supporting Information:

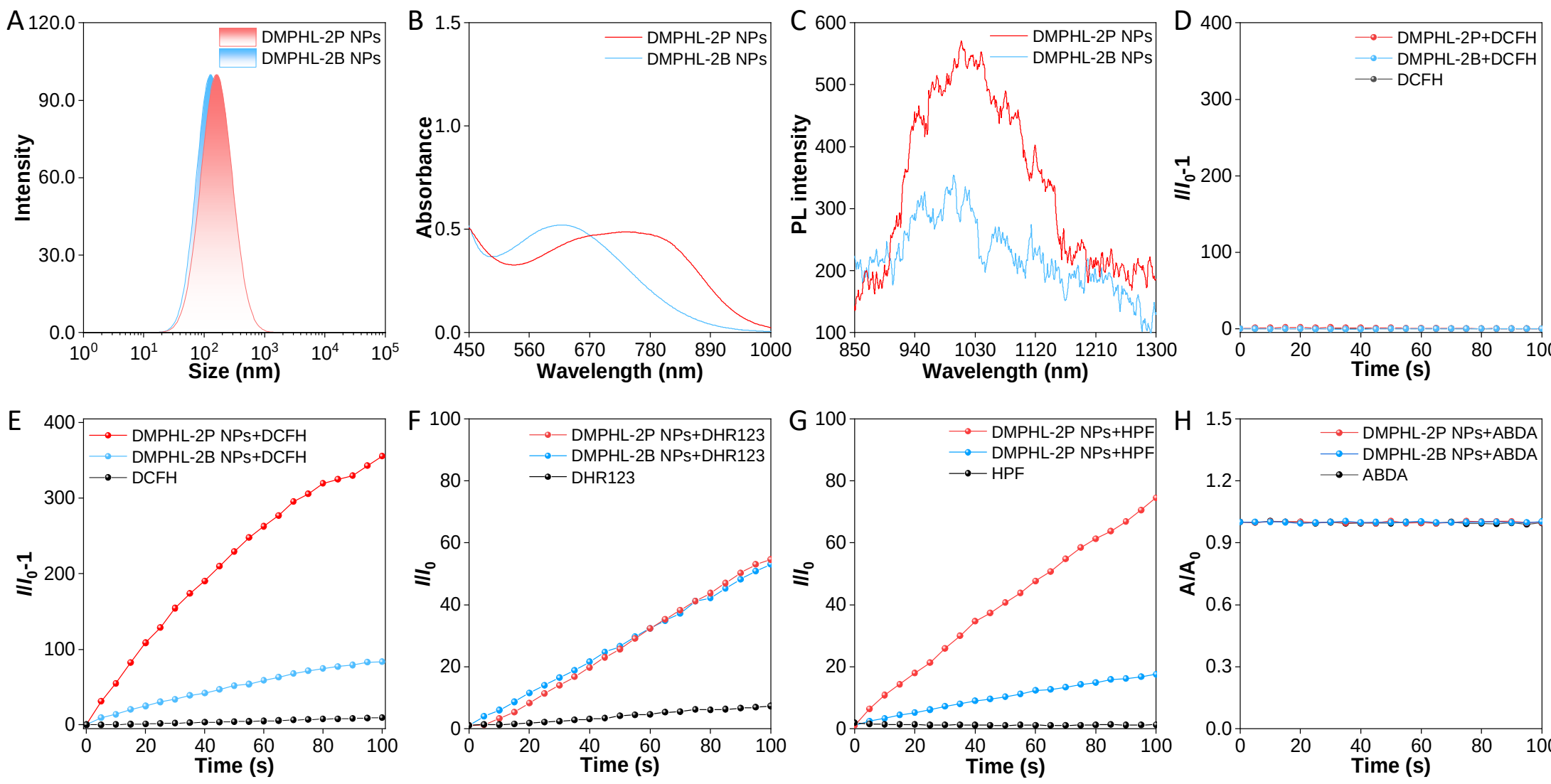

Figure S36. (A) DLS of DMPHL-2P NPs and DMPHL-2B NPs in aqueous solution. (B) Absorption spectra and (C) Photoluminescence (PL) spectra of DMPHL-2P NPs and DMPHL-2B NPs in PBS. λ_{ex}=808 nm. (D) ROS generation of DMPHL-2P NPs and DMPHL-2B NPs (10 µM) in DMSO detected by DCFH upon 808 nm laser irradiation (0.1 W cm^{-2}). (E) ROS generation of DMPHL-2P NPs and DMPHL-2B NPs (10 µM) in PBS detected by DCFH upon 808 nm laser irradiation (0.1 W cm^{-2}). Type I radical ROS generation of DMPHL-2P NPs and DMPHL-2B NPs (10 µM) in PBS detected by (F) DHR123 and (G) HPF upon 808 nm laser irradiation (0.1 W cm^{-2}). (H) $^1O_2$ generation of DMPHL-2P NPs and DMPHL-2B NPs in PBS detected by ABDA upon 808 nm laser irradiation (0.1 W cm^{-2}).

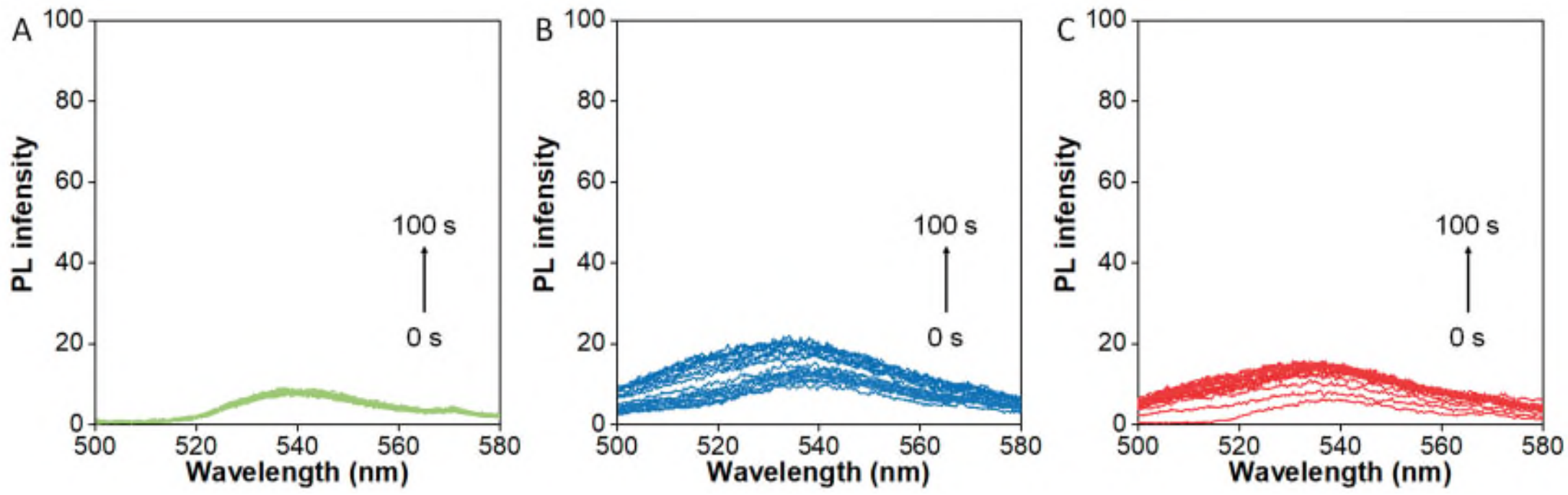

Figure S37. Fluorescence spectra changes of DCFH alone (A) after 808 nm laser irradiation. Fluorescence spectra changes of DCFH induced by DM PHL-2B (B) and DMPHL-2P (C) in DMSO solution under the 808 nm laser irradiation (0.1 W cm^{-2}). The concentration of DCFH was 1 μM and the concentration of compounds were 10 μM, respectively.

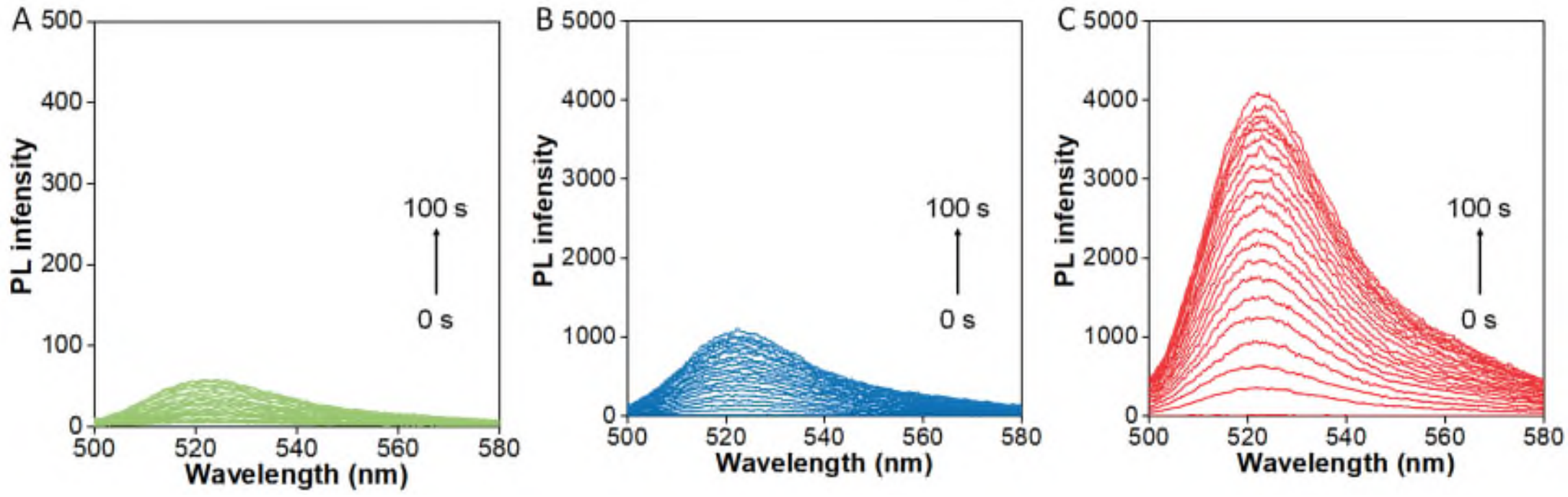

Figure S38. Fluorescence spectra changes of DCFH alone (A) after 808 nm laser irradiation. Fluorescence spectra changes of DCFH induced by DMPHL-2B NPs (B) and DMPHL-2P NPs (C) in PBS (pH 7.4) under the 808 nm laser irradiation (0.1 W cm^{-2}). The concentration of DCFH was 1 μM and the concentration of NPs were 10 μM, respectively.

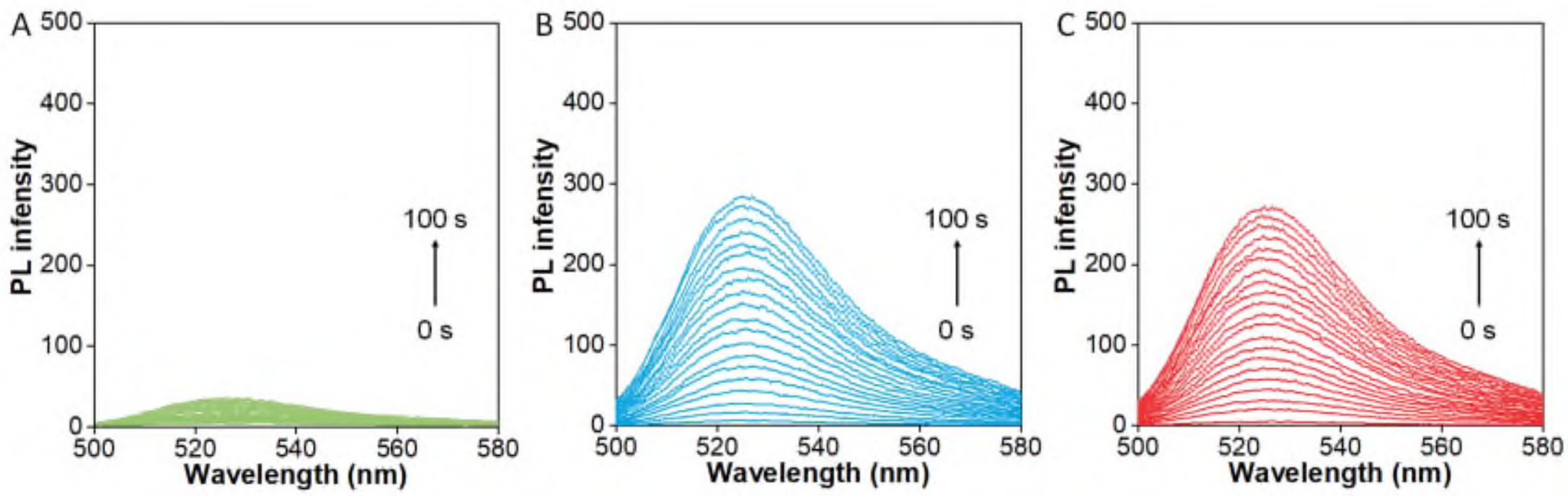

Figure S39. Fluorescence spectra changes of DHR123 alone (A) after the 808 nm laser irradiation. Fluorescence spectra changes of DHR123 induced by DMPHL-2B NPs (B) and DMPHL-2P NPs (C) in PBS (pH 7.4) under the 808 nm laser irradiation (0.1 W cm^{-2}). The concentration of DHR123 was 5 μM and the concentration of NPs were 10 μM, respectively.

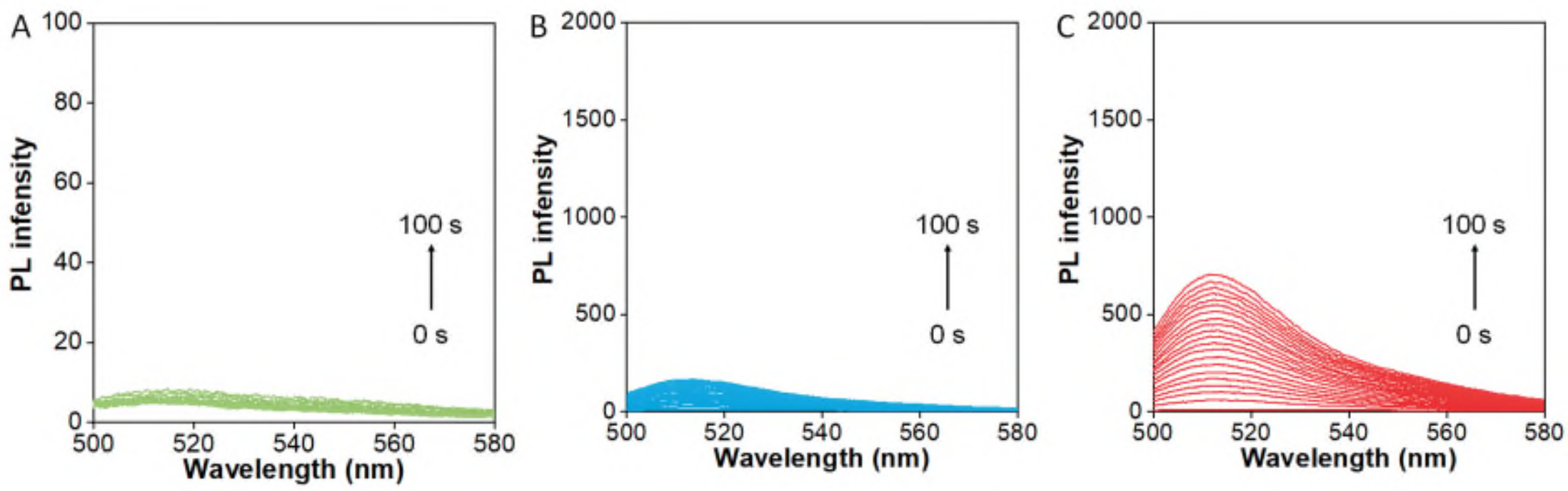

Figure S40. Fluorescence spectra changes of HPF alone (A) after the 808 nm laser irradiation. Fluorescence spectra changes of HPF induced by DMPHL-2B NPs (B) and DMPHL-2P NPs (C) in PBS under the 808 nm laser irradiation (0.1 W cm^{-2}). The concentration of HPF was 5 μM and the concentration of NPs were 10 μM, respectively.

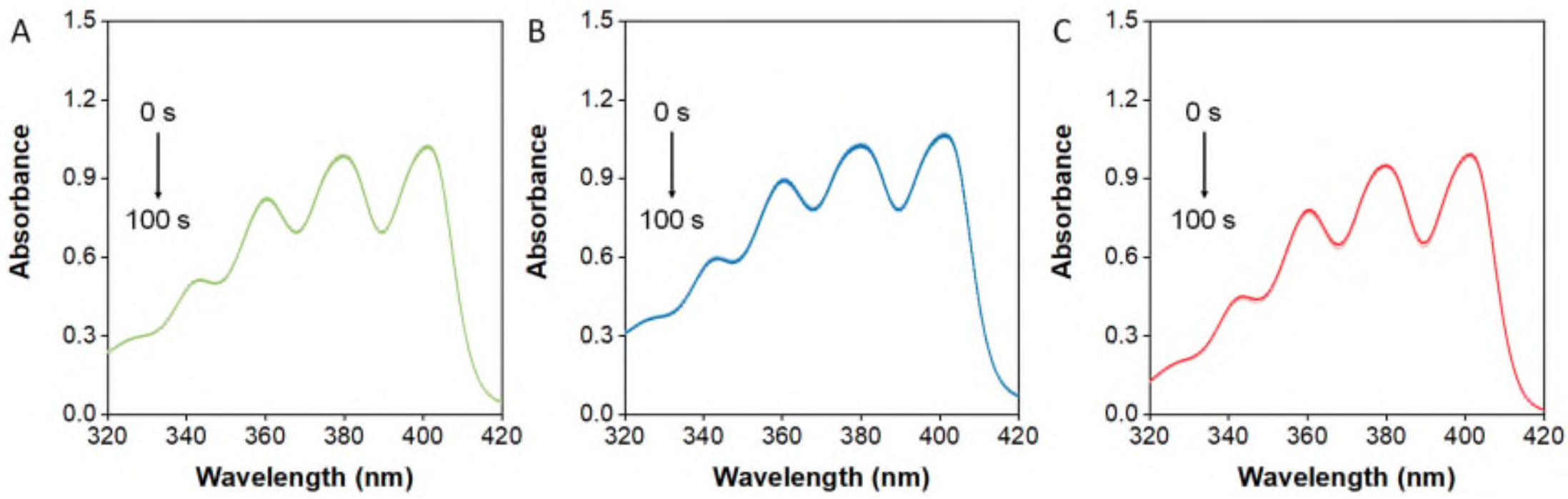

Figure S41. Absorption spectra changes of ABDA alone (A) after 808 nm laser irradiation. Absorption spectra changes of ABDA induced by DMPHL-2B NPs (B) and DMPHL-2P NPs (C) in PBS (pH 7.4) under the 808 nm laser irradiation (0.1 W cm^{-2}). The conentration of ABDA was 50 μM and the concentration of NPs were 10 μM, respectively.

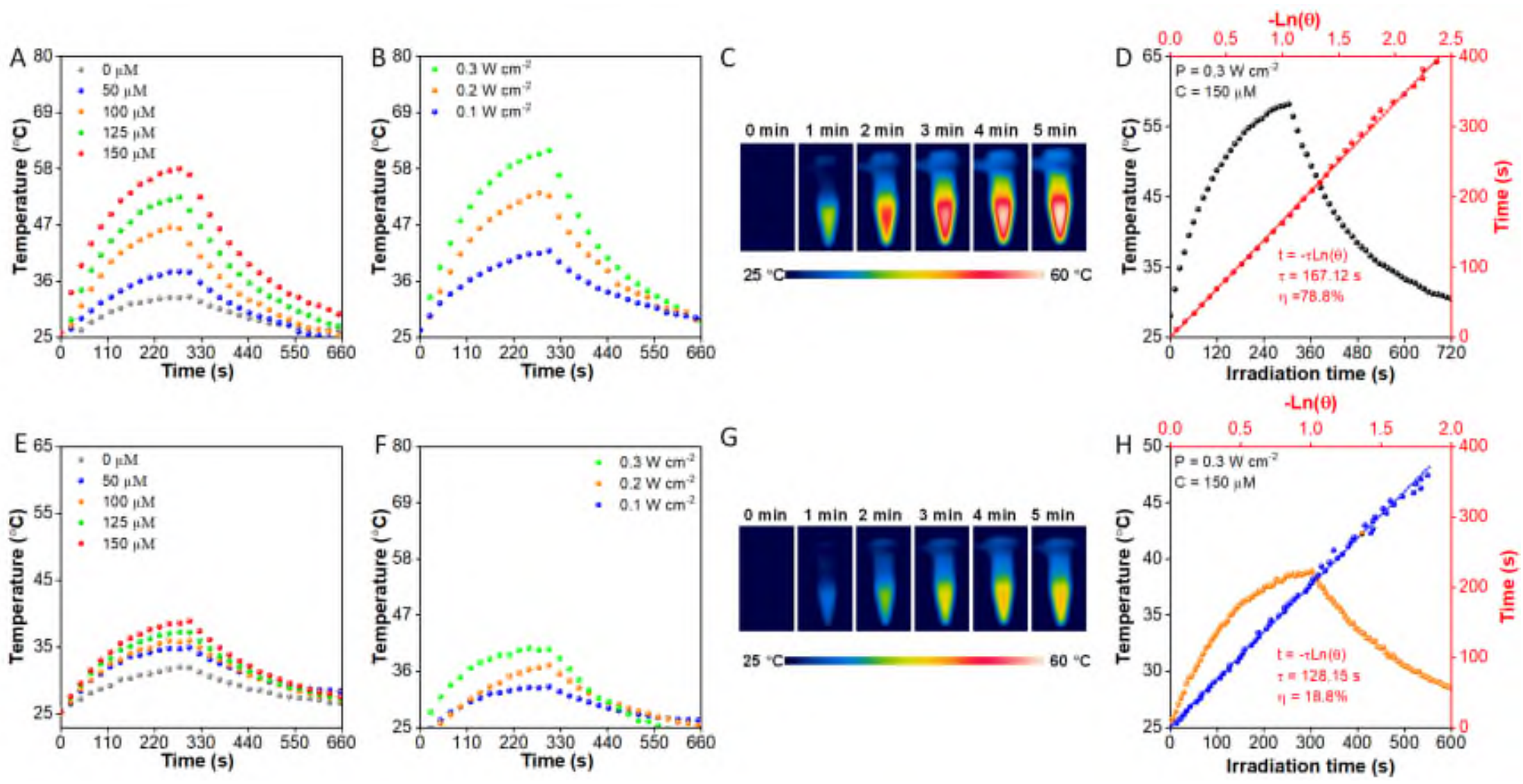

Figure S45. The concentration-dependent (A) and laser power-dependent (B) temperature elevation curves of DMPHL-2P NPs upon 808 nm laser irradiation. Infrared thermal images of DMPHL-2P NPs (C) and DMPHL-2B NPs (G) under 808 nm laser irradiation (0.3 W cm^{-2}) for different times. (D) Temperature increasing/decreasing curve and cooling time vs -ln(θ) plot (θ: temperature driving force) of DMPHL-2P NPs. The concentration-dependent (E) and laser power-dependent (F) temperature elevation curves of DMPHL-2B NPs upon 808 nm laser irradiation. (H) Temperature increasing/decreasing curve and cooling time vs -ln(θ) plot of DMPHL-2B NPs.

(1) General comments:

In Scheme 1 the corresponding references should be added for the previous strategies. In the bottom left, all the compounds have the same name.

Reply: We thank the reviewer for the careful reading and for pointing out these issues. We have added the related references to the corresponding reaction schemes in the Scheme 1. We also feel very sorry for mistakenly labelling the compound in the bottom left with the same name, and we have now corrected this.

On page 5 in the revised manuscript:

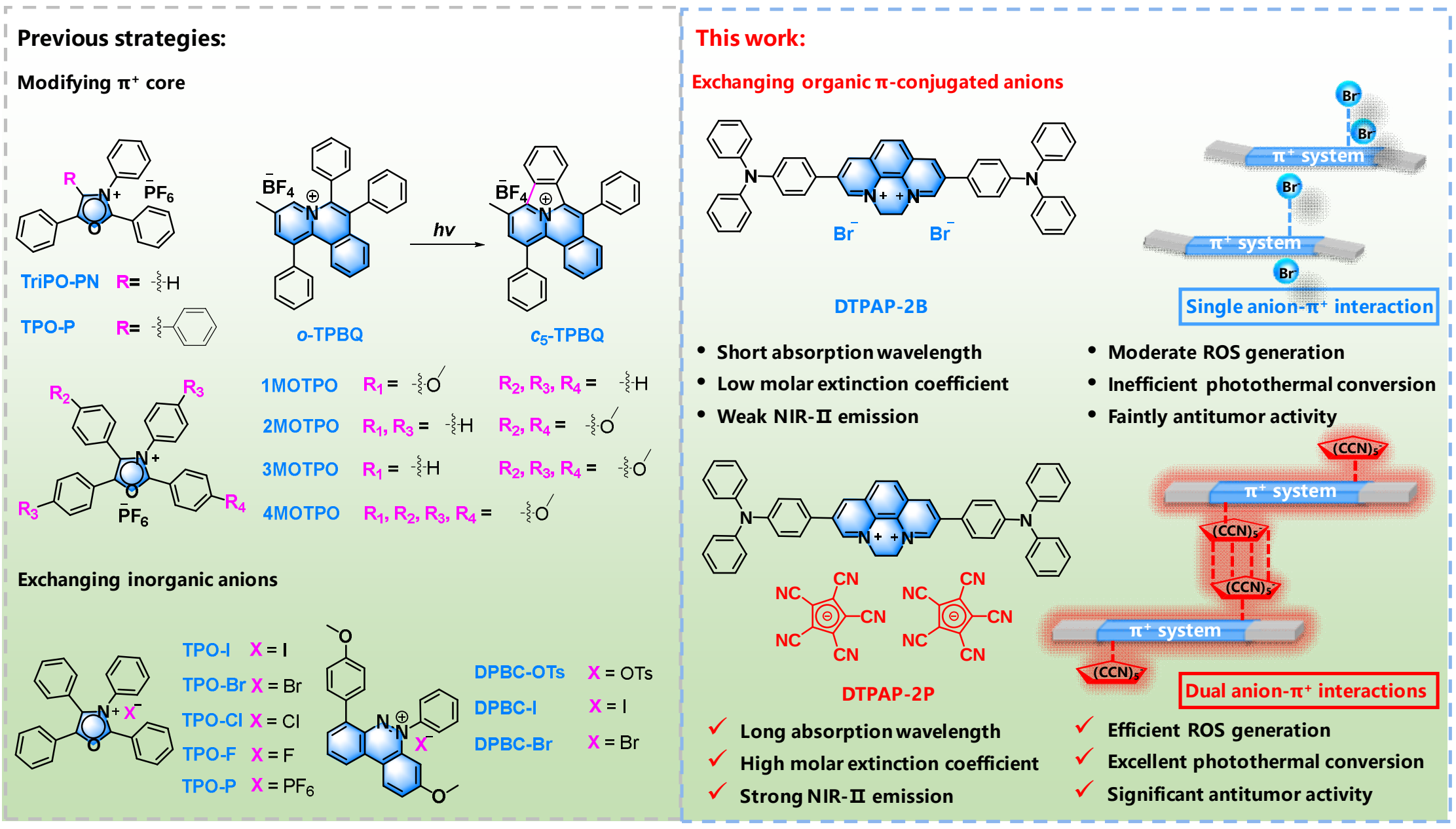

Scheme 1. Schematic illustration of organic planar π-conjugated anion exchange strategy for structural design of anion-π^+ type NIR-II PTAs.[47-48, 52-53, 59]

(2) What about the absorption, fluorescence and photothermal properties of DTPAP-2P as a raw powder?

Reply: We thank the reviewer for this insightful question regarding the solid-state properties of DTPAP-2P. Accordingly, we measured the solid-state absorption spectra of DTPAP-2P and DTPAP-2B powder using a UV-Vis spectrophotometer equipped with an integrating sphere. As shown in Figure S19, both powders exhibit a broad absorption band ranging from 450 nm to 1050 nm, with a maximum peak at approximately 660 nm and 575 nm for DTPAP-2P and DTPAP-2B, respectively. Compared to their solution-state spectra, the solid-state spectra are broadened and slightly redshifted, which can be attributed to enhanced anion–π^+ interactions and electronic coupling in the solid state. Strikingly, in contrast to DTPAP-2B powder which exhibits only very weak emission, DTPAP-2P powder displays bright NIR-II fluorescence under the same conditions. This difference likely arises from the stronger anion-π^+ interactions in DTPAP-2P solid, which effectively disrupt detrimental intermolecular π-π stacking, suppress non-radiative decay pathways, and enhance the radiative transition efficiency.

We also evaluated the photothermal effect of DTPAP-2P and DTPAP-2B powders under 660 nm irradiation (0.4 W·cm^{-2}). As shown in Figure S43, DTPAP-2P powder exhibits a rapid temperature increase, reaching a plateau with a maximum temperature rise of approximately 74 °C, whereas

DTPAP-2B powder shows a much smaller temperature increase ($\Delta T \approx 45$ °C) under identical conditions. The significantly higher photothermal response of DTPAP-2P powder confirms that the anion–π^+ interactions not only enhance solid-state NIR-II emission but also facilitate efficient non-radiative decay for photothermal conversion. These results indicate that DTPAP-2P retains its broadband absorption and effective light-to-heat conversion capability even in the solid state. We have added these results and related discussion to the revised manuscript.

On page 6-7 in the revised manuscript:

As shown in Figure S18, PL intensity of DTPAP-2P was gradually enhanced with the increase of f_w, indicating typical AIE characteristics. Meanwhile, the absorption and emission spectra of DTPAP-2B and DTPAP-2P were further recorded in the solid powder state. Both powders exhibit a broad absorption band ranging from 450 nm to 1050 nm, with absorption maxima at approximately 575 nm for DTPAP-2B and 660 nm for DTPAP-2P, respectively. Notably, DTPAP-2P displays significantly enhanced NIR-II emission compared to DTPAP-2B under the same conditions (Figure S19).

On page 11 in the revised manuscript:

And the rotors in TPA donors promote the conversion of light to heat through the non-radiative pathways. Then, the photothermal performance of DTPAP-2B and DTPAP-2P in the solid powder state was further evaluated. Both samples exhibited pronounced concentration- and power density-dependent temperature elevations upon 660 nm laser irradiation. As shown in Figure S43, under 660 nm irradiation at 0.4 W cm^{-2} for 30 s, the temperature of DTPAP-2P powder rapidly increased to 96 °C, whereas DTPAP-2B powder reached 75 °C under identical conditions.

On page S25 in the revised Supporting Information:

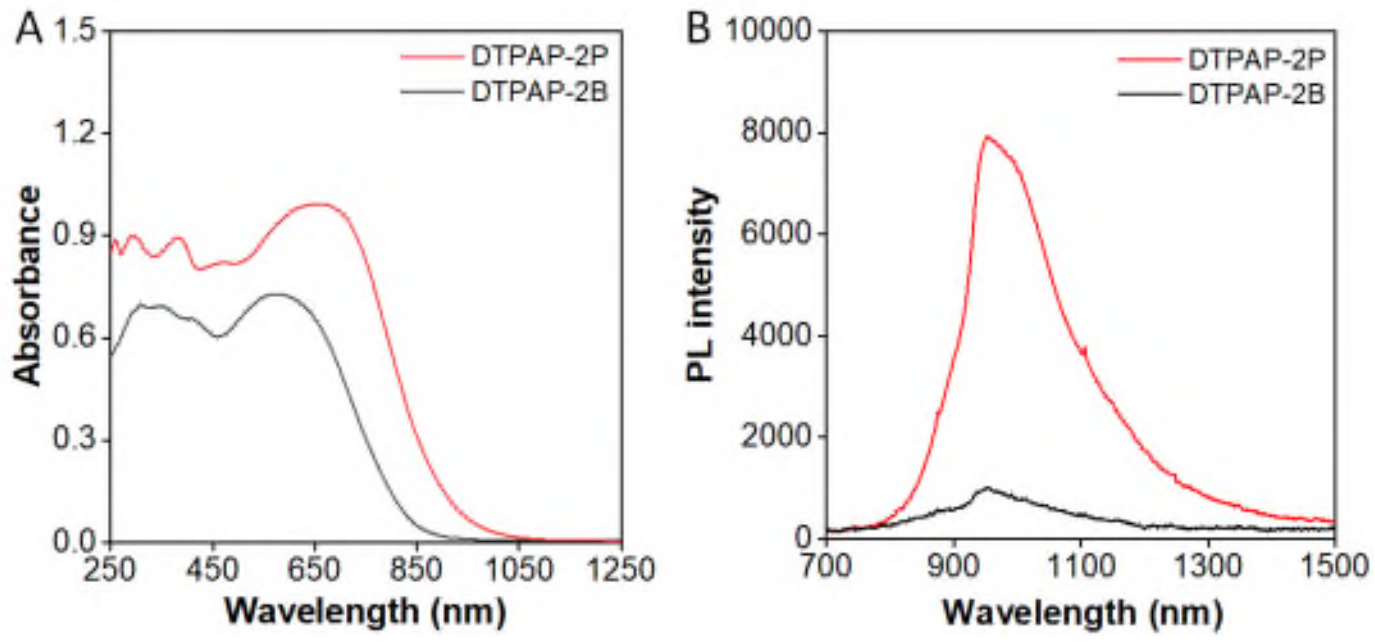

Figure S19. (A) Absorption spectra and (B) Photoluminescence (PL) spectra of DTPAP-2P, DTPAP-2B as a raw powder. λ_{ex}=660 nm.

On page S36 in the revised Supporting Information:

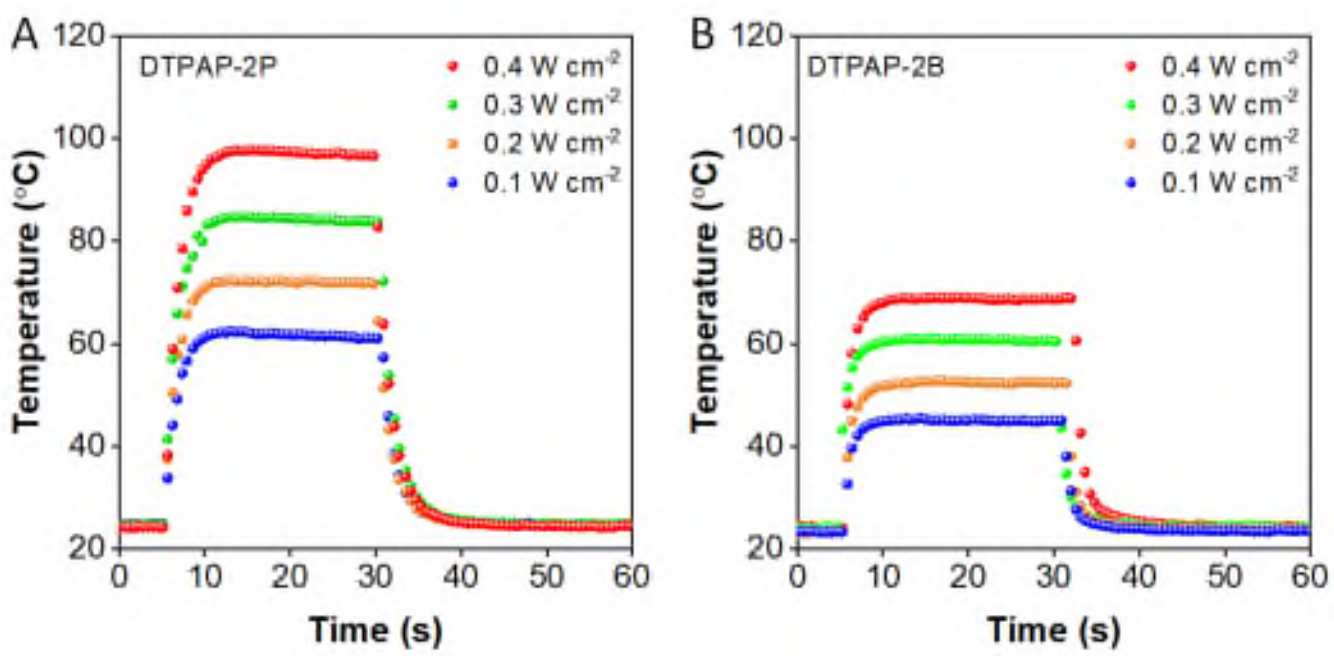

Figure S43. The laser power-dependent temperature elevation curves of DTPAP-2P (A) and DTPAP-2B (B) as a raw powder upon 660 nm laser irradiation.

(3) How can the mean diameter observed by TEM be superior than the hydrodynamic diameter measured by DLS?

Reply: We thank the reviewer for pointing out this inconsistency. We agree that under normal circumstances, the mean diameter observed by TEM (dry state) should be smaller than or comparable to the hydrodynamic diameter measured by DLS (hydrated state). Upon re-examination, we found that the previously observed larger TEM diameter likely resulted from measurements performed on different batches of nanoparticles. To ensure data consistency, we have recollected these data using nanoparticles from the same batch. The mean diameters obtained by TEM (counting over 200 well-dispersed individual nanoparticles) are now determined to be 77 nm for DTPAP-2P NPs and 95 nm for DTPAP-2B NPs, which are smaller than the corresponding hydrodynamic diameters measured by DLS (96 nm for DTPAP-2P NPs and 102 nm for DTPAP-2B NPs). This

revised trend is consistent with the expected physical relationship between dry-state and hydrated-state particle sizes. The corrected data have been updated in the revised manuscript and the Supporting Information.

On page 7 in the revised manuscript:

In order to improve their water solubility and biocompatibility for subsequent biological applications, the corresponding NPs of DTPAP-2B and DTPAP-2P were prepared according to a one-step nanoprecipitation strategy with amphiphilic copolymer Pluronic F127. Dynamic light scattering (DLS) results (Figure 1c) showed that DTPAP-2B NPs and DTPAP-2P NPs displayed an average hydrodynamic diameter of 102 nm and 96 nm, respectively. Images obtained through transmission electron microscopy (TEM) also verified the spherical morphology of DTPAP-2B NPs and DTPAP-2P NPs as shown in Figure S20.

On page S26 in the revised Supporting Information:

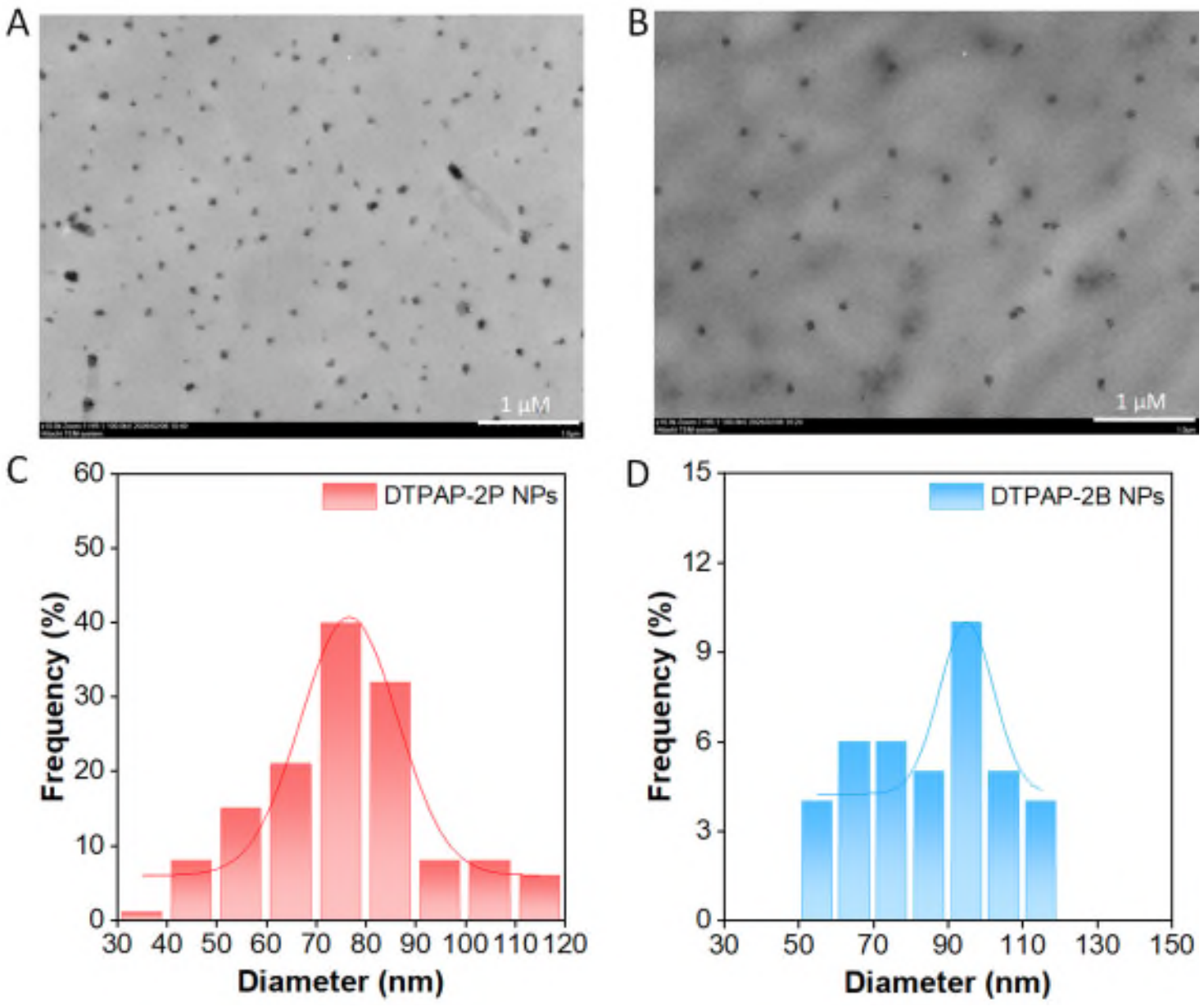

Figure S20. TEM images of (A) DTPAP-2P NPs and (B) DTPAP-2B NPs. The size histogram for (C) DTPAP-2P NPs and (D) DTPAP-2B NPs.

(4) Why the cellular uptake was studied on HeLa cells and the phototoxicity on 4T1 cells?

Reply: We thank the reviewer for this insightful question regarding the choice of cell lines in our study. We agree that consistency in experimental design is important for the interpretation of cellular

uptake and phototoxicity data. In our initial study, cellular uptake was examined in HeLa cells, while phototoxicity was evaluated in 4T1 cells, primarily due to the different availability of cell lines at the time of each experiment. However, to address the reviewer's concern and to rule out the possibility of cell-line-dependent effects, we have performed additional comparative experiments using both cell lines under identical conditions.

Specifically, we investigated the uptake kinetics of DTPAP-2P NPs in 4T1 cells and compared the results with those previously obtained in HeLa cells. As shown in Figures S50–S51, the uptake profile in 4T1 cells closely resembles that in HeLa cells, with both cell lines reaching near-maximum internalization at 8 h post-incubation. These results indicate that the uptake efficiency and kinetics of DTPAP-2P NPs are comparable between the two cell lines and are not cell-type-dependent.

Furthermore, to ensure that the phototherapeutic efficacy is not unique to 4T1 cells, we evaluated the phototoxicity of DTPAP-2P NPs on HeLa cells under 660 nm irradiation. As depicted in Figure S54, the phototoxic effect observed in HeLa cells is comparable to that in 4T1 cells, confirming that the phototherapeutic activity of DTPAP-2P is robust and not limited to a specific cell line. The related data and discussion have been added to the revised manuscript and the Supporting Information.

We sincerely appreciate the reviewer's careful attention to this point, which has allowed us to strengthen the generality and rigor of our conclusions.

On page 15 in the revised manuscript:

As time goes by, the red fluorescent signals enhanced obviously, reaching a plateau at 8 h (Figure S51A). A similar trend was observed in 4T1 cells, where the intracellular uptake approached a plateau at approximately 8 h (Figure S50B-S51B).

On page 17 in the revised manuscript:

However, under the same conditions, the killing rate of DTPAP-2B NPs is much weaker compared to DTPAP-2P NPs, with the cell survival rate exceeding 60%. Consistent results were likewise obtained in HeLa cells, further corroborating the photocytotoxicity of DTPAP-2P NPs (Figure S54).

On page S8 in the revised Supporting Information:

Cellular Uptake Assay

20 μM of DTPAP-2P NPs and DTPAP-2B NPs were added to 5 × 10^6 HeLa cells or 4T1 cells and incubated at 37 °C for 2, 4, 6, 8, 10, 12 h. Then cells were imaged using confocal laser scanning microscopy (CLSM). The excitation wavelength of DTPAP-2P NPs and DTPAP-2B NPs was 543 nm and the emission collection wavelength was LP570 nm.

On page S9 in the revised Supporting Information:

Cell Viability

4T1 cells or HeLa cells were seeded on 96-well plates in RPMI 1640 medium or DMEM medium (with 10% FBS, 1% penicillin/streptomycin) in 5% CO_2, 20% O_2 at 37 °C in a humidified incubator. Then, the cells in a logarithmic growth phase were harvested and seeded in 96-well plates at a density of 5 × 10^3 cells for 24 h incubation. Subsequently, the medium was replaced with the fresh medium containing different concentrations of DTPAP-2B NPs or DTPAP-2P NPs.

On page S40-S42 in the revised Supporting Information:

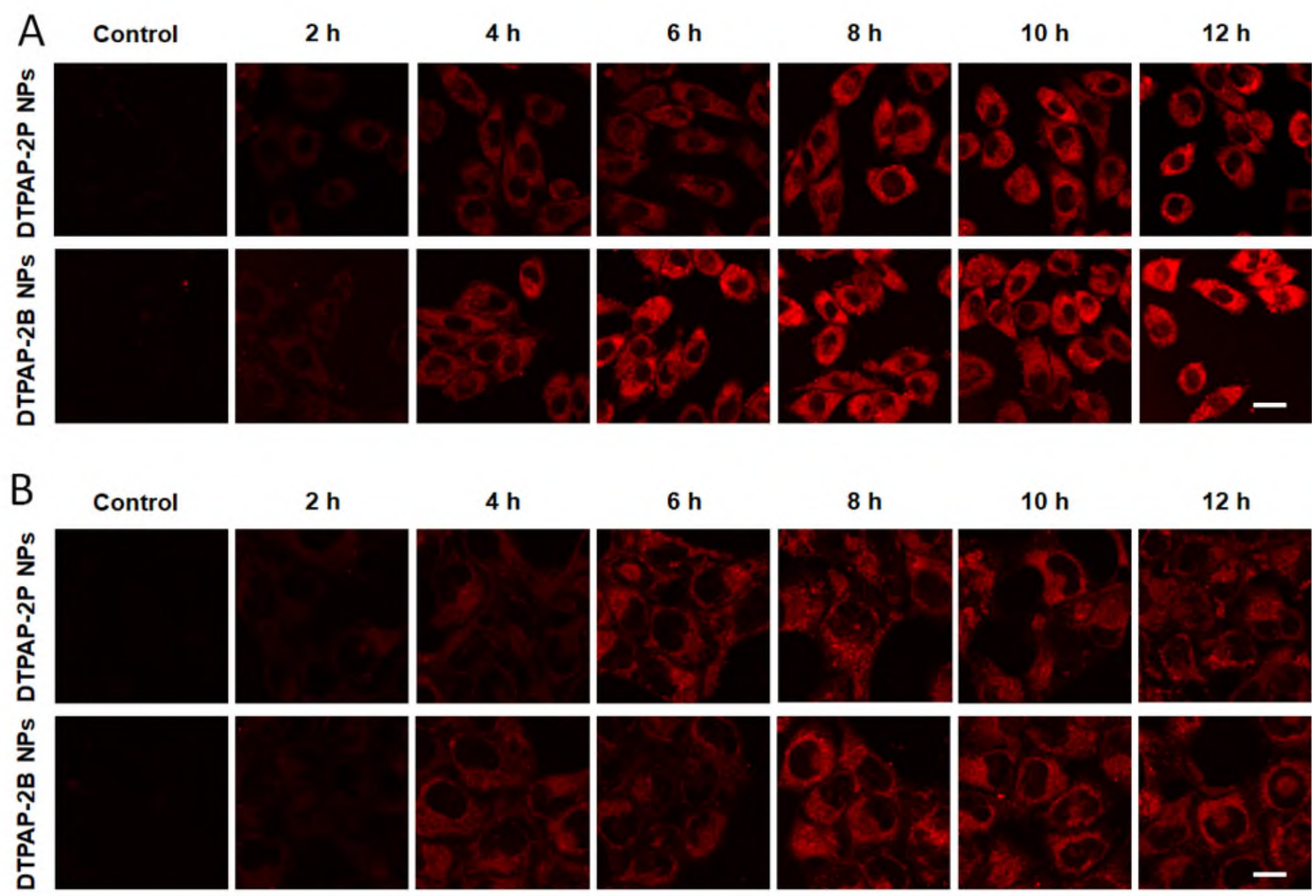

Figure S50. Uptake of DTPAP-2P NPs and DTPAP-2B NPs by 4T1 cells (A) and HeLa cells (B) at different incubation times. Scale bar = 20 μm. Control represents the cells incubated with the absence of any NPs.

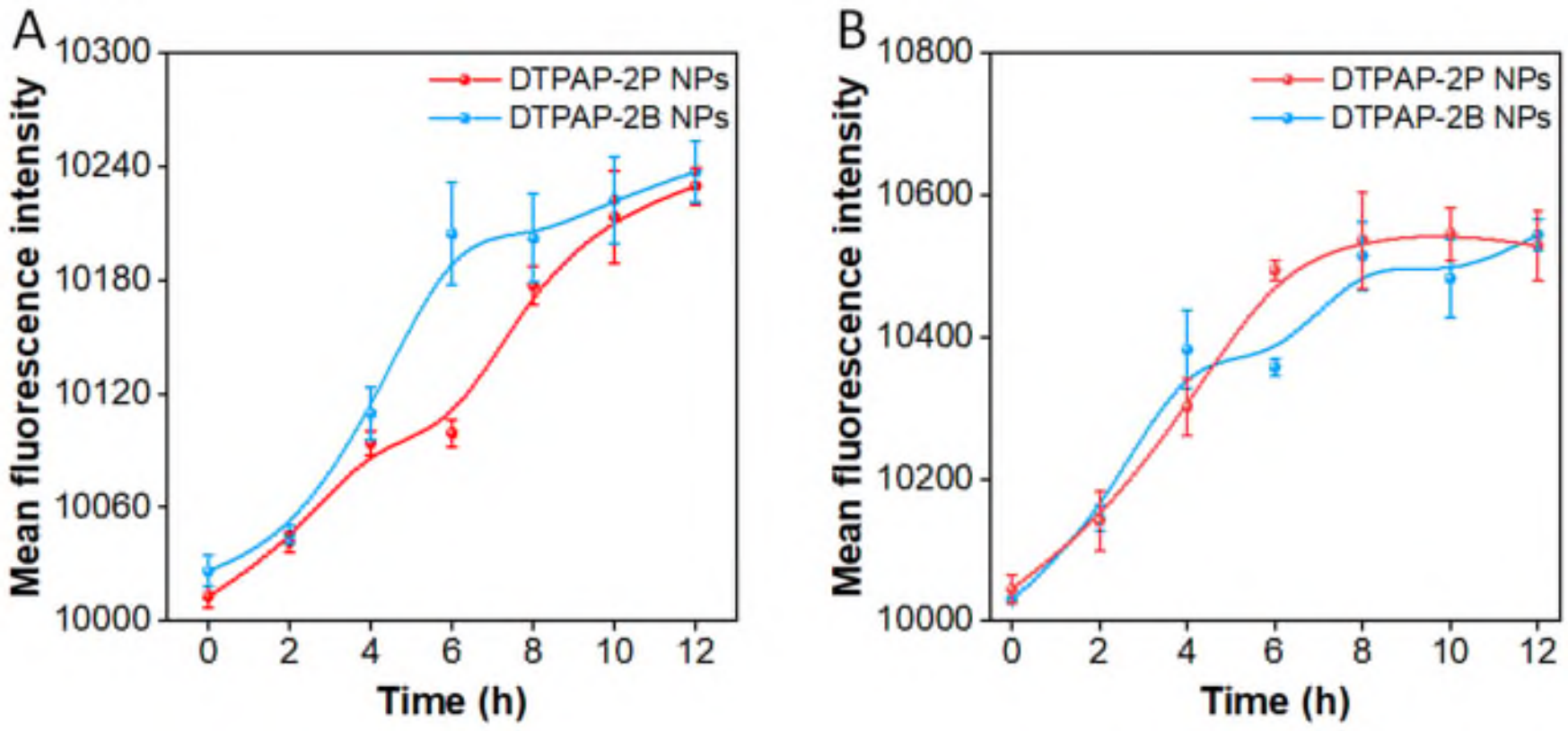

Figure S51. The mean fluorescence intensity of DTPAP-2P NPs and DTPAP-2B NPs inside 4T1 cells (A) and HeLa cells (B) at different incubation times obtained from Figure S50.

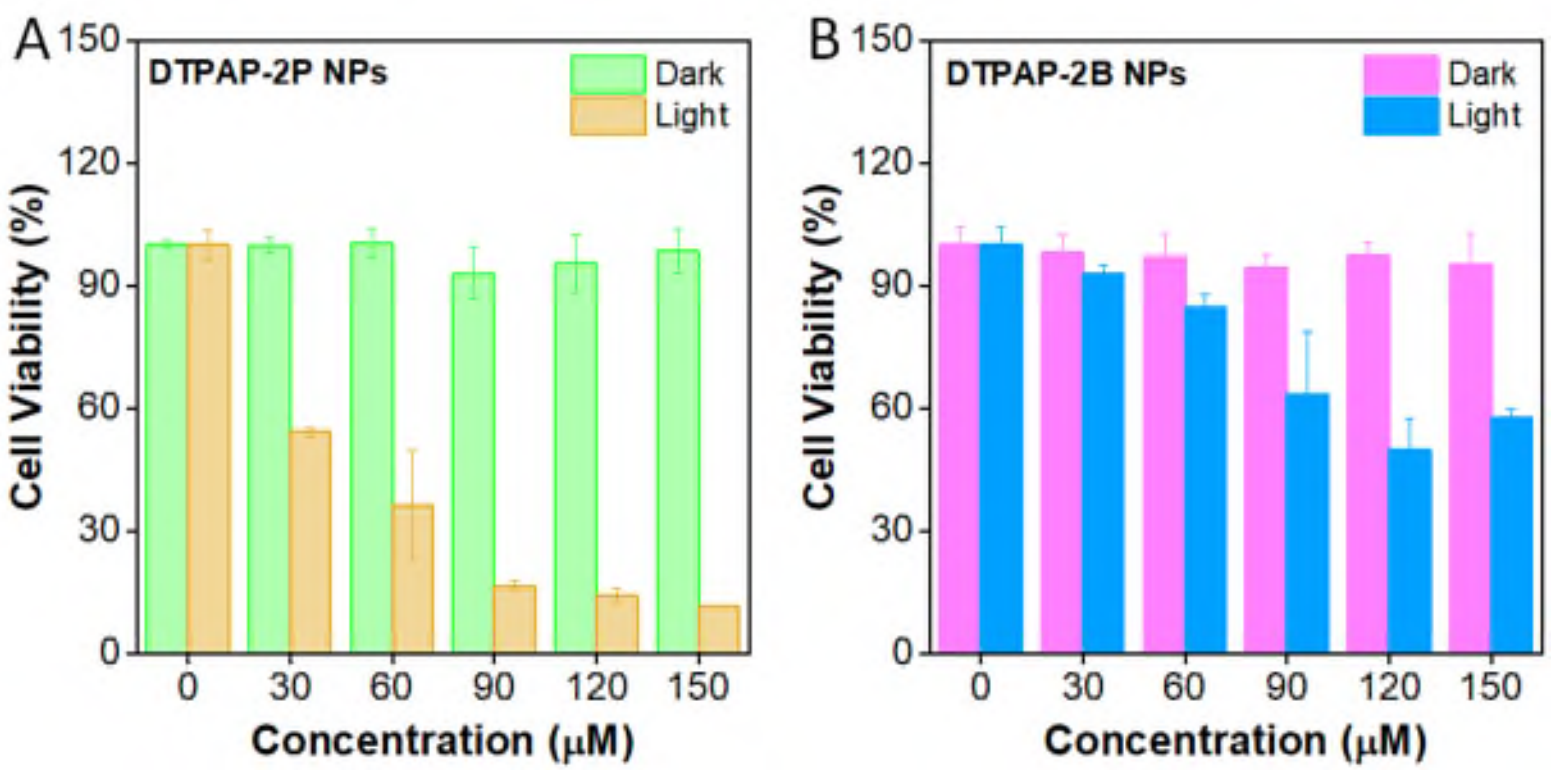

Figure S54. Viability of HeLa cancer cells incubated with increasing concentrations of DTPAP-2P NPs (A) and DTPAP-2B NPs (B) without or with 660 nm laser irradiation (0.4 W cm^{-2}) for 10 min.

(5) For the in vivo cancer study, the nanoparticles (NPs) were administered via intratumoral injection. Could the authors clarify whether systemic administration (e.g. intravenous injection) was also considered or evaluated? In addition, since the NPs are locally injected and confined to the tumor site, is it expected that their toxicity toward other organs would be limited? Further discussion or experimental evidence regarding systemic distribution and off-target toxicity would strengthen the manuscript.

Reply: We sincerely thank the reviewer for this important and clinically relevant comment. We fully agree that intravenous administration represents a more clinically relevant route. Although the uniform hydrodynamic size of DTPAP-2P NPs and their excellent colloidal stability in biological media initially suggested their suitability for systemic delivery and EPR-mediated tumor

accumulation, our preliminary attempts at intravenous injection revealed that DTPAP-2P NPs did not achieve efficient tumor enrichment via the EPR effect (Figure R1-R3). In light of this observation, we intentionally adopted intratumoral injection to evaluate the intrinsic phototherapeutic efficacy of the nanoparticles. This administration strategy enables precise control over the local dose, maximizes tumor retention, and allows a more direct assessment of photodynamic and photothermal performance *in vivo*.

Regarding off-target toxicity, because the NPs were administered intratumorally and largely confined to the tumor site, systemic exposure is expected to be substantially limited. As displayed in Figure S56, bright NIR-II emission was captured in the tumor site, while a weak signal was also observed in the liver and in the kidney, indicating that the DTPAP-2P NPs may be metabolized by these two organs and excreted from the body. Consistently, no significant body-weight loss was observed throughout the treatment period. Furthermore, histological examination (H&E staining) of major organs, including heart, liver, spleen, lung, and kidney, revealed no obvious pathological abnormalities, indicating negligible acute systemic toxicity under the experimental conditions. To clarify these points, we have added a corresponding discussion in the revised manuscript.

On page 19 in the revised manuscript:

As shown in Figures 5d and 5e, the NIR-II fluorescence signals at tumor site were rapidly increased and then maintained constantly for 96 h after the NPs administration, indicating DTPAP-2P NPs may be used for long-term tracking of the therapeutic process. After 24 h of DTPAP-2P NPs injection, the fluorescence signal of the tumor was very strong. Then, the major organs (heart, liver, spleen, lung, and kidney) and tumors of mice were dissected for *ex vivo* NIR-II imaging. As displayed in Figure S56, the bright NIR-II luminescence on tumor, in contrast to the negligible signal on the heart and lung, further confirmed the prolonged retention capability of DTPAP-2P NPs at the tumor site. The fluorescence quantitative assay demonstrated, DTPAP-2P NPs are mainly distributed in the liver, spleen, kidney and tumor, suggesting liver, spleen and kidney may be involved in the metabolism and clearance of DTPAP-2P NPs.

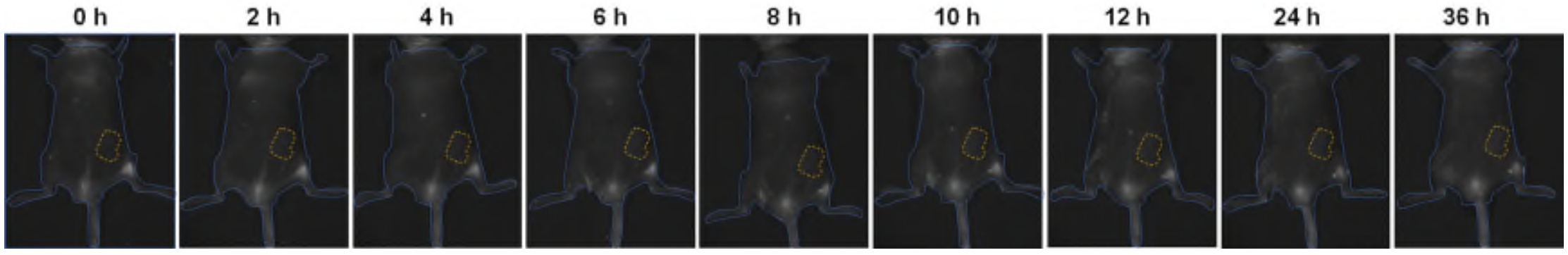

Figure R1. NIR-II fluorescence imaging of the tumor before and after *i.v.* injection of DTPAP-2P NPs at different time points (0, 2, 4, 6, 8, 10, 12, 24, and 36 h).

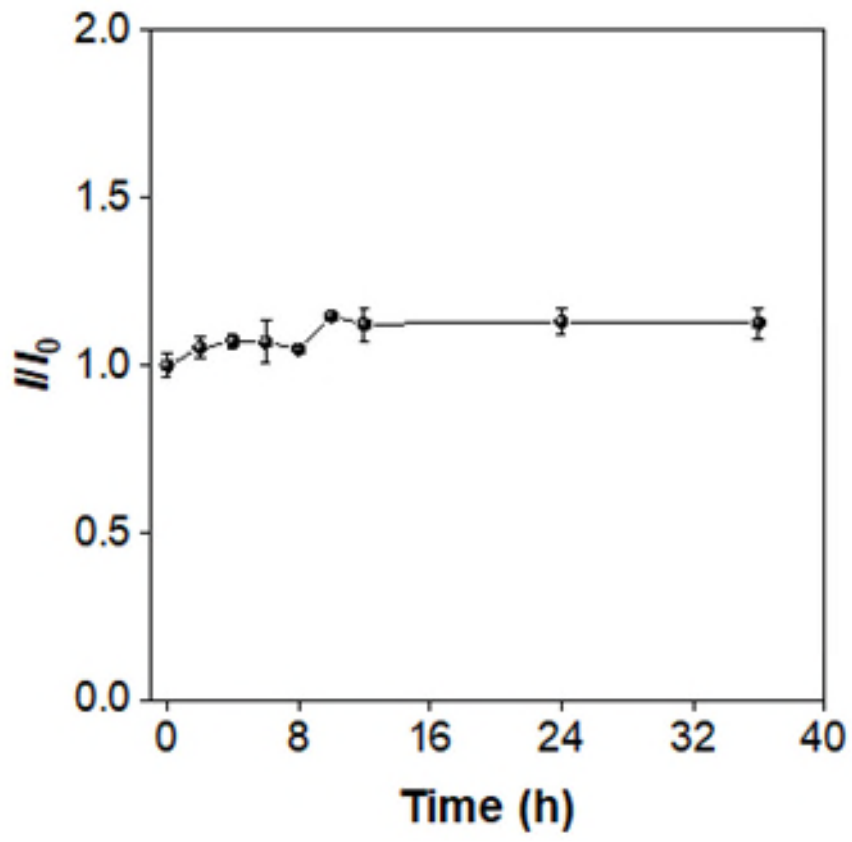

Figure R2. Corresponding fluorescence signals of the tumor before and after *i.v.* injection of DTPAP-2P NPs at different time points obtained from Figure R1.

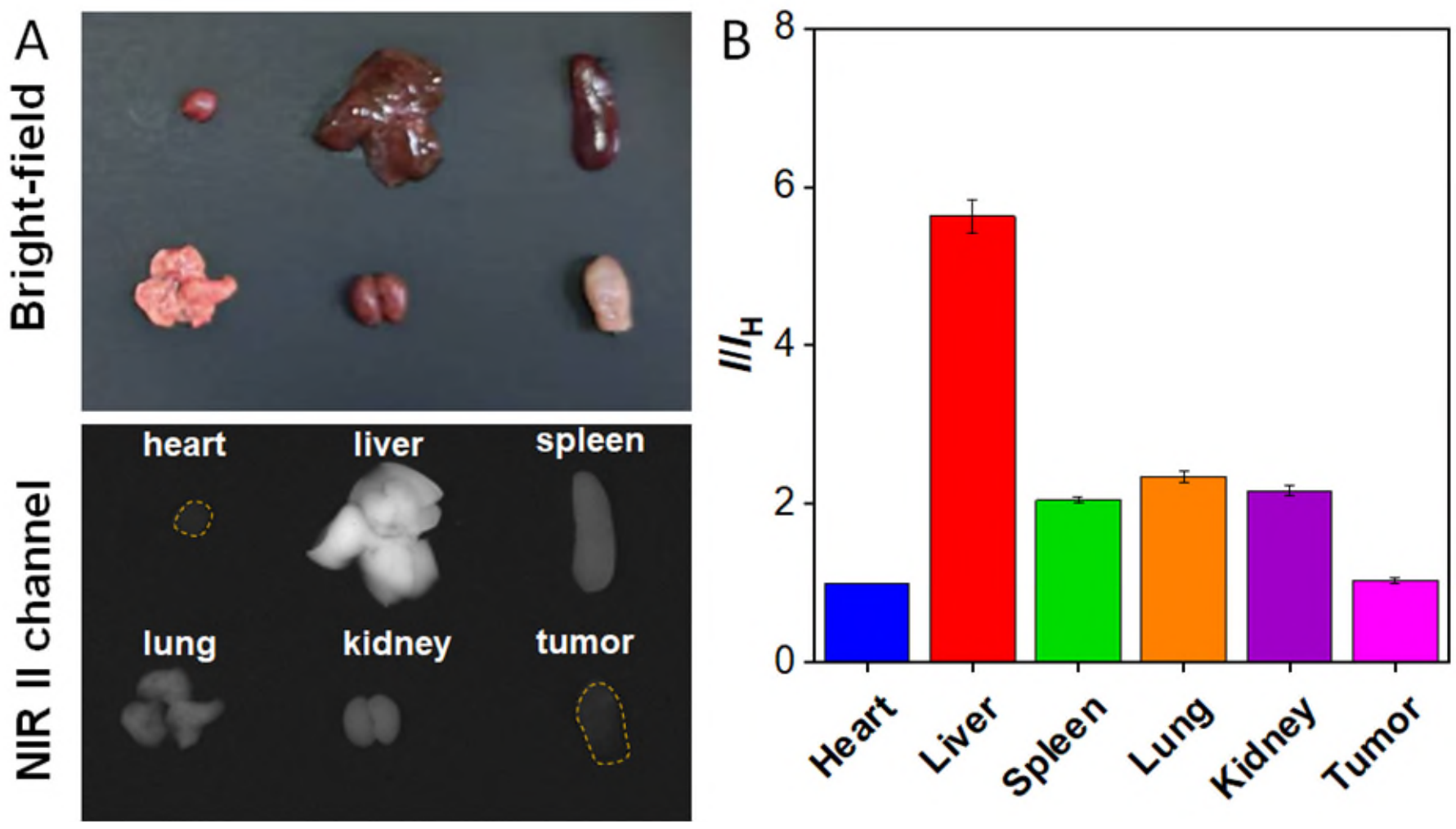

Figure R3. (A) The images of the major organs (heart, liver, spleen, lung, kidney) and tumor in bright-field and NIR-II channel after 12 h post-injection of DTPAP-2P NPs. (B) The I/I_H ratio of the major organ (heart, liver, spleen, lung, kidney) after 12 h *i.v.* injection of DTPAP-2P NPs. I represent the PL intensity of the major organ, while I_H indicates the PL intensity of the heart.

On page S43 in the revised Supporting Information:

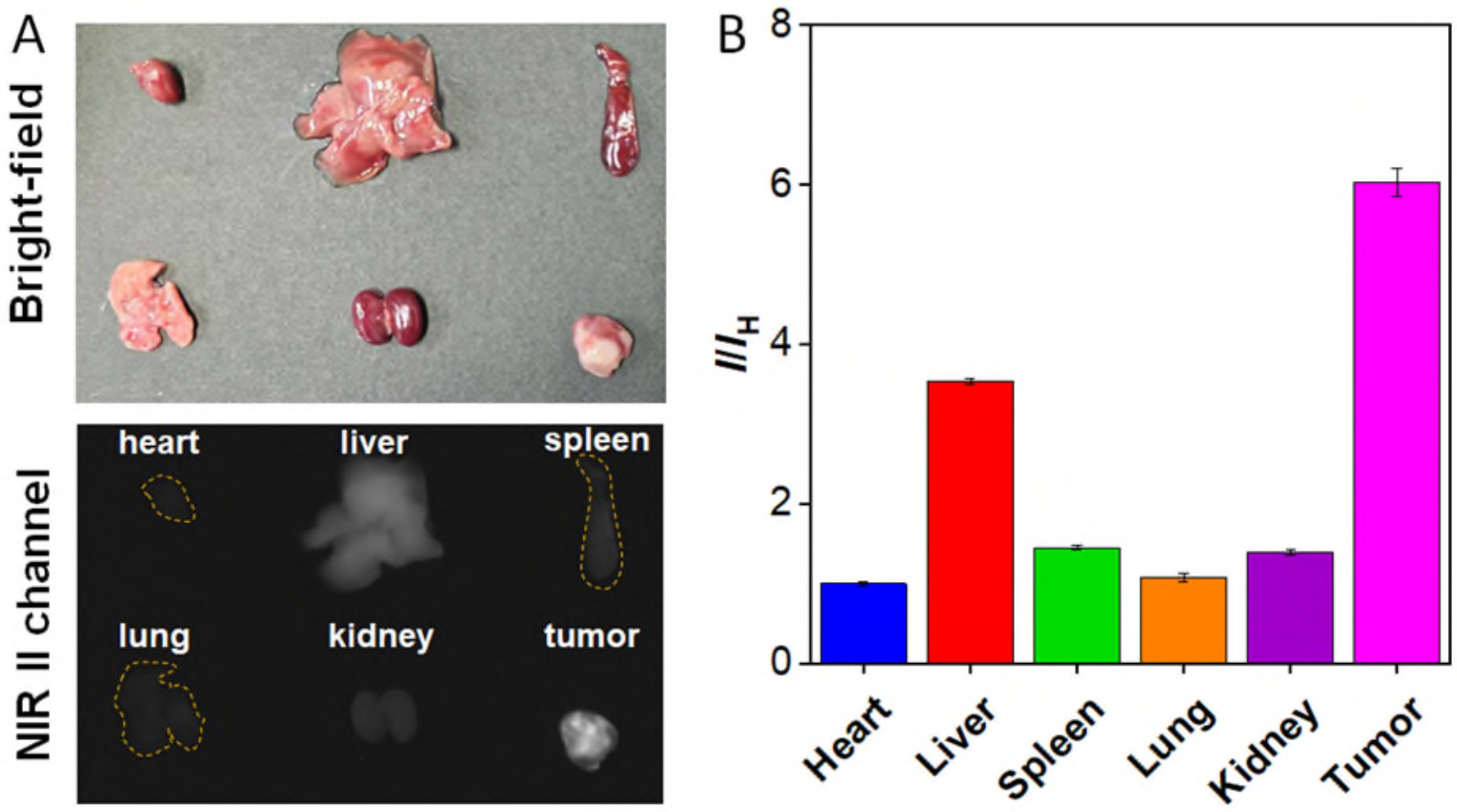

Figure S56. (A) The images of the major organs (heart, liver, spleen, lung, kidney) and tumor in bright-field and NIR-II channel after 24 h post-injection of DTPAP-2P NPs. (B) The I/I_H ratio of the major organ (heart, liver, spleen, lung, kidney) after 24 h post-injection of DTPAP-2P NPs. I represent the PL intensity of the major organ, while I_H indicates the PL intensity of the heart.

(6) The final paragraph functions as a conclusion rather than a discussion. The authors should use "results and discussion".

Reply: We sincerely thank the reviewer for this helpful suggestion. According to your suggestion and the journal's requirements, we have revised this part to give a discussion of the design strategy.

On page 22-23 in the revised manuscript:

The development of robust NIR-II PTAs is of high significance for advanced deep-tissue phototheranostics. Although anion–π^+-type photosensitizers have shown great promise, current studies have largely focused on modifying the π^+-system or introducing simple inorganic counterions, leaving the development of anion-π^+ type NIR-II PTAs largely unexplored. In this work, we propose a simple yet effective strategy to construct an anion-π^+ type NIR-II PTA (DTPAP-2P) by replacing the inorganic Br^- in DTPAP-2B with organic planar π-conjugated $(CCN)_5^-$. Stark contrast in properties between DTPAP-2B and DTPAP-2P underscores the critical role of the organic planar π-conjugated anion. In solution, both DTPAP-2B and DTPAP-2P exhibited similar and poor optical properties. In contrast, upon formation of NPs, DTPAP-2P NPs demonstrated redshifted absorption, bright NIR-II emission, and dramatically enhanced ROS generation and PCE,

illustrating the advantages of the strong, dual anion-π^+ interactions induced by the introduction of organic planar π-conjugated $(CCN)_5^-$. Single crystal structure analysis confirms that DTPAP-2P exhibits much stronger dual anion–π^+ interactions compared to the weak single anion–π^+ interaction in DTPAP-2B. Further crystallographic analysis and theoretical calculations reveal that these dual anion–π^+ interactions enhance intramolecular D–A interactions while suppressing detrimental intermolecular π–π stacking between luminogens. Consequently, DTPAP-2P NPs exhibit significant red shifts in maximum absorption and emission wavelengths, as well as a marked increase in the molar extinction coefficient at 660 nm, relative to DTPAP-2B NPs. The successful extension of this strategy to other π-conjugated cations (DMPHL-2B and DMPHL-2P) further demonstrates the generality of our approach.

The biological evaluation validates the power of our molecular design. Efficient mitochondrial targeting, high-resolution NIR-II angiography capable of resolving capillary-level microvessels, and successful NIR-II imaging-guided synergistic therapy in a murine tumor model collectively demonstrate the great potential of DTPAP-2P NPs for cancer theranostics. The absence of systemic toxicity in major organs and blood panels further confirms the excellent biocompatibility of this nanoplatform. Overall, the strategy of introducing an organic planar π-conjugated counterion provides a facile route to anion–π^+-type NIR-II PTAs.

(7) In SI:

The 13C NMR peak should attributed.

Reply: We thank the reviewer for the careful reading of the Supporting Information and for pointing out this important issue regarding the NMR characterization. In response to this comment, we have performed a detailed assignment of the ^1H NMR and ^{13}C NMR peaks for both DTPAP-2B and DTPAP-2P based on comprehensive analyses of the ^1H NMR, ^{13}C NMR and ^1H–^{13}C NMR heteronuclear correlation (HSQC) spectra. We sincerely appreciate the reviewer's constructive suggestion, which has helped improve the clarity and rigor of our Supporting Information.

On page S17 and S19 in the revised Supporting Information:

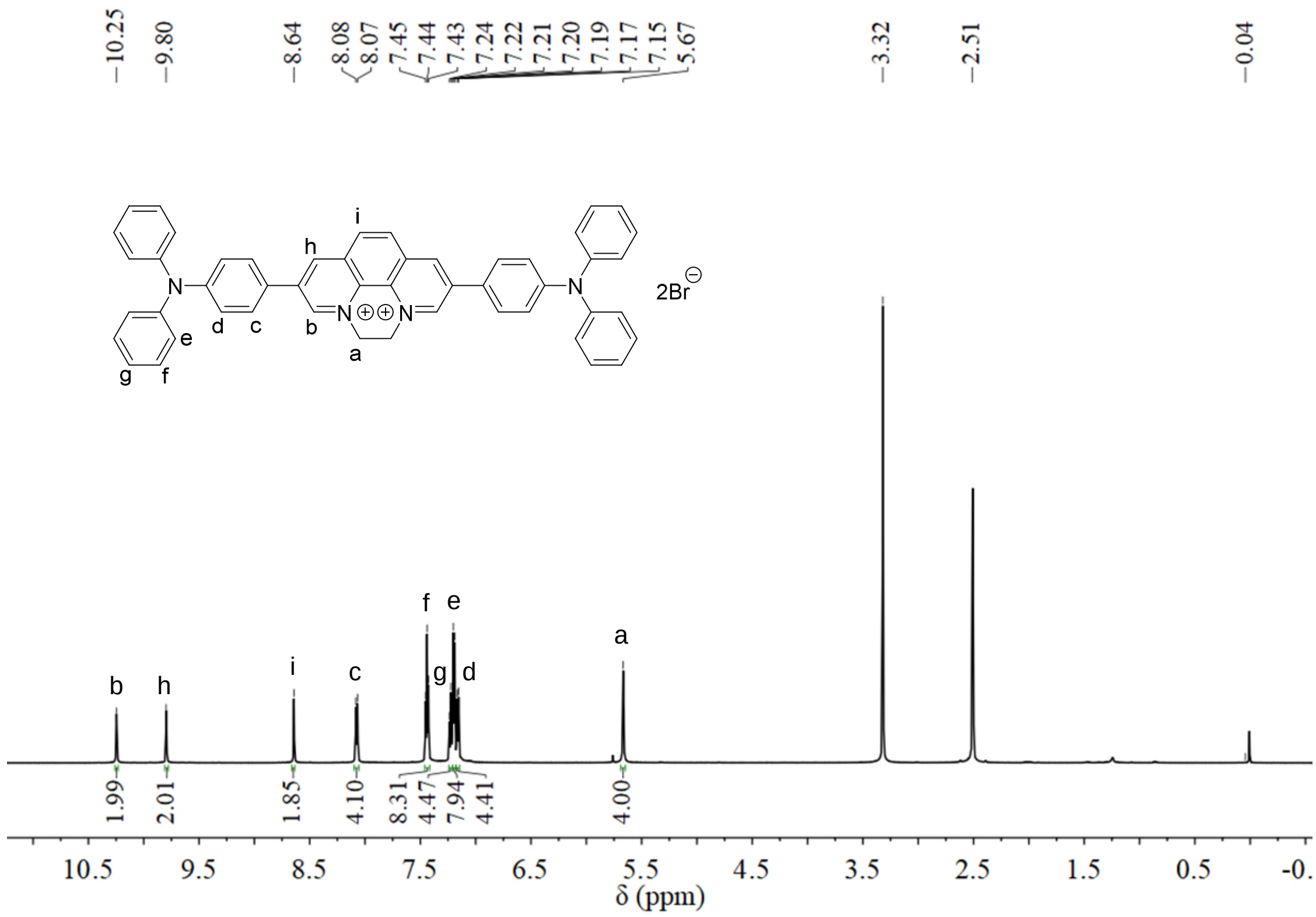

Figure S1. ^{1}H NMR spectrum of DTPAP-2B in DMSO-d_6.

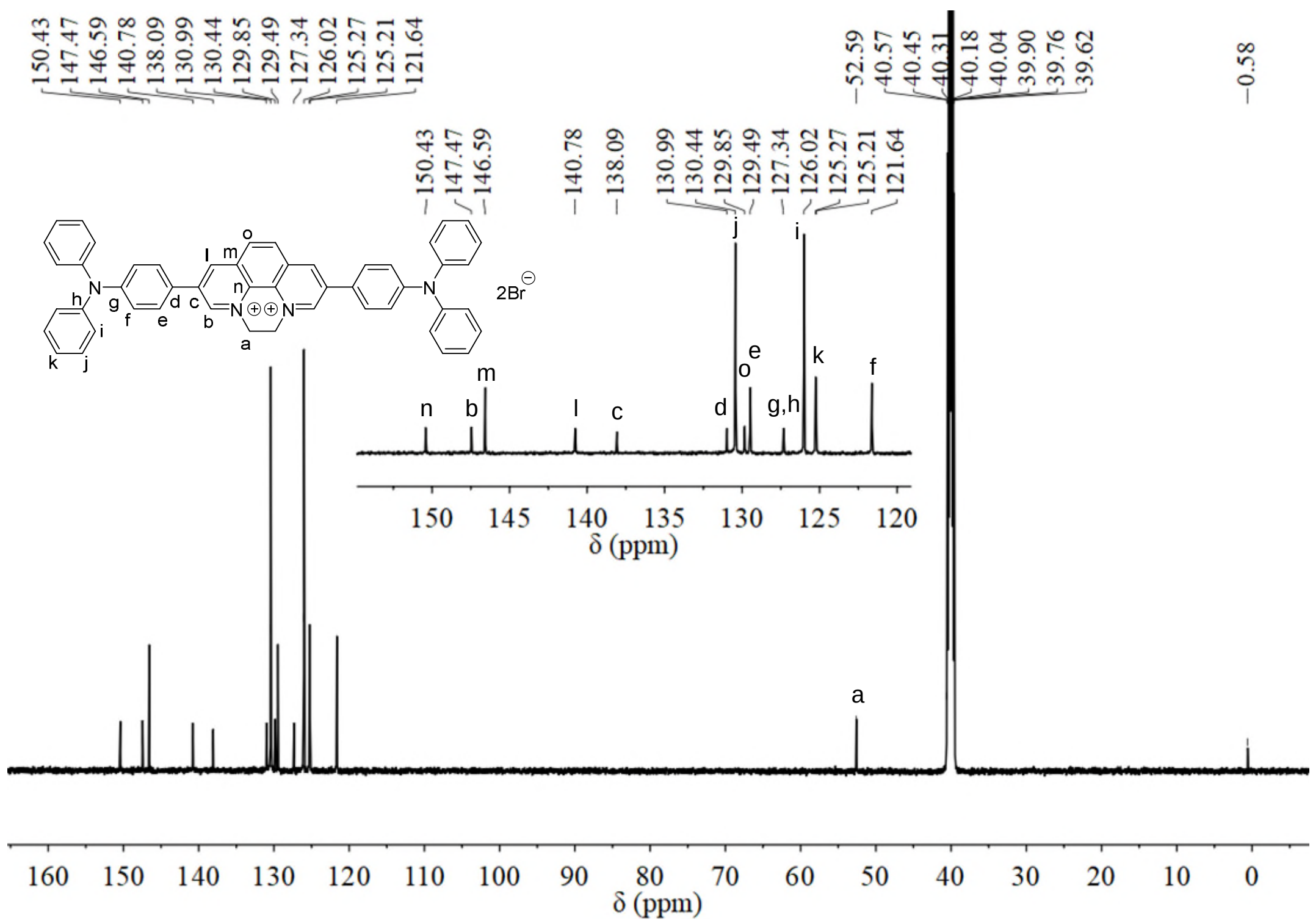

Figure S2. ^{13}C NMR spectrum of DTPAP-2B in DMSO-d_6.

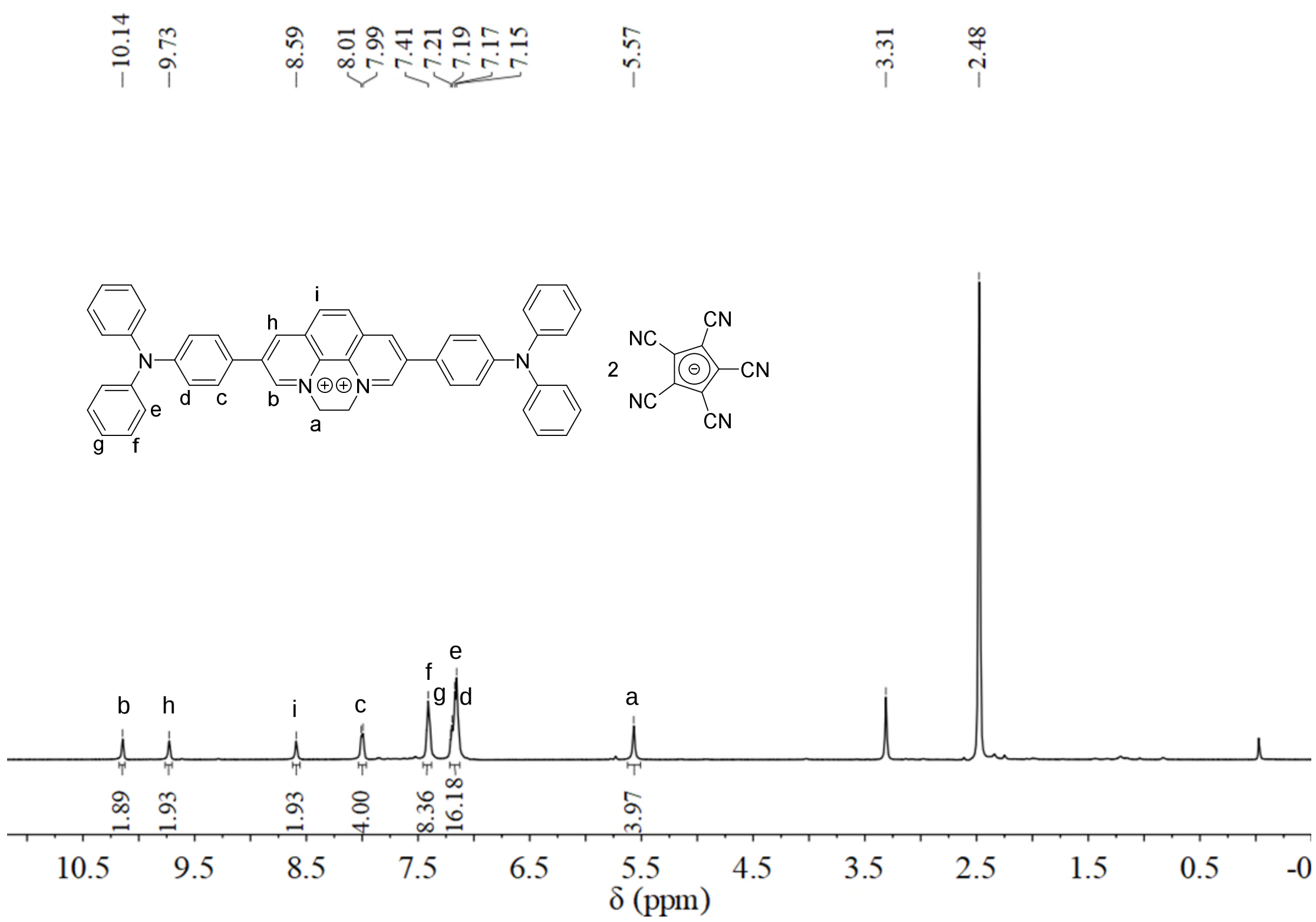

Figure S5. ^{1}H NMR spectrum of DTPAP-2P in DMSO-d_6.

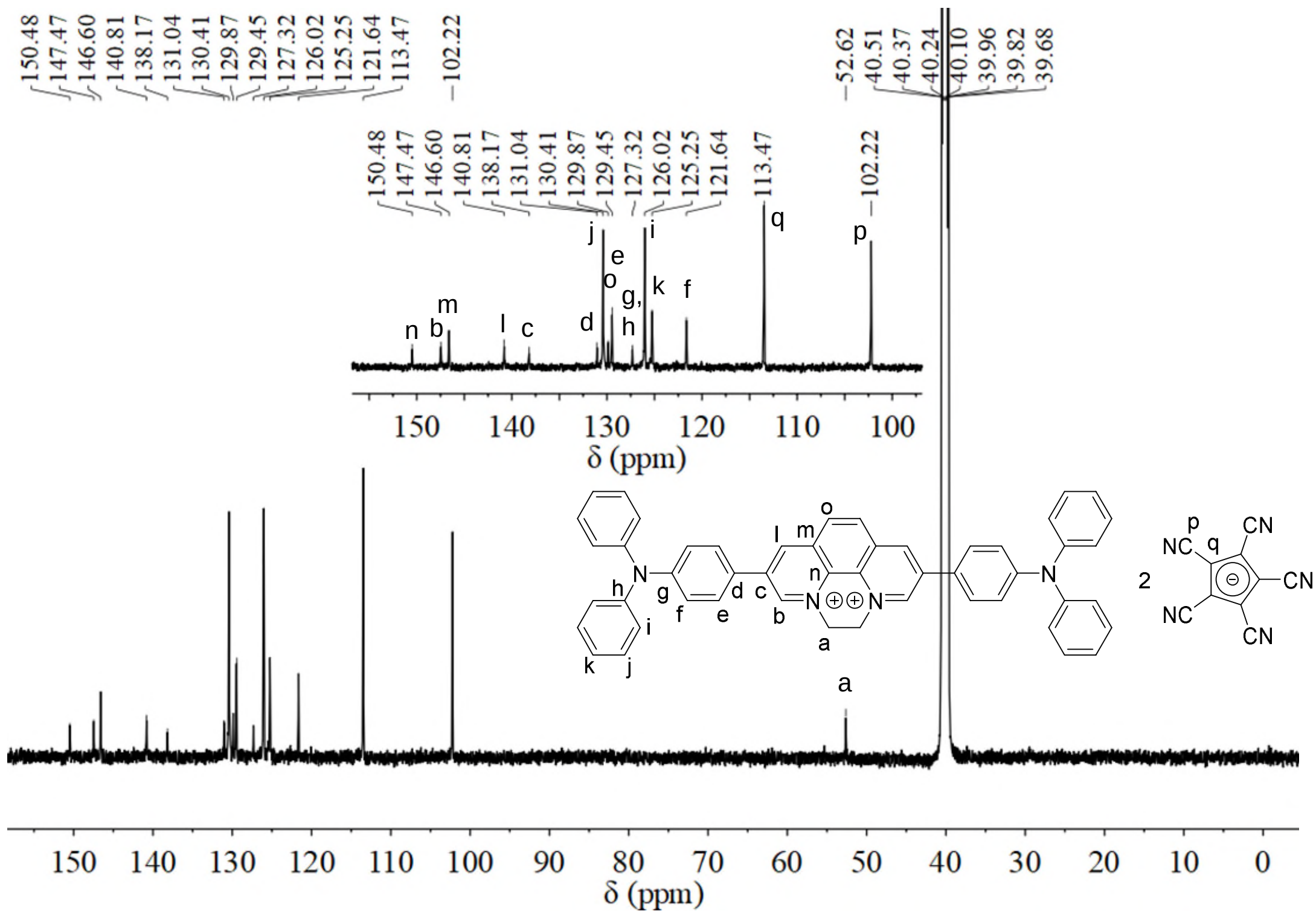

Figure S6. ^{13}C NMR spectrum of DTPAP-2P in DMSO-d_6.

(8) equation a, nref not nnef

Reply: We sincerely thank you for your kind suggestion. Accordingly, we have carefully checked the whole manuscript. We have amended the n_{ref} in equation (a) (now equation (b)), and checked the details of related experiments in the manuscript.

On page S5 in the revised Supporting Information:

$$QY_{sample} = QY_{ref} \times \frac{slope_{sample}}{slope_{ref}} \times \left(\frac{n_{sample}}{n_{ref}}\right)^2 \quad (b)$$

(9) In table S1, which reference was used to calculate absolute fluorescence quantum yield in DMSO? Please revised caption.

Reply: We sincerely thank the reviewer for pointing out this. The absolute fluorescence quantum yield of compounds in DMSO was determined using an integrating sphere system without reference to an external standard. Specifically, the measurements were carried out on a calibrated spectrofluorometer equipped with an integrating sphere, following the instrument's built-in absolute quantum yield measurement protocol. To avoid ambiguity, we have revised the caption of Table S1 (now Table S2) to explicitly state the measurement method. We appreciate the reviewer's careful attention to details, which has helped us to improve the accuracy and clarity of our manuscript.

On page S27 in the revised Supporting Information:

Table S2. Photophysical properties and excited-state decay rate of DTPAP-2B and DTPAP-2P.

Sample	QY (%)	τ_f (10^{-9} s)	k_r (10^6 s^{-1})	k_{nr} (10^8 s^{-1})	$^1O_2$
DTPAP-2B	0.30[a]	2.48	1.21	4.02	0
DTPAP-2B NPs	0.30[a]	0.76	3.95	13.12	0.25
DTPAP-2P	0.50[a]	2.37	2.11	4.20	0
DTPAP-2P NPs	0.62[b]	1.01	6.14	9.84	0.21

[a] Absolute fluorescence quantum yield were determined using an integrating sphere. The QY of DTPAP-2P and DTPAP-2B was determined in DMSO upon excited at 540 nm. The QY of DTPAP-2B NPs was determined in aqueous solution upon excitation at 530 nm.

[b] Relative fluorescence quantum yield. The QY of DTPAP-2P NPs upon 660 nm laser irradiation was calculated using ICG (QY = 2.7%) in water as the reference.

(10) Figure S17b, where Is the second bromide atom?

Reply: We sincerely thank the reviewer for the careful observation. In Figure S17B (now Figure S24B), the second bromine atom was obscured by the molecular skeleton. To avoid any misunderstanding, we have carefully re-examined the figure and adjusted the structural orientation and layout to clearly indicate both bromide atoms. The updated figure now unambiguously reflects the accurate ionic composition.

On page S28 in the revised Supporting Information:

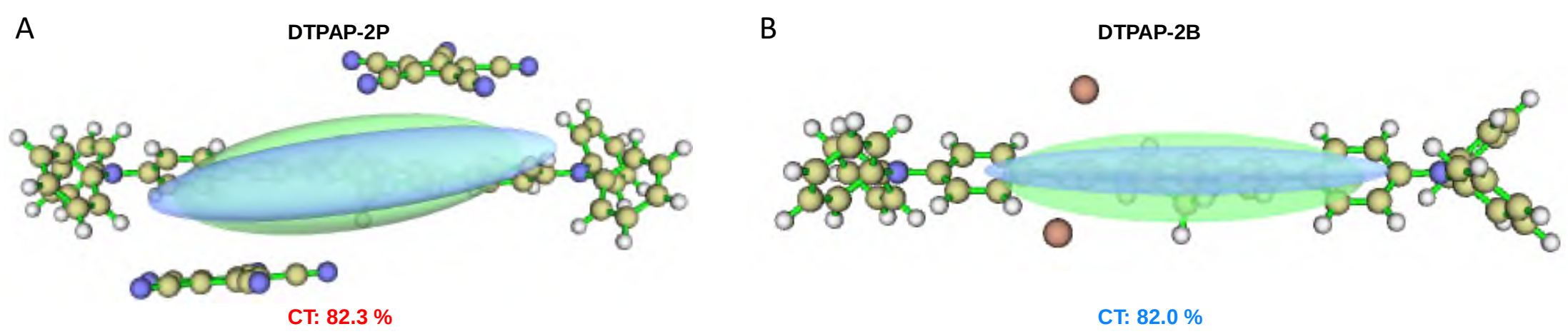

Figure S24. C_{hole}-C_{ele} isosurface plots of DTPAP-2P (A) and DTPAP-2B (B) in the S_1 states (isovalue = 0.0003), CT represented the proportion of CT state.

(11) Why Figure S18a and S19a are not the same? Which concentration have been used?

For the irradiation experiments, in addition of the laser power, the spot size should also be given.

Reply: We thank the reviewer for the careful examination of our Supporting Information figures and for pointing out the missing experimental details. We apologize for the omission of experimental details. The fluorescence spectra of DCFH in Figure S18A (now Figure S25A) and 19A (now Figure S26A) were obtained in DMSO solution and in PBS (pH 7.4) respectively. The concentration of DCFH used in both experiments was 1 μM, which was chosen based on standard protocols for ROS detection to ensure optimal sensitivity without self-quenching. The laser spot size of the 660 nm laser was 7.5 × 7.5 cm. This spot size was sufficiently large to uniformly irradiate the entire area of cuvette used in the experiment, ensuring that all samples received consistent and homogeneous illumination. We apologize for the incomplete experimental details. Now, we have supplemented these details in the revised supporting information.

On page S28-S29 in the revised Supporting Information:

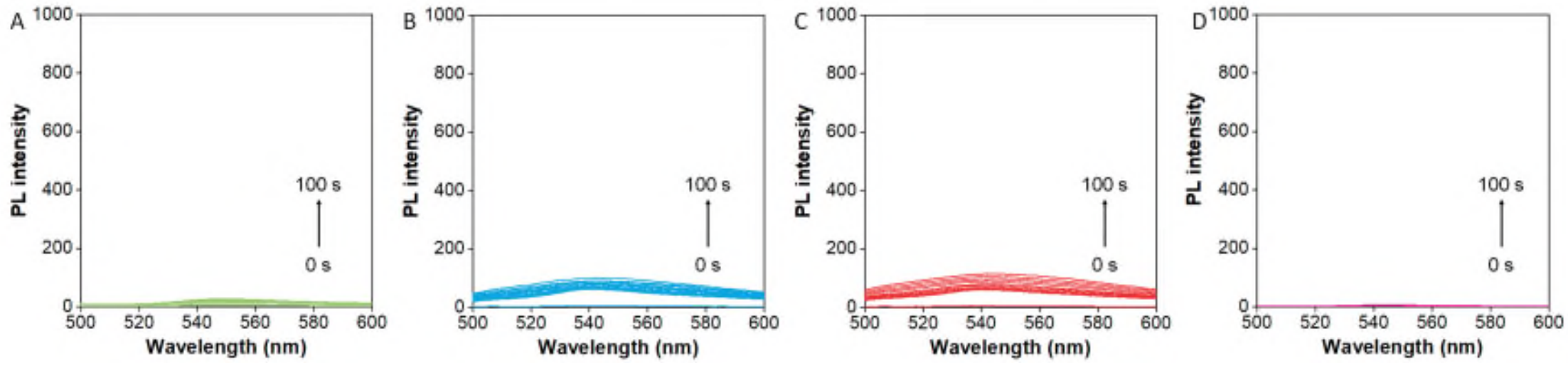

Figure S25. (A) Fluorescence spectra changes of DCFH alone after 660 nm laser irradiation. Fluorescence spectra changes of DCFH induced by DTPAP-2B (B), DTPAP-2P (C) and $Na(CCN)_5$ (D) in DMSO solution under the 660 nm laser irradiation (0.1 W cm^{-2}). The concentration of DCFH was 1 μM and the concentration of compounds were 10 μM, respectively.

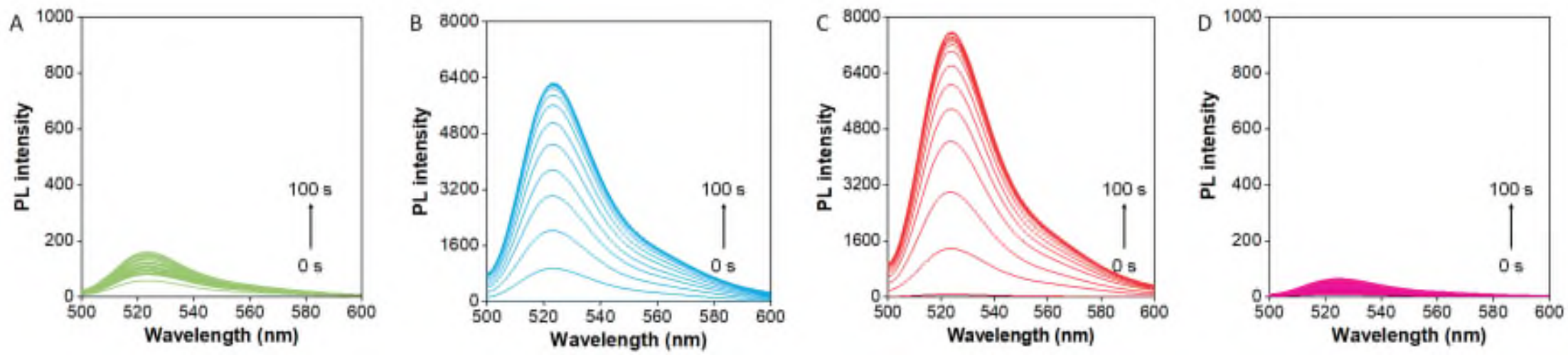

Figure S26. (A) Fluorescence spectra changes of DCFH alone after 660 nm laser irradiation. Fluorescence spectra changes of DCFH induced by DTPAP-2B NPs (B), DTPAP-2P NPs (C) and $Na(CCN)_5$ NPs (D) in PBS (pH 7.4) under the 660 nm laser irradiation (0.1 W cm^{-2}). The concentration of DCFH was 1 μM and the concentration of NPs were 10 μM, respectively.

(12) Page 4, "The as-prepared NPs were concentrated and calibrated before use."? Please explain how the exact concentration of compound was determined in the NPs suspensions after dialysis. How the molar extinction coefficient was calculated?

Reply: We thank the reviewer for the careful examination of our Supporting Information figures. To calculate the exact concentration of compound, a series of standard solutions of DTPAP-2B or DTPAP-2P in DMSO were prepared, and a calibration curve was constructed by plotting absorbance versus concentration. Then, a small volume (50 μL) of NPs in aqueous solution was dispersed in DMSO, and the absorption was recorded. Thus, the molar concentration was determined according to the standard calibration curve. Subsequently, the UV–vis absorption spectra of the NPs (50 μM)

was recorded at room temperature using a UV–vis spectrophotometer equipped with a 1 cm quartz cuvette. The absorbance at 660 nm was extracted from the recorded spectra. The ε at 660 nm was calculated according to the Beer–Lambert law.

On page S3-S4 in the revised Supporting Information:

Preparation of Nanoparticles

All nanoparticles (NPs) were prepared by a well-established nanoprecipitation method. Compounds DTPAP-2B, DTPAP-2P or DMPHL-2P, DMPHL-2B (1 mg) and F127 (5 mg) were dissolved respectively into 1 mL of dimethyl sulfoxide (DMSO) and mixed homogeneously. The mixed solution was injected once into 10 mL of double distilled water under ultrasonic conditions for 5 min, then transferred to a dialysis bag (molecular weight cutoff = 3600) and dialyzed against deionized water for 24 h. The as-prepared NPs were concentrated before use. The concentration of these nanoparticles was estimated by UV–Vis absorption spectroscopy using a calibration curve established from DMSO solutions of the corresponding compounds as the reference.

Determination of Molar Extinction Coefficient (ε) of NPs

The molar concentration of NPs was determined using the calibration curve established from the corresponding molecular compound in DMSO. Briefly, a series of standard solutions of the molecular compound in DMSO were prepared, and a calibration curve was constructed by plotting absorbance versus concentration. The absorption spectrum of the NPs dispersed in DMSO was then recorded, and their molar concentration was determined according to the standard calibration curve. Subsequently, the UV–vis absorption spectrum of the NPs in 2 mL PBS (working concentration: 50 μM) was recorded at room temperature using a UV–vis spectrophotometer equipped with a 1 cm quartz cuvette. The absorbance at 660 nm was extracted from the recorded spectrum. The ε at 660 nm was calculated according to the Beer–Lambert law, based on equation (a):

$$\varepsilon = \frac{A}{c \times l} \quad \text{(a)}$$

Where A is the absorbance at 660 nm, c is the molar concentration of the NPs, and l is the optical path length (1 cm).

(13) For the Total ROS Detection by DCFH, 50 μL DCFH (concentration: 40 μM) was mixed with the NPs solution or Ce6 (working concentration: 10 μM). Which volume of solution of NPs or Ce6 was used? The concentration of DCFH cannot be calculated. It is also mentioned that PBS has been used ? Not in figure 1. This part must be strongly revised.

Reply: We sincerely thank the reviewer for the careful evaluation and constructive comments. According to the suggestion, we have revised this part to provide a detailed concentration of all related reagents. In the *Total ROS detection* experiment, stock solutions of DCFH (40 μM) in PBS, Ce6 (1 mM) in DMSO and NPs (1 mM) in aqueous solution were prepared respectively. In a general procedure, 50 μL of DCFH stock solution and 20 μL of Ce6 or NPs stock solution were added to 2 mL PBS buffer (pH 7.4). Therefore, the final concentration of DCFH in the assay system was 1 μM. And the final concentration of Ce6 or NPs in the assay system was 10 μM. We have also revised all the other experimental details accordingly to ensure clarity and reproducibility. We appreciate the reviewer's comment, which has significantly improved the rigor and transparency of our methodology.

On page S5 in the revised Supporting Information:

Total ROS Detection by DCFH

Dichlorodihydrofluorescein (DCFH) was applied to detect ROS. Typically, stock solutions of DCFH (40 μM) in PBS, Ce6 (1 mM) in DMSO and NPs (1 mM) in aqueous solution were prepared respectively. In a general procedure of ROS detection in PBS solution, 50 μL of DCFH stock solution and 20 μL of Ce6 or NPs stock solution were added to 2 mL PBS buffer (pH 7.4). Therefore, the final concentration of DCFH in the assay system was 1 μM. And the final concentration of Ce6 or NPs in the assay system was 10 μM. Control groups were prepared by adding 50 μL DCFH stock solution into 2 mL PBS. After that, the mixtures were exposed to 660 nm irradiation (0.1 W cm^{-2}, spot size 7.5 × 7.5 cm) or white light irradiation (10 mW cm^{-2}), and the PL intensities at ~530 nm were recorded for every five seconds in a PL spectrofluorometer (Excitation wavelength: 489 nm). The detection of ROS generation in DMSO solutions was carried out in similar procedure. And for DMPHL-2P NPs and DMPHL-2B NPs, an 808 nm laser (spot size 6.5 × 4.5 cm) was used instead of the 660 nm laser.

On page 6 in the revised manuscript:

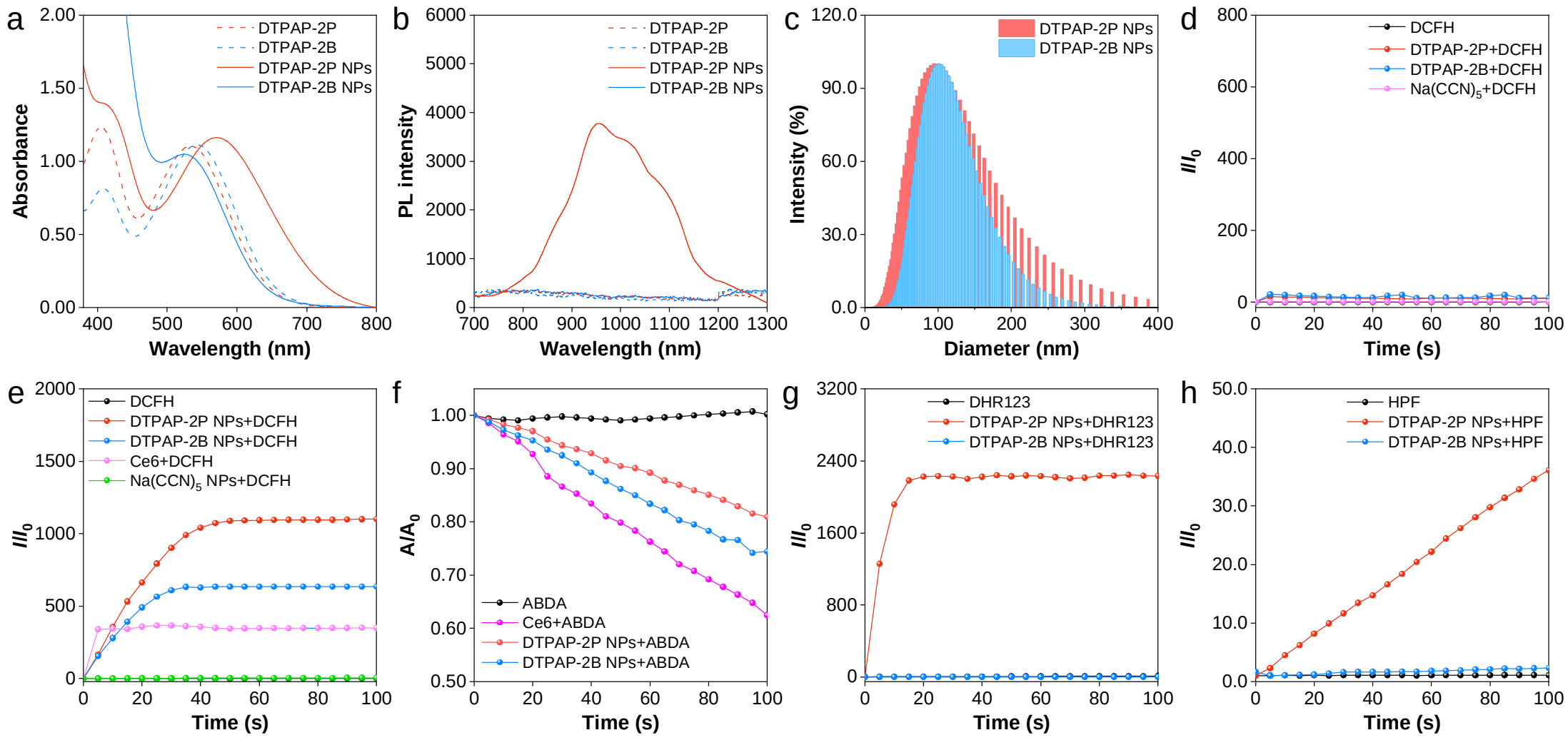

Fig. 1. Photophysical properties and ROS generation of DTPAP-2P and DTPAP-2B. (a) Absorption spectra and (b) Photoluminescence (PL) spectra of DTPAP-2P, DTPAP-2B in DMSO and DTPAP-2P NPs, DTPAP-2B NPs in PBS. λ_{ex}=660 nm. (c) DLS of DTPAP-2P NPs and DTPAP-2B NPs in aqueous solution. (d) ROS generation of DTPAP-2P, DTPAP-2B and $Na(CCN)_5$ (10 μM) in DMSO detected by DCFH upon 660 nm laser irradiation (0.1 W cm^{-2}). (e) ROS generation of DTPAP-2P NPs, DTPAP-2B NPs, Ce6 and $Na(CCN)_5$ NPs (10 μM) in PBS detected by DCFH upon 660 nm laser irradiation (0.1 W cm^{-2}). (f) $^1O_2$ generation of DTPAP-2P NPs, DTPAP-2B NPs and Ce6 in PBS detected by ABDA upon 660 nm laser irradiation (0.1 W cm^{-2}). Type I radical ROS generation of DTPAP-2P NPs, DTPAP-2B NPs (10 μM) in PBS detected by (g) DHR123 and (h) HPF upon 660 nm laser irradiation (0.1 W cm^{-2}).

(14) Page 5, "10 μM of DTPAP-2B NPs or DTPAP-2P NPs were dissolved in 2 mL PBS containing 5 μM of DHR123." Did the authors mean aqueous solution when using PBS? For me, aqueous solution is in pure water. Idem, here the volume of DTPAP-2B NPs or DTPAP-2P NPs is not given.

Reply: We thank the reviewer for the helpful comments which helps us to strengthen the scientific rigor of our manuscript. To avoid ambiguity, we have carefully checked the use of PBS and pure water in all experiments. Accordingly, we have also revised the related expression to clearly demonstrate this point as you suggested.

On page S5-S7 in the revised Supporting Information:

Total ROS Detection by DCFH

Dichlorodihydrofluorescein (DCFH) was applied to detect ROS. Typically, stock solutions of DCFH (40 μM) in PBS, Ce6 (1 mM) in DMSO and NPs (1 mM) in aqueous solution were prepared respectively. In a general procedure of ROS detection in PBS solution, 50 μL of DCFH stock solution and 20 μL of Ce6 or NPs stock solution were added to 2 mL PBS buffer (pH 7.4). Therefore, the final concentration of DCFH in the assay system was 1 μM. And the final concentration of Ce6 or NPs in the assay system was 10 μM. Control groups were prepared by adding 50 μL DCFH stock solution into 2 mL PBS. After that, the mixtures were exposed to 660 nm irradiation (0.1 W cm^{-2}, spot size 7.5 × 7.5 cm) or white light irradiation (10 mW cm^{-2}), and the PL intensities at ~530 nm were recorded for every five seconds in a PL spectrofluorometer (Excitation wavelength: 489 nm). The detection of ROS generation in DMSO solutions was carried out in similar procedure. And for DMPHL-2P NPs and DMPHL-2B NPs, an 808 nm laser (spot size 6.5 × 4.5 cm) was used instead of the 660 nm laser.

Detection of $^1O_2$ Generation by ABDA

$^1O_2$ generation in solution was monitored with ABDA as the indicator, and Ce6 was employed as the standard photosensitizer. Briefly, stock solutions of ABDA (10 mM) in DMSO, Ce6 (1 mM) in DMSO and NPs (1 mM) in aqueous solution were prepared respectively. Then, 10 μL ABDA stock solution and 20 μL stock solutions of DTPAP-2B NPs, DTPAP-2P NPs or Ce6 were added into 2 mL PBS. Thus, the final concentration of ABDA was 50 μM, and the final concentration of Ce6 or NPs in the assay system was 10 μM. The mixture was irradiated by 660 nm laser (0.1 W cm^{-2}, spot size 7.5 × 7.5 cm) or white light irradiation (10 mW cm^{-2}). Then, the absorbance of ABDA at 325-425 nm was recorded for every five seconds. And for DMPHL-2P NPs and DMPHL-2B NPs, an 808 nm laser was used instead of the 660 nm laser. ROS quantum yield of NPs was calculated by the equation (c):

$$\emptyset_{NPs} = \emptyset_{Ce6}\frac{K_{NPs}}{K_{Ce6}}\Big/\frac{A_{NPs}}{A_{Ce6}} \qquad (c)$$

where K_{NPs} and K_{Ce6} are the decomposition rate constants of ABDA with NPs and Ce6, respectively. A_{NPs} and A_{Ce6} represent the light absorbed by NPs and Ce6, respectively, which are determined by integration of the areas under the absorption bands in the wavelength range of 400-800 nm. Φ_{Ce6} is the $^1O_2$ quantum yield of Ce6, which is 0.65 in water.[S11-S12]

Detection of $O_2^{\bullet-}$ Generation by DHR123

Compound DHR123 was used as indicator for detection of $O_2^{\bullet-}$ in solution. When $O_2^{\bullet-}$ is generated in the system, DHR123 will be oxidized to emit strong fluorescence at 530 nm. Briefly, stock solutions of DHR123 (5 mM) in DMSO, Ce6 (1 mM) in DMSO and NPs (1 mM) in aqueous solution were prepared respectively. Then, 2 μL DHR123 stock solution and 20 μL stock solutions of DTPAP-2B NPs, DTPAP-2P NPs or Ce6 were added into 2 mL PBS. Thus, the final concentration of ABDA was 5 μM, and the final concentration of Ce6 or NPs in the assay system was 10 μM. The mixture was then placed in a cuvette and irradiated by 660 nm laser (0.1 W cm^{-2}, spot size 7.5 × 7.5 cm) or white light irradiation (10 mW cm^{-2}). And for DMPHL-2P NPs and DMPHL-2B NPs, an 808 nm laser was used instead of the 660 nm laser. The fluorescence changes of sample at 530 nm were recorded by the PL spectrofluorometer (Excitation wavelength: 480 nm).

Detection of •OH Generation by HPF

Compound HPF was used as indicator for detection of •OH in solution. When •OH is generated in the system, HPF will be oxidized to emit strong fluorescence at 515 nm. Briefly, stock solutions of HPF (5 mM) in DMSO, Ce6 (1 mM) in DMSO and NPs (1 mM) in aqueous solution were prepared respectively. Then, 2 μL HPF stock solution and 20 μL stock solutions of DTPAP-2B NPs, DTPAP-2P NPs or Ce6 were added into 2 mL PBS. Thus, the final concentration of ABDA was 5 μM, and the final concentration of Ce6 or NPs in the assay system was 10 μM. The mixture was then placed in a cuvette and irradiated by 660 nm laser (0.1 W cm^{-2}, spot size 7.5 × 7.5 cm) or white light irradiation (10 mW cm^{-2}). And for DMPHL-2P NPs and DMPHL-2B NPs, an 808 nm laser was used instead of the 660 nm laser. The fluorescence changes of sample at 530 nm were recorded by the PL spectrofluorometer (Excitation wavelength: 490 nm).

REVIEWER COMMENTS

Reviewer #1 (Remarks to the Author):

I have carefully reviewed the revised manuscript (NCOMMS-26-005084A) and the authors' point-by-point response to my previous comments. I am pleased to note that the authors have addressed all the issues raised in the first round of review in a thorough and convincing manner.

Specifically, the additional data on nanoparticle size distribution and colloidal stability in biological media (Response to Comment 1), the clarified discussion distinguishing anion–π^+ interactions from general counterion effects (Response to Comment 2), and the expanded comparison with previously reported anion–π^+ photosensitizers (Response to Comment 3) have significantly strengthened the manuscript. The newly provided photothermal cycling experiments and comparison with a commercial NIR dye (Response to Comment 4), along with the complementary ROS probe assays (Response to Comment 5) and hemocompatibility evaluation (Response to Comment 6), provide solid experimental support for the conclusions and demonstrate the robustness and translational potential of the proposed system.

The revised manuscript is well organized, methodologically rigorous, and meets the high standards expected for publication in Nature Communications. The results remain highly novel and noteworthy: the strategic use of an organic planar π-conjugated anion to construct anion–π^+ type NIR-II phototheranostic agents represents an important conceptual advance over conventional approaches relying on inorganic counteranions. This work not only fills a critical gap in the development of NIR-II anion–π^+ systems but also provides valuable design principles that are likely to inspire further research in the field of organic phototheranostics.

In my judgment, the authors have satisfactorily addressed all concerns, and no further revision is required. I recommend that the manuscript be accepted for publication in its current form.

Reply: We sincerely thank the reviewer for recognizing the revision of our manuscript. We also appreciate a lot for the reviewer's precious time to review our manuscript and make professional and constructive suggestions to improve further the quality of our manuscript.

Reviewer #2 (Remarks to the Author):

The authors have properly addressed and corrected all the points raised by the reviewers. The article is now fully consistent with the claims and can be published.

Reply: We sincerely thank the reviewer for recognizing the revision of our manuscript. We also appreciate a lot for the reviewer's precious time to review our manuscript and for helping us enhance both the accuracy and the clarity of our work. We have also revised the manuscript according to the additional comments.

Minor corrections:

(1) Please add the storage temperature on Figure S21 and S22.

Reply: We thank the reviewer for the careful reading and for pointing out these issues. The storage temperature is 4 °C in a fridge. We have added this detail on Figure S21 and S22 (now figures S25 and S26).

On page S13-S14 of the Supplementary Information:

Supplementary Figure 25. (**a**) Size stability of DTPAP-2P NPs and DTPAP-2B NPs in H_2O during 7 days of storage at 4 °C. (**b**) The UV-Vis absorption and fluorescence spectra of the DTPAP-2P NPs solution during 7 days of storage at 4 °C. (**c**) Time-dependent absorbance variation of DTPAP-2P NPs at 572 nm. (**d**) Time-dependent emission intensity of DTPAP-2P NPs at 950 nm under 660 nm excitation. Data in (**a**) were presented as mean values ± SEM.

Supplementary Figure 26. Size stability of DTPAP-2P NPs and DTPAP-2B NPs in dulbecco's modified eagle medium (DMEM) during 7 days of storage at 4 °C. Data were presented as mean values ± SEM.

(2) ABDA is also instead of DHR123 or HPF in the experimental descriptions. Revised.

Reply: We thank the reviewer for the careful reading and feel really sorry for this mistake. We have revised these details in Methods of the manuscript (To meet the journal's requirements, the detailed Methods have been moved to the main text).

On page 27-28 of the main text:

Detection of $O_2^{\bullet-}$ generation by DHR123

Compound DHR123 was used as indicator for detection of $O_2^{\bullet-}$ in solution. When $O_2^{\bullet-}$ is generated in the system, DHR123 will be oxidized to emit strong fluorescence at 530 nm. Briefly, stock solutions of DHR123 (5 mM) in DMSO, Ce6 (1 mM) in DMSO and NPs (1 mM) in aqueous solution were prepared respectively. Then, 2 μL DHR123 stock solution and 20 μL stock solutions of DTPAP-2B NPs, DTPAP-2P NPs or Ce6 were added into 2 mL PBS. Thus, the final concentration of DHR123 was ~5 μM, and the final concentration of Ce6 or NPs in the assay system was ~10 μM. The mixture was then placed in a

Detection of •OH generation by HPF

Compound HPF was used as indicator for detection of •OH in solution. When •OH is generated in the system, HPF will be oxidized to emit strong fluorescence at 515 nm. Briefly, stock solutions of HPF (5 mM) in DMSO, Ce6 (1 mM) in DMSO and NPs (1 mM) in aqueous solution were prepared respectively. Then, 2 μL HPF stock solution and 20 μL stock solutions of DTPAP-2B NPs, DTPAP-2P NPs or Ce6 were added into 2 mL PBS. Thus, the final concentration of HPF was ~5 μM, and the final concentration of Ce6 or NPs in the assay system was ~10 μM. The mixture was then placed in a cuvette and irradiated

(3) I still do not agree with the expression "the molar concentration of NPs," because this actually corresponds to the molar concentration of the molecular compound in the NP suspension rather than the molar concentration of nanoparticles themselves. The exact nanoparticle concentration (i.e., the exact particle count or number of nanoparticles per unit volume) has not been determined. Therefore, throughout the manuscript, the correct terminology should be "molar concentration of the molecular compound in the NP suspension."

Consequently, the molar extinction coefficient (ε) of the nanoparticles themselves cannot be accurately determined, since the nanoparticle molar concentration is unknown.

For example, when the manuscript states "NPs in 2 mL PBS (working concentration: 50 μM)," this concentration actually refers to DTPAP-2B or DTPAP-2P in the nanoparticle suspension, not to the nanoparticles themselves.

"Notably, the maximum absorption peaks of DTPAP-2P NPs were obviously redshifted from 535 nm to 570 nm, with its molar extinction coefficient at 660 nm calculated to be 1.16 ×104 L mol-1 cm-1", this is the molar extinction coefficient of DTPAP-2P aggregated into nanoparticles and not

the one of a nanoparticle. The sentence should be ""Notably, the maximum absorption peaks of DTPAP-2P in NPs were obviously redshifted from 535 nm to 570 nm, with its molar extinction coefficient at 660 nm calculated to be 1.16 ×104 L mol-1 cm-1".

Remark:

"Thus, the final concentration of ABDA was 5 μM, and the final concentration of Ce6 or NPs in the assay system was 10 μM." Should be "Thus, the final concentration of ABDA was ~5 μM, and the final concentration of Ce6 or NPs in the assay system was ~10 μM." as the final concentration is 2.022 mL and not 2mL.

Reply: We thank the reviewer for the careful reading to help us further improve the rigorous of our manuscript. We have thoroughly checked the main text and the supplementary information to revise these details.

On page 24-25 in the main text:

Determination of molar extinction coefficient of the molecular compound in the NP suspension

Molar concentration of the molecular compound in the NP suspension was determined using the calibration curve established from the corresponding molecular compound in DMSO. Briefly, a series of standard solutions of the molecular compound in DMSO were prepared, and a calibration curve was constructed by plotting absorbance versus concentration. The absorption spectrum of the NPs dispersed in DMSO was then recorded, and molar concentration of the molecular compound in the NP suspension was determined according to the standard calibration curve. Subsequently, the UV–vis absorption spectrum of DTPAP-2B or DTPAP-2P in the nanoparticle suspension (2 mL PBS, working concentration: 50 μM) was recorded at room temperature using a UV–vis spectrophotometer equipped with a 1 cm quartz cuvette. The absorbance at 660 nm was extracted from the recorded spectrum. The ε at 660 nm was

On page 6 in the main text:

were then investigated. Compared with the absorption spectra in DMSO solution, the maximum absorption wavelength of DTPAP-2B NPs displayed slight hypsochromic shift of 17 nm (Fig. 2a). Notably, the maximum absorption peaks of DTPAP-2P in NPs were obviously redshifted from 535 nm to 570 nm, with its molar extinction coefficient at 660 nm calculated to be 1.16×10^4 L mol^{-1} cm^{-1}, which is conducive to better light harvesting capability in longer wavelength window. More surprisingly, under 660 nm laser irradiation, DTPAP-2P NPs presented remarkably enhanced broad fluorescence emission in

On page 27-28 of the main text:

Detection of $O_2^{\bullet-}$ generation by DHR123

Compound DHR123 was used as indicator for detection of $O_2^{\bullet-}$ in solution. When $O_2^{\bullet-}$ is generated in the system, DHR123 will be oxidized to emit strong fluorescence at 530 nm. Briefly, stock solutions of DHR123 (5 mM) in DMSO, Ce6 (1 mM) in DMSO and NPs (1 mM) in aqueous solution were prepared respectively. Then, 2 µL DHR123 stock solution and 20 µL stock solutions of DTPAP-2B NPs, DTPAP-2P NPs or Ce6 were added into 2 mL PBS. Thus, the final concentration of DHR123 was ~5 µM, and the final concentration of Ce6 or NPs in the assay system was ~10 µM. The mixture was then placed in a

Detection of •OH generation by HPF

Compound HPF was used as indicator for detection of •OH in solution. When •OH is generated in the system, HPF will be oxidized to emit strong fluorescence at 515 nm. Briefly, stock solutions of HPF (5 mM) in DMSO, Ce6 (1 mM) in DMSO and NPs (1 mM) in aqueous solution were prepared respectively. Then, 2 µL HPF stock solution and 20 µL stock solutions of DTPAP-2B NPs, DTPAP-2P NPs or Ce6 were added into 2 mL PBS. Thus, the final concentration of HPF was ~5 µM, and the final concentration of Ce6 or NPs in the assay system was ~10 µM. The mixture was then placed in a cuvette and irradiated
